# Creating bottom-up RNA transfer vehicles from synthetic protein assemblies

**DOI:** 10.1038/s41586-026-10952-3

**Published:** 2026-09-02

**Authors:** Maren Kirstin Schuhmacher, Christoph Gruber, Christopher M. R. Lang, Ricardo M. W. Ruijpers, Lyupka Mazneykova, Brice Beinsteiner, Ariane Krus, Barbara Tremmel, Friederike Reinhardt, Karoline Kadletz, Zhe Ma, Lucie Casalta, Josep Miquel Cambra Bort, Dina Y. Otify, Iolo Balken, Leon Hetzel, Juliane Merl-Pham, Tatjana Dorn, Marina Luchner, Lea Bauersachs, Karin Ganea, Natascha Wieser, Alexander Emrich, Emirhan Yağmur, Katrin Rager, Gauhar Sagindykova, Niklas Armbrust, Julian Geilenkeuser, Gil G. Westmeyer, Elvir Becirovic, Martin Biel, Rouzanna Istvanffy, Daniela M. Vogt Weisenhorn, Dong-Jiunn Jeffery Truong, Fabian J. Theis, Gregor Ebert, Alessandra Moretti, Ali Ertürk, Andrea Bähr, Christian Kupatt, Marion Jasnin, Nikolai Klymiuk, Florian Giesert, Wolfgang Wurst

**Affiliations:** 1 Institute of Developmental Genetics, Helmholtz Munich, Oberschleissheim, Germany; 2 TUM School of Life Sciences, Technical University of Munich, Freising, Germany; 3 Helmholtz Pioneer Campus, Helmholtz Munich, Oberschleissheim, Germany; 4 Department of Bioscience, TUM School of Natural Sciences, Technical University of Munich, Garching, Germany; 5 Institute for Tissue Engineering and Regenerative Medicine, Helmholtz Munich, Oberschleissheim, Germany; 6 Deep Piction, Munich, Germany; 7 Institute of Virology, Helmholtz Munich, Oberschleissheim, Germany; 8 Large Animal Models in Cardiovascular Research, Klinik und Poliklinik für Innere Medizin I, Klinikum rechts der Isar, Technical University Munich, Munich, Germany; 9 Center for Innovative Medical Models, LMU Munich, Munich, Germany; 10 Department of Pharmacy-Center for Drug Research, LMU Munich, Munich, Germany; 11 Institute of Computational Biology, Helmholtz Munich, Oberschleissheim, Germany; 12 Department of Mathematics, TUM School of Natural Sciences, Technical University of Munich, Garching, Germany; 13 Metabolomics and Proteomics Core, Helmholtz Munich, Oberschleissheim, Germany; 14 First Department of Medicine and Regenerative Medicine in Cardiovascular Diseases, Klinikum rechts der Isar, Munich, Germany; 15 TUM School of Medicine and Health, Technical University of Munich, Munich, Germany; 16 Institute for Synthetic Biomedicine, Helmholtz Munich, Oberschleissheim, Germany; 17 Laboratory for Retinal Gene Therapy, Department of Ophthalmology, University Hospital Zurich, University of Zurich, Schlieren, Switzerland; 18 Department of Surgery, TUM University Hospital Rechts der Isar, Munich, Germany; 19 Wellcome Trust Sanger Institute, Wellcome Genome Campus Hinxton, Saffron Walden, UK; 20 Institute for Stroke and Dementia Research, Medical Centre of the University of Munich, LMU Munich, Munich, Germany; 21 Cluster for Systems Neurology (SyNergy), Munich, Germany; 22 Graduate School of Neuroscience (GSN), Munich, Germany; 23 Klinik und Poliklinik für Innere Medizin I, Klinikum rechts der Isar, Technical University of Munich, Munich, Germany; 24 DZHK (German Center for Cardiovascular Research), partner site Munich Heart Alliance, Munich, Germany; 25 Department of Chemistry, TUM School of Natural Sciences, Technical University of Munich, Garching, Germany; 26 Helmholtz Munich Cryo-Electron Microscopy Platform, Helmholtz Munich, Oberschlessheim, Germany; 27 Institute of Stem Cell Research, Helmholtz Munich, Oberschleissheim, Germany; 28 Deutsches Zentrum für Psychische Gesundheit (DZPG), Site Munich-Augsburg, Munich, Germany; 29 German Center for Neurodegenerative Diseases (DZNE), Munich, Germany; 30 Emeritus of Excellence Senior Faculty (SEF), Technical University of Munich, Munich, Germany

**Keywords:** Protein design, Gene delivery, Gene therapy

## Abstract

Evolution guides biological systems to populate ecological niches, with viruses among the most successful examples of this principle. Viruses evolved over billions of years to efficiently transfer genetic information. Although viruses are highly diverse, most have converged towards remarkable similarity in the size and shape of their capsids^1,2^. By contrast, generative models for protein design enable the creation of protein architectures that are absent from nature^3–5^. Here we investigate whether protein assemblies designed by artificial intelligence can be functionalized to construct nucleic acid transport vehicles that are independent of evolutionary trajectories. By combining natural protein domains with synthetic protein assemblies, we create more than 100 bottom-up RNA transfer vehicles with unique sizes and shapes. These vehicles surpass the RNA transfer efficiency of widely used delivery vehicles by several orders of magnitude. In addition, we demonstrate that their tropism can be programmed by incorporation of computationally designed peptide binders and use them to deliver therapeutically relevant cargo RNAs into a wide range of cellular models. We show the in vivo biodistribution of one of these vehicles in a mouse at near-single-cell resolution, confirm its safety, and use it to perform a gene-editing treatment strategy for Duchenne muscular dystrophy in patient-derived cells and a pig. Our work demonstrates how proteins created by generative artificial intelligence can be harnessed for the rational engineering of RNA transport systems with the desired properties by overcoming the limitations of natural protein diversity.

## Abstract (web summary)

Synthetic virus-like protein architectures designed by artificial intelligence show superior ability to deliver RNA into cells, compared with their naturally occurring counterparts, by overcoming the evolutionary constraints limiting viral evolution.

## Main

Selective pressure drives biological systems towards a local minimum on the evolutionary landscape, enabling them to occupy an ecological niche^6–8^. Viruses, for instance, are highly optimized vehicles for gene transfer; however, despite their diversity, they have converged on similar features. Most viruses rely on large supramolecular protein capsids composed of thousands of subunits, which self-assemble mostly into icosahedral or helical symmetries to enclose and protect their genome^1,2,9,10^. Viral capsids are selected for their resilience in harsh environmental conditions. However, when repurposed as vectors for genetic engineering, they are handled in controlled environments. This raises the question of whether certain features selected for by evolution may be unnecessary or even disadvantageous when a biological system is placed in a context outside its original ecological niche. Recently developed artificial intelligence models for protein design can be harnessed to explore this question. These models create protein structures that are physically feasible but do not occur naturally^3–5,11,12^, enabling the manipulation of evolutionary trajectories with non-natural protein architectures.

Here we exemplify this idea by constructing bottom-up RNA transfer vehicles consisting of natural protein domains, combined with artificial-intelligence-designed synthetic protein assemblies. We name these RNA carriers synthetic transfer vehicles (STVs). STVs are distinct from known natural RNA transfer vehicles, exhibiting unique characteristics that include cyclic and dihedral symmetries, open structures and low complexity of the assembled protein. We develop a multidimensional screening system that enables testing of hundreds of designs and identify STV-C8, which is built from an unusual planar symmetry, as the most efficient structure for RNA delivery. We characterize the shape, content and packaging capacity of STV-C8 and program its tropism by combining it with computationally designed peptide binders. Regardless of its distinct structure, STV-C8 is several orders of magnitude more efficient in RNA transfer compared with its natural counterparts and with lipid nanoparticles (LNPs) in clinical use. We demonstrate the versatility of STV-C8 by delivering various cargo RNAs, including reporter RNAs, gene editors, programmable antivirals and transcription factors, into a wide variety of cellular models from several species. We perform a comprehensive in vivo biodistribution analysis of STV-C8 at near-single-cell resolution in a mouse model and confirm its excellent safety profile in two animal models. Finally, we evaluate the translational capacity of STV-C8 by delivering the CRISPR–Cas9 gene editor into the patient-derived and pig skeletal muscle cells to delete dystrophin exon 51 as a treatment strategy for Duchenne muscular dystrophy (DMD)^13^.

Capsid-forming proteins of enveloped viruses typically consist of multiple domains that orchestrate the packaging of genetic material, as well as the assembly and release of the capsid at the plasma membrane of infected cells. The curved surface of assembled viral capsids induces the first step of vesicle release by membrane bending, and it has been proposed that partially assembled protomers, as well as the fully assembled capsid multimer, can induce membrane bending^14,15^. This proposal raises the question of whether such a mechanism could be harnessed to create RNA transfer vehicles from scratch using simple, low-dimensional protein multimers. To explore this possibility, we leverage artificial-intelligence-designed symmetric protein assemblies with various symmetries to build hundreds of diverse vehicles in a bottom-up approach.

## Screening of synthetic assemblies

**Fig. 1:**
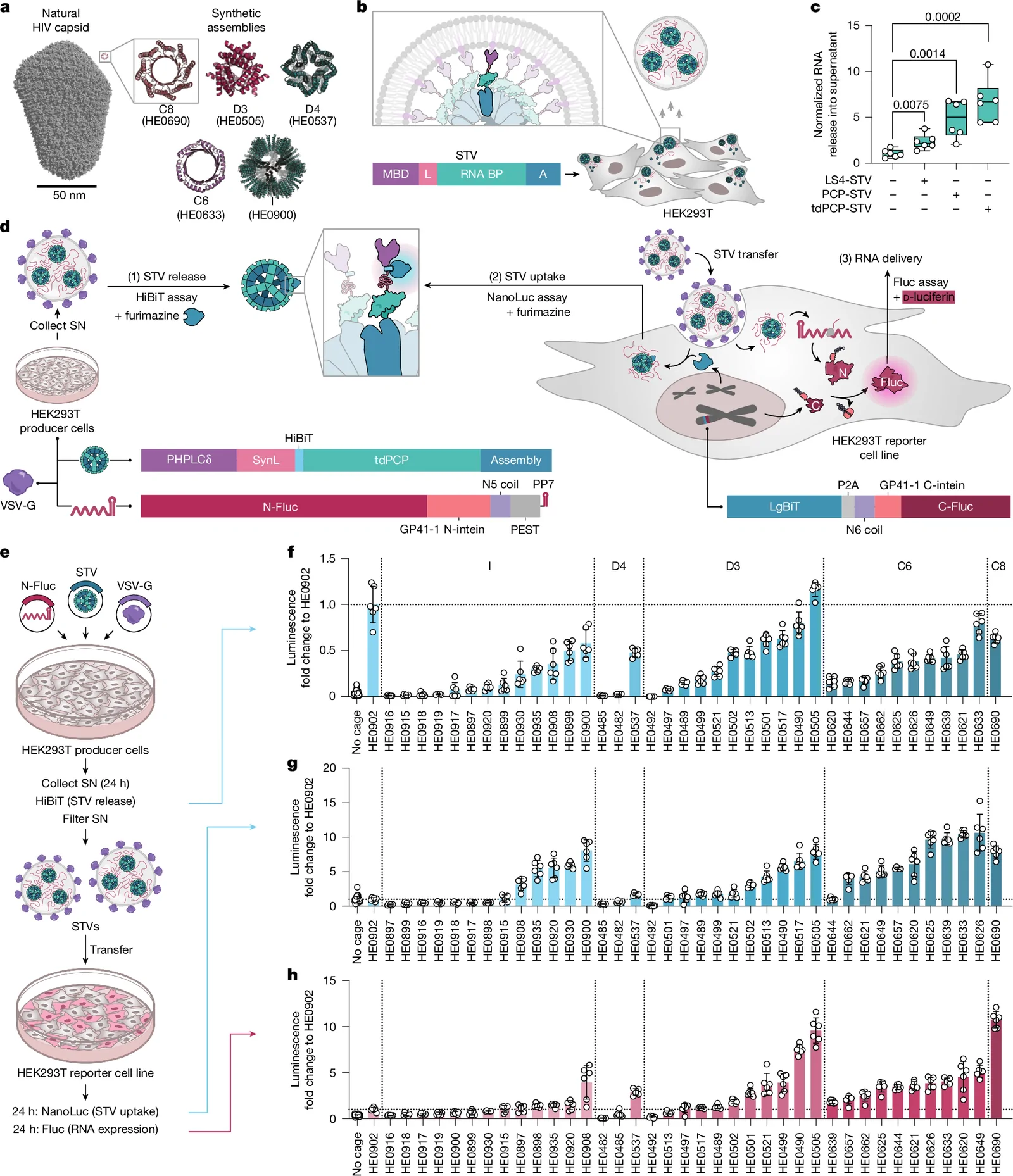
Design and screening of bottom-up-assembled STV RNA carrier. **a**, Size and shape comparison of viral and artificial-intelligence-designed protein assemblies. Representative icosahedral (I), dihedral (D) and cyclic (C) symmetries are shown. **b**, Schematic of the STV architecture and the principle of mimicking viral release and RNA packaging by expressing synthetic protein assemblies in cells. **c**, RT–qPCR quantification of RNA release by HE0902-based STV constructs, consisting of different RNA-binding proteins (in box plots, the centre line represents the median, the box represents the interquartile range, and whiskers represent the minimum and maximum values; two-sided unpaired Student’s *t*-test; *n* = 6 biological replicates). **d**, Screening scheme for STV release by HiBiT assay in supernatant, STV uptake by LgBiT–HiBiT interaction in reporter cells and RNA delivery efficiency based on reconstitution of split-Fluc in reporter cells. **e**, Experimental workflow for testing of STV release, uptake and RNA expression over the course of 72 h. **f**, Luminescence measurements of HiBiT signal in the supernatant of producer cells to quantify the release of STV constructs containing different artificial-intelligence-designed assembly domains (mean ± s.d. for *n* = 6 biological replicates). **g**, Luminescence measurements in the lysate of LgBiT-expressing split-Luc reporter cells to test for uptake of different STV constructs by reconstituting NanoLuc from genomically expressed LgBiT and STV containing HiBiT (mean ± s.d. for *n* = 6 biological replicates). **h**, Firefly measurements in the lysate of C-split-Fluc-expressing reporter cells to quantify N-split-Fluc RNA delivery efficiency from different STV constructs (mean ± s.d. for *n* = 6 biological replicates). Colours of arrows from **e** to **f**,**g**,**h**, correspond to the wavelength emitted by the luciferases. A, assembly domain; L, late-budding domain; MBD, membrane-binding domain; RNA BP, RNA-binding protein. Source Data

**Extended Data Fig. 1:**
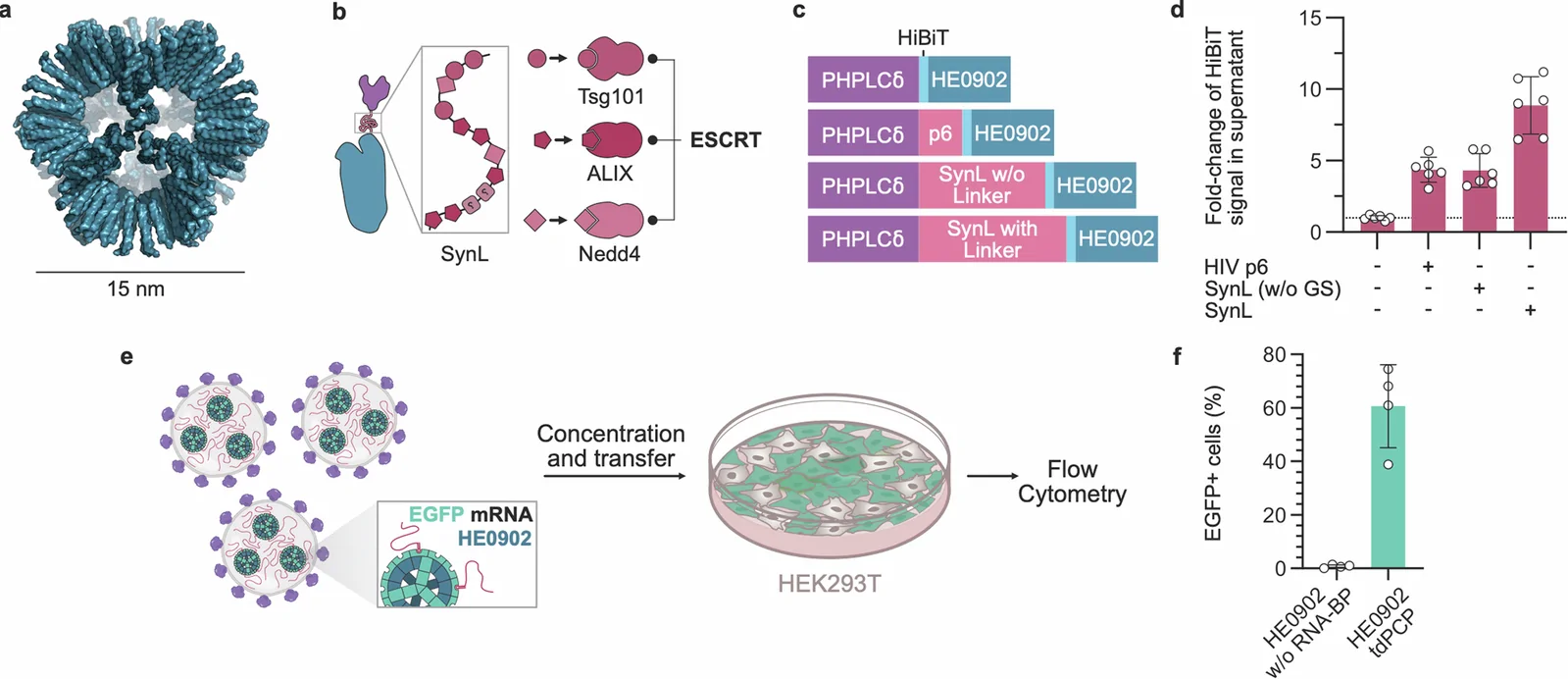
Detailed characterization of fundamental STV domains. **a**, Solved structure and size of the initial assembly domain HE0902^3^. **b**, Design concept of the synthetic budding domain SynL, derived from ESCRT recruiting viral peptides. **c**, Basic STV design containing different budding domains tested in panel d. **d**, HiBiT-based quantification of STV release by fusing different budding domains (mean ± s.d. for n = 6 biological replicates). **e**, Schematic illustration of EGFP mRNA delivery by basic STV construct. **f**, EGFP mRNA delivery into HEK293T cells, mediated by original STV construct with SynL domain, quantified by Flow Cytometry (mean ± s.d. for n = 4 biological replicates). Source Data

Generative models for protein design, such as RFdiffusion^3^, can generate virtually infinite numbers of protein assemblies with various shapes, including icosahedral, dihedral and cyclic symmetries, which differ greatly in size and architecture compared with natural capsids (Fig. 1a). To implement such synthetic protein assemblies as bottom-up RNA transfer vehicles, we fused them to three functional domains that typically form part of capsid-forming proteins: a membrane-binding domain, a late-budding domain and an RNA-binding domain^15^ (Fig. 1b). We used HE0902, a well-characterized icosahedral protein assembly^3^ (Extended Data Fig. 1a) as the initial scaffold for such STV carriers and fused it to a membrane-binding domain derived from the pleckstrin homology domain of *Rattus norvegicus* phospholipase C delta (PHPLCδ). In addition, we created a synthetic budding domain composed of budding motifs from several viruses. The budding efficiency of this synthetic late-budding domain (SynL) exceeded that of the natural HIV p6 L-domain (Extended Data Fig. 1b–d). To enable RNA packaging, we added high-affinity RNA-binding proteins to the construct^16,17^. We transfected HEK293T cells with these initial STV constructs, along with RNAs containing the corresponding packaging signal, and quantified the STV-mediated target RNA release into the cell culture supernatant. All constructs successfully transferred their RNA cargo into the supernatant, and the STV construct built on tandem PCP (tdPCP) was the most efficient (Fig. 1c). Furthermore, we demonstrated that co-expression of VSV-G as a fusogenic protein enabled these synthetic vesicles to deliver *EGFP* cargo RNA into target cells (Extended Data Fig. 1e,f).

**Extended Data Fig. 2:**
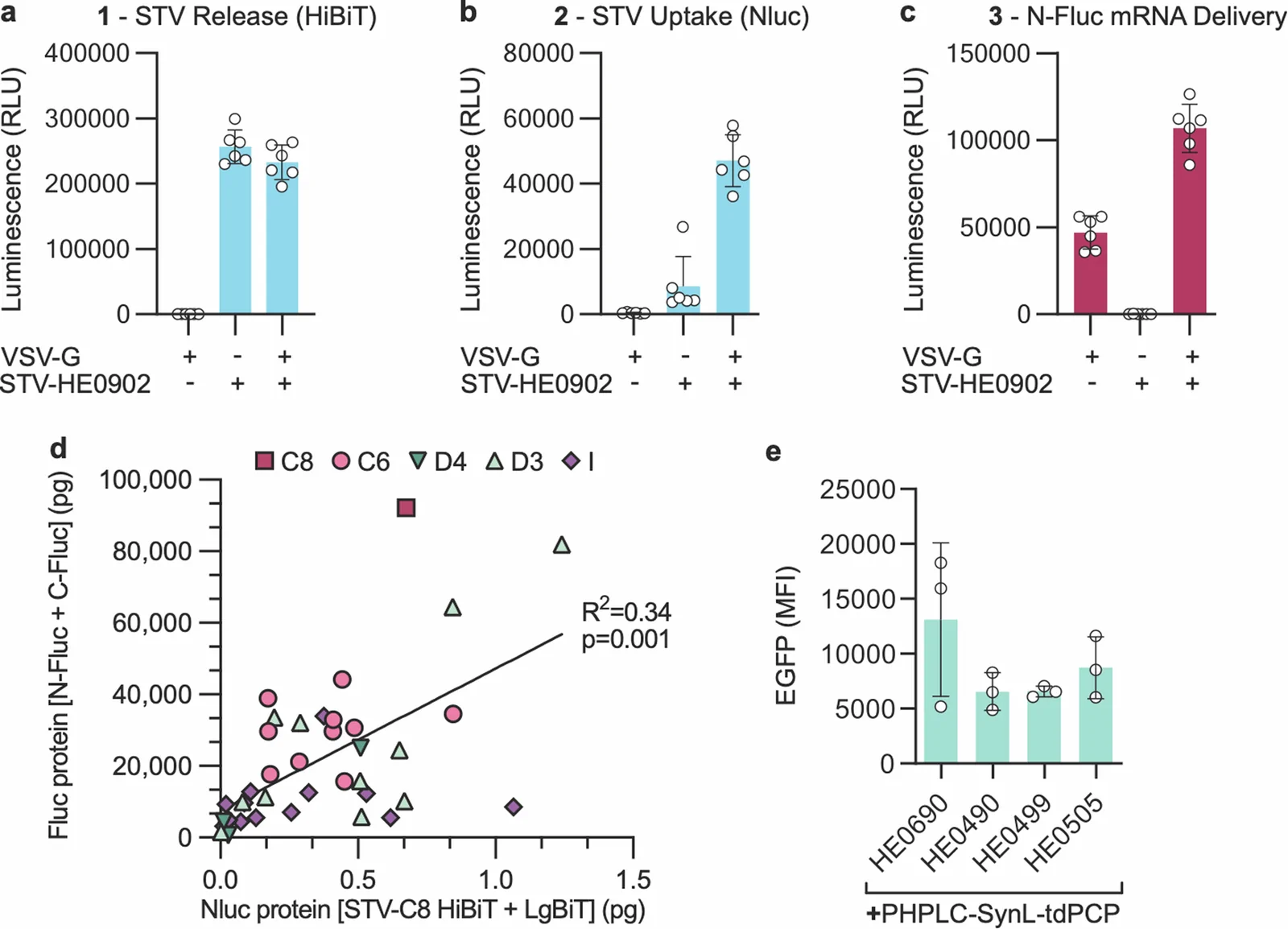
Establishment and validation of the dual Split-Luciferase based screening method. **a**, Analysis of HE0902-based STV release by measuring HiBiT signal in the supernatant of producing cells (mean ± s.d. for n = 6 biological replicates). **b**, Quantification of HE0902-based STV uptake in C-Split-Luc reporter cells by measuring NanoLuc signal in cell lysates (mean ± s.d. for n = 6 biological replicates). **c**, Measurement of STV-HE0902 mediated N-Split-Luc mRNA delivery into C-Split-Luc reporter cells by quantifying Firefly Luciferase signal in cell lysates (mean ± s.d. for n = 6 biological replicates). **d**, Correlation analysis of STV release into the supernatant and mRNA delivery efficiency into target cells for different STV symmetries (C, cyclic; D, dihedral; I, icosahedral). Both dimensions are measured as total protein content by standardizing against Fluc or Nluc reference proteins (Pearson correlation, two-sided, each data point shown as mean from n = 6 biological replicates). **e**, Flow Cytometry-based validation of Split-Luc screening results by delivering EGFP mRNA into HEK293T cells (mean ± s.d. for n = 3 biological replicates). Source Data

HE0902 has icosahedral symmetry similar to that of many viral capsids. Encouraged by the initial proof that STV-HE0902 enabled efficient RNA release and transfer, we explored embedding non-natural symmetries into the STV scaffold. To comprehensively characterize these vehicles, we developed a screening method to enable monitoring of three relevant dimensions: (1) STV release, (2) STV uptake, and (3) RNA transfer efficiency. In brief (a detailed description is provided in Supplementary Note 1), STV constructs were fused to a HiBiT tag, enabling us to quantify release of STV into the supernatant by complementing HiBiT with recombinant LgBiT protein. For characterization of uptake efficiency, we created a reporter cell line expressing LgBiT and used it to measure STV uptake by HiBiT/LgBiT complementation in target cells. In addition, the reporter cells expressed the C-split part of firefly luciferase (C-split-Fluc). STV-mediated delivery of mRNA encoding the N-split part of Fluc (N-split-Fluc) enabled quantification of mRNA delivery efficiency through reconstitution of full-length Fluc (Fig. 1d and Extended Data Fig. 2a–c).

**Extended Data Fig. 3:**
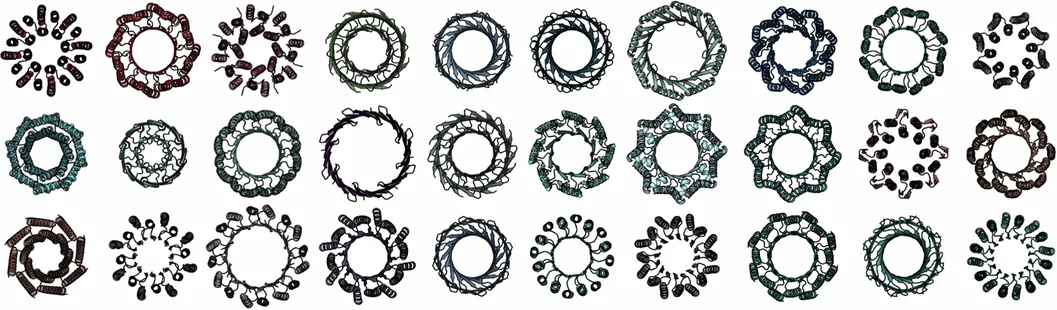
Additional C8 symmetric assemblies. Predicted design of additional protein assemblies by running RFdiffusion with C8 symmetry constraints.

Building on the established method, we screened 39 STV constructs consisting of synthetic assembly domains with icosahedral, dihedral or cyclic symmetries (Fig. 1e). Many of these STV constructs were efficiently released from producer cells and delivered their cargo RNA into target cells. Notably, STVs built on non-natural dihedral and cyclic symmetries largely outperformed their icosahedral counterparts (Fig. 1f–h). We also found a correlation between the efficiency of STV release and cargo RNA delivery. STVs built on cyclic symmetries transferred more RNA per packaging protein, indicating greater RNA loading capacity than that of other symmetries, such as icosahedral (Extended Data Fig. 2d). We confirmed high delivery efficiency for another cargo RNA, *EGFP* mRNA, into unmodified target cells with the four best-performing designs: HE0490, HE0499 and HE0505 (all D3 symmetry) and HE0690 (C8 symmetry) (Extended Data Fig. 2e). These results indicate that artificial-intelligence-designed protein assemblies with non-natural symmetries could be harnessed for creation of virtually infinite numbers of synthetic RNA delivery vehicles. To demonstrate this large potential for scaling, we designed a further 30 assemblies with C8 symmetry, the symmetry of the best-performing structure HE0690 (Extended Data Fig. 3). Although they were built on the same C8 symmetry, these newly created structures were highly diverse in sequence and structural composition, allowing them to be screened for custom characteristics, such as altered packaging density or low immunogenicity.

**Fig. 2:**
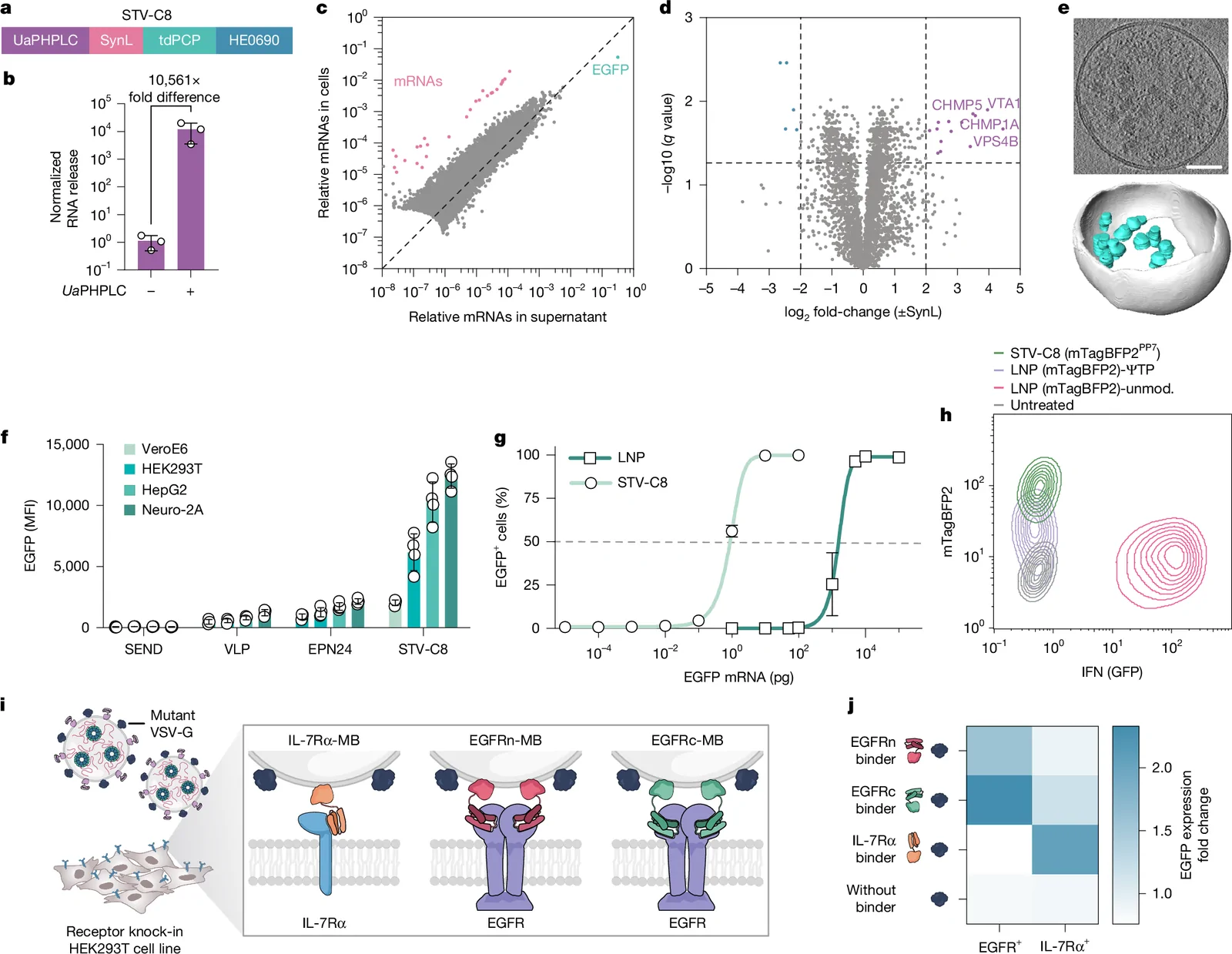
Characterization and programming of the STV-C8 RNA carrier. **a**, Optimized STV-C8 construct consisting of the pleckstrin homology domain from *Ua*PHPLC, a synthetic budding domain derived from viral ESCRT recruiting motifs (SynL), a tandem coat protein from *Pseudomonas* phage PP7 (tdPCP) and an artificial-intelligence-designed assembly domain with C8 symmetry (HE0690). **b**, RT–qPCR of target RNA released into the supernatant by STV-C8 relative to a membrane-binding-deficient control (mean ± s.d.; *n* = 3 biological replicates). **c**, RNA sequencing correlations of producer cells and STV-C8 (*n* = 4 biological replicates). For each mRNA, the read counts in supernatant and cytosol relative to all mRNAs are shown (red indicates mitochondrial RNAs and green the EGFP cargo). **d**, Proteomic comparison of purified supernatants from budding (+SynL) and non-budding (−SynL) STV-C8 constructs (purple indicates ESCRT-related proteins; *n* = 3 biological replicates). **e**, Representative 6.52-Å-thick slice from a tomographic volume of a STV-C8 vesicle (top; scale bar, 50 nm) and corresponding three-dimensional segmentation (bottom) showing the STV membrane (grey) and STV-C8 protein assemblies (cyan). **f**, Comparison of *EGFP* mRNA delivery by STV-C8 and other genetically encoded delivery vehicles, enveloped with VSV-G, into different target cell lines (quantified by flow cytometry; each mRNA was tagged with its corresponding packaging signal; mean ± s.d. for *n* = 4 biological replicates). **g**, EGFP-positive cells as a function of *EGFP* mRNA input delivered by STV-C8 or LNPs (STV-C8 RNA content was quantified by absolute RT–qPCR; mean ± s.d. for *n* = 3 biological replicates). **h**, IFNβ reporter activation in A549 cells after delivery of cellularly transcribed STV-C8-packaged mTagBFP2 mRNA or *N*^1^-methylpseudouridine-modified or unmodified LNP-packaged mRNA. mRNA doses were adjusted to achieve comparable expression. **i**, Concept for programming STV-C8 cell-type specificity by incorporating artificial-intelligence-designed minibinders. **j**, Flow cytometry quantification of *EGFP* mRNA delivery into HEK293T EGFR or IL-7Rα knock-in cell lines using mutant VSV-G together with receptor-targeting minibinders (mean of *n* = 3 biological replicates). MB, minibinders. Source Data

**Extended Data Fig. 4:**
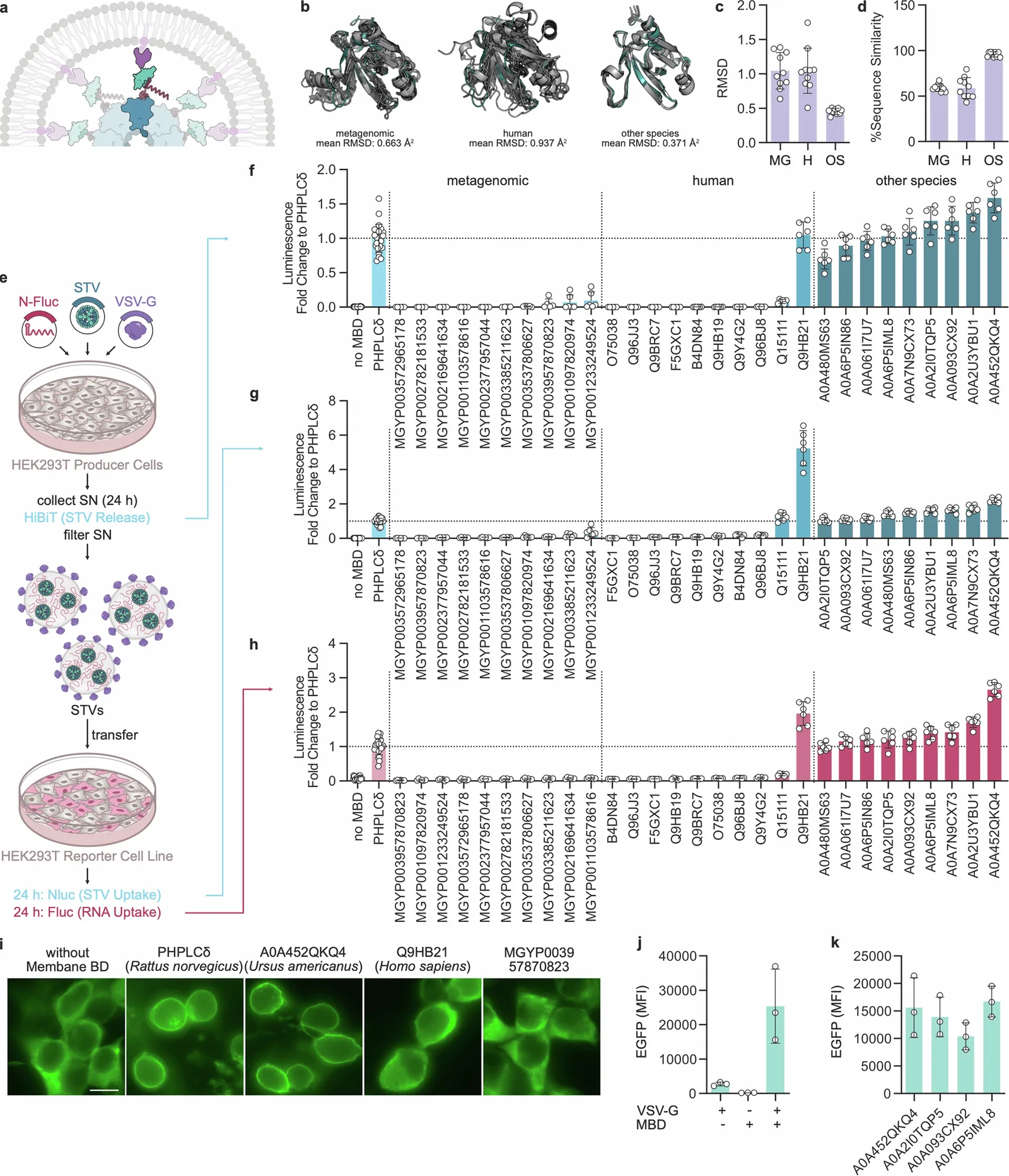
Screening for improved membrane binding domains. **a**, Illustration of STV release by PH domain-dependent binding to the plasma membrane of producer cells. **b**, Structural alignment of pleckstrin homology domains derived from unrestricted (OS) or human (H)/metagenomic (M) restricted FoldSeek search. **c**, Structural similarity of FoldSeek PH domains compared to *Rattus norvegicus* PHPLCδ domain (mean ± s.d. for metagenomic and human n = 10 samples, mean ± s.d. for other species n = 9 samples). **d**, Sequence similarity of FoldSeek PH domains compared to *Rattus norvegicus* PHPLCδ domain (mean ± s.d. for metagenomic and human n = 10 samples, mean ± s.d. for other species n = 9 samples). **e**, Schematic workflow for measuring release, uptake, and RNA delivery efficiency of STVs containing different membrane binding domains. **f**, Quantification of the release of STV variants by measuring HiBiT signal in producer cell supernatant (mean ± s.d. for n = 6 biological replicates). **g**, Quantification of STV uptake in Split-Luc reporter cells by measuring NanoLuc signal from reconstituted LgBiT/HiBiT in cell lysate (mean ± s.d. for n = 6 biological replicates). **h**, Quantification of RNA transfer efficiency by measuring Firefly luciferase signal in transduced Split-Luc reporter cell lysate (mean ± s.d. for n = 6 biological replicates). **i**, Representative image of the subcellular localization of STVs with different membrane binding domains, determined by immunostaining of STVs in transfected producer cells (scale bar: 10 µm). **j**, Flow Cytometry analysis of STV’s MBD requirement by EGFP delivery into HEK293T cells (mean ± s.d. for n = 3 biological replicates). **k**, Flow Cytometry-based validation of screening results by delivering EGFP into HEK293T cells with STVs containing different PH domains (mean ± s.d. for n = 3 biological replicates). Source Data

To further scale the design space of STVs, we combined the identified HE0690 assembly domain with a diverse panel of membrane-binding domains. We selected these membrane-binding domains by performing a structure-based search using FoldSeek^18^, with the PHPLCδ domain from *R. norvegicus* as the template structure. We selected 29 domains from an unrestricted search across all species, a restricted search for human proteins and a search for metagenomic proteins in the ESMAtlas^19^ (Extended Data Fig. 4a–d). We created a library of membrane-binding domains fused to SynL-tdPCP-HE0690 and tested them using the established screening scheme for STV release, STV uptake and RNA transfer efficiency (Extended Data Fig. 4e). This analysis revealed that the membrane-binding domain from *Ursus americanus* (*Ua*PHPLC), a previously uncharacterized PH domain, is a highly efficient domain for RNA packaging and transfer (Extended Data Fig. 4f–h). In addition, we confirmed robust membrane localization of *Ua*PHPLC-STVs in producer cells and validated their ability to efficiently transfer *EGFP* mRNA into target cells (Extended Data Fig. 4i–k). The STV construct, consisting of* Ua*PHPLC and SynL-tdPCP-HE0690, was subsequently named STV-C8 (Fig. 2a).

## Characterization and programming of STV-C8

**Extended Data Fig. 5:**
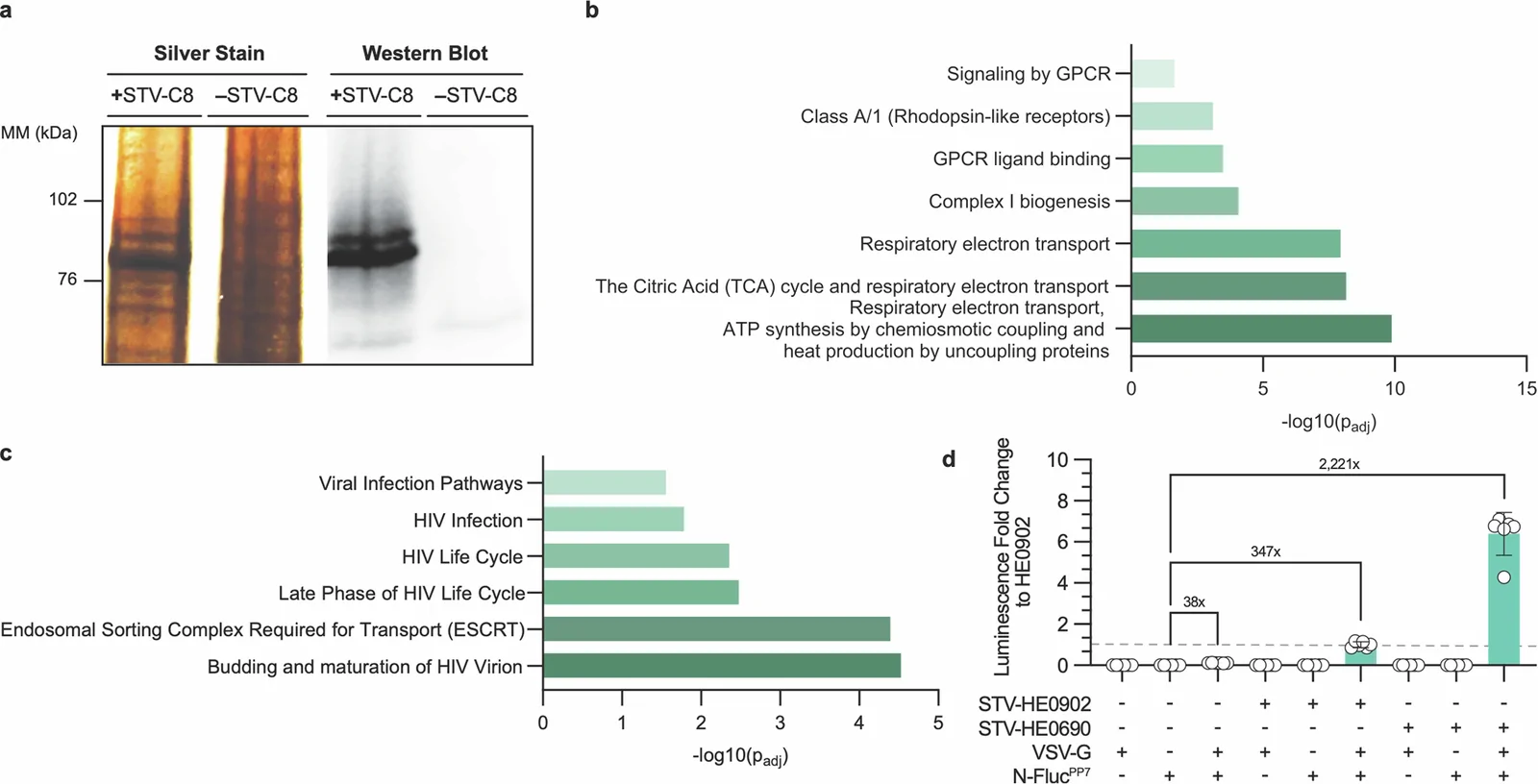
Analysis of purity and particle parameters of STV-C8. **a**, Representative gel images of the characterization of the protein content of purified STV-C8 particles by Silver Stain and Western Blot. **b**, GO term analysis of at least 3-fold excluded mRNAs in STV-C8 particles. **c**, GO term analysis of at least 3-fold enriched proteins in STV-C8 vesicles. GO term enrichment analysis were performed using the cumulative hypergeometric test (one-sided Fisher’s exact test) and P values were corrected for multiple testing using g:SCS multiple testing correction method. **d**, Comparison of N-Split mRNA delivery into Split-Luc reporter cells for vesicles released by VSV-G expression, the original STV design based on HE0902, and the optimized STV-C8 (mean ± s.d. for n = 6 biological replicates). Source Data

**Extended Data Fig. 6:**
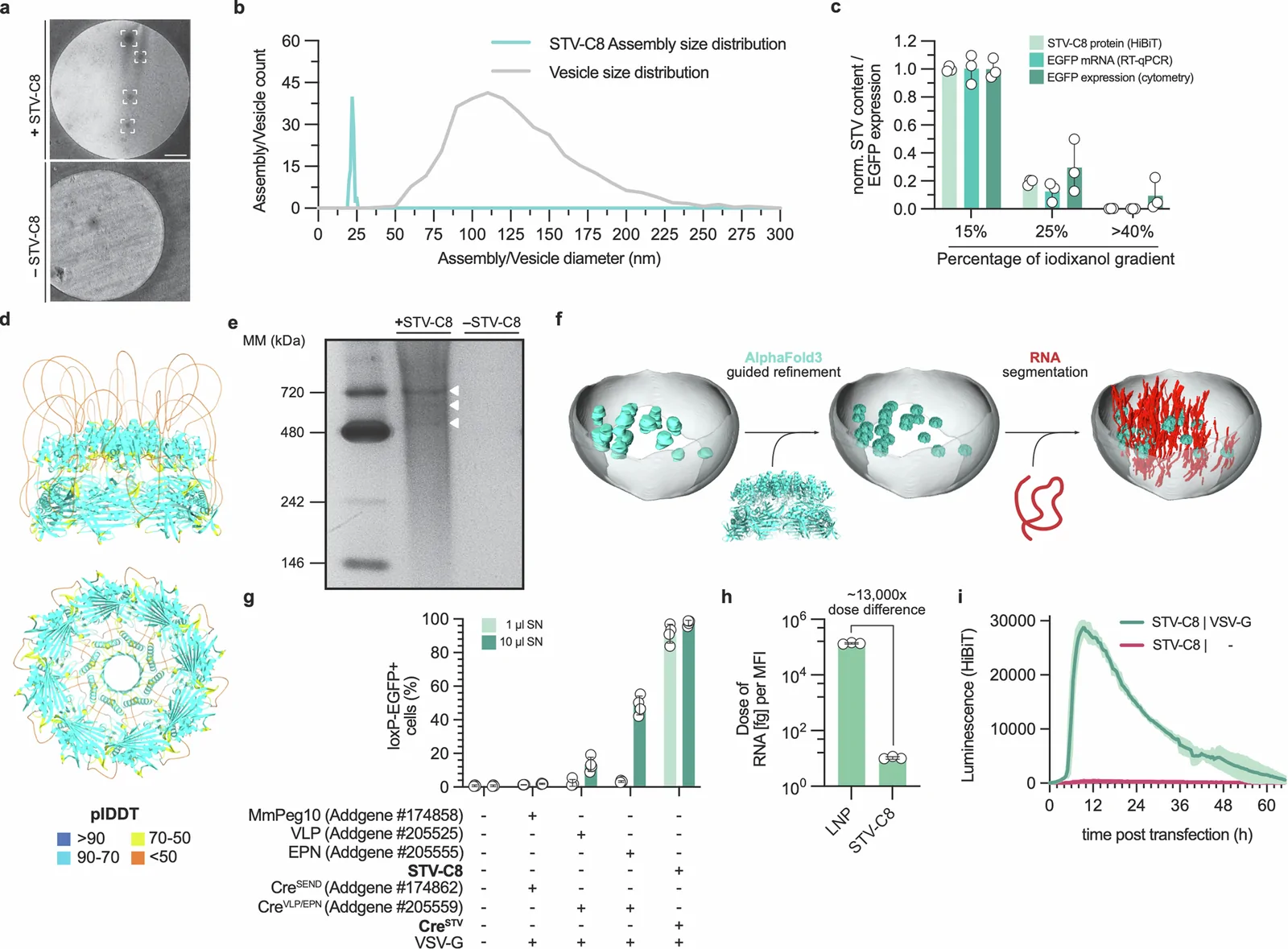
Comprehensive characterization and benchmarking of STV-C8. **a**, Zoomed-in EM grid region (representative example image; scale bar: 500 nm) of concentrated supernatants from STV-C8 transfected or untransfected cells. Representative STV-C8 vesicles are indicated by white squares. **b**, Size distribution of STV-C8 assemblies within vesicles and vesicles on EM grids (500 assemblies and 1,100 vesicles analyzed). **c**, Characterization of different iodixanol concentrations for purifying STV-C8 vesicles by ultracentrifugation, based on STV protein or cargo RNA content and expression in target cells (mean ± s.d. for n = 3 biological replicates). **d**, AlphaFold3 structure prediction of the STV-C8 oligomer, colored based on pIDDT score. **e**, Representative native PAGE of concentrated supernatants from STV-C8–transfected and untransfected cells. High-molecular-mass assemblies are indicated by white triangles. **f**, Segmentation of an STV-C8 vesicle tomogram, showing the STV-C8 oligomer with a docked AlphaFold3-predicted atomic model, as well as the segmentation of the RNA. **g**, Benchmarking of STV-C8 efficiency for delivering Cre mRNA into lox-stop-lox (LSL) EGFP reporter cells for different cell-derived RNA transport vehicles (mean ± s.d. for n = 4 biological replicates). **h**, Comparison of the required dose of EGFP mRNA to induce the expression of one MFI unit (Mean Fluorescence Intensity), quantified by Flow Cytometry in target cells for LNPs or STV-C8 dependent delivery. The STV-C8 mRNA content was quantified by RT-qPCR by comparing it to an in vitro transcribed reference EGFP mRNA (mean ± s.d. for n = 3 biological replicates). **i**, Live measurement of STV-C8 uptake kinetics into target cells by HiBiT-LgBiT reconstitution in Split-Luc target cells. (mean ± s.d. for n = 3 biological replicates). Source Data

Having optimized the domain composition, we characterized the properties of STV-C8 as a bottom-up assembled RNA transfer vehicle. More than 10,000-fold enrichment of the cargo RNA in the supernatant was achieved with STV-C8 vesicles compared with a control missing the membrane-binding domain (Fig. 2b). To further analyse the characteristics of STV-C8, we established a purification method using ultracentrifugation (Extended Data Fig. 5a and Supplementary Fig. 1a) and analysed particle numbers and absolute protein and/or RNA content per particle (Supplementary Fig. 2). Furthermore, we characterized the RNA content of purified STV-C8 particles and found strong enrichment of the *EGFP* cargo RNA (Fig. 2c). Consistent with previous reports^20^, mRNAs were excluded from STV-C8 particles, presumably because they are not accessible for packaging (Extended Data Fig. 5b). In addition to RNA, we characterized the protein content of STV-C8 particles and found strong enrichment of ESCRT-related proteins involved in budding (Fig. 2d and Extended Data Fig. 5c). VSV-G expression in mammalian cells induces release of vesicles^21^. We found that the expression of STV-C8 enhanced the intrinsic budding ability of VSV-G by more than 50-fold (Extended Data Fig. 5d). Next, we imaged the STV-C8 induced vesicles and characterized their size distribution using cryo-electron tomography (cryo-ET) (Fig. 2e). In purified supernatants of STV-C8 transfected cells, we observed vesicles ranging from 50 nm to 300 nm in diameter, with most measuring 110 nm, whereas we could not detect any such vesicles in supernatants from untransfected control cells. Cryo-ET analysis revealed dozens of protein structures within these vesicles, with a typical diameter of 22 ± 1.4 nm (Extended Data Fig. 6a,b); this was consistent with the expected STV-C8 assemblies, although the precise atomic organization of the assembly could not be resolved. On the basis of the observed size distribution of STV-C8 vesicles, we characterized the impact of different purification conditions. We found clear enrichment of STV-C8 vesicles in the 15% iodixanol fraction, as indicated by enriched RNA and protein content and the highest delivery efficiency into target cells (Extended Data Fig. 6c). The size and shape of the vesicles matched the AlphaFold-predicted oligomeric structure of STV-C8 (Extended Data Fig. 6d). In addition, we analysed supernatants from STV-C8-transfected cells by native PAGE and found high-molecular-mass assemblies, confirming the multimeric nature of STV-C8 (Extended Data Fig. 6e and Supplementary Fig. 1b). To further elucidate the organization of the STV-C8 oligomer within released vesicles, we reconstructed vesicle tomograms; these revealed the presence of STV-C8 assemblies. We used an AlphaFold3-predicted model for docking to evaluate the consistency of the dimensions of the assemblies and the segmentation of the packaged RNA (Extended Data Fig. 6f and Supplementary Fig. 3). Although STV-C8 assemblies were generally smaller than viral capsids, they induced release of vesicles of similar size to those released by enveloped viruses^9^. This indicates a biophysical optimum of vesicle sizes and supports the initial hypothesis that non-natural synthetic protein assemblies function similarly to natural capsid protomers by initiating budding through membrane bending. Furthermore, our observations indicate that STV-C8 may package the cargo RNA on the surface of its oligomers, as the tdPCP RNA-binding protein faced outside the HE0690 structure, with the surrounding membrane providing protection for the cargo RNA.

Given the different architecture of STV-C8, we next investigated how efficiently it delivered cargo RNAs into cells compared with previously established delivery systems. We benchmarked the efficiency of *EGFP* mRNA transfer into target cells against that of commonly used genetically encoded systems: virus-like particles (VLPs)^20^, enveloped protein nanocages (EPN24)^15,20^ and selective endogenous encapsidation for cellular delivery (SEND)^22^. For each of these vehicle types, we added the corresponding packaging signal to an *EGFP* mRNA and compared the delivery efficiency in four cell lines from three species. We cloned the coding sequence of each system into a CAG-promoter-driven plasmid backbone; transfected identical amounts of VSV-G, cargo and packaging plasmid into HEK293T cells; collected the supernatant for 3 consecutive days; concentrated the supernatant; and added the processed samples in identical volumes to the target cells. Across all cell lines tested, we found the highest EGFP expression in target cells for STV-C8-delivered *EGFP* mRNA (Fig. 2f). Similarly, we packaged mRNAs encoding Cre recombinase in each system, with plasmids directly obtained from Addgene, and also found superior efficiency of STV-C8 delivery for for Cre mRNA (Extended Data Fig. 6g). To further evaluate STV-C8 as an RNA carrier, we benchmarked it against clinically used LNPs, formulated with the ionizable lipid ALC-0315 that is used in the COVID-19 vaccine BNT162b2^23^. We performed serial dilution of LNP and STV-C8 packaged *EGFP* mRNA with target HEK293T cells and found that the transfection rate was more than 1,000-fold higher with STV-C8 than with LNPs (Fig. 2g). In addition, we analysed the mRNA dose delivered by STV-C8 or LNPs that was required to induce a certain EGFP protein expression level. The mRNA dose requirement for STV-C8 to induce the same expression as LNP-delivered mRNA was more than 10,000-fold lower (Extended Data Fig. 6h and Supplementary Fig. 4). Use of the HibiT tag, which is part of the STV-C8 construct, allowed us to further characterize uptake kinetics. By adding live luciferase substrate Endurazine to STV-C8-treated split luciferase (split-Luc) reporter cells, we monitored the uptake of STV-C8 vesicles into target cells and found that onset occurred after 2–4 h, with the maximum uptake after 10–12 h (Extended Data Fig. 6i).

Unmodified mRNA is a potent trigger for the innate immune response^24^. Therefore, we tested whether STV-C8-delivered RNA induced interferon signalling. Unlike plasmid DNA, which is a known trigger of the innate immune response, treatment with RNA-containing STV-C8 did not cause any detectable interferon response in A549-IFN reporter cells (Supplementary Fig. 5a). We proposed that the biological production process of the cargo RNA might alleviate the interferon response. To test this hypothesis, we transcribed mTagBFP2 mRNA in vitro (capped and polyadenylated) containing either regular uridine or *N*^1^-methylpseudouridine. We packaged these mRNAs into LNPs and produced, in parallel, mTagBFP2 mRNA, packaged in STV-C8. We treated A549-IFN reporter cells with these samples. As expected, unmodified mRNA caused a strong activation of reporter cells, whereas neither modified mRNA nor STV-C8-delivered mRNA induced activation of the reporter cells (Fig. 2h).

**Extended Data Fig. 7:**
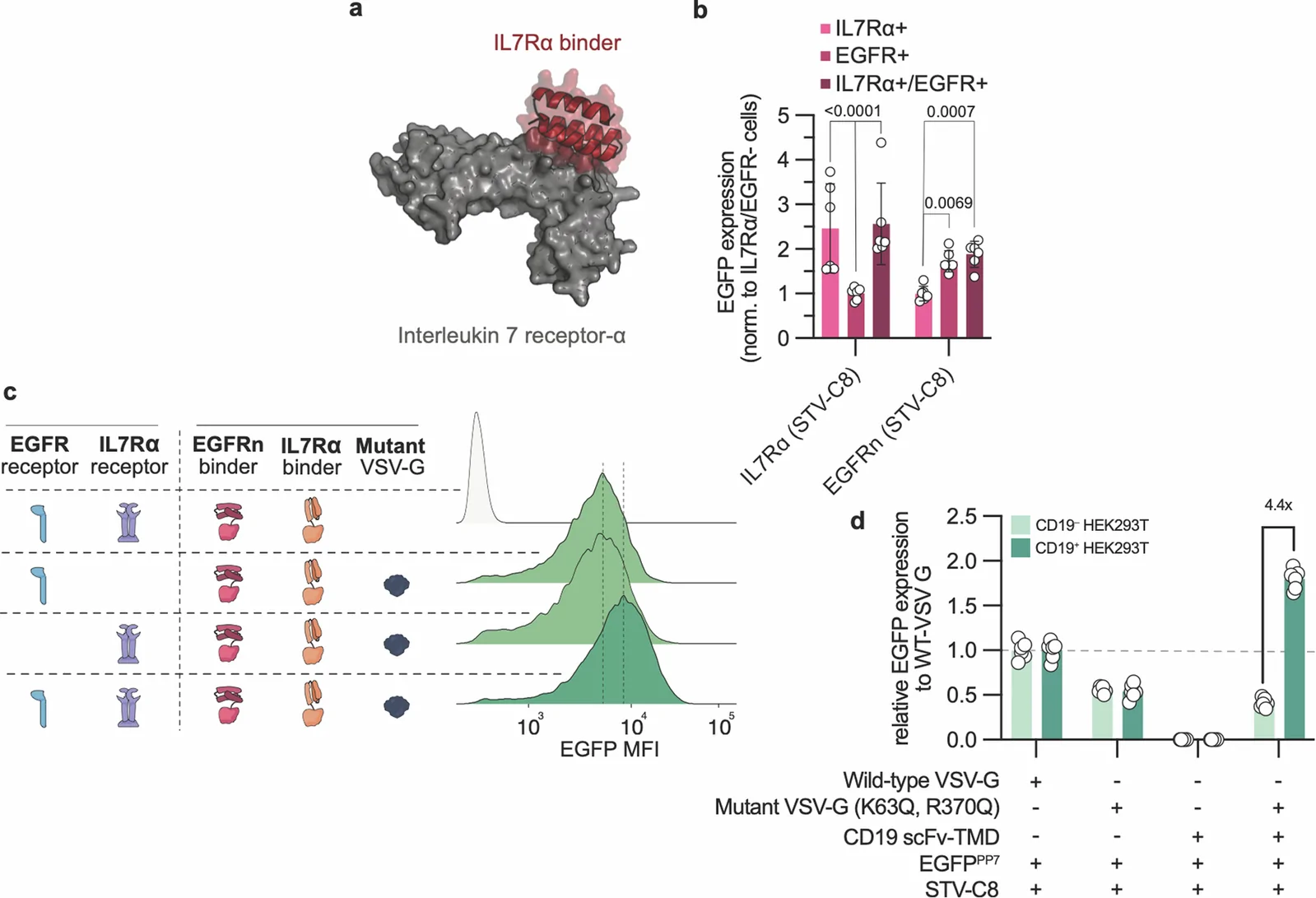
Programming of STV-C8 cell-type specificity with AI-designed minibinders. **a**, AlphaFold2 structural prediction of IL7Rα minibinder bound to its target receptor. **b**, Transduction efficiency of IL7Rα+/EGFR+ or double-positive HEK293T cells with minibinder-equipped STV-C8 particles, containing EGFP mRNA (two-way ANOVA, mean ± s.d. for n = 6 biological replicates). **c**, Flow Cytometry analysis of EGFP expression in IL7Rα+/EGFR+ or double-positive HEK293T cells, transduced with STV-C8(EGFP) that were equipped with both IL7Rα and EGFR minibinders simultaneously. **d**, Delivering EGFP mRNA into CD19-expressing cells by co-expressing a signal peptide/transmembrane domain (TMD) fused CD19 scFv for targeting (mean ± s.d. for n = 6 biological replicates), analyzed by Flow Cytometry. Source Data

A critical aspect of delivery vehicles, in addition to efficiency, is their cell-type specificity. Recently, mutant versions of VSV-G have been created that retain endosomal escape activity but abolish binding to the target receptor LDLR. When combined with antibodies, such blinded VSV-Gs can guide VLPs to preferentially target alternative receptors^25–27^. We considered whether the concept of using artificially designed proteins could be extended to the programming of cell-type specificity, to overcome the limited availability of antibodies. We added computationally designed peptide binders against EGFR or IL-7Rα to STV-C8 particles^28^ by fusing the binders to a signal peptide along with a transmembrane domain and expressed these constructs together with a blinded VSV-G (K63Q/R370Q) in STV-C8(EGFP)-producing cells. After budding of STV-C8(EGFP) from the plasma membrane, these synthetic binding modules were incorporated into the STV-C8 surface and mediated cell-type specificity (Fig. 2i and Extended Data Fig. 7a). We added programmed STV-C8(EGFP) to EGFR or IL-7Rα target cells and measured their uptake into receptor-expressing cells using flow cytometry. We found a strong preference of programmed STV-C8 for the respective target cell line (Fig. 2j) and confirmed that cells expressing both receptors were permissive for STV-C8(EGFP) containing either EGFR or IL-7Rα binders (Extended Data Fig. 7b). We also found that the uptake of STV-C8(EGFP) containing both binders on their surface was further increased in target cells expressing both receptors, indicating a possible additive effect. This finding could be explored further for precise cell-type-specific targeting and could offer enhanced uptake efficiency compared with previously described targeting strategies based on antibody fragments^26^ (Extended Data Fig. 7c,d).

**Extended Data Fig. 8:**
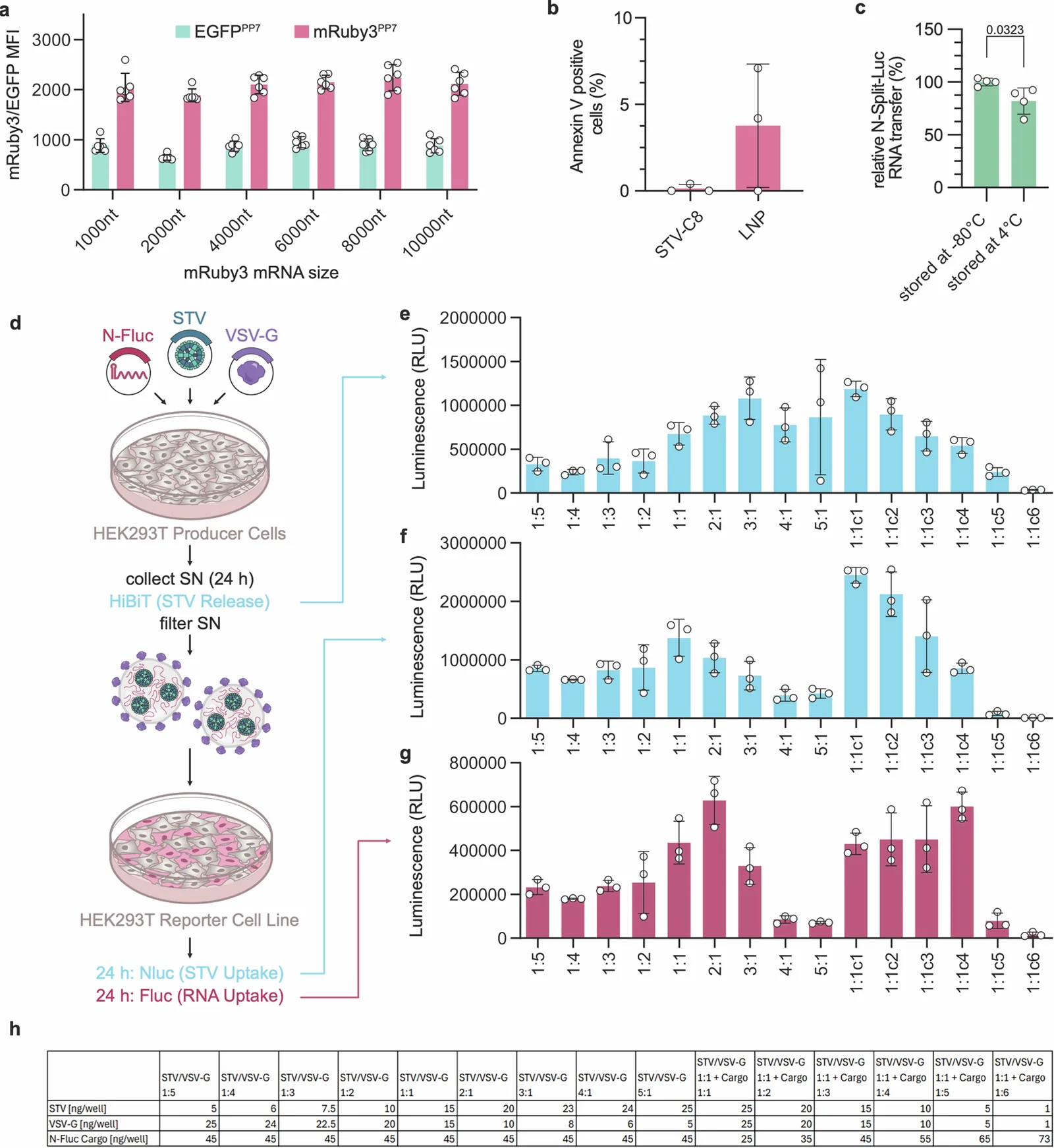
Characterization of STV-C8 packaging capacity, toxicity, and production conditions. **a**, Flow Cytometry-based analysis of cells transduced with STV-C8, co-packaging EGFP mRNA of 1 kb constant length and mRuby3 of varying 3′UTR lengths (mean ± s.d. for n = 6 biological replicates). **b**, Analysis of Annexin V positive cells by Flow Cytometry. Cells were transduced with EGFP mRNA-containing vehicles in sufficient concentration to reach 50–60% EGFP+ cells in each condition (mean ± s.d. for n = 3 biological replicates). **c**, Measurement of N-Split-Luc RNA transfer into Split-Luc reporter cells after 7 d of STV-C8 storage at different temperatures (two-sided unpaired Student’s t-test, mean ± s.d. for n = 4 biological replicates). **d**, Schematic workflow to optimize plasmid amounts for STV-C8 release, uptake, and RNA delivery efficiency. **e-g**, Comparing STV-C8 release (HiBiT assay in supernatant), STV-C8 uptake (NanoLuc assay in target cell lysate), and RNA transfer (Firefly assay in target cell lysate) upon transfection of different amounts of cargo, packaging and fusogen plasmids (mean ± s.d. for n = 3 biological replicates). **h**, Summary of tested plasmid amounts and ratios. Source Data

Viruses such as adeno-associated viruses (AAVs) and lentiviruses are widely used for delivery of genetic material into cells, but they have limited packaging capacity. Therefore, we characterized the cargo size that could efficiently be packaged by STV-C8. We copackaged an *EGFP* mRNA of constant length with mRuby3 mRNAs of varying lengths. Across the mRNA sizes tested (1–10 kb), we detected similar expression levels of EGFP and mRuby3 in target cells, suggesting that STV-C8 did not have a packaging limit within the range of most cargo RNAs (Extended Data Fig. 8a). We proposed the hypothesis that RNA packaging on the surface of HE0690 oligomers instead of inside a capsid shell might relax the size constraints of STV-C8 compared to viruses. In addition, we could not detect signs of STV-C8-induced apoptosis (Extended Data Fig. 8b), we confirmed that STV-C8 are stable for at least 1 week under 4 °C storage conditions, which would facilitate their practical use (Extended Data Fig. 8c), and we characterized the optimal cargo RNA/STV-C8/fusogen ratio (Extended Data Fig. 8d–h).

## Delivery of mRNAs into cellular models

**Fig. 3:**
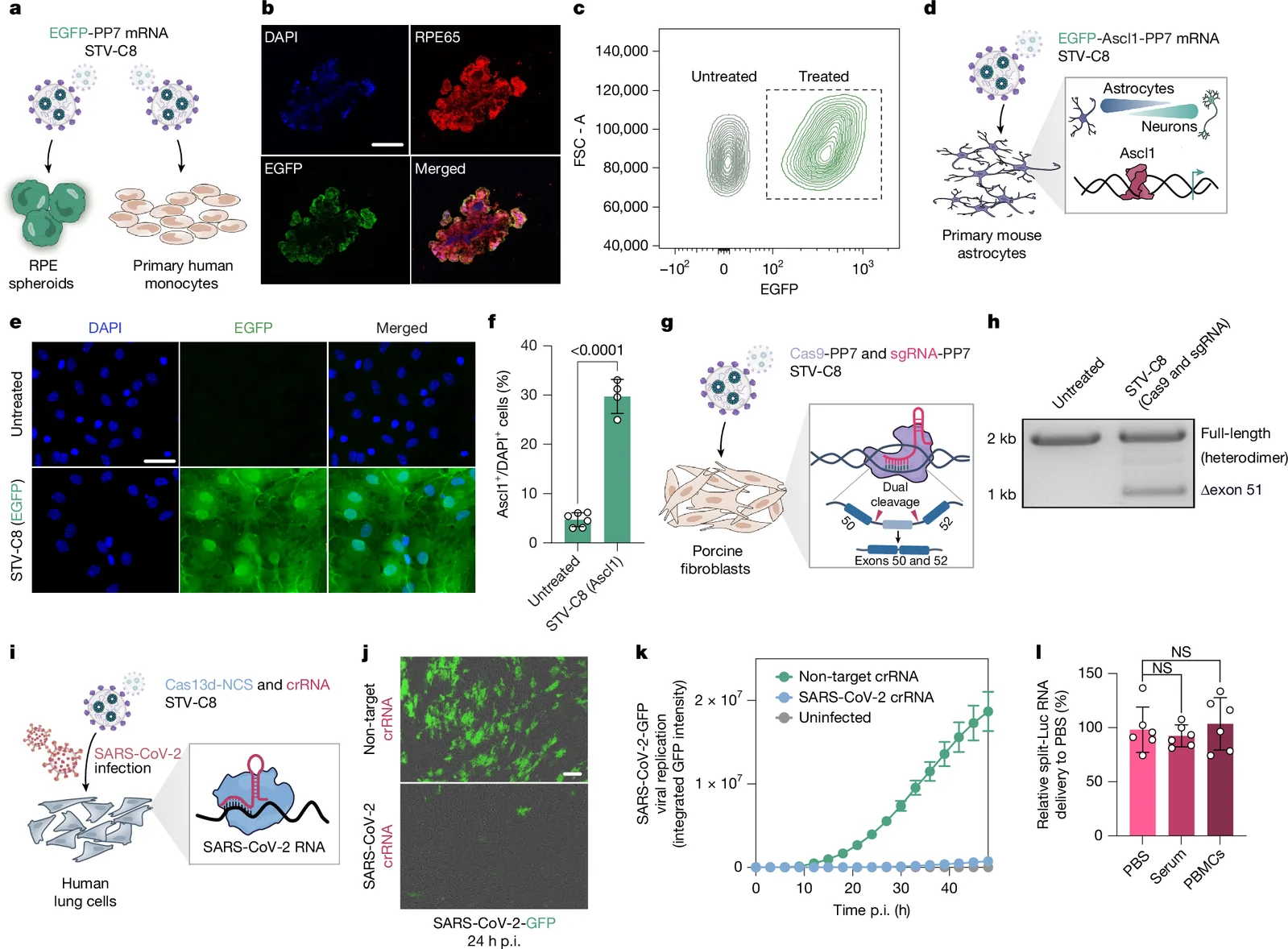
STV-C8 dependent cargo RNA delivery into diverse cellular models. **a**, Packaging of *EGFP* mRNA into STV-C8 and delivery into human monocyte suspension cells and iPS cell-derived RPE spheroids. **b**, Confocal imaging of RPE spheroid cryosections, 2 days after transduction with STV-C8, containing *EGFP* mRNA and costained with RPE65 as a specific marker protein for RPE cells (representative images; scale bar, 100 µm). **c**, Flow cytometry analysis of human monocytes, untransduced or transduced with STV-C8 containing *EGFP* mRNA, 24 h after treatment. **d**, Packaging of polycistronic mRNA encoding the ASCL1 transcription factor with EGFP into STV-C8 and transduction of primary mouse astrocytes. **e**, Fluorescence imaging of primary mouse astrocytes, 3 days after transduction with STV-C8 packaging *EGFP* mRNA (representative images; scale bar, 50 µm). **f**, Quantification of ASCL1-positive astrocytes, transduced with ASCL1 containing STV-C8 and stained for ASCL1 (two-sided unpaired Student’s *t*-test, mean ± s.d. for *n* = 6 control and *n* = 4 treated independent astrocyte cultures). **g**, Schematic illustration of STV-C8-mediated Cas9/sgRNA delivery into porcine fibroblasts, resulting in deletion of dystrophin exon 51 by sgRNAs cleaving in the flanking introns. **h**, Representative PCR amplification of the dystrophin gene from Cas9/sgRNA STV-C8-treated porcine fibroblasts 3 days after treatment. **i**, STV-C8-mediated delivery of SARS-CoV-2-targeting Cas13d-NCS into virus-infected human lung cells. **j**, Fluorescence imaging of iPS cell-derived human lung cells, infected with SARS-CoV-2-GFP (multiplicity of infection: 10) and treated with STV-C8(Cas13d-NCS/crRNA), 24 h post-infection (p.i.) (representative images; scale bar, 100 µm). **k**, Live imaging of SARS-CoV-2-GFP replication in STV-C8(Cas13d-NCS/crRNA)-treated iPS cell-derived human lung cells for 48 h (mean ± s.d. for *n* = 3 biological replicates). **l**, Measurement of N-split-Luc mRNA transfer into split-Luc reporter cells after pretreatment of STV-C8 delivery vehicles with human whole-blood and serum samples (two-sided unpaired Student’s *t*-test, mean ± s.d. for *n* = 6 biological replicates). Source Data

**Fig. 4:**
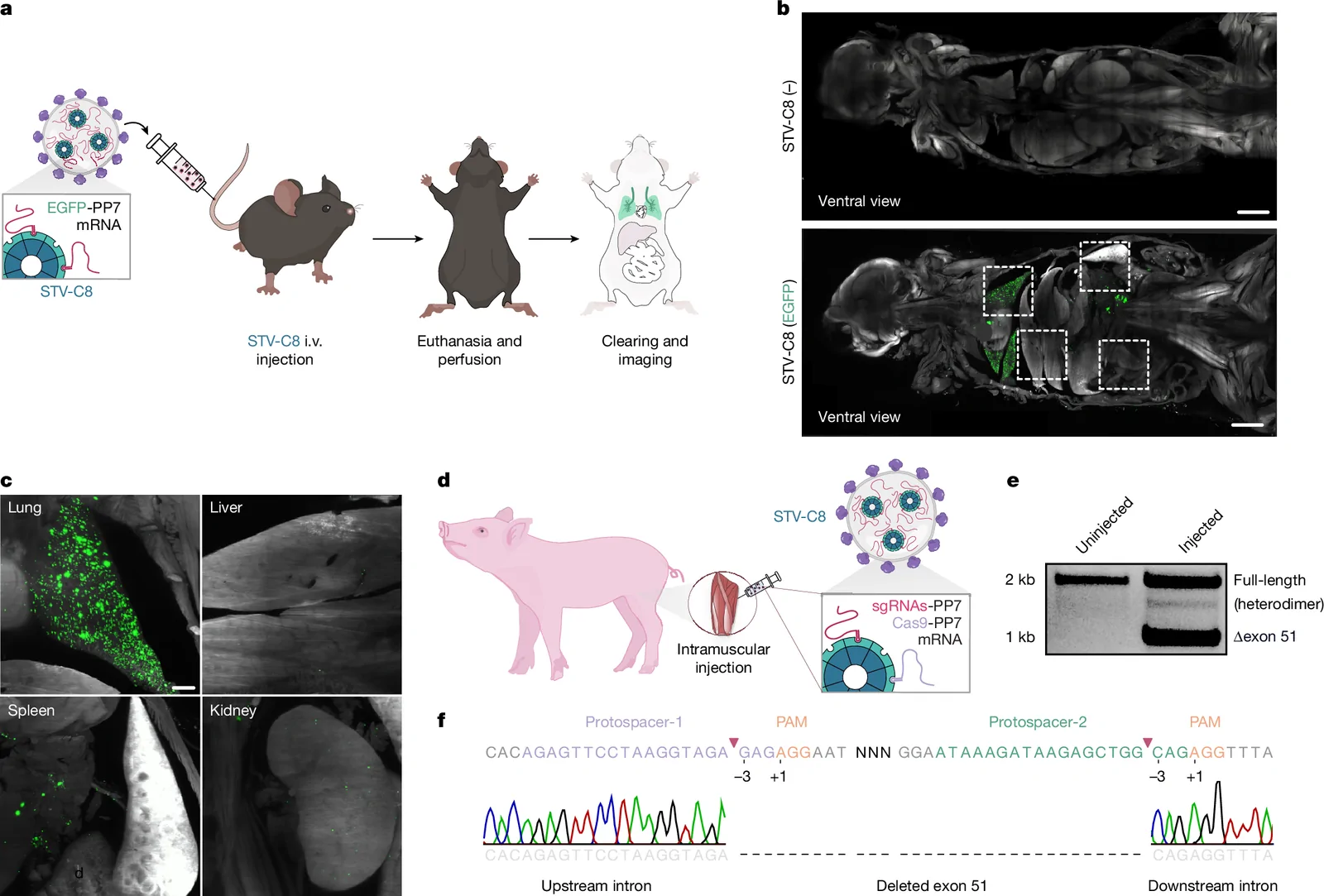
STV-C8 biodistribution and delivery of gene-editing cargos in mouse and pig models. **a**, Schematic illustration of in vivo biodistribution analysis of STV-C8-mediated EGFP expression by mouse whole-body clearing and imaging. i.v., intravenous. **b**, Ventral view of amplified EGFP expression in cleared mouse body, treated with *EGFP* mRNA or empty STV-C8 vesicles, imaged by light-sheet microscopy 72 h after intravenous injection (representative images; EGFP in green, with tissue autofluorescence in greyscale; scale bars, 5 mm). Dashed rectangles outline regions magnified in **c**. **c**, Imaging of EGFP signal in cleared lung, liver, spleen and kidney tissues (representative images; scale bar, 500 µm; kidney tissue was imaged from a different plane). **d**, Schematic illustration of Cas9/sgRNA delivery into pig muscle by local injection of STV-C8 vehicles. **e**, Representative PCR analysis of the edited dystrophin gene 72 h after intramuscular injection. After treatment with STV-C8(Cas9/sgRNAs), exon 51 was deleted from the gene. **f**, Sanger sequencing of the PCR band, corresponding to the deleted exon 51 in treated pig muscle cells.

Having shown that STV-C8 could function as a transport vehicle to enable highly efficient transfer of reporter RNAs into various cell lines, we delivered a panel of relevant cargo RNAs into complex cellular models. Recently, sequences of novel CRISPR proteins were created in silico by a protein language model. We demonstrated that one of these proteins, OpenCRISPR-1 (ref. ^29^), could be efficiently delivered with STV-C8 and induce similar editing rates to wild-type Cas9 in traffic light reporter (TLR) cells^30^ (Supplementary Fig. 5b). Next, we tested the ability of STV-C8 to deliver the *EGFP* cargo RNA into a multilayer cellular model. Retinal pigment epithelial (RPE) spheroids dissected from induced pluripotent stem cell (iPS cell)-derived human retinal organoids were transduced with STV-C8(EGFP), and we also observed EGFP expression in inner spheroid layers, demonstrating that STV-C8 can mediate delivery beyond the outer cell layers (Fig. 3a,b and Supplementary Fig. 6a,b). We also found high delivery rates in primary human monocytes as a model for suspension cells (Fig. 4c). Next, we isolated primary cortical astrocytes from mouse brains and also confirmed a high transduction rate of STV-C8(EGFP) in this model (Fig. 3d,e). In addition, we delivered mRNA encoding proneuronal transcription factor *Ascl1* into cortical astrocytes and induced ASCL1 expression in more than 30% of cells (Fig. 3f). Efficient transfer of gene editors is among the most relevant applications of novel delivery vehicles. We added the PP7 packaging signal to the 3′ untranslated region (UTR) of the Cas9 mRNA and cloned two single guide RNAs (sgRNAs) containing the PP7 signal in the tetraloop of the sgRNA, along with spacers targeting the intronic region upstream and downstream of exon 51 of the dystrophin gene. Deletion of exon 51 of the dystrophin gene has been shown to be a viable strategy for rescuing a high proportion of DMD-related phenotypes^13,31^. We treated primary porcine fibroblasts with STV-C8(Cas9/sgRNA) particles (Fig. 3g). Amplification of the dystrophin locus from treated cells revealed a deletion frequency of approximately 40% (Fig. 3h and Supplementary Fig. 7a). To further demonstrate the flexibility of STV-C8, we packaged and delivered the programmable antiviral Cas13d-NCS^32^. We added the PP7 signal to the 3′ UTR of Cas13d-NCS mRNA and to the 3′ part of a CRISPR RNA (crRNA) targeting the SARS-CoV-2 genome. We produced STV-C8(Cas13d-NCS/crRNA) particles, delivered them into iPS cell-derived human lung cells and infected these cells with SARS-CoV-2-GFP (Fig. 3i and Supplementary Fig. 6c). The viral replication was monitored in a live imaging setup; this showed that STV-C8-delivered Cas13d-NCS almost entirely blocked the virus (Fig. 3j,k). To further validate the therapeutic potential of STV-C8 particles, we tested their stability in human blood samples and found that they were unaffected by the blood treatment (Fig. 3l).

## Delivery of mRNAs into animal models

**Extended Data Fig. 9:**
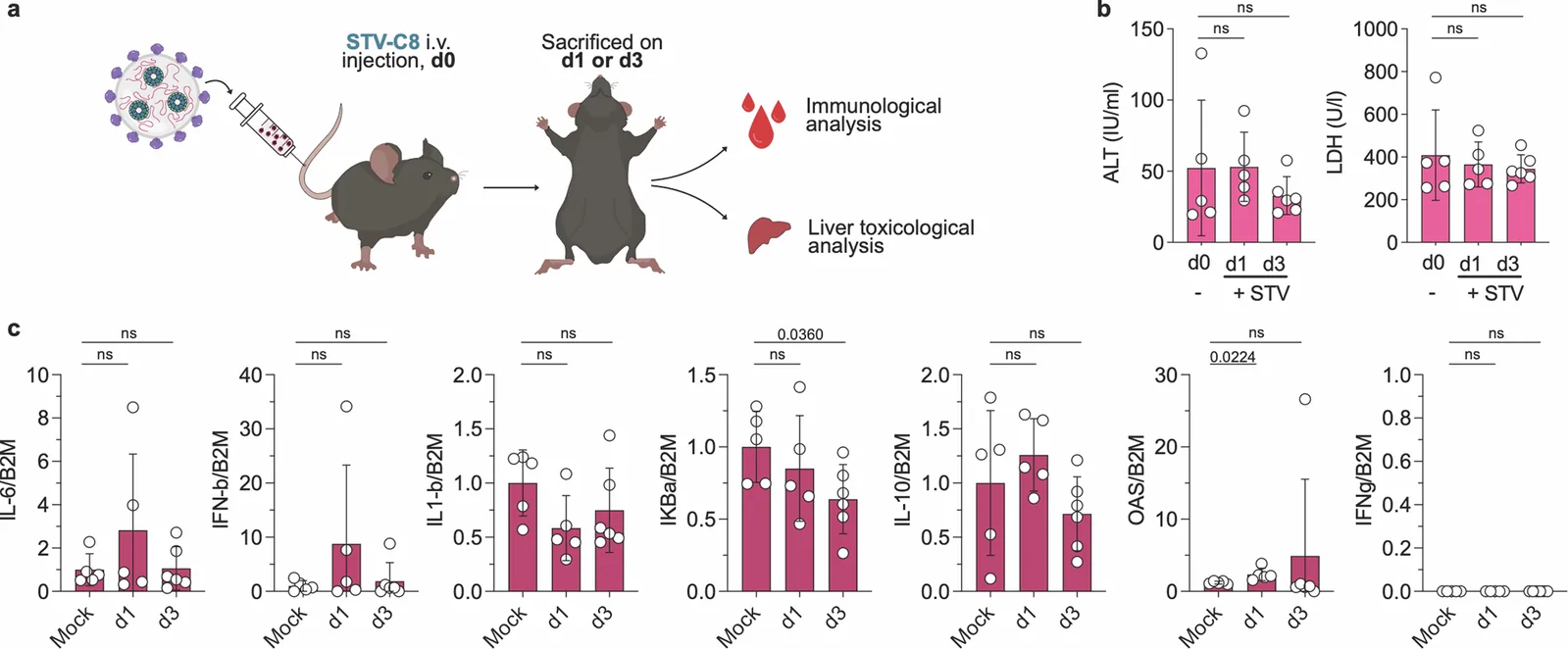
Evaluation of STV-C8-mediated immunological response and liver damage in vivo. **a**, Scheme of the experimental setup for testing immunological markers in blood and liver toxicity. **b**, Analysis of potential liver toxicity (ALT, alanine aminotransferase) and broader toxicological side effects (LDH, lactate dehydrogenase) of animals, treated by systemic STV-C8 injection (two-sided, Mann-Whitney, mean ± s.d. for Mock and d1 n = 5 animals, for d3 n = 6 animals). **c**, Testing for immunological markers by RT-qPCR (two-sided unpaired t-test, mean ± s.d. for Mock and d1 n = 5 animals, for d3 n = 6 animals) animals). Source Data

Having demonstrated that STV-C8 could effectively deliver a wide variety of cargo RNAs into diverse cellular models, we next tested the system in animal models; to our knowledge, this is among the first examples of an artificial-intelligence-designed protein to be tested in vivo. We injected mice intravenously with STV-C8(EGFP) and used an advanced whole-body clearing and imaging technique to comprehensively analyse the biodistribution of STV-C8-mediated EGFP expression at near-single-cell resolution^33,34^ (Fig. 4a). We detected strong and highly specific expression in the lung but no expression in the liver, the common target tissue for most lipid-based vehicles (Fig. 4b,c and Supplementary Fig. 8). The high resolution of the imaging method enabled us to detect a punctate expression pattern in the lung, suggesting specificity within the tissue. In addition, we did not detect any immunological or toxicological side effects of systemic STV-C8 injection (Extended Data Fig. 9).

**Extended Data Fig. 10:**
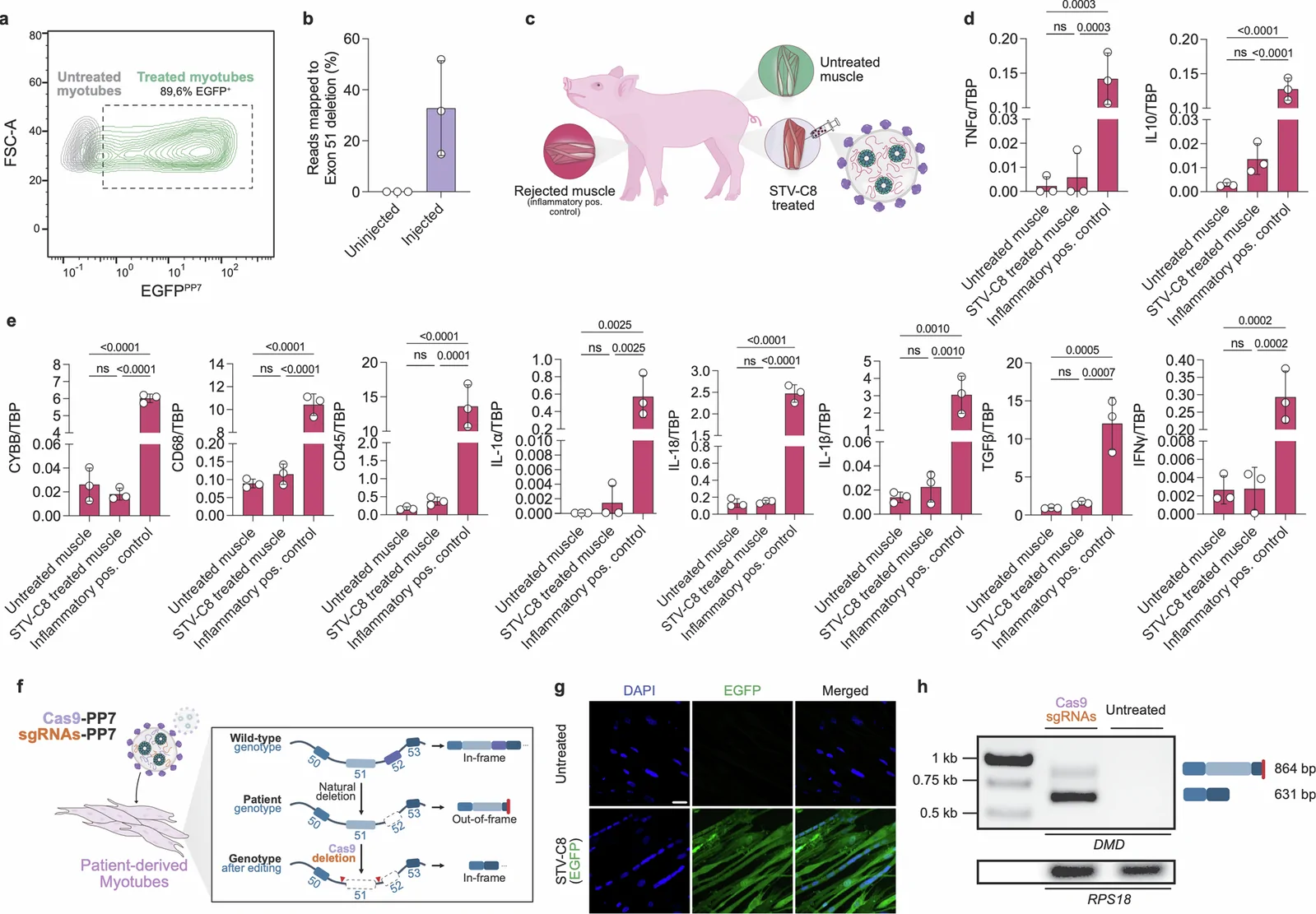
Continued analysis of intramuscular delivery of mRNAs with STV-C8. **a**, Quantification of the EGFP mRNA delivery into C2C12 mouse myotubes by Flow Cytometry. **b**, Long-read nanopore sequencing estimation of deletion efficiency in pig muscle (mean ± s.d. for n = 3 technical replicates of the same injection site). **c**, Schematic description of STV-C8-mediated intramuscular delivery of mRNAs and subsequent immunological analysis. **d** and **e**, Analysis of inflammatory markers of muscle samples by RT-qPCR (one-way ANOVA, mean ± s.d. for n = 3 replicates of different injection sites). **f**, Schematic overview of the experimental setup and genotypes of edited DMD patient-derived myotubes. **g**, Imaging of STV-C8(EGFP) treated myotubes (representative image; scale bar: 25 µm). **h**, Representative gel picture of the size analysis of amplified *DMD* exon 49–54 cDNA obtained from treated and untreat*e*d DMD patient-derived myotubes. Source Data

Exon-skipping strategies are being explored with respect to treatment of DMD^35,36^. We recently reported that CRISPR–Cas9-mediated deletion of exon 51 from the dystrophin gene induced phenotypic rescue of muscular and cardiac function in a DMD pig model^13^. As the AAVs used in that study have been reported to occasionally cause severe side effects^37^, we evaluated STV-C8 as a delivery modality for genetic medicines, such as for DMD. We first confirmed that STV-C8 could efficiently deliver cargo RNA into muscle cells (Extended Data Fig. 10a). Next, we packaged the CRISPR–Cas9 system to delete exon 51 from the dystrophin gene into STV-C8 particles and injected them into the muscle of a pig (Fig. 4d). We amplified the dystrophin locus and confirmed successful deletion of exon 51 in STV-C8(Cas9/sgRNA)-treated muscle (Fig. 4e,f, Extended Data Fig. 10b and Supplementary Fig. 7b). As for the systemic injection, we did not detect any immunological response following local application of STV-C8 in the treated muscle (Extended Data Fig. 10c–e). To further demonstrate that this strategy could be a viable therapeutic approach for DMD, we used skeletal muscle cells (Δ52) derived from patients with DMD and treated the cells with STV-C8(Cas9/sgRNA). We confirmed successful deletion of exon 51 (Extended Data Fig. 10f–h and Supplementary Figs. 7c and 9a) and restoration of the *DMD* open-reading frame (Supplementary Fig. 9b). We acknowledge that intramuscular delivery is not directly applicable to DMD, and that future studies will need to address comparative performance, targeting and cell-type specificity; however, the successful deletion of exon 51 in a large animal model and in skeletal muscle cells derived from patients with DMD, combined with a beneficial safety profile, indicates that STV-C8 has strong clinical potential.

## Discussion

Here we demonstrated that protein assemblies designed by RFdiffusion^3^ can be harnessed to build synthetic RNA transfer vehicles from scratch. By establishing a multidimensional screening platform, we tested more than 100 STV constructs with diverse structures. Using STV-C8, we developed a vehicle architecture that outperformed the RNA transfer efficiency of biological and chemical vehicles and applied it to deliver a wide variety of cargo RNAs, including transcription factors, gene editors and programmable antivirals, into various human-, mouse- and pig-derived cellular and animal models.

Viruses are highly diverse, yet most have converged towards packaging their genomes in large, multimeric protein shells with icosahedral and helical symmetry^1,2,9,10^. Instead of mimicking such natural capsid characteristics^15,20^, we leveraged artificial-intelligence-based protein design to create atypical but functional RNA transport vehicles that outperformed their natural counterparts. The high transport efficiency of STV-C8, combined with its unique size and shape characteristics, suggests that overcoming evolutionary constraints can be beneficial when building RNA transport vehicles from scratch.

The vehicles created here represent an ideal use case for current protein design methods, which are very effective in creating static, symmetric structures but limited in their ability to generate dynamic proteins or enzymatic activities^3,5,38^. Future advances, such as incorporation of molecular dynamics data or development of multimodal models that integrate structure, sequence and functional annotations, could extend the abilities of these models with respect to challenging biological problems^4,39^. Application of tools that enable true de novo design of proteins could overcome the limitations of natural protein diversity, unlocking new directions in various areas of biological and biomedical research.

## Methods

### Molecular cloning

All cloning was performed using standard molecular techniques. Fragments for cloning were generated by PCR using Platinum SuperFi II Master Mix (Thermo Fisher Scientific) and appropriate oligonucleotides (IDT DNA) by plasmid digest using standard restriction enzymes (NEB) or synthesized as gene fragments (Twist Bioscience or IDT DNA). Fragment assemblies were constructed using NEBuilder HiFi DNA Assembly Mix (NEB) or Instant Sticky-end Ligase Master Mix (NEB). Assembled fragments were transformed into self-made chemically competent *Escherichia coli* DH5α cells. Correct clones were identified by plasmid preparation (Monarch Plasmid Miniprep Kit, NEB) and Sanger sequencing (Azenta) or rolling circle amplification directly on cells (Microsynth). Subsequently, plasmids were isolated using a Plasmid Maxiprep Kit (QIAGEN) and used for transfection. All sequences cloned in this study are listed in Supplementary Table 1.

### Plasmid transfection

One day before transfection, cells were seeded at 3.0 × 10^4^ cells per well for 96-well plates, 2.2 × 10^5^ for 24-well plates, 7.5 × 10^5^ for 6-well plates and 4.0 × 10^6^ for 10-cm dishes. Cells were transfected using JetOptimus DNA transfection reagent (Polyplus transfection) with 75 ng DNA per well for 96-well plates, 300 ng DNA per well for 24-well plates, 1 µg DNA per well for 6-well plates, and 5 μg DNA for 10-cm dishes.

### Cell culture and cell lines

HEK293T cells (a gift from the Institute of Developmental Genetics, Helmholtz Munich) were cultivated at 37 °C, 5% CO_2_ in an H_2_O-saturated atmosphere, and maintained in Dulbecco’s modified Eagle medium (DMEM; Gibco) supplemented with 10% fetal bovine serum (FBS; Gibco) and 1% penicillin–streptomycin (Gibco). The HEK293T split-Luc reporter cell line was generated by Cas9 cleavage at the *AAVS1* locus and homology-directed integration of a donor construct containing LgBiT, the carboxy-terminal fragment of Fluc, separated by a P2A sequence, and the puromycin resistance gene. Three days after transfection, the cells were selected for 2 weeks with 2 µg ml^−1^ puromycin (Thermo Fisher Scientific). HEK293T cells stably expressing EGFR or IL-7Rα were generated by amplification of the EGFR sequence from Addgene plasmid 23935 (a gift from W. Hahn and D. Root), whereas IL-7Rα was synthesized (Twist Bioscience). Both coding sequences were cloned into the AAVS1 knock-in donor plasmid, transfected with AAVS1 targeting Cas9 and selected with 2 µg ml^−1^ puromycin. Dual-positive EGFR and IL-7Rα receptor cells were generated by cloning of IL-7Rα into an AAVS1 donor plasmid containing a blasticidin resistance gene, and cells were transfected and selected in 10 µg ml^−1^ blasticidin medium (Thermo Fisher Scientific).

### Quantification of STV-mediated target RNA release into the cell culture supernatant

Supernatants from STV-releasing cells were collected and filtered through 0.45-µm polyvinylidene fluoride (PVDF) filters (Merck Millipore) after 48 h. RNA was extracted with a Monarch Total RNA Miniprep Kit (NEB), and isolated RNA was used as a template for quantitative PCR with reverse transcription (RT–qPCR) with a Luna Universal One-Step RT-qPCR Kit (NEB) and a primer/FAM-probe set (custom design, Metabion) specific for *EGFP* mRNA. The reaction was analysed on a QuantStudio 7 Flex device (Thermo Fisher Scientific).

### Integration of diffusion-designed symmetric oligomers into STV design

Previously designed RFdiffusion symmetric oligomers were filtered for successfully assembled oligomers on the basis of size exclusion data^3^. In addition, all D2 symmetric oligomers were excluded. The resulting 39 sequences were synthesized (eBlocks, IDT DNA) and cloned as a C-terminal fusion to the extra STV components (PHPLC, SynL and tdPCP).

### Integrating structure-mined membrane-binding domains into STV design

The pleckstrin homology domain (PDB: 1MAI) was used as the input structure for a structure-based homology search with FoldSeek^18,40^. Ten sequences from three categories (other species, human, metagenome) were selected on the basis of their having the highest homology to the input structure. Each sequence was synthesized (IDT DNA) and fused to the amino terminus of the previously identified ideal STV construct containing SynL, tdPCP and HE0690.

### Sequence and structural alignments of the structure-mined membrane-binding domains

The amino acid sequences of the structure-mined membrane-binding domains were aligned to that of *R. norvegicus* PHPLCδ, using the residues visible in the X-ray structure (PDB: 1MAI). The alignment was performed with the MAFFT v.7 add tool^41^, using default settings (strategy: auto, scoring matrix: BLOSUM62, gap opening penalty = 1.53, offset value = 0.0). For structural alignment, the structure of the membrane-binding domain region was extracted from the AlphaFold2 (ref. ^42^) (human and other species membrane-binding domains) or ESMFold^19^ (metagenomic membrane-binding domains) predictions of the respective structure-mined proteins containing these membrane-binding domains. These structures were aligned, and the root mean square deviation compared with the X-ray structure (PDB: 1MAI) was calculated using the PyMOL super alignment tool.

### Screening of symmetric oligomer and membrane-binding proteins for RNA release and uptake

Cells were seeded in 96-well format and transfected with each of the oligomer or membrane STV constructs, as well as plasmids encoding VSV-G and N-split-Luc-PP7 (in a 2:1:7 ratio). Twenty-four hours post-transfection, 5 µl of supernatant was collected from the transfected cells, mixed with 45 µl phosphate-buffered saline (PBS), and measured using a Nano-Glo HiBiT Lytic Detection System (Promega) with a Centro LB960 device (Berthold Laboratories), with 0.5 s integration time. Forty-eight hours post-transfection, 120 µl of supernatant was collected and filtered through a 0.45-µm PVDF 96-well filter plate (Sigma-Aldrich) by centrifugation (1,500*g*, 4 °C, 20 min). Cleared supernatant was added to a seeded 96-well plate of C-split-Luc reporter cells. Twenty-four hours later, a Nano-Glo Dual-Luciferase Reporter Assay (Promega) was performed on the cells after complete removal of the supernatant. STV uptake was quantified on the basis of light emission from the NanoLuc substrate. N-split-Luc-PP7 mRNA uptake and expression were measured on the basis of the light emission from the Fluc substrate. In addition, total STV protein transfer and Fluc protein expression in target cells were quantified by comparison of luminescent signals obtained from the STV screen with a Fluc reference sample (Abcam) or HiBiT control protein (Promega).

### Validation of STV-mediated transfer of *EGFP* mRNA by flow cytometry

HEK293T producer cells were transfected in 24-well format with plasmids encoding STV constructs, VSV-G and EGFP-PP7 (2:1:7 ratio). STV-containing supernatant was collected for 2 consecutive days, filtered through a 0.45-μm PVDF membrane filter, and concentrated 5- to 10-fold with Lenti-X Concentrator (Takara Bio) in fresh DMEM. Then, 10–20 µl of resuspended STVs were added to a 96-well plate of HEK293T cells. After 24 h, the treated cells were detached using StemPro Accutase (Thermo Fisher Scientific), mixed with FACS buffer (EDTA/bovine serum albumin (BSA)), and filtered through cell-strainer-containing tubes. Subsequently, samples were gated for living single cells, and *EGFP* mRNA uptake and expression were analysed by flow cytometry (BD FACSaria III, BD Biosciences). Data were analysed using BD FACSDiva (v.6.1.3, BD Biosciences) and FlowJo (v.10, BD Biosciences).

### Design of extra oligomers with C8 symmetry

Extra oligomers featuring C8 symmetry were generated using the open-source version of RFdiffusion, along with the script provided for symmetric oligomers^3^. These computations were performed on a single A100 GPU.

### Determination of subcellular STV localization

HEK293T cells were transfected with STV constructs containing different membrane-binding domains. Twenty-four hours later, cells were fixed with 10% formalin (Sigma-Aldrich) and permeabilized in 1% BSA/0.5% Triton X-100 (diluted in PBS). Permeabilized cells were incubated with primary anti-HA antibody (Sigma-Aldrich, H3663) overnight at 4 °C. Subsequently, the cells were washed and stained with an Alexa 488-coupled secondary donkey anti-mouse antibody (Thermo Fisher Scientific, A21202) overnight at 4 °C. Stained cells were mounted with ProLong Diamond reagent (Thermo Fisher Scientific) and imaged using an Axio Imager M2 fluorescence microscope (Carl Zeiss).

### Characterization of packaging capacity by flow cytometry

EGFP-PP7-STVs were produced in 24-well format as previously described. In addition, producer cells were transfected with mRuby3-PP7 constructs containing random UTR sequences of variable lengths. Concentrated STVs were added to HEK293T target cells. After 24 h, EGFP and mRuby3 expression levels were quantified using flow cytometry as described previously.

### Concentration of STVs by ultracentrifugation for analytical and experimental purposes

Producer cells were seeded in 10-cm dishes coated with poly-l-lysine (Sigma-Aldrich) and transfected with plasmids encoding STV-C8 components required for the respective experiments. Unless otherwise specified, supernatants were collected for 3 consecutive days and stored until day 3 at 4 °C. The collected supernatant was centrifuged for 5 min at 1,000*g* and passed through a 0.45-μm PVDF membrane filter. Filtered supernatant was added to a cushion of 20% (w/v) sucrose (Sigma-Aldrich) in PBS. Subsequent ultracentrifugation was performed at 26,000 rpm for 2 h and 4 °C using a SW28 rotor in an Optima L-60 ultracentrifuge (Beckman Coulter). After centrifugation, the supernatant and the sucrose solution were removed, and the pellet was resuspended in 50 µl ice-cold 1× PBS (Thermo Fisher Scientific) on an orbital shaker at 150 rpm for 45 min at 4 °C. The resuspended pellet was centrifuged at 1,000*g* for 5 min at 4 °C for removal of debris and stored at −80 °C. Following this process, samples were concentrated approximately 300-fold.

### Determination of STV purity for downstream analysis

STV-C8 samples were concentrated by ultracentrifugation, and sample purity was determined by silver staining. Samples were prepared in 2× Laemmli buffer (Sigma-Aldrich) for 10 min at 98 °C. SDS–PAGE was run on a TGX gel with a 4–15% gradient (Bio-Rad) using 1× Tris/glycine/SDS running buffer (Bio-Rad) for 60 min at 130 V. Subsequently, the gel was silver-stained according to the manufacturer’s instructions (Serva). A gel was run in parallel with the same samples and blotted on to a nitrocellulose membrane for 60 min, at 100 V and 4 °C, in transfer buffer (Tris/glycine buffer, Bio-Rad). The position of the STV-C8 protein on the membrane was determined by imaging with a Nano-Glo HiBiT Blotting system (Promega) in a Fusion SL Vilber machine (Peqlab). The HiBiT signal on the membrane was used as a reference to identify STV proteins on the corresponding silver-stained gel.

### Sample and grid preparation for cryo-ET

Seeded producer cells were transfected with plasmids encoding STV-C8 and EGFP-PP7 in 10-cm dishes coated with poly-l-lysine (Sigma-Aldrich). Twenty-four hours after transfection, the cells were washed with PBS, and serum-free DMEM was added. After a further 24 h, the supernatant was collected and concentrated by ultracentrifugation, as previously described. The purified STV-C8 vesicles were diluted to 10^9^ particles per microlitre in PBS. Samples were applied to copper EM grids with Quantifoil R 3.5/1 holey carbon films (200 mesh, Quantifoil) and covered with a homemade 3-nm-thick continuous carbon film produced by flotation. The grids were then glow-discharged at 4 mA for 10 s, blotted and plunge-frozen into liquid ethane using a Vitrobot IV (Thermo Fisher) with the chamber set to 95% humidity at 10 °C. A total of 16 grids were prepared in 2 experiments.

To further characterize the size distribution of STV-C8 vesicles, we prepared a gradient of iodixanol (15%, 25%, 40% and 60%) in PBS-MgCl_2_/KCl/NaCl buffer. Supernatant containing STV-C8 vesicles was produced as described previously with FBS, applied to the iodixanol gradient and concentrated by ultracentrifugation (26,000*g* at 4 °C for 4.30 h). Subsequently, the fractions were collected by puncturing the tube wall with a 27-G needle. The collected samples were applied to 200 mesh copper EM grids with Quantifoil R 3.5/1 holey carbon films and covered with a homemade 3-nm-thick continuous carbon film produced by flotation. The grids were glow-discharged at 4 mA for 10 s, blotted and plunge-frozen into a liquid ethane/propane mix using a Vitrobot IV (Thermo Fisher) with the chamber set to 95% humidity at 4 °C. A total of 18 grids were prepared in 1 experiment, with 2 grids for each triplicate of the 3 conditions (15%, 25% and 40–60% iodixanol).

### Cryo-ET data acquisition, reconstruction, and quantification of vesicle and assembly sizes

Tilt series were acquired using Tomo5 software on a Titan Krios G4 transmission electron microscope (Thermo Fisher Scientific) equipped with a cold-FEG (operated at 300 kV), a Falcon IVi camera and a Selectris X energy filter. Tilt series were acquired at a magnification of ×81,000, corresponding to a pixel size of 1.63 Å, from −60° to 60°, at 2° tilt increments and using a dose-symmetric tilt scheme. The total dose was 122 e^−^ per Å^2^, and the target defocus varied between −2.5 µm and −4 µm. Data were collected in EER format. Statistical analyses of vesicle size distribution were performed on search maps at ×11,500 magnification using Tomo5. For the first experiment with purified STV-C8 vesicles in PBS, at least 500 search map micrographs were collected for each grid. For the second experiment involving the iodixanol gradient, 1,125 search map micrographs were collected for each grid.

For tomogram reconstruction and segmentation, tilt series were aligned and reconstructed using AreTomo3 (ref. ^43^) (binned by a factor of 4, with a final pixel size of 6.52 Å per pixel). Frame alignment and CTF estimation were performed using the MotionCor3 (ref. ^43^) and GCtfFind^44^ implementations, respectively, in AreTomo3. The aligned tilt series were manually inspected, and problematic tilts were removed before reconstruction. Membranes were segmented using MemBrain-seg, and particles were manually segmented in Amira^45^ (Thermo Fisher Scientific).

Assembly size homogeneity was assessed by morphometric analysis of cryo-electron tomograms. The diameters of 500 individual assemblies were measured directly from tomographic slices using calibrated pixel distances. Measurements were converted to physical units on the basis of the tomogram pixel size and compiled to generate a frequency distribution of assembly sizes.

### Characterization of STV particles obtained from iodixanol gradient

STV-C8(EGFP) vesicles were purified through iodixanol gradients as described in the previous section. Subsequently, RNA was extracted from the three fractions, and the relative amounts of *EGFP* mRNA in the fractions were analysed by RT–qPCR. STV protein content was characterized by HiBiT measurement, and the delivery efficiency was measured by addition of the fractions to target cells and analysis of EGFP expression by flow cytometry after 24 h.

### Prediction of STV-C8 multimer structure

The sequence of STV-C8 (*Ua*PHPLC-SynL-tdPCP-HE0690) was fed into the AlphaFold3 server with octameric settings^46^. The obtained structure file was coloured to represent pLDDT (predicted local distance difference test) scores and was captured in two orientations.

### Live measurement of STV-C8 uptake kinetics

STV-C8 vesicles enveloped with VSV-G were produced and transferred to split-Luc reporter cells, which had been seeded in black-walled 96-well plates the day before. Nano-Glo Endurazine live substrate (Promega) was added to transduced cells, and the plates were subsequently transferred to a Cytation 3 plate reader (Agilent). The luminescence signal from HiBiT/LgBiT reconstituted nanoluciferase was recorded at 15-min intervals for 3 days.

### Characterization of STV-C8 RNA content

STV-C8 particles were produced and purified as described in the previous section. RNA was isolated from the particles, as well as from corresponding producer cells, using a Monarch Total RNA Miniprep Kit (NEB). Subsequently, Illumina RNA sequencing library prep and sequencing with 20 million paired-end reads per sample were performed on a NovaSeq device. Sequencing reads were mapped to the human reference transcriptome using the STAR aligner, and differential expression was analysed using DESeq2. Library preparation, sequencing and data analysis were performed by Azenta (Leipzig).

### Characterization of STV-C8 protein content

STV-C8 were produced and purified as described in the previous section. Total protein was extracted by lysing the sample with lysis buffer (PreOmics) supplemented with cOmplete Protease Inhibitor (Roche). The released protein was quantified by BCA assay (Thermo Fisher Scientific). Then, 10 µg of protein per sample was further processed by filter-aided sample preparation^47^ and measured on a QExactive HFx mass spectrometer online coupled to a Ultimate 3000 RSLC (Thermo Fisher Scientific). Data were analysed by label-free quantification in MaxQuant 2.4.9.0 (MPI)^48^ using a merged dataset comprising the SwissProt human protein database and the sequences of exogenously expressed proteins. Statistics were analysed in Perseus (MPI)^49^.

### RNA and protein gene set enrichment analysis

Significantly enriched or depleted genes (adjusted *P* < 0.005, log_2_[fold change] +3/−3) or proteins (−log q < 0.05, log_2_[fold change] +3/−3) were identified, and gene set enrichment analysis was performed using gProfiler2 with default options (e111_eg58_p18_30541362).

### Native PAGE of purified STV-C8 vesicles

HEK293T cells were transfected with STV-C8 and an unrelated control plasmid in T175 flasks coated with poly-l-lysine (Sigma-Aldrich). Twenty-four hours after transfection, the cells were washed with PBS, and serum-free DMEM was added. Another 24 h later, the supernatant was collected and concentrated by ultracentrifugation as described before. Concentrated supernatants were lysed with M-PER (Thermo Fisher) for 10 min at room temperature. Lysates were mixed in a 1:4 ratio with native PAGE sample buffer (Invitrogen) and loaded on to a 12% Tris-glycine gel (Invitrogen) with a NativeMark Unstained Protein Standard (Invitrogen). The gel was run for 2 h at 150 V using Tris/glycine buffer (Bio-Rad). Afterwards, the gel was stained with Coomassie solution (Thermo Fisher) for 30 min and imaged using a Fusion SL Vilber machine (Peqlab).

### Benchmarking of *EGFP* mRNA delivery efficiency of STVs compared with SEND, EPN and VLP

SEND/MmPeg10 was ordered from Addgene (174858; a gift from F. Zhang), and the EPN-MCP, VLP-MCP, EGFP-MS2 and SEND-EGFP constructs were synthesized by Twist Bioscience and cloned into CAG promoter expression backbones. For each system, the corresponding capsid scaffold and cargo RNA plasmids, encoding EGFP, were cotransfected with the VSV-G plasmid in 24-well format. Supernatants were produced for 48 h and concentrated as previously described. Concentrated vehicles were added to a 96-well plate of HEK293T, Vero E6, N2a and HepG2 cells (all other cell lines were gifts from the Institute of Virology, Helmholtz Munich). Twenty-four hours later, EGFP expression was analysed by flow cytometry as described earlier.

In addition, a reporter plasmid consisting of a lox-stop-lox cassette upstream of the EGFP-coding sequence was cloned. VLP (205525), EPN (205555) and SEND (174858) coding plasmids and Cre cargo plasmids (174862 and 205559) were ordered from Addgene and directly compared with STV-C8 packaging Cre mRNA without any modification of the constructs. Each plasmid was cotransfected with its respective Cre cargo plasmid and VSV-G in a 10-cm dish. The supernatant for each condition was produced for 72 h and concentrated by ultracentrifugation as previously described. HEK293T cells were transfected with the lox-stop-lox reporter plasmid and transduced with two concentrations of concentrated supernatants after 24 h. After a further 24 h, EGFP expression was analysed by flow cytometry.

### Benchmarking of STV against LNP characteristics in vitro and in vivo

A plasmid encoding EGFP under the control of the T7 promoter was cloned. The plasmid was linearized by digestion downstream of the stop codon, leaving a 3′ UTR of similar length to that in the STV cargo plasmid. The reaction product was purified (Monarch DNA Cleanup Kit, NEB) and used as a template for in vitro transcription (HiScribe T7 Quick High Yield RNA Synthesis Kit, NEB). Subsequently, the RNA was purified (Monarch RNA Cleanup Kit, NEB) and capped with a Vaccinia Capping System (NEB). The reaction product was purified again and polyadenylated with *E. coli* poly(A) polymerase (NEB). After a final purification step, the EGFP-coding mRNA was diluted to 150 ng μl^−1^ in 20 mM citrate buffer (pH 4.0). LNPs were composed of ALC-0315 (Cayman Chemical, 34337), DOPE (Avanti Polar Lipids, 850725), cholesterol (ChemCruz, sc-202539) and DMG-PEG 2000 (Avanti Polar Lipids, 880151) in a ratio of 50:10:38.5:1.5. The lipid and RNA solutions were quickly mixed in a 1:3 volume ratio, resulting in a final weight ratio of 40:1. Then, 1 μl of the prepared sample was diluted in 3 ml of PBS in a cuvette (Sarstedt) and analysed by dynamic light scattering using a Zetasizer Pro (Malvern Panalytical). STV-C8 particles containing *EGFP* mRNA were prepared by ultracentrifugation, as described previously. The absolute STV-C8 protein content was determined by extrapolation from a HiBiT Control Protein (Promega) standard curve. EGFP mRNA content of STV-C8 particles was determined by absolute RT–qPCR quantification (Luna Universal One-Step RT-qPCR Kit, NEB) with an in vitro-transcribed *EGFP* mRNA standard, and the STV-C8 particle number was determined by dynamic light scattering.

The delivery efficiency of STV-C8 was compared with that of LNPs on two levels. First, the amount of mRNA required to induce EGFP expression in the same percentage of transfected cells was measured by titration of STV or LNP on target cells and subsequent analysis of EGFP expression by flow cytometry. Second, the expression levels induced by mRNA delivery for the two vehicle types were compared. STV-C8 or LNPs were titrated to induce EGFP expression in 50% of cells. EGFP expression levels at the respective mRNA concentrations were measured by flow cytometry and used to calculate the (theoretical) dose of mRNA required to induce one mean fluorescence intensity unit.

Then, 8-week-old female C57BL/6 mice were intravenously injected with 150 µl of either LNPs or concentrated STV-C8 formulated with Akaluc mRNA (both vehicles were prepared as previously described). At 24 h after administration of the vehicles, mice received 150 µl of 33 mM TokeOni substrate by intraperitoneal injection. Bioluminescence was subsequently measured using an IVIS Lumina S5 imaging system (PerkinElmer) with an exposure time of 20 s. Animal experiments were performed according to the institutional guidelines of the Helmholtz Munich Center German Mouse Clinic after approval of the Ethical Review Board of the Government of Upper Bavaria (Regierung von Oberbayern, Munich, Germany).

### Analysis of STV-C8-induced interferon signalling

A549-IFN-GFP cells (a gift from R. Bartenschlager) that reported interferon signalling by GFP expression were transfected with luciferase plasmid DNA as a positive control for interferon stimulation and treated with STV-C8 particles containing a luciferase mRNA. GFP expression following treatment was monitored after 24 h using an EVOS imaging device (Thermo Fisher Scientific).

In addition, in vitro-transcribed mTagBFP2 mRNA was produced as previously described, using *N*^1^-methylpseudouridine (Jena BioScience) or unmodified uridine. A549-IFN-GFP reporter cells were treated with STV-C8 containing mTagBFP2 mRNA or LNPs containing modified or unmodified mTagBFP2 mRNA to induce similar expression levels of mTagBFP2. Subsequently, activation of IFN–GFP expression was measured by flow cytometry.

### Comparison of LNP and STV-C8-induced cytotoxicity

HEK293T cells were seeded in 96-well plate format, transfected with 50 ng *EGFP* mRNA-containing LNPs and transduced with purified STV-C8(EGFP) particles. Both particle types were used at a concentration that induced EGFP expression in approximately 50% of cells. After 24 h, cells were detached with 0.05% trypsin (Thermo Fisher Scientific), resuspended in Annexin V binding assay buffer (10 mM HEPES, 140 mM NaCl and 2.5 mM CaCl_2_ (pH 7.4)) and labelled 1:100 with Annexin V-iFluor 680 (Abcam). Subsequently, Annexin V staining intensity was quantified using flow cytometry.

### Establishing cell-type-specific STV-C8 by peptide binder engineering

Previously designed EGFRn, EGFRc and IL-7Rα minibinders^28^ or an CD19 scFv were exposed on the STV-C8 surface by expressing them as fusion constructs consisting of a signal peptide, minibinder sequences and a transmembrane domain, along with STV-C8 components and an LDLR-binding deficient mutant of VSV-G (K63Q, R370Q^27^). Transfections were performed in 6-well plates with *EGFP* mRNA cargo, and the supernatant was collected for 48 h and concentrated with Lenti-X concentrator (Takara Bio). Then, 30 μl of concentrated supernatant was transferred to either wild-type HEK293T cells or HEK293T cells stably expressing the EGFR or IL-7Rα receptor, and 24 h later, EGFP expression was analysed by flow cytometry as described earlier.

### STV-C8-mediated *EGFP* mRNA delivery into RPE spheroids isolated from human retinal organoids

Human retinal spheroids were differentiated from a human IPS cell (hiPS cell) line (F49B7) derived from healthy donors and tested for pluripotency markers and germ layer differentiation potential. hiPS cells were seeded on six-well plates coated with Matrigel (Corning) and cultured in mTeSR Plus medium (STEMCELL Technologies). The medium was changed every 2 days. At 70% confluency, iPS cells were passaged in small clumps using 0.5 mM EDTA (0.5 M (pH 8.6); Thermo Fisher Scientific). On day 0, hiPS cells were dissociated as small aggregates using 0.5 mM EDTA. The aggregates were suspended in cold Matrigel (GFR, Corning) and incubated at 37 °C for 20 min to allow gelling. hiPS cell–Matrigel aggregates were gently dispersed in neural induction medium (DMEM/F12 + GlutaMAX, 1% B27 with vitamin A supplement, 0.5% N2 supplement, 0.1 mM 2-mercaptoethanol, 2 mM GlutaMAX and 1% penicillin–streptomycin; all from Thermo Fisher Scientific). The aggregates were cultivated in ultra-low adherent six-well culture plates (Costar, Corning). On day 5, floating cysts were seeded on Matrigel-coated 6-well plates. On day 15, cysts were detached by addition of dispase (0.5 mg m^−1^ in DMEM/F12; STEMCELL Technologies) for 3–4 min at 37 °C, followed by washing with DMEM/F12 medium and growth in the retinal differentiation medium (DMEM/F12 + GlutaMAX, 2% B27 without vitamin A, 1% non-essential amino acids (NEAA) and 1% penicillin–streptomycin; all from Thermo Fisher Scientific). On day 25, immature retinal spheroids were transferred to retinal maturation medium (DMEM/F12 + GlutaMAX, 8% FBS, 2% B27 without vitamin A, 1% NEAA, 1% antibiotic–antimycotic (all from Thermo Fisher Scientific) and 1% 100 mM taurine from Sigma-Aldrich). Half of the medium was changed every 2–3 days, and all spheroids were cultured in a humidified incubator at 37 °C and 5% CO_2_ until the end of the experiment. Retinal pigment epithelium was developed during generation of the retinal spheroids in the form of patches attached to the spheroids. On day 200, RPE spheroids were dissected from human retinal spheroids. Then, they were sorted into a 96-well U-bottomed ultra-low-attachment plate (Nucleon Sphera, Thermo Scientific), with each well containing 3–4 RPE spheroids. The RPE spheroids were transduced with 10 µl of STV-C8(EGFP)/VSV-G or STV-C8(EGFP), fixed 2 days after treatment and then gradually dehydrated in 10% sucrose at room temperature, 30% sucrose at room temperature, and 50% sucrose overnight at 4 °C. The spheroids were embedded in Tissue-Tek O.C.T. compound (Sakura) and immediately frozen at −80 °C until solidification occurred. They were then sectioned at 10-µm thickness using a cryostat (Leica CM3050 S, Leica Biosystems). Cryosections were rehydrated and incubated in a 5% chemo-blocker solution (Merck) for 30 min, followed by 30 min incubation in 0.3% Triton X. Sections were incubated overnight at 4 °C with anti-RPE65 (Proteintech, 17939-1-AP) and anti-GFP (Santa Cruz, sc-101536) primary antibodies diluted in 5% chemo-blocking solution; the sections were washed three times in PBS, then were further incubated for 1 h at room temperature with goat anti-rat Alexa Fluor 488 (Thermo Fisher Scientific) and donkey anti-rabbit Alexa Fluor 555 (Thermo Fisher Scientific) secondary antibodies diluted in 5% chemo-blocking solution. Finally, the sections were washed with PBS and mounted using Fluoroshield with DAPI (Sigma-Aldrich). Immunolabelled RPE spheroids were imaged using a Leica TCS SP8 spectral confocal laser scanning microscope (Leica Microsystems).

### EGFP delivery into human monocytes

Primary human monocytes (ATCC, CRL-3622) were seeded in 96-well format. Then, 5 µl of concentrated *EGFP-*mRNA-containing STVs were added to the cells, and EGFP expression was analysed by flow cytometry 24 h later.

### Isolation of primary astroglia from mouse postnatal cortex and *Ascl1* mRNA delivery

Primary astrocytes were isolated from the cerebral cortex of postnatal day 5 C57BL/6N mice. The cortex was isolated, cut into small pieces and mechanically dissociated by vigorous pipetting. Subsequently, the cell suspension was centrifuged for 7 min at 1,300 rpm, and the cell pellet was plated in a T25 flask and cultivated for 7–13 days in DMEM/F12 + GlutaMAX, supplemented with 10% FBS, 10% penicillin–streptomycin, 5% horse serum, 4.5 g l^−1^ d-(+)-glucose, 2% B27, 10 ng ml^−1^ bFGF and 10 ng ml^−1^ EGF (all from Thermo Fisher Scientific). After reaching 90% confluency, the cells were passaged using 0.05% Trypsin/EDTA (Thermo Fisher Scientific), and approximately 75,000 cells were seeded on to glass coverslips coated with poly-d-lysine (Sigma-Aldrich). Twenty-four hours later, 15 µl of concentrated EGFP or Ascl1-P2A-EGFP-containing STV-C8 was added to the cells. After 48 h, cells were fixed in 10% formalin (Sigma-Aldrich) and incubated with anti-GFP (Abcam, ab13970) or anti-Mash1 (Abcam, ab211327) primary antibody in PBS containing 1% BSA (Sigma-Aldrich) and 0.3% Triton X-100 (Sigma-Aldrich) overnight at 4 °C. After washing, the cells were stained with Alexa 488-coupled donkey anti-chicken (Dianova, 703-546-155) or Alexa594-coupled donkey anti-rabbit (Thermo Fisher Scientific, A21207) secondary antibody for 1–2 h in the dark at room temperature. Subsequently, cells were stained with DAPI, coverslips were mounted using Aqua Poly/Mount (Polyscience), and samples were imaged using an Axio Imager M2 fluorescence microscope (Carl Zeiss).

### Deletion of exon 51 of dystrophin gene in primary porcine fibroblasts

Two Cas9 sgRNA plasmids containing a PP7 motif in the stem–loop of the sgRNA, along with porcine dystrophin targeting spacers, were cloned. The sgRNAs targeted intron 50 (AGAGTTCCTAAGGTAGAGAG) and intron 51 (ATAAAGATAAGAGCTGGCAG) to delete exon 51 (ref. ^13^). In addition, a plasmid encoding nuclear localization signal (NLS)- and nuclear export signal (NES)-fused Cas9, along with a 3′ UTR PP7 motif, was cloned. HEK293T producer cells were seeded in 10-cm dishes coated with poly-l-lysine and cotransfected with Cas9 mRNA and the two sgRNA plasmids (in a 1:1:1 ratio), along with STV-C8 and VSV-G coding plasmids. STV-C8 particles were collected and concentrated by ultracentrifugation as described before. Pig primary fibroblasts were seeded in a collagen-coated 48-well plate in DMEM^50^ supplemented with 1% NEAA, 10 mM HEPES and 15% FBS (all from Thermo Fisher Scientific) and 2-mercaptoethanol (Merck). Seeded cells were treated with 20 µl STVs for 72 h. Subsequently, genomic DNA was extracted using a Monarch Genomic DNA Purification Kit (NEB), and a 2-kb fragment covering the deleted region was amplified (primers: CCCATGACATTTACCCTATTATTATCCC and GCTAATGTTCATTTTAAAAAGGAATCTGTC) using Platinum SuperFi II Master Mix (Thermo Fisher Scientific). The PCR product was run on a 1.5% agarose gel and imaged.

### Treatment of SARS-CoV-2-infected iPS cell-derived human lung cells with STV-delivered Cas13d-NCS

For lung cell differentiation, hiPS cells (ISFi001-A, RRID: CVCL_YT30) were cultured in StemMACS medium (Miltenyi Biotec) on plates coated with Geltrex Reduced Growth Factor (Thermo Fisher Scientific). At 70% confluence, iPS cell colonies were isolated as a single-cell suspension with Accutase (Thermo Fisher Scientific) for 5 min at 37 °C, neutralized with StemMACS medium and centrifuged for 3 min at 200*g* at room temperature; then, 1.0–1.2 × 10^6^ cells were seeded on to non-adherent 6-well plates (Corning, 3471) in StemMACS medium supplemented with 10 μM Y-27632 (Enzo Life Sciences). Differentiation basal medium (DBM) was prepared with DMEM/F12 1:1 and GlutaMAX (Thermo Fisher Scientific) supplemented with 1× NEAA (Thermo Fisher Scientific), 0.1% Albumax (Thermo Fisher Scientific) and 1× B27 (Thermo Fisher Scientific). Formation of embryonic bodies was induced by changing the medium to 50% StemMACS medium/50% DBM with 20 ng ml^−1^ activin A (Bio-Techne). The medium was replaced entirely with DBM with 20 ng ml^−1^ activin for 48 h. Definitive endoderm (days 0 to 5) was induced by plating embryonic bodies on to plates coated with Geltrex Reduced Growth Factor at 7 embryonic bodies per cm^2^ of culture surface in DBM supplemented with 150 ng ml^−1^ activin A and 25 ng ml^−1^ bone morphogenic protein 4 (BMP4) (Thermo Fisher Scientific) for 5 days with daily medium changes. Anteriorization of definitive endoderm (days 6 to 10) was elicited by changing DBM supplements to 50 ng ml^−1^ EGF (Invitrogen), 50 ng ml^−1^ bFGF (Thermo Fisher Scientific), 3 μM SB431542 (Miltenyi Biotec) and 10 ng ml^−1^ Noggin (Sigma-Aldrich) for 5 days with medium changes every day. Lung progenitors giving rise to type II alveolar epithelial cells (days 10 to 17) were generated by changing the medium to DBM containing 50 ng ml^−1^ BMP2 (Thermo Fisher Scientific), 50 ng ml^−1^ FGF10 (Peprotech), 50 ng ml^−1^ BMP4, 50 ng ml^−1^ bFGF and 50 ng ml^−1^ WNT3A (Bio-Techne) for 7 days. Successful differentiation into alveolar epithelial cells was confirmed by analysis of expression of ACE2 and SLC34A2 by RT–qPCR (Luna Universal One-Step RT-qPCR, NEB). In addition, NLS- and NES-containing Cas13d-NCS^32^ was cloned into a PP7-motif-containing backbone in the 3′ UTR, and a PP7 motif was attached 3′ to a crRNA, targeting the SARS-CoV-2 3′ UTR region (GUCAUCCAAUUUGAUGGCACCUG). Subsequently, lung progenitor cells were seeded into Geltrex-coated 96-well plates at a density of 2 × 10^4^ cells per well (Merck) and differentiated for 7 days in differentiation medium. Differentiated lung cells were transduced with 40 μl concentrated STV-C8 containing Cas13d-NCS/SARS-CoV-2 or non-target crRNA. Twenty-four hours later, the cells were infected with SARS-CoV-2-GFP (multiplicity of infection: 10), and viral replication was monitored for 72 h in an Incucyte S3 live imaging system (Sartorius).

### Analysis of STV-C8 inactivation in human blood samples

Peripheral blood mononuclear cells (PBMCs) were isolated by diluting blood in 2–4 times the volume of PBS. Then, 35 ml of the diluted blood suspension was carefully layered on to 15 ml of Ficoll (density = 1.077 g ml^−1^) in a Falcon tube, and the tube was centrifuged without brake at 400*g* for 30 min at 20 °C. After centrifugation, the upper layer was aspirated, leaving PBMCs at the interphase. The PBMC layer was transferred to a fresh Falcon tube, which was filled with PBS and centrifuged again at 300*g* for 10 min at 20 °C. The resulting cell pellet was resuspended in PBS, and cell counting was performed using Trypan blue staining. For long-term storage, PBMCs were frozen at a density of 1 × 10^7^ cells ml^−1^ in FBS supplemented with 20% dimethyl sulfoxide. Blood samples were collected in EDTA-free tubes for isolation of blood serum. The tubes were gently inverted several times to mix the blood, and the samples were then allowed to clot at 4 °C for 3–4 h. After clotting, the samples were centrifuged at 2,500*g* for 10 min at room temperature. The top clear layer (serum) was carefully transferred to new sterile microcentrifuge tubes or storage vials using a sterile pipette. For long-term storage, aliquoted serum was stored at −80 °C. STV-C8(N-split-Luc) particles were produced in 24-well plates and collected for 48 h. The collected supernatant was concentrated using Lenti-X (Takara Bio) as described. Then, 30 μl of concentrated STV-C8 particles were mixed with 30 μl of 1:10 diluted serum and 30 μl of resuspended PBMCs (approximately 3.0 × 10^5^ cells) or PBS and incubated at 37 °C for 60 min. After the incubation period, 50 μl of STV-C8 with PBMCs or serum mix was transferred to a 96-well plate of split-Luc reporter cells. The next day, N-split-Luc RNA expression was analysed using ONE-GloEX Luciferase (Promega) assay.

### Testing of STV-C8 storage conditions

STV-C8(N-split-Luc) were produced in 6-well format for 2 days, concentrated using Lenti-X (Takara Bio), and stored for 7 days at 4 °C or −80 °C. Subsequently, 50 µl of stored samples was added to split-Luc reporter cells. The next day, N-split-Luc RNA expression was analysed using ONE-GloEX Luciferase (Promega) assay.

### Delivering of OpenCRISPR-1 with STV-C8

The coding sequence of OpenCRISPR-1 was ordered from Twist Bioscience and cloned into a CAG-promoter-containing expression plasmid. The coding sequence was fused to two NLS signals and one NES signal, and the PP7 aptamer was added to the 3′ UTR. In addition, an sgRNA containing the PP7 aptamer in the stem–loop region and a spacer targeting the stop codon in eTLR cells was cloned (GCUCCCACAACGAAGACUGAC; the cells were a gift from the Institute of Synthetic Biomedicine, Helmholtz Munich)^30^. STV-C8 particles containing OpenCRISPR-1 or Cas9 and the sgRNA were produced in 6-well format for 3 days and concentrated using Lenti-X (Takara Bio), and 20 µl concentrated particles were added to a 96-well plate of eTLR cells. Three days later, the cells were imaged using an EVOS imaging device (Thermo Fisher Scientific).

### Analysis of mouse whole-body biodistribution of STV-C8-mediated EGFP expression

STV-C8(EGFP) or empty STV-C8 vesicles were produced in coated 10-cm dishes for 3 days and concentrated by ultracentrifugation as described before. Then, 50 µl of concentrated STV-C8(EGFP) samples were injected intravenously into 4-week-old female BALB/c wild-type mice (Charles River Laboratories) in accordance with institutional animal care guidelines and with approval by the Ethical Review Board of the Government of Upper Bavaria (Regierung von Oberbayern, Munich, Germany). The mice were euthanized 24 h or 72 h after injection and intracardially perfused with heparinized PBS (10 U ml^−1^ heparin) and 4% paraformaldehyde. The skin was removed, and the bodies were fixed in 4% paraformaldehyde overnight at 4 °C. Then, vDISCO whole-body staining and clearing were performed as previously described^33^; in brief, the steps comprised decolourization (25% CUBIC reagent in PBS), decalcification (10% (w/v) EDTA in PBS), signal-enhancement with anti-GFP nanobodies (Chromotek, anti-GFP-AF647), dehydration (with tetrahydrofuran), delipidation (with dichloromethane) and refractive-index matching with a mixture of benzyl alcohol and benzyl benzoate. A Blaze light-sheet system (LaVision BioTec) with an axial resolution of 4 µm was used for light-sheet imaging. Full-scale mouse body imaging was performed using a ×4 magnification objective (Olympus XFLUOR ×4 corrected/0.28 numerical aperture; working distance: 10 mm). High-magnification tile scans were obtained with 22% overlap, and the light-sheet width was reduced to 80%. For the z-step, the size was set to 6 µm, with time exposures of 40 ms in the background channel (488 nm) and 60 ms in the signal channel (640 nm, 647-boosted GFP signal). A Fiji plugin was used to stitch the raw TIFF files to a full plane. The individual planes were merged into a three-dimensional file format with Imaris converter and visualized using Imaris^33^.

### Probing potential immunological response and liver toxicity after systemic STV-C8 injection in mice

PBS (as a control for untreated mice) or 50 µl of STV-C8 was injected intravenously into female C57BL/6J mice (11 weeks old, Charles River Laboratories). On days 1 and 3 post-injection, mice were euthanized using carbon dioxide (CO_2_) inhalation in accordance with institutional animal care guidelines and with the approval of the Ethical Review Board of the Government of Upper Bavaria (Regierung von Oberbayern, Munich, Germany) (ROB-2532.Vet_02-23-143). Blood samples were collected by means of cardiac puncture, and liver tissues were immediately excised for subsequent analysis. Serum alanine aminotransferase levels were measured by ALAT (GPT) FS (IFCC mod.) assay on a respons910 random-access clinical chemistry analyser (DiaSys Diagnostic Systems). Liver tissue was homogenized, and total RNA was extracted from the aqueous phase using a NucleoSpin RNA Mini Kit (Macherey-Nagel). Then, 400 ng of RNA was reverse transcribed into complementary DNA (cDNA) using a PrimeScript RT Reagent Kit (TaKaRa), and RT–qPCR of RNA samples was performed using PowerUp SYBR Green Master Mix (Applied Biosystems) on a QuantStudio 3 Real-Time PCR System (Applied Biosystems).

### *EGFP* mRNA delivery into differentiated mouse myotubes

C2C12 mouse myoblasts (a gift from the Institute of Developmental Genetics, Helmholtz Munich) were differentiated into myotubes by cultivation in DMEM supplemented with 2% horse serum. Successful differentiation was verified through formation of multinucleated cells. Differentiated myotubes were then treated with STV-C8(EGFP) for 2 days, and EGFP expression was analysed by flow cytometry.

### In vivo treatment of porcine muscle cells to delete exon 51 from the dystrophin gene

Large animal work was approved and ethically monitored by the Bavarian local authority (ROB-55.2-2532.Vet_02-19-39). STV-C8(Cas9/sgRNA) were produced in coated 10-cm dishes and concentrated by ultracentrifugation as described before. To provide a proof of concept, a 3-month-old 25-kg wild-type German landrace pig was sedated by intramuscular injection of ketamine and azaperone. Fentanyl was injected intravenously through a 20-G catheter in the ear vein to provide analgesia during the procedure. Subsequently, the prospective injection site on the right hind limb was shaved and disinfected, and 1 ml of concentrated STV-C8 vector was injected at 1.75 cm depth, using a 1-ml Luer lock syringe equipped with a 22-G safety needle, into the right M. biceps femoris. After injection, the pig was monitored until regaining consciousness and assessed twice daily for the following 3 days for clinical signs of infection, inflammation or any adverse reaction to the injection. After 3 days, the animal was sedated according to the protocol above and euthanized by intravenous injection of pentobarbital. Systematic tissue sampling surrounding the injection site and the uninjected contralateral leg, as well as the M. latissimus dorsi, was performed after cardiac arrest. Genomic DNA was extracted from all procured samples using a Monarch Genomic DNA Purification Kit (NEB). PCR was performed using Platinum SuperFi II Master Mix (Thermo Fisher Scientific) was performed with primers CCCATGACATTTACCCTATTATTATCCC and GCTAATGTTCATTTTAAAAAGGAATCTGTC, and the deletion efficiency was assessed on an agarose gel by comparison of band intensities. The resulting bands at 2 kb (wild type) and 1 kb (genomic deletion) were extracted from the gel and verified by Sanger sequencing (Microsynth). In addition, the PCR product was sequenced using Oxford Nanopore sequencing (Eurofins Genomics), and the deletion frequency was analysed using Geneious Prime (2025.1.2, Dotmatics).

### Analysis of immunological response to local STV-C8 injection in pig muscle

RNA was extracted from muscle tissues by phenol–chloroform extraction. Induction of inflammation-related genes after STV-C8 treatment was evaluated by RT–qPCR in four experimental groups, using *TBP* as an endogenous control gene. The target group consisted of muscle samples from STV-C8-injected regions. Non-injected muscles from the same animal and muscle samples from untreated animals were used as negative controls, whereas muscle samples from rejected tissue were used as positive indicators of strong inflammatory response.

### iPS cell differentiation into myotubes and treatment of patient-derived cells

Skeletal muscle differentiation of control and DMDΔ52 hiPS cells was performed using an SKM-KIT (Amsbio). For STV-C8 transduction, wild-type or patient-derived myoblasts were seeded at a density of 40,000 cells cm^−2^ on collagen I-coated plates (5 µg cm^−2^; Sigma-Aldrich, 122-20) in skeletal muscle myoblast medium (SKM02). At confluence, myoblasts were switched to skeletal muscle myotube medium (SKM03) to induce differentiation. After 4 days, myotubes were transduced with STV-C8 or left untreated. Live-cell imaging was performed 24 h and 72 h after transduction, followed by fixation at 72 h and DAPI staining. For STV-C8(Cas9)-treated conditions, RNA was collected following transduction at 96 h. Subsequently, the RNA was converted into cDNA using a Maxima First Strand cDNA Synthesis Kit (Thermo Fisher) and amplified with primers binding to exons 49 and 54 of the *DMD* gene. In addition, RNA from *RPS18* was amplified to confirm the quality of the extracted RNA. The expected sizes of the amplified cDNAs from treated and untreated cells were analysed by agarose gel electrophoresis, and the integrity of the splicing events was confirmed by nanopore sequencing of the PCR product.

### Statistical analysis and reproducibility

Statistical tests of the numerical data were performed and graphical representations were produced using GraphPad Prism. Unless otherwise stated, immunofluorescence images and gel electrophoresis images are representative of at least three independent experiments. Sample sizes were chosen on the basis of previous experience and expected experimental variability, with at least biological duplicates unless otherwise stated. For in vivo studies, animals were randomly assigned to experimental groups after enrolment. Blinding was not applied to most in vitro and molecular experiments, because outcomes were based on objective quantitative measurements analysed using predefined criteria or standardized analysis pipelines. Samples from different groups were processed in parallel under identical conditions, and automated image analysis or computational data processing was used where applicable to minimize bias.

### Reporting summary

Further information on research design is available in the Nature Portfolio Reporting Summary linked to this article.

## Online content

Any methods, additional references, Nature Portfolio reporting summaries, source data, extended data, supplementary information, acknowledgements, peer review information; details of author contributions and competing interests; and statements of data and code availability are available at https://doi.org/10.1038/s41586-026-10952-3.

## Supplementary information

Supplementary Information Supplementary Note 1, Figs. 1–9, exemplary gating strategy, Table 1 and references.

Reporting Summary

Peer Review File

## Source data

Source Data Figs. 1–3 and Extended Data Figs. 1, 2 and 4–10.

## Data Availability

All data are available in the manuscript or Supplementary Information. The RNA sequencing data generated in this study have been deposited in the NCBI Sequence Read Archive under accession code PRJNA1489338 and in the NCBI Gene Expression Omnibus under accession code GSE338002. Source data are provided with this paper.
