# Supplementary material for: Creating bottom-up RNA transfer vehicles from synthetic protein assemblies
Source: Nature. 2026 Sep 2;658(8136):805–13. doi: 10.1038/s41586-026-10952-3 (PMC13668671; doi:10.1038/s41586-026-10952-3)
Supplement: Supplementary file 1 [file 41586_2026_10952_MOESM1_ESM.pdf]

nature portfolio

https://doi.org/10.1038/s41586-026-10952-3

Supplementary information

Creating bottom-up RNA transfer vehicles from synthetic protein assemblies

In the format provided by the authors and unedited

Creating bottom-up RNA transfer vehicles from synthetic protein assemblies

Maren Kirstin Schuhmacher [*,1,2], Christoph Gruber [#,*,1,2], Christopher M. R. Lang [*,1,2], Ricardo M. W. Ruijpers [*,1,2], Lyupka Mazneykova [*,1,2], Brice Beinsteiner [3], Ariane Krus [1,2], Barbara Tremmel [1,2], Friederike Reinhardt [1,4], Karoline Kadletz [5,6], Zhe Ma [7], Lucie Casalta [8,9], Josep Miquel Cambra Bort [8,9], Dina Y. Otify [10], Iolo Balken [1,2], Leon Hetzel [11,12], Juliane Merl-Pham [13], Tatjana Dorn[14,15], Marina Luchner [1,4], Lea Bauersachs [1,4], Karin Ganea_[1,2], Natascha Wieser [1,2], Alexander Emrich [1,4], Emirhan Yağmur [1,2], Katrin Rager [1,2], Gauhar Sagindykova [1,2], Niklas Armbrust [4,16], Julian Geilenkeuser [4,16], Gil G. Westmeyer [4,15,16], Elvir Becirovic [17], Martin Biel [10], Rouzanna Istvanffy [15,18], Daniela M. Vogt Weisenhorn [1,2], Dong-Jiunn Jeffery Truong [4,15,16], Fabian J. Theis [11,12,19], Gregor Ebert [7], Alessandra Moretti[14,15], Ali Ertürk [5,6,20,21,22], Andrea Bähr [8,9], Christian Kupatt[23,24], Marion Jasnin [3,25,26], Nikolai Klymiuk [8,9], Florian Giesert [#,1,2] and Wolfgang Wurst [#,1,2,21,27,28]

[*] Authors contributed equally: Maren Kirstin Schuhmacher, Christoph Gruber, Christopher M. R. Lang, Ricardo M. W. Ruijpers, Lyupka Mazneykova

[#] Authors jointly supervised this work: Christoph Gruber, Florian Giesert, Wolfgang Wurst

[1] Institute of Developmental Genetics, Helmholtz Munich, Oberschleißheim, Germany
[2] TUM School of Life Sciences, Technical University of Munich, Freising, Germany
[3] Helmholtz Pioneer Campus, Helmholtz Munich, Oberschleißheim, Germany
[4] Department of Bioscience, TUM School of Natural Sciences, Technical University of Munich, Garching, Germany
[5] Institute for Tissue Engineering and Regenerative Medicine, Helmholtz Munich, Oberschleißheim, Germany
[6] Deep Piction GmbH, Munich, Germany
[7] Institute of Virology, Helmholtz Munich, Oberschleißheim, Germany
[8] Large Animal Models in Cardiovascular Research, Klinik und Poliklinik für Innere Medizin I, Klinikum rechts der Isar, Technical University Munich, Munich, Germany
[9] Center for Innovative Medical Models, LMU Munich, Munich, Germany
[10] Department of Pharmacy-Center for Drug Research, LMU Munich, Munich, Germany
[11] Institute of Computational Biology, Helmholtz Munich, Oberschleißheim, Germany
[12] Department of Mathematics, TUM School of Natural Sciences, Technical University of Munich, Garching, Germany
[13] Metabolomics and Proteomics Core, Helmholtz Munich, Oberschleißheim, Germany
[14] First Department of Medicine and Regenerative Medicine in Cardiovascular Diseases, Klinikum rechts der Isar, Munich, Germany
[15] TUM School of Medicine and Health, Technical University of Munich, Munich, Germany
[16] Institute for Synthetic Biomedicine, Helmholtz Munich, Oberschleißheim, Germany
[17] Laboratory for Retinal Gene Therapy, Department of Ophthalmology, University Hospital Zurich, University of Zurich, Schlieren, Switzerland
[18] Department of Surgery, Klinikum rechts der Isar, Technical University of Munich, Munich, Germany
[19] Wellcome Trust Sanger Institute, Wellcome Genome Campus Hinxton, Cambridgeshire, UK
[20] Institute for Stroke and Dementia Research, Medical Centre of the University of Munich, LMU Munich, Munich, Germany
[21] Cluster for Systems Neurology (SyNergy), Munich, Germany
[22] Graduate School of Neuroscience (GSN), Munich, Germany
[23] Klinik und Poliklinik für Innere Medizin I, Klinikum rechts der Isar, Technical University of Munich, Munich, Germany
[24] DZHK (German Center for Cardiovascular Research), partner site Munich Heart Alliance, Munich, Germany
[25] Department of Chemistry, TUM School of Natural Sciences, Technical University of Munich, Garching, Germany
[26] Helmholtz Munich Cryo-Electron Microscopy Platform, Helmholtz Munich, Oberschleißheim, Germany
[27] Deutsches Zentrum für Psychische Gesundheit (DZPG), Site Munich-Augsburg, Germany
[28] German Center for Neurodegenerative Diseases (DZNE), Munich, Germany
[29] Emeritus of Excellence Senior Faculty (SEF), Technical University of Munich, Munich, Germany

Figures, data, and information to supplement the main manuscript.

Supplementary Note 1 | Detailed description of the STV screening and reporter system. **P3**

Supplementary Fig. 1 | Uncropped Western Blots and protein gels. **P5**
Supplementary Fig. 2 | Additional characterization of STV-C8 vesicle size and content. **P6**
Supplementary Fig. 3 | Modeling of the STV-C8 structural features. **P6**
Supplementary Fig. 4 | in vivo comparison of STV-C8 and LNP-mediated mRNA delivery. **P6**
Supplementary Fig. 5 | Additional probing of immunogenicity and cargo programmability. **P7**
Supplementary Fig. 6 | Further characterization of RPE spheroid and lung cell models. **P7**
Supplementary Fig. 7 | Uncropped Agarose gels. **P8**
Supplementary Fig. 8 | Additional analysis of STV-C8 whole-body tissue distribution. **P10**
Supplementary Fig. 9 | Extended analysis of STV-C8 treated myotubes. **P10**

Exemplary gating strategy **P11**

Supplementary Table 1 | Protein, RNA, and DNA sequences used in this study **P12**

References **P43**

Supplementary Note 1 | Detailed description of the STV screening and reporter system

The Split-luc reporter system quantifies three sequential steps in the STV workflow in a single experiment. STV release from producer cells is monitored by complementation of HiBiT tag[1], which is part of the STV protein, with externally supplemented LgBiT protein. The addition of Furimazine (NanoLuciferase substrate) induces a light signal which is proportional to the amount of STV/HiBiT protein present in the supernatant. STV Uptake into target cells is measured by intracellular complementation of reporter cell expressed LgBiT and STV-fused HiBiT tag. Functional RNA transfer is reported by the reconstitution of wild-type Firefly luciferase through coiled-coil assisted split intein splicing of the C-split part of Firefly luciferase, expressed in reporter cells, and STV delivered mRNA coding for the N-split part of Firefly luciferase. Upon cell entry, the mRNA is expressed, the affinity of the N5/N6 coils brings the GP41-1 split intein in proximity and induces splicing to reconstitute full-length Firefly luciferase that reports efficiency of mRNA delivery[2,3].

Architecture of the reporter system

1. **Reporter cell line**

   The reporter cell line stably expresses two components: LgBiT, which is the large fragment of NanoLuciferase. LgBiT resides in the cytosol and can complement with HiBiT to form active NanoLuciferase. And C-Fluc fused to two helper modules. The first is the heterodimeric coiled-coil N6. The second is the C intein of the GP41-1 split intein. This fusion enables conditional reconstitution of full-length Firefly luciferase only when the matching N-Fluc fusion is present in the same cell.

2. **Producer cell configuration**

   Producer cells express the STV protein assembly together with a PP7-tagged cargo mRNA. The cargo encodes N-Fluc that is fused to the N intein GP41-1 and the N5 coiled-coil partner. The PP7 hairpins allow selective packaging through a cognate PP7 binding protein in the STV architecture.

3. **STV constructs**

   The STV constructs carry a HiBiT tag. HiBiT complements with LgBiT in supernatant or target cells to form an active NanoLuciferase enzyme.

Readout of the reporter system

1. **STV release from producer cells**

   Supernatants from producer cells contain HiBiT-tagged STV particles. In the release assay, exogenous LgBiT present in the detection reagent complements HiBiT and generates a light signal. The signal scales with the number of HiBiT-tagged particles and reports the efficiency of assembly and budding.

2. STV uptake by reporter cells

Reporter cells express intracellular LgBiT. After exposure to STVs and subsequent uptake, HiBiT complements with cellular LgBiT and produces a light signal in cell lysates. The signal requires particle binding and internalization, which makes HiBiT accessible in the cytosol. This readout is independent of RNA packaging and translation and therefore isolates the entry step.

3. Functional RNA transfer

The reporter cells additionally express C-Fluc fused to an N6 coil and the GP41-1 C intein. The STV expressing producer cell packages a PP7-tagged mRNA that encodes N-Fluc fused to N5 and the GP41-1 N intein. Delivery of this mRNA and its translation in the reporter cell produces the N-Fluc fusion protein. The N5 and N6 coils heterodimerize and bring the two split intein halves into proximity. The GP41-1 split intein performs protein trans splicing and generates a covalently joined, wild-type Firefly luciferase that is measured with luciferin.

Supplementary Figures

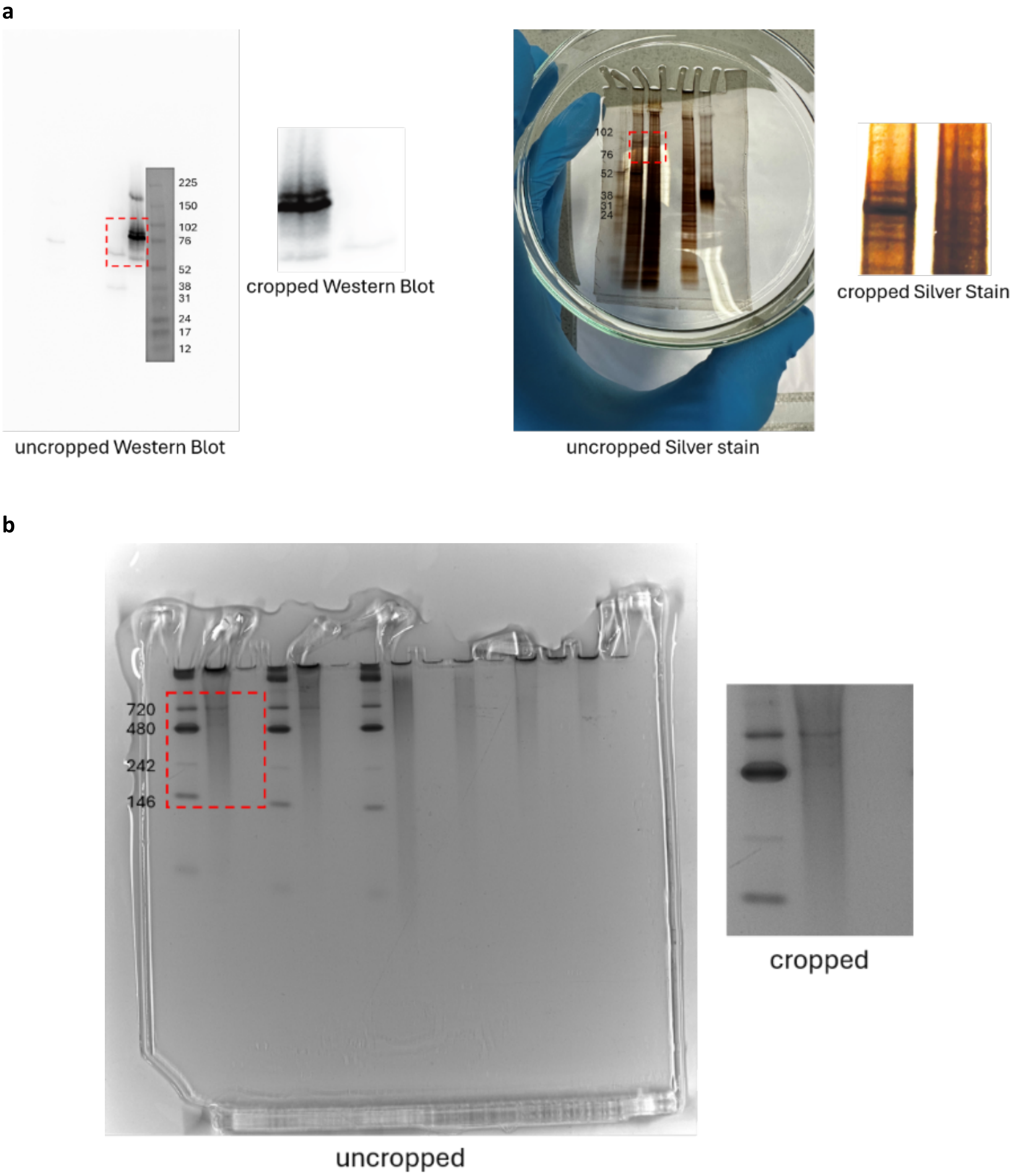

Supplementary Fig. 1 | Uncropped Western Blots and protein gels. a, Full-size western blot image and silver stain corresponding to Extended Data Fig. 5a. **b,** Uncropped native page of Extended Data Fig. 6e.

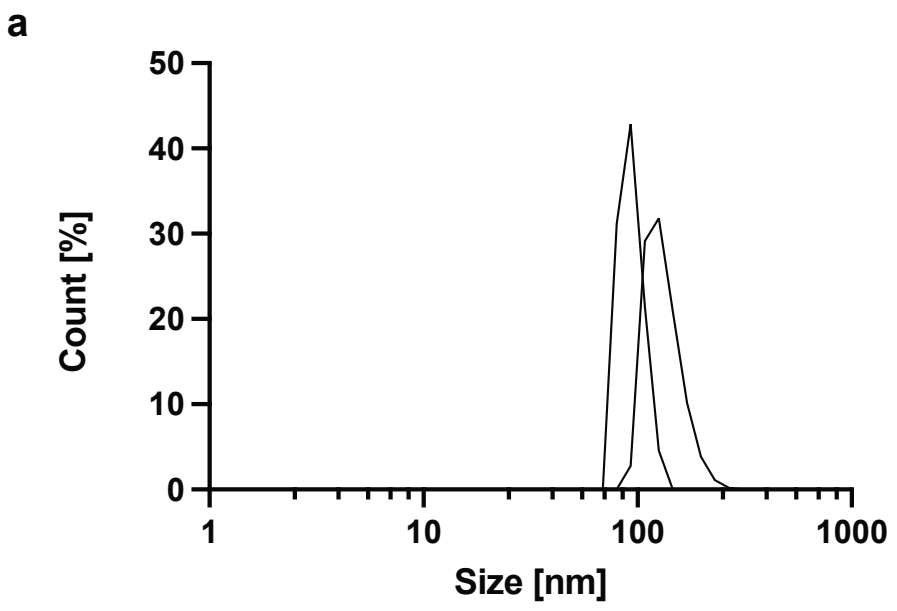

b

Vector	Volume (µL)	RNA content (pg)	STV protein molecules (fmol)	Particle number
STV-C8 (uncon.)	1	1	0.27	2.4-36 e4
LNP	1	110.000	NA	1.3-16 e5

Supplementary Fig. 2 | Additional characterization of STV-C8 vesicle size and content. a, Analysis of purified STV-C8 particle size by DLS in duplicates. **b,** Summary of STV-C8 particle characteristics in comparison to LNPs.

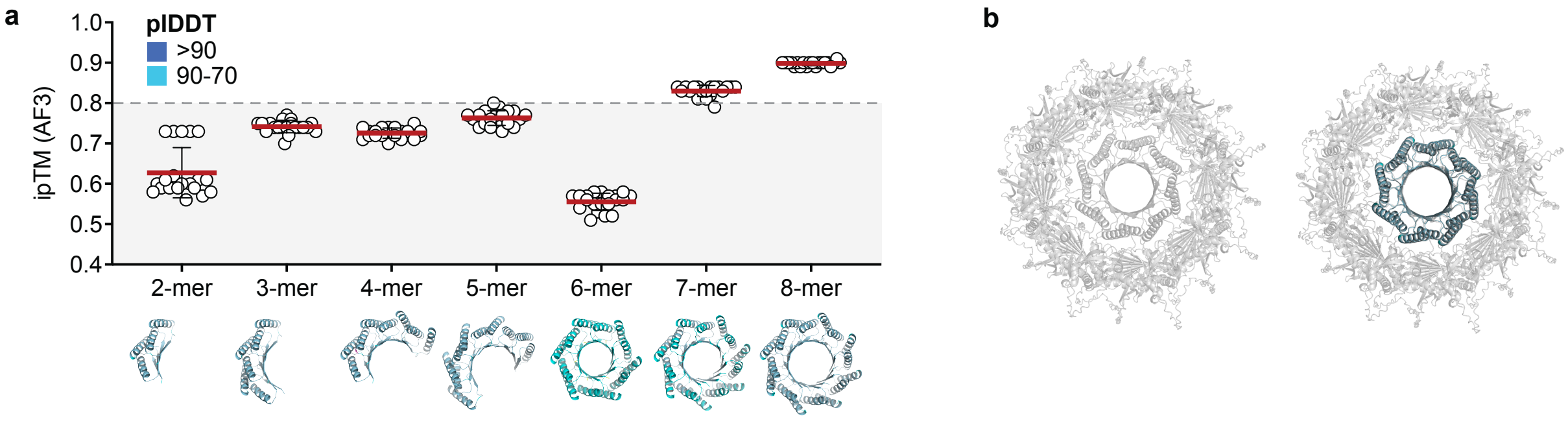

Supplementary Fig. 3 | Modeling of the STV-C8 structural features. a, AlphaFold3-based prediction of the HE0690 oligomeric state. An ipTM threshold of 0.8 is considered as highly confident. **b,** Structural alignment of the predicted HE0690 scaffold (blue) with the STV-C8 oligomer (grey).

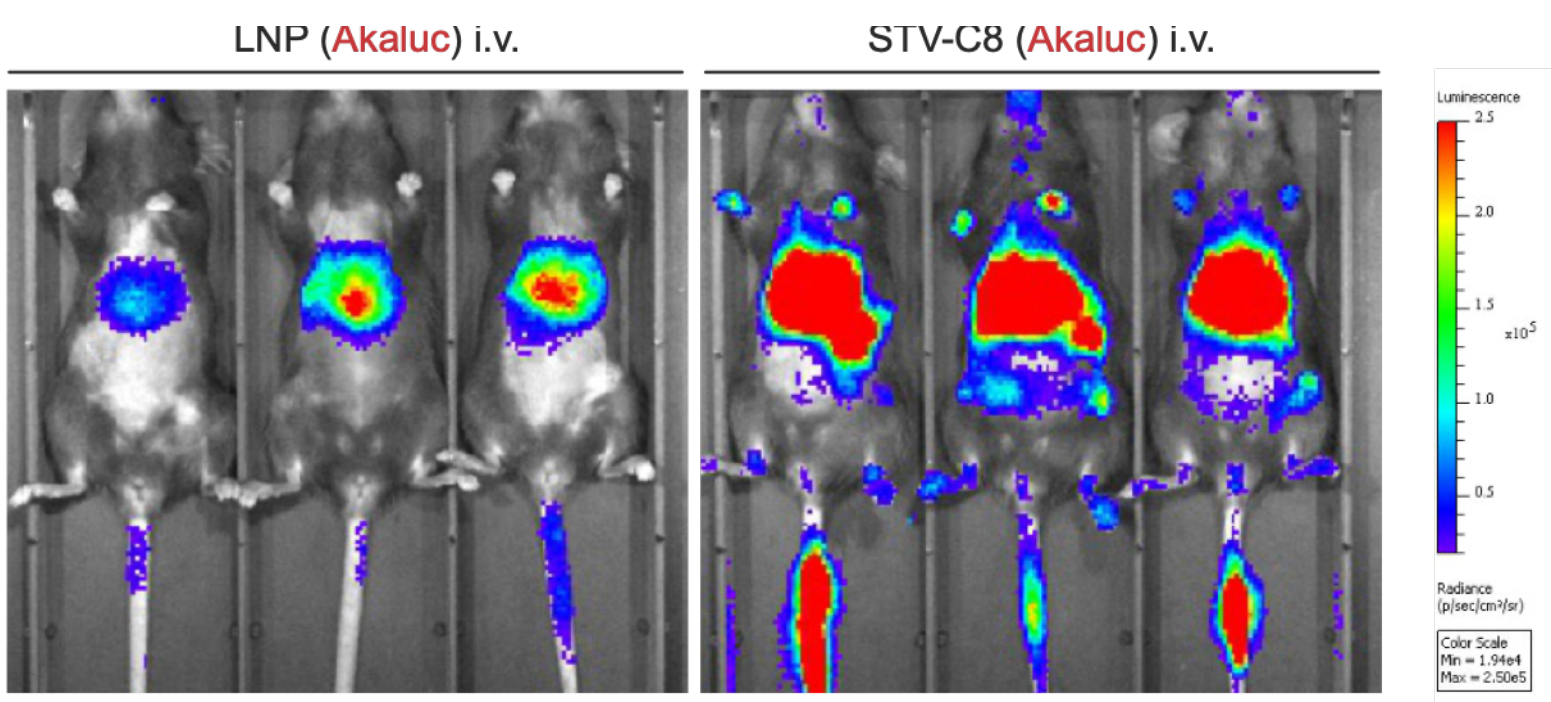

Supplementary Fig. 4 | *in vivo* comparison of STV-C8 and LNP-mediated mRNA delivery. Female C57BL/6 mice (n = 3) were intravenously injected with LNPs or STV-C8 carrying Akaluc mRNA and imaged 24 h later after substrate administration.

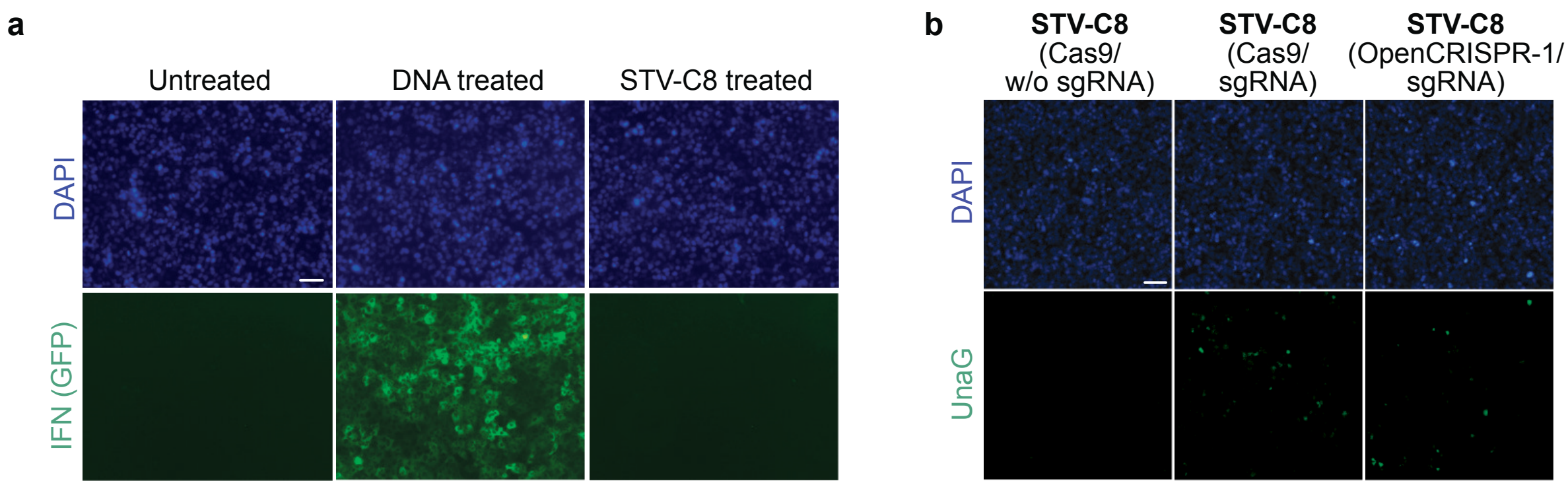

Supplementary Fig. 5 | Additional probing of immunogenicity and cargo programmability. a, Induction of GFP expression under the control of the IFN-β promoter in A549 reporter cells upon treatment with STV-C8 or plasmid transfection (scale bar: 50 µm). **b,** Imaging of TLR reporter cells 3 d after treatment with STV-C8 vehicles containing CRISPR gene editing systems (scale bar: 50 µm).

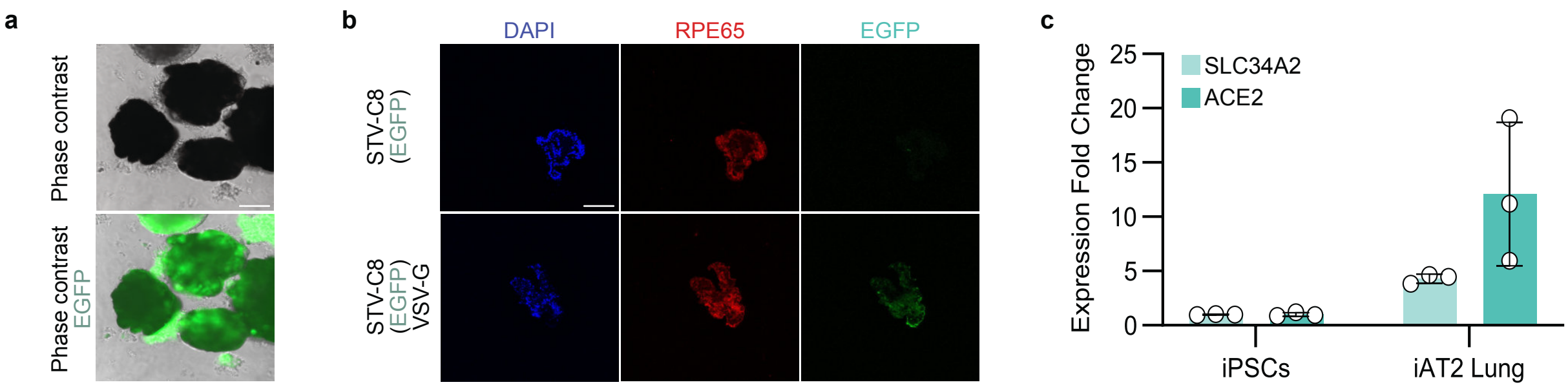

Supplementary Fig. 6 | Further characterization of RPE spheroid and lung cell models. a, Imaging of STV-C8(EGFP) treated RPE spheroids (scale bar: 100 µm). **b,** Confocal microscopy imaging of STV-C8(EGFP) treated and immunostained spheroid slices (scale bar: 100 µm). **c,** RT-qPCR characterization of the lung-specific AT2 marker gene and ACE2 in iAT2 differentiated cells (mean ± s.d. for n = 3 biological replicates).

a

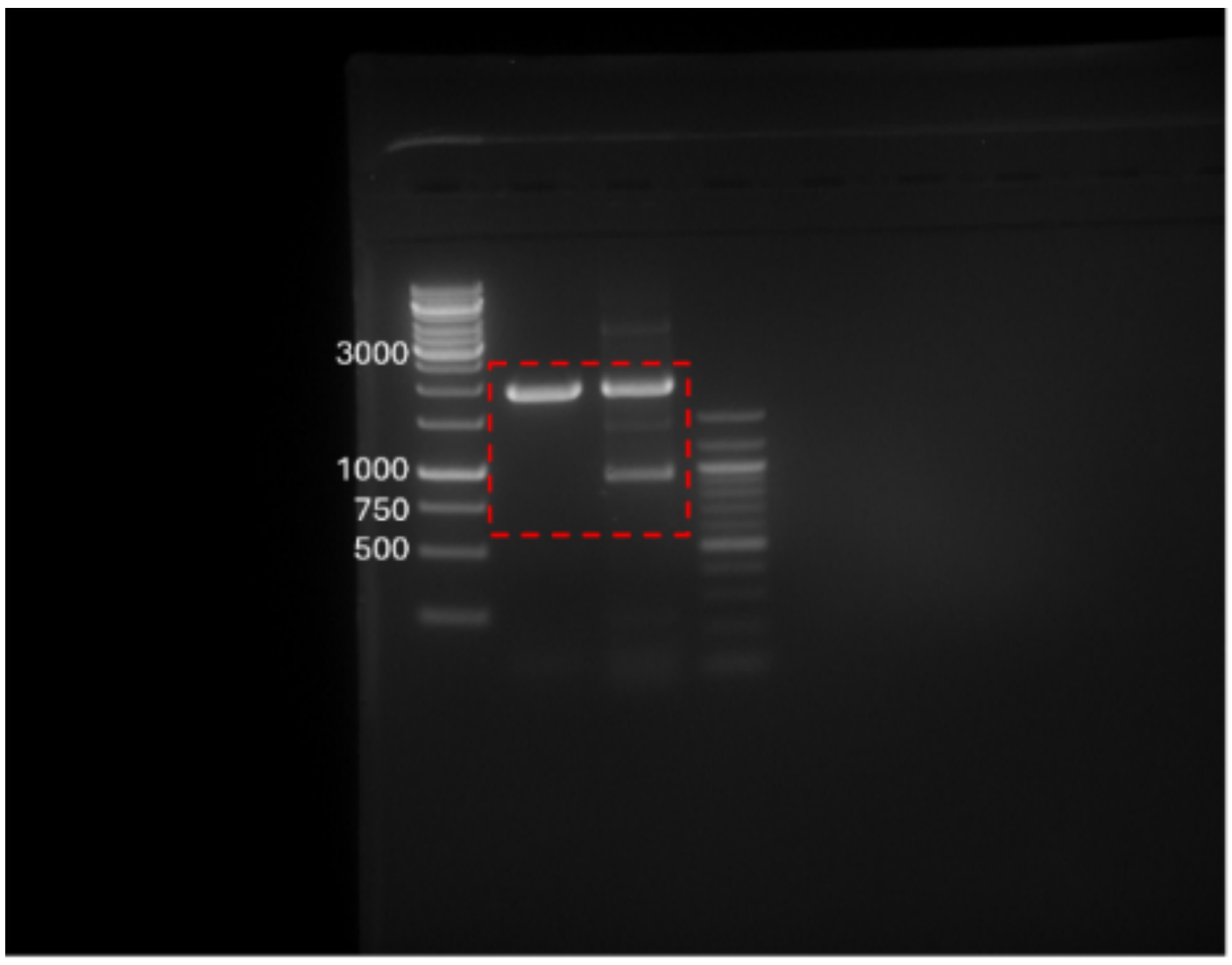

uncropped

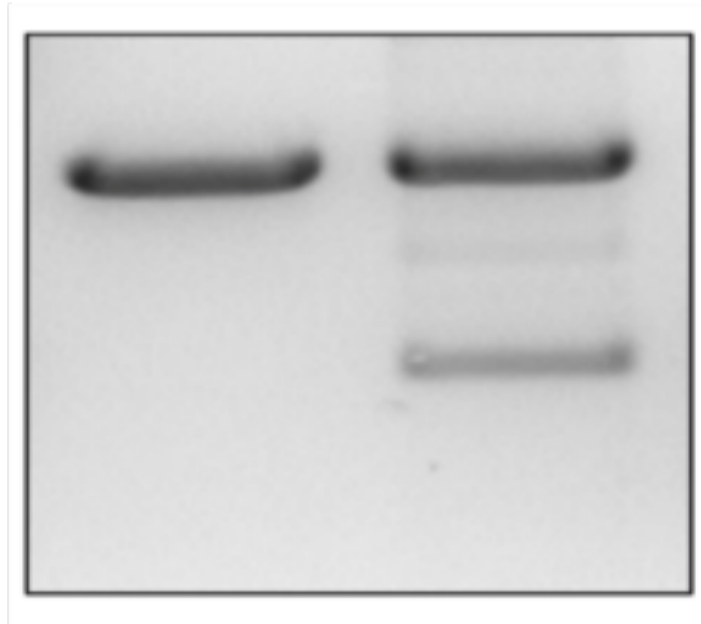

cropped

b

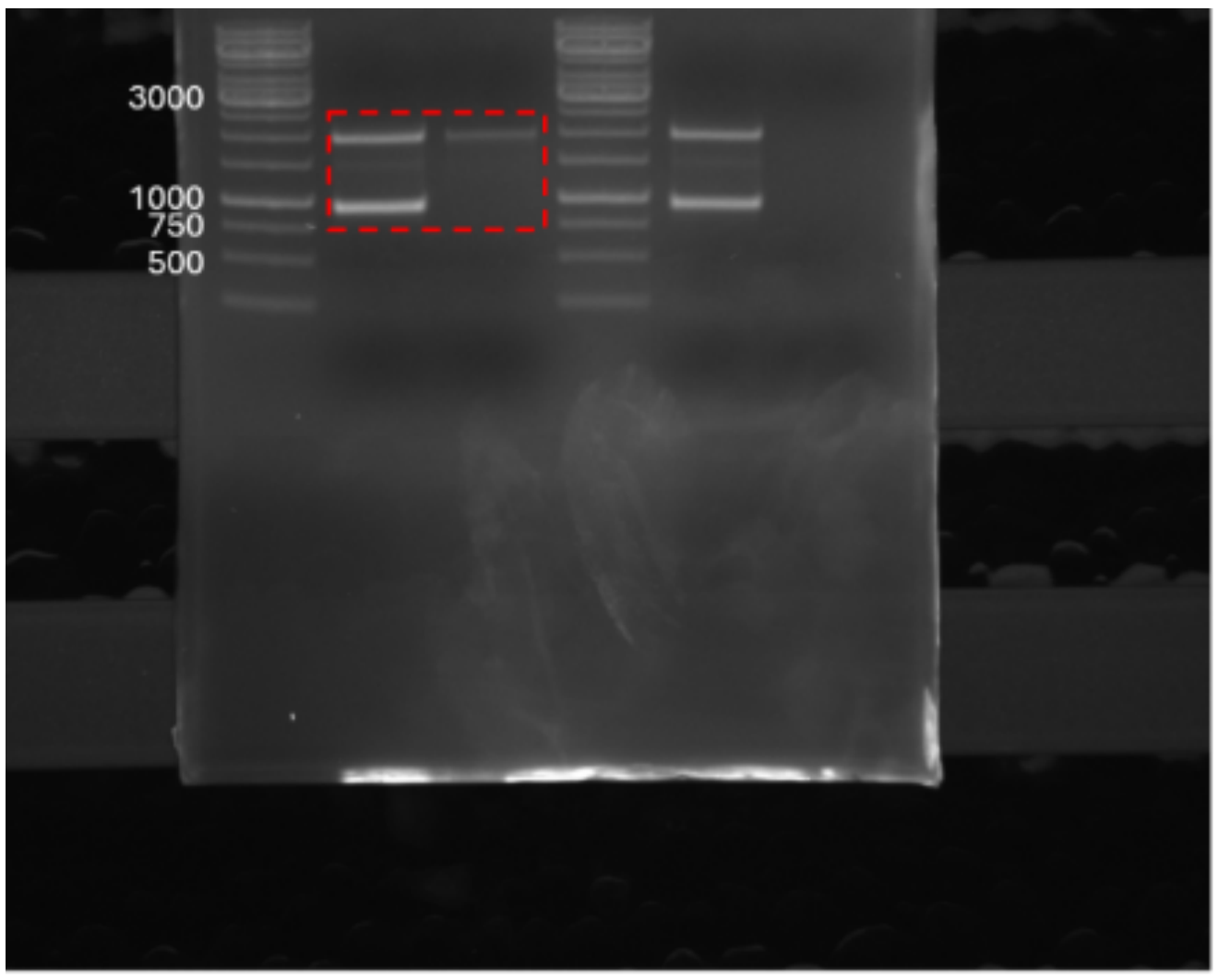

uncropped

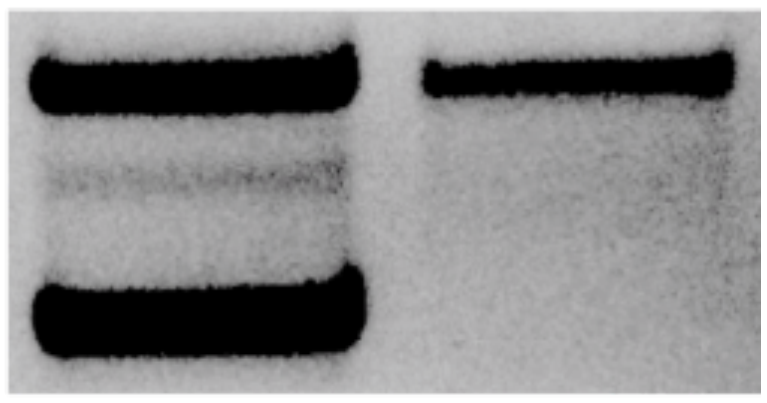

cropped

c

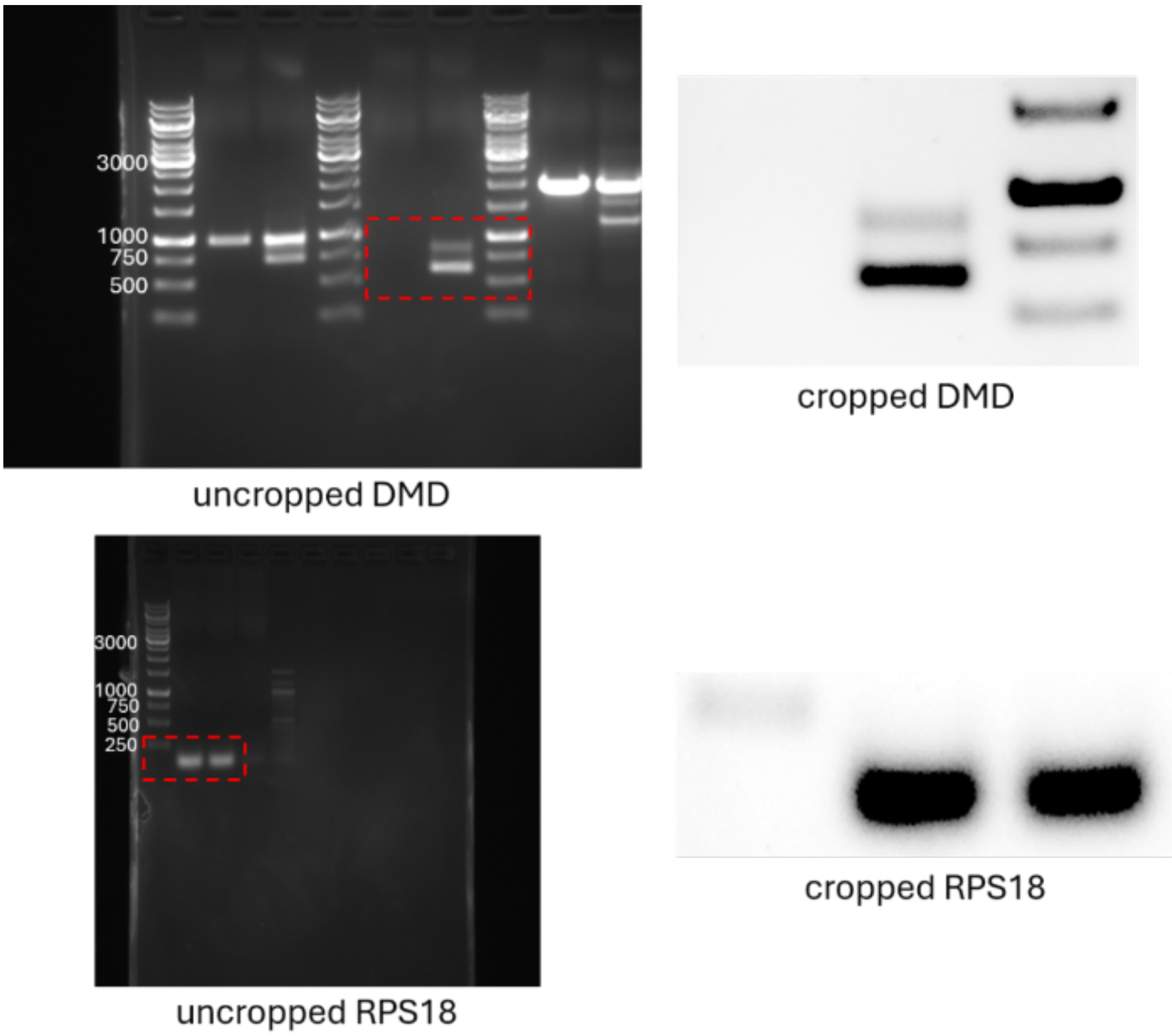

Supplementary Fig. 7 | Uncropped Agarose gels. a, Agarose gel corresponding to Figure 3h. **b,** Full-sized agarose gel of Figure 4e. **c,** Uncropped agarose gel of Extended Data Fig. 10h.

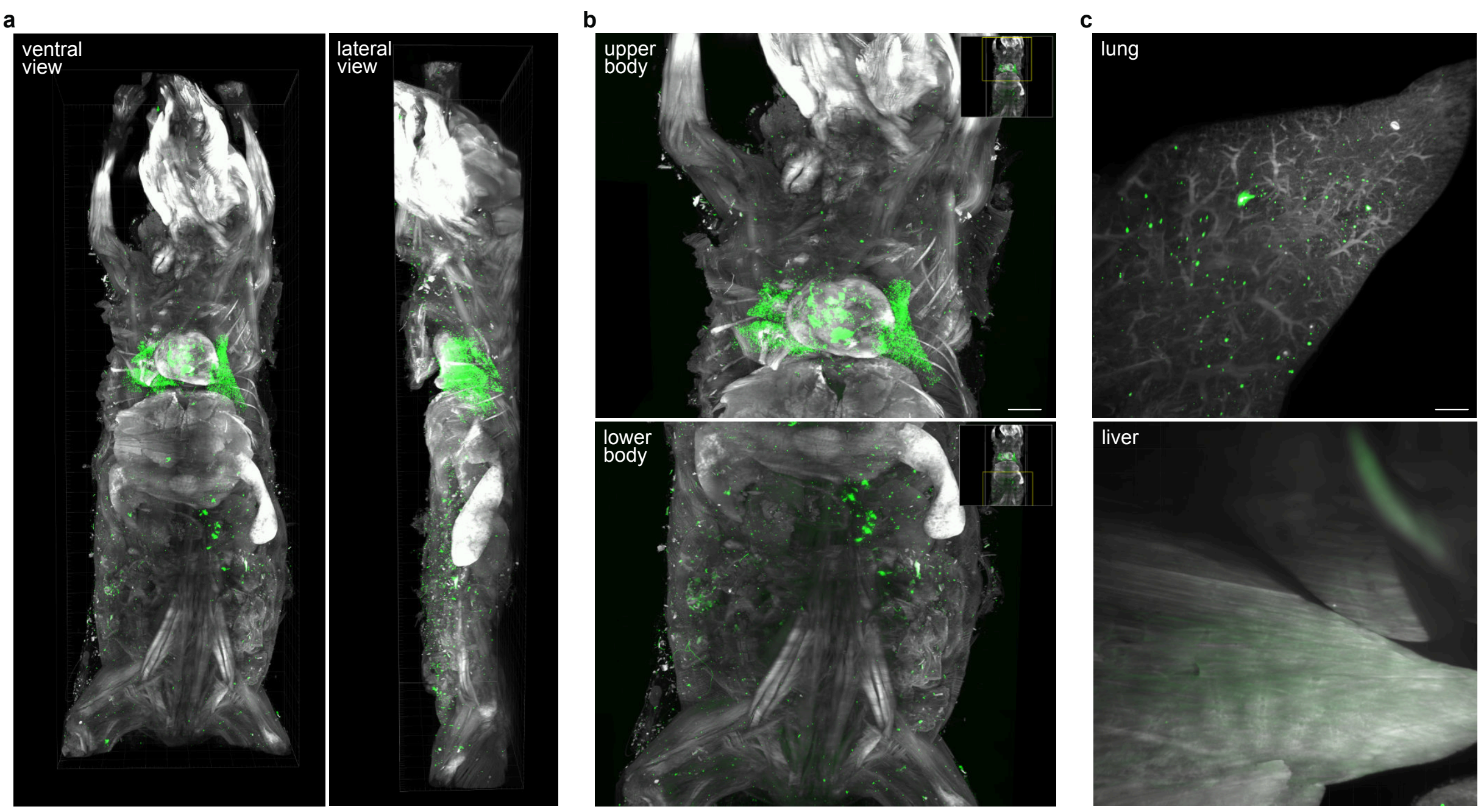

Supplementary Fig. 8 | Additional analysis of STV-C8 whole-body tissue distribution. a, Light-sheet imaging of cleared and signal-enhanced mouse body, treated with STV-C8(EGFP), 72 h post i.v. injection. **b,** Magnified upper and lower body parts of panel a (scale bar: 2 mm). **c,** Imaging of lung and liver tissues of an independently treated mouse, 24 h post i.v. injection (scale bar: 200 µm). EGFP is shown in green, tissue autofluorescence in grayscale.

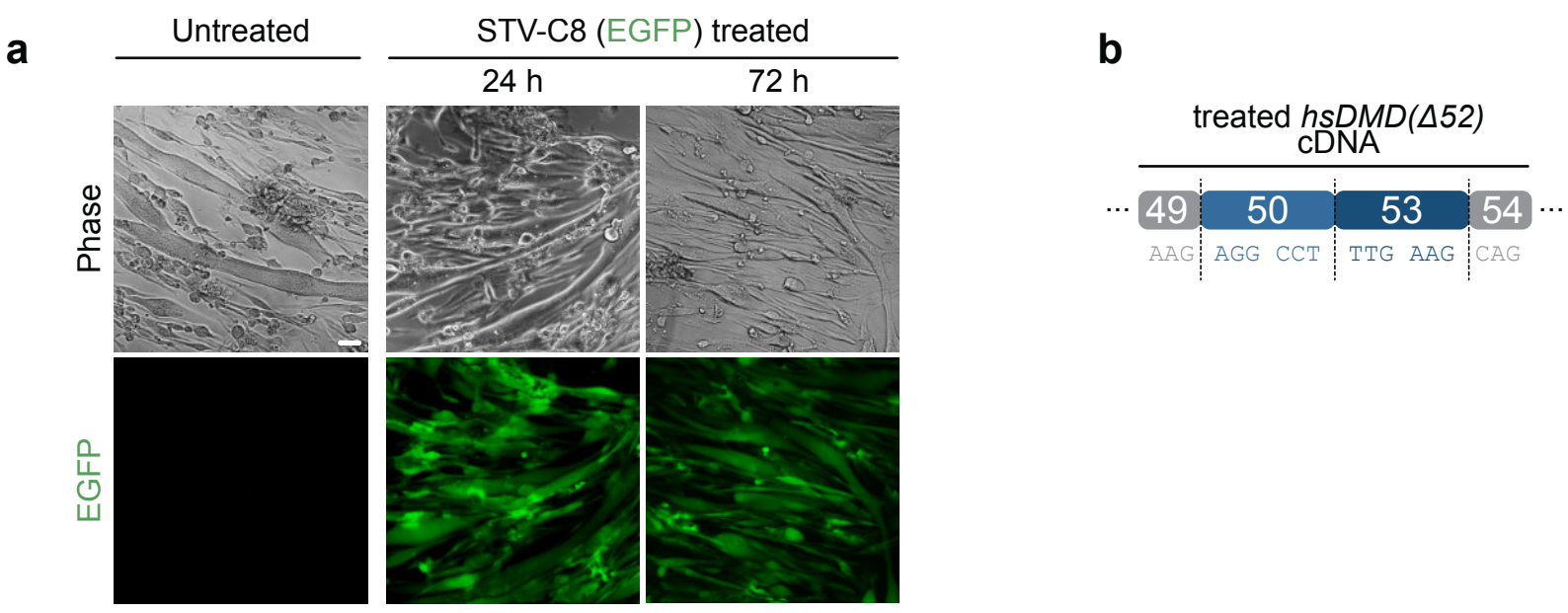

Supplementary Fig. 9 | Extended analysis of STV-C8 treated myotubes. a, Live imaging of STV-C8(EGFP) treated myotubes (scale bar: 25 µm). **b,** Exon-exon junction analysis of cDNA derived from edited patient-derived myotubes by nanopore sequencing.

Exemplary gating strategy

Samples were first gated for living cells and subsequently for the fluorescence intensity, relevant for the experiment.

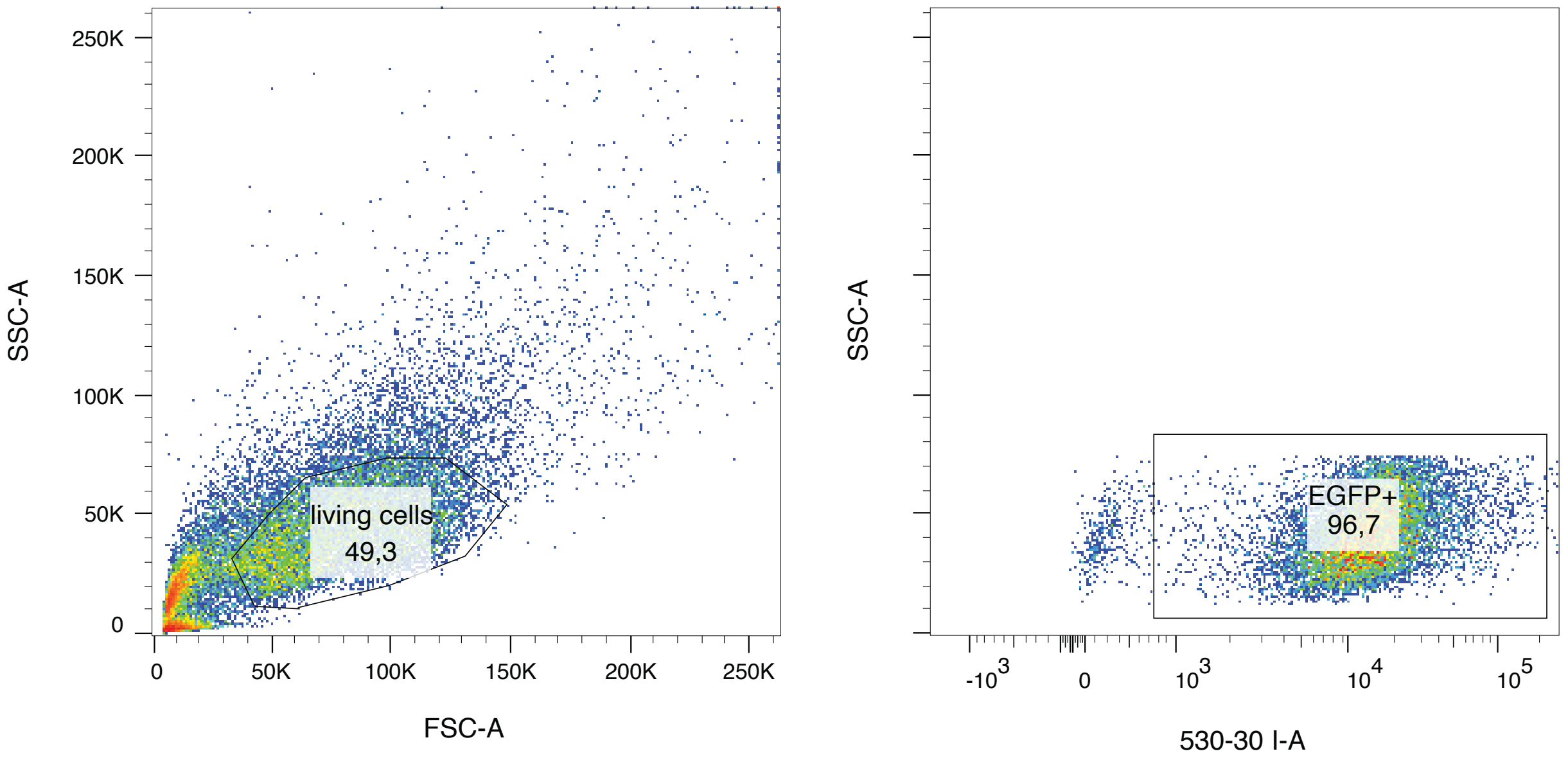

Supplementary Table 1 | Protein, RNA, and DNA sequences used in this study

Name	Description	Sequence
EGFR Receptor	EGFR Receptor for Minibinder Targeting Experiments	MRPSGTAGAALLALLAALCPASRALEEKKVCQGTSNKLTQLGTFEDHF<br>LSLQRMFNNCEVVLGNLEITYVQRNYDLSFLKTIQEVAGYVLIALNTVE<br>RIPLENLQIIRGNMYYENSYALAVLSNYDANKTGLKELPMRNLQEILHG<br>AVRFSNNPALCNVESIQWRDIVSSDFLSNMSMDFQNHLGSCQKCDP<br>SCPNGSCWGAGEENCQKLTKIICAQQCSGRCRGKSPSDCCHNQCAA<br>GCTGPRESDCLVCRKFRDEATCKDTCPPLMLYNPTTYQMDVNPEGKY<br>SFGATCVKKCPRNYVVTDHGSCVRACGADSYEMEEDGVRKCKKCEGP<br>CRKVCNGIGIGEFKDSLSINATNIKHFKNCTSISGDLHILPVAFRGDSFTH<br>TPPLDPQELDILKTVKEITGFLLIQAWPENRTDLHAFENLEIIRGRTKQH<br>GQFSLAVVSLNITSLGLRSLKEISDGDVIISGNKNLCYANTINWKKLFGT<br>SGQKTKIISNRGENSCKATGQVCHALCSPEGCWGPEPRDCVSCRNVS<br>RGRECVDKCNLLEGEPREFVENSECIQCHPECLPQAMNITCTGRGPDN<br>CIQCAHYIDGPHCVKTCPAGVMGENNTLVWKYADAGHVCHLCHPNC<br>TYGCTGPGLEGCPTNGPKIPSIATGMVGALLLLLVVALGIGLFMRRRHI<br>VRKRTLRRLLQERELVEPLTPSGEAPNQALLRILKETEFKKIKVLGSGAFG<br>TVYKGLWIPEGEKVKIPVAIKELREATSPKANKEILDEAYVMASVDNPH<br>VCRLLGICLTSTVQLITQLMPFGCLLDYVREHKDNIGSQYLLNWCVQIA<br>KGMNYLEDRRLVHRDLAARNVLVKTPQHVKITDFGLAKLLGAEEKEYH<br>AEGGKVPIKWMALESILHRIYTHQSDVWSYGVTVWELMTFGSKPYD<br>GIPASEISSILEKGERLPQPPICTIDVYMIMVKCWMIDADSRPKFRELIIE<br>FSKMARDPQRYLVIQGDERMHLPSPTDSNFYRALMDEEDMDDVVD<br>ADEYLIPQQGFFSSPSTSRTPLLSSLSATSNNSTVACIDRNGLQSCPIKED<br>SFLQRYSSDPTGALTEDSIDDTFLPVPEYINQSVPKRPAGSVQNPVYHN<br>QPLNPAPSRDPHYQDPHSTAVGNPEYLNTVQPTCVNSTFDSPAHWA<br>QKGSHQISLDNPDYQQDFFPKEAKPNGIFKGSTAENAEYLRVAPQSSE<br>FIGA
IL7Ra Receptor	IL7Ra Receptor for Minibinder Targeting Experiments	MTILGTTFGMVFSLLQVVSGESGYAQNGDLEDAELDDYSFSCYSQLEV<br>NGSQHSLTCAFEDPDVNITNLEFEICGALVEVKCLNFRKLQEIYFIETKKF<br>LLIGKSNICVKVGEKSLTCKKIDLTTIVKPEAPFDLSVVYREGANDFVVTF<br>NTSHLQKKYVKVLMHDVAYRQEKDENKWTHVNLSSTKLTLLQRKLQP<br>AAMYEIKVRSIPDHYFKGFWSEWSPSYYFRTPEINNSSGEMDPILLTISI<br>LSFFSVALLVILACVLWKKRIKPIVWPSLPDHKKTLEHLCKKPRKNLNVS<br>FNPESFLDCQIHRVDDIQARDEVEGFLQDTFPQQLEESEKQRLGGDVQ<br>SPNCPSEDVVITPESFGRDSSLTCLAGNVSACDAPILSSSRSLDCRESGK<br>NGPHVYQDLLLSLGTTNSTLPPPFSLQSGILTLNPVAQGQPILTSLGSN<br>QEEAYVTMSSFYQNQ
CD19 Receptor		MPPPRLLFFLLFLTPMEVRPEEPLVVKVEEGDNAVLQCLKGTSDGPTQ<br>QLTWSRESPLKPFLKLSLGLPGLGIHMRPLAIWLFIFNVSQQMGGFYLC<br>QPGPPSEKAWQPGWTVNVEGSGELFRWNVSDLGGLGCGLKNRSSE<br>GPSSPSGKLMSPKLYVWAKDRPEIWEGEPPCLPPRDSLNQSLSQDLT<br>MAPGSTLWLSCGVPPDSVSRGPLSWTHVHPKGPKSLLSLELKDDRPA<br>RDMWVMETGLLLPRATAQDAGKYYCHRGNLTMSFHLEITARPVLW<br>HWLLRTGGWKVSAVTLAYLIFCLCSLVGILHLQRALVLRRKRKRMTDP<br>TRRFFKVTPPPGSGPQNQYGNVLSLPTPTSGLGRAQRWAAGLGGTAP<br>SYGNPSSDVQADGALGSRSPPGVGPEEEEGEGYEEPDSEEDSEFYEND<br>SNLGQDQLSQDGSGYENPEDEPLGPEDEDSFSNAESYENEDEELTQP<br>VARTMDFLSPHGSAWDPSREATSLGSQSYEDMRGILYAAPQLRSIRG<br>QPGPNHEEDADSYENMDNPDGPDPAWGGGGRMGTWSTR

EGFRn Minibinder	EGFRn Minibinder Sequence deisgned by Cao et al	DHWEEVFRWALEHLQEATQQNDPQKAKKILEEAHKWLRRELSEEEA RAVVRWLKQLVDRELS
EGFRc Minibinder	EGFRc Minibinder Sequence deisgned by Cao et al	SLDEAKKLLQEAEKLARKLNDRMELAYVEFLKHILETAKKQNDKRTIESV RDMARDALEELQS
IL7Ra Minibinder	IL7Ra Minibinder Sequence deisgned by Cao et al	SVIEKLRKLEKQARKQGDEVLVMLARMVLEYLEKGWVSEEDADESAD RIEEVLKK
CD19scFv	Light chain (GGGGS)$_3$ Heavy chain	DIQMTQTTSSLSASLGDRVTISCRASQDISKYLNWYQQKPDGTVKLLIY HTSRLHSGVPSRFSGSGSGTDYSLTISNLEQEDIATYFCQQGNTLPYTF GGGTKLEITGGGGSGGGGSGGGGSEVKLQESGPGLVAPSQSLSVTCT VSGVSLPDYGVSWIRQPPRKGLEWLGVIWGSETTYYNSALKSRLTIIKD NSKSQVFLKMNSLQTDDTAIYYCAKHYYYGGSYAMDYWGQGTSVTV SS
EGFRn-STV-Presentation	EGFRn Minibinder Presentation (SP, export signals, etc.) - **Minibinder Sequence**	MGVKVLFALICIAVAEAKGSGKPIPNPLLGLDSTPGWEAALAEALAEAL AEHLAEALAEALEALAAGPGSEPATSGSETPGTSESATPESGPGGSG**D HWEEVFRWALEHLQEATQQNDPQKAKKILEEAHKWLRRELSEEEAR AVVRWLKQLVDRELS**TGGPGSEPATSGSETPGTSESATPESGPGSGA HHFSEPEITLIIFGVMALVIGTILLISYGIRRLIKKSPSGGGTGFCYENEVG SGRSRFVKKDGHCNVQFINVGSGKSRITSEGEYIPLDQIDINVGGSGGD YKDDDDK
EGFRc-STV-Presentation	EGFRc Minibinder Presentation (SP, export signals, etc.) - **Minibinder Sequence**	MGVKVLFALICIAVAEAKGSGKPIPNPLLGLDSTPGWEAALAEALAEAL AEHLAEALAEALEALAAGPGSEPATSGSETPGTSESATPESGPGGSG**SL DEAKKLLQEAEKLARKLNDRMELAYVEFLKHILETAKKQNDKRTIESV RDMARDALEELQS**TGGPGSEPATSGSETPGTSESATPESGPGSGAHH FSEPEITLIIFGVMALVIGTILLISYGIRRLIKKSPSGGGTGFCYENEVGSGR SRFVKKDGHCNVQFINVGSGKSRITSEGEYIPLDQIDINVGGSGGDYKD DDDK
IL7Ra-STV-Presentation	IL7Ra Minibinder Presentation (SP, export signals, etc.) - **Minibinder Sequence**	MGVKVLFALICIAVAEAKGSGKPIPNPLLGLDSTPGWEAALAEALAEAL AEHLAEALAEALEALAAGPGSEPATSGSETPGTSESATPESGPGGSG**SV IEKLRKLEKQARKQGDEVLVMLARMVLEYLEKGWVSEEDADESADR IEEVLKK**TGGPGSEPATSGSETPGTSESATPESGPGSGAHHFSEPEITLII FGVMALVIGTILLISYGIRRLIKKSPSGGGTGFCYENEVGSGRSRFVKKD GHCNVQFINVGSGKSRITSEGEYIPLDQIDINVGGSGGDYKDDDDK
HE0482	cage screen/cage sequence	DTKEKAETFAKIVAELVKQGFLETAAAFTREVLAELPEEDKLEVALAMT KAFLEELEKKE
HE0485	cage screen/cage sequence	EEEKQKELYKTIVESAKEISEKYGIDETVAQQQLLQAVALLSLLDEETIKR LTEEVLKMK
HE0489	cage screen/cage sequence	WEEKARRFLRIELIKELLHLYVLARVLGLEETAKLLAKAIKTLIEEDPESEK VLEEVKSLLNETGREILERLLEEVEKEE
HE0490	cage screen/cage sequence	LKKRVVRYVATRLFTEILSLEPLIGRETALELLLETARILYKASGELELILEVA REEMRRAGVPEEDIEALLAELRAWA
HE0492	cage screen/cage	EERLEKLNKLETVKEYLKKAVYLGTGDEVLEELLKYVPEEYKELILKVREA

	sequence	AELEKALL
HE0497	cage screen/cage sequence	SEEIAKDLRLMAEISHDNLATLAIILIREGEDIDFLLEIAEDLSELSRLTMRYVAEKLKA
HE0499	cage screen/cage sequence	KLLEVAVLKAIAELLGLAILDPRAIPLAREALEKLRKIHPSEIIKEMCDMGERILELIEE
HE0501	cage screen/cage sequence	KMAEMLVEHVILELERYEERGNAAAMIYTFIERLGKLALLGPEALRLAAAEFAKLPSPLAPELAAACETAAALLERLLAA
HE0502	cage screen/cage sequence	KMAEMLVEHVILELEQYEARGNEAAMIYTFIEHLGKLALLGPEALELAAARLAKLPSPLAPELAAAAEEAAALLKRLLAA
HE0505	cage screen/cage sequence	DLLKEAEELVKKILETDPEANPAALNLYTILKTYVDIGAEKQAKKILELLKVVAEHLEKK
HE0513	cage screen/cage sequence	DAEALVERAAELLLERVRAGVDLEKALTEVLLAVLVRLTEEDAERFVELVVARFAETAELVRTLCEAALLKAKALVEALD
HE0517	cage screen/cage sequence	ERRERARLMMELLAEIAEEAAILQLAAQLAVLMYLSSLVGEDPEELLEIFEEIVLARVQTEFQREVAERVLERLREFVEK
HE0521	cage screen/cage sequence	AEEVLALVAARVMRQLAEDALLAAELGLEEVAAALVELMKALAEEVAKRVSPEVAERVKRVLALLLTRLEKEIAEAKAAE
HE0537	cage screen/cage sequence	SSFQEEAEKTFENLLELLLIATNLDTRVASELAEALVYIMGLLEENKEEFFEKFLKLSKYLNNVTLARAIKTAIELGDKEFTEKLLKEFNKIIEEKKKLV
HE0620	cage screen/cage sequence	KKIVIEAEMIPENHETILAVVKELVEEGVENLEIVLKVTPETTKEQSVELVKKIIEILK
HE0621	cage screen/cage sequence	AAAEIIKLNLTITENAKFQLLQDYAKFLGDKEELKKITEEANKWFEERMKELEERIKKLE
HE0625	cage screen/cage sequence	VKRKFTLKLTTGASDDMTEVVPEFLAMIKEAAELFDEVTIKVTTESPEMARAVMEGVGILIKEGVDVTLEIELGSNVKARVEVLKTLAEELKKIKEEIE
HE0626	cage screen/cage sequence	IKRHFTLKLTTGASDDMKEVVPEFLAMIKEAAELFDEVTLKLTTENPEMARALMEGVGILIKEGVDVHLEVELGSNVKARVEVLKTLAEELKKIKEEIE
HE0633	cage screen/cage sequence	ATTTTTTVTLDSASGERTLTTTTTVPAGTTLAEMVALAEAQLAAASEFDRRTATVTVTEE
HE0639	cage screen/cage sequence	SKAEELAKEMIELLRLVKELLLETTPEFPETTLIALAIVLNAVVTTLNALLDKVPLELLKELVEVMKDTVEGLKEKLKAL
HE0644	cage screen/cage sequence	VSISLSGKTSEELKVMAKLAEYLGEEKTAEILKKLIPEVKELEAKGETSLEVSVTITFE
HE0649	cage screen/cage sequence	KKSVTIRVTADAAVLAEFTALLLEWLDLDAREVLEAFRKWLEEMKARGIKDDYTVELTVE
HE0657	cage screen/cage sequence	EELIKELKEKQKETQAELEKEYKEYKEKGESESAAIMKATSTQVATKYEIEIAKLKLELL
HE0662	cage screen/cage sequence	EAILEAIRRLCEAGIKVTVVLAGPISLMEAIVETCIEVGVDEVVIDTSKDHLELIEADK
HE0690	cage screen/cage sequence	LKVKIKVKDNPAVARGVLRLADKLKKAGVDVEIEIDLYGDEEQALATLAAMEAEVEELA
HE0897	cage screen/cage sequence	SAEALKLARLAALVGLVGAAVATADPANPLFLELLKNPDPFVLQEALTAALVAAILTGEAARCNALALGLAARLSREDAARAALRAAELAYETAALLASL
HE0898	cage screen/cage sequence	SFTEEELEKLKEAVKLAVEALLLTPKDFEKALKLLEEVTLKVVEILRRDPLEALKAAFKSTTAIAKLYVAHASKDVSEAQAIAAEAVKALLDLYEKALKE
HE0899	cage screen/cage sequence	SFTEEELEELKKAVKLAVEALLLTPDDFEKALKLLEEVTLRVVEILKRDPLEALKAAFYFNTQIAKLYVAHASGDVSEAQKEMAKFVKYLLDLYEKALEE
HE0900	cage screen/cage sequence	AFTEEQLKALQESLKLVIEAAELMPDDFEKAIELVEEVARRLVEIFASDPLAALHLAFKFNTAIAKAIVANASKGKEEAMKVLVELAKYVFDLLVAALEA

HE0902	cage screen/cage sequence	FTEEEIKKIRESLKLSVEALEVTPKDFEKALELLEEVAINLMEIFKDDPMKALKIAFKFTNAIAKLYVAHESKDVADAMAIMAEVTKYILEILEKVLEE
HE0908	cage screen/cage sequence	ELAEKERLARINIGAVLAGLAMAVVGACKELAEAGMVMAIMSVVAVELAQDPEAAVELAERCIEMAGGLSEEVKKMIKAAVRALKGVVEAEEAKIKKFKA
HE0915	cage screen/cage sequence	EEELLEEILEFFEKLSDPKALVAFMLEKAPPDPGLKTALRIALSGPNITVGKALTLGKAIAEVSAGRYGGAGMIAGAAGGFEIGDEEVKRECKLALLKYL
HE0916	cage screen/cage sequence	SKEVEEALLKALAAALIAAVAANKAAAVEVLRAIAEEGLDVLVAVVREAMRALADSPLVATLREAAREVAAELPPEERARFLAALEAAAAEVEAEAARAR
HE0917	cage screen/cage sequence	SAEVEQALLEALAAALIGAVAASREAAKAVLRAIAELGLDVALAVVRRALRALAESPLVEVLLEAAREVAEELPPEERARFLAALARAAAEVEEEERERR
HE0918	cage screen/cage sequence	EEAVKKALIEALAAALIAAVAASKEAAKAVLKAIAELGLDVAVEVVEKALRALADSPLVAVLIEAAKEVAEELPPEERARFLAALAEAVARVEAEAKEAA
HE0919	cage screen/cage sequence	EAVQQALLEALAAALIAAAAASAAAAKAVLRAIAELGLDVLVEVVRRALRALADSPLVATLLAAAREVAAELPPEERAEFLAALERAAAEVEEEAARAK
HE0920	cage screen/cage sequence	SEEVRQALLEALAAALIAAAAASREAAVAVLRYLVELGLEVALEVVRLALRALRGSPLRERLLAAAEEVAAELPGEEAARFRAALARAAAEVAEEAAREE
HE0930	cage screen/cage sequence	VKELVAELAELAGEADAELRAQIALLLAVIALAPEEQKEELAELALRVVREALEAAASPAVPVALAAAAAAFARSEDPEAVRREAEAIITGLRLAKEGLA
HE0935	cage screen/cage sequence	KELLAEIAELAGEADAELRAQAALLLAVIALAPEEDKEFYAELALRVLRAGLEAAASPAVPVALAKAAAALARGEDREAVERTIEAILTGLELTKKGLA
no cage	cage screen/cage sequence	GGS
F5GXC1	MBD screen/MBD sequence	LRGSRLRKIRSRTWHKERLYRLQEDGLSVWFQRRIPRAPSQHIFFVQHIEAVREGHQSEGLRRFGGAFAPARCLTIAFKGRRKNLDLAAPTAEEAQRWVRGLTKLR
Q9BRC7	MBD screen/MBD sequence	LTTDQDLLLMQEGMPMRKVRSKSWKKLRYFRLQNDGMTVWHARQARGSAKPSFSISDVETIRNGHDSELLRSLAEELPLEQGFTIVFHGRRSNLDLMANSVEEAQIWMRGLQLLV
O75038	MBD screen/MBD sequence	CMGAMQEGMQMVKLRGGSKGLVRFYYLDEHRSCIRWRPSRKNEKAKISIDSIQEVSEGRQSEVFQRYPDGSFDPNCCFSIYHGSHRESLDLVSTSSEVARTWVTGLRYLM
Q15111	MBD screen/MBD sequence	SANDCISFMQAGCELKKVRPNSRIYNRFFTLDTDLQALRWEPSKKDLEKAKLDISAIKEIRLGKNTETFRNNGLADQICEDCAFSILHGENYESLDLVANSADVANIWVSGLRYLV
Q96BJ8	MBD screen/MBD sequence	RLLRLCEGTLFRKISSRRRQDKLWFCCLSPNHKLLQYGDMEEGASPPTLESLPEQLPVADMRALLTGKDCPHVREKGSGKQNKDLYELAFSISYDRGEEEAYLNFIAPSKREFYLWTDGLSALL
Q96JJ3	MBD screen/MBD sequence	RLNRLCEGSSFRKIGNRRRQERFWYCRLALNHKVLHYGDLDDNPQGEVTFESLQEKIPVADIKAIVTGKDCPHMKEKSALKQNKEVLELAFSILYDPDETLNFIAPNKYEYCIWIDGLSALL
B4DN84	MBD screen/MBD sequence	ASLLQDLSISDVETIRNGHDSELLRSLAEELPLEQGFTIVFHGRRSNLDLMANSVEEAQIWMRGLQLLV
Q9HB19	MBD	LIKSGYCVKQGNVRKSWKRRFFALDDFTICYFKCEQDREPLRTIFLKDVL

	screen/MBD sequence	KTHECLVKSGDLLMRDNLFEIITSSRTFYVQADSPEDMHSWIKEIGAAV
Q9HB21	MBD screen/MBD sequence	KPPQDSAVIKAGYCVKQGAVMKNWKRRYFQLDENTIGYFKSELEKEPLRVIPLKEVHKVQECKQSDIMMRDNLFEIVTTSRTFYVQADSPEEMHSWIKAVSGAI
Q9Y4G2	MBD screen/MBD sequence	EPDAIKESLLYLYMDRTWMPYIFSLSLEALKCFRIRNNEKMLSDSHGVETIRDILPDTSLGGPSFFKIITAKAVLKLQAGNAEEAALWRDLVRKVL
A0A2U3YBU1	MBD screen/MBD sequence	GLQDDEDLQALLKGSQLLKVKSNSWRRERFYKLQEDCKTIWQESRKVMRTPESQLFSIEDIQEVRKGHRTEGMEKFARDVPEDRCFSIVFKDQRNTLDLIAPSPAEAQHWVQGLRKIIH
A0A061I7U7	MBD screen/MBD sequence	GLQDDQDLQALLKGSQLLKVKSSSWRRERFYKLQEDCKTIWQESRKVMRSPESQLFSIEDIQEVRMGHRTEGLEKFARDIPEDRCFSIVFKDQRNTLDLIAPSSADAQHWVQGLRKIIH
A0A7N9CX73	MBD screen/MBD sequence	DDEDLQALLKGSQLLKVKSSSWRRERFYKLQEDCKTIWQESRKVMRTPESQLFSIEDIQEVRMGHRTEGLEKFARDVPEDRCFSIVFKDQRNTLDLIAPSPADAQHWVLGLRKIIH
A0A480MS63	MBD screen/MBD sequence	GLQDDKDLQALLKGGQLLKVKSNSWRRERFYKLQEDCKTIWQESRKVMRTPESQLFSIEDIQEVRMGHRTEGLEKFARDVPEDRCFSIVFKDQRNTLDLIAPSPADAQHWVLGLRKIIH
A0A452QKQ4	MBD screen/MBD sequence	GLQDDEDLQVLLKGSQLLKVKSNSWRRERFYKLQEDCKTIWQESRKVMRTPESHLFSIEDIQEVRKGHRTEGMEKFARDVPEDRCFSIVFKDQRNTLDLISPSPAEAQHWVRGLHKIIH
A0A1S3GCG9	MBD screen/MBD sequence	GLQDDRDLQALLKGGQLLKVKSGSWRRERFYKLQEDCKTIWQESRKVMRTPESQLFSIEDIQEVRMGHRTEGLEKFARDIPDDRCFSIVFKDQRSTLDLIAPSPADAQHWVQGLRKVIH
A0A6P5IN86	MBD screen/MBD sequence	GPADDGDLKFLLKGSQLLKVKSSSWRKDRFYKLQEDCKTIWQESKKMMRSPESQLFSIEDIQEVRRGHQTEGMEKFARDAPEHRCFSIIFKDQRSNLDLIAGSEVEAQHWVDGLRKII
A0A2I0TQP5	MBD screen/MBD sequence	GLKDDKDLKFLLKGSQLLKVKSGSWRKERFYKLQEDCKTIWQESKKMLRSPESQIFSIEDIRDVRSGHKTEGMEKYAKDVPEYRCFSIIFKDQRKTLDLIASSEDDANHWISGLGKII
A0A093CX92	MBD screen/MBD sequence	KDDKDLKFLLKGSQLLKVKSGSWRKERFYKLQEDCKTIWQESKKMLRSPESQIFSIEDIRDVRSGHKTEGMEKYAKDVPEYRCFSIIFKDQRKNLDLIASSEDDANHWISGLGKII
A0A6P5IML8	MBD screen/MBD sequence	GPADDGDLKFLLKGSQLLKVKSSSWRKDRFYKLQEDCKTIWQESKKMMRSPESQLFSIEDIQEVRRGHQTEGMEKFARDAPEHRCFSIIFKDQRSNLDLIAGSEVEAQHWVDGLRKII
MGYP002377957044	MBD screen/MBD sequence	QEGSDFLKVRSYARQFRRLYKLNESLTMISWYPTSKKPSKATSRNVLRRKKNEIISICLFFRLVPIDSIKEVRLGKTTERLREYAQQYENETLFSIIYTNGSNEYDSLDLVASTADEANIWVTGLSCLI
MGYP001103578616	MBD screen/MBD sequence	FMKKGTPFLKYGHYGYPHFRQFQLSEDNARIQWYSQAKSLKDTTISIYDIDEIRLGQTTAIFAKHRAPELSKSSFSVIYTGGKTLDVIAKDPNEYRLWTEGLRRLV
MGYP002782181533	MBD screen/MBD sequence	GSDFLKVRSYARQFRRFYRLNETLTSISWYPTSKKAAKAMSKNASSNREMEMIYLSTSVLIDSIKEVRLGKTSERLRECTHQFQNESLFSIIYSNGTHDNVSLDLVTSSPDEANIWVTGLSCLI
MGYP002169641634	MBD screen/MBD sequence	INLVTEMQTGSVMLKCAKHGKPHFSLFQLSSDAEMLQWTSRKKAPAETQLRIDHIRELLLGQQTPVFARCSLPQFEGLSFSVITPERSLDIVCTTAAELRTWTRGLEAL
MGYP003572965	MBD	PVAETLTELKGGHRLIKCRGSKSKYTRHFRITEDLIAMVYTDSHKPDCVA

178	screen/MBD sequence	GKDDMSNVYITEIHEIRDTPSSDVFKKQLPSLSGKEQQCFSLVVGERHKTLDLVAPSPKVKDSWVIGLNHLK
MGYP001233249524	MBD screen/MBD sequence	YDGTMLLKFGRKGQAHERLFRLSQDNRYISWKSSIFTFKSAEDCRVDLEAVSRIQHGQMTANFERHKAHFGRAESNSFSIIYNGDRTLDLIAPSYDIFEIWFHGLKYLL
MGYP003385211623	MBD screen/MBD sequence	TIEFMIRGENVNKLPHQIHKKKITKKPQQRFLRLTSDLKNIFWRSSTANSVFSSATRSKATERGIYIRDVLDVQVGQTTETFRLQQMEMPVASSFSLITAERSVDIVCESSYHRDLWVAGMRLLM
MGYP003537806627	MBD screen/MBD sequence	AQKYLPMLYQGERFLKYGNYTTPHERFFKLSPDHKKITWSTISGCRTFHRNKCIFIDSIKDIYIGVDSSRVFQQFRIPVDYDQNCFSIVTTSRSLDLRNENEAISMKWFSAIKYLI
MGYP001097820974	MBD screen/MBD sequence	TIGQLERGTVVTKFYSKKKPEKKMLAVRRETMLVVWHRALSGRNMFEGAVDIRDIKEIRLGKLSRDFEKWSDDCQNIKQGRCFVVYYGREFNLKTLSVVALAESECDLWIRGLRFLV
MGYP003957870823	MBD screen/MBD sequence	NLLKQGAIFRKHNLHGKPVERFITLSQDLQYLQWFPVEDHTKMSGKVSVSDIFGVRTGLSSAALKRSGLTGAKIKLCFTVYCSNDVLLSLEAASPEQCEEWVSSLRYLIS
noMBD	MBD screen/MBD sequence	G
noBudding	no budding domain	TG
SynL	budding domain	PTAPPGSGSPTAPPEYSGSGPSAPPMEEKLLDLGGSGPYKEGSGSPPPPYVGSGLYPSLSGGFPIVSGGPLPPVGSGSYLDL
LS4	Alternative RNA binding protein	YVRFEVPEDMQNEALSLLEKVRESGKVKKGTNRTTHAVYRGLAKLVYIAEDVDPPEIVAHLPLLCEEKNVPYIYVKSKNDLGRAVGGWPIGASAAIINEGELRKELGSLVEKIKGLQKRSHMHLE
PCP	Alternative RNA binding protein	SKTIVLSVGEATRTLTEIQSTADRQIFEEKVGPLVGRLRLTASLRQNGAKTAYRVNLKLDQADVVDSGLPKVRYTQVWSHDVTIVANSTEASRKSLYDLTKSLVATSQVEDLVVNLVPLGR
tdPCP	Mainly used RNA binding protein	SKTIVLSVGEATRTLTEIQSTADRQIFEEKVGPLVGRLRLTASLRQNGAKTAYRVNLKLDQADVVDSGLPKVRYTQVWSHDVTIVANSTEASRKSLYDLTKSLVATSQVEDLVVNLVPLGRGGSGGSKTIVLSVGEATRTLTEIQSTADRQIFEEKVGPLVGRLRLTASLRQNGAKTAYRVNLKLDQADVVDSGLPKVRYTQVWSHDVTIVANSTEASRKSLYDLTKSLVATSQVEDLVVNLVPLGR
final STV construct	MBD:A0A452QKQ4/budding domain:SynL/spacer:PCR XTEN-HiBiT-HA/RNA binding:tdPCP/assembly:HE0690	MGLQDDEDLQVLLKGSQLLKVKSNSWRRERFYKLQEDCKTIWQESRKVMRTPESHLFSIEDIQEVRKGHRTEGMEKFARDVPEDRCFSIVFKDQRNTLDLISPSPAEAQHWVRGLHKIIHTGPTAPPGSGSPTAPPEYSGSGPSAPPMEEKLLDLGGSGPYKEGSGSPPPPYVGSGLYPSLSGGFPIVSGGPLPPVGSGSYLDLGPGSEPATSGSETPGTSESATPESGPGGSGGVSGWRLFKKISGGGSKTIVLSVGEATRTLTEIQSTADRQIFEEKVGPLVGRLRLTASLRQNGAKTAYRVNLKLDQADVVDSGLPKVRYTQVWSHDVTIVANSTEASRKSLYDLTKSLVATSQVEDLVVNLVPLGRGGSGGSKTIVLSVGEATRTLTEIQSTADRQIFEEKVGPLVGRLRLTASLRQNGAKTAYRVNLKLDQADVVDSGLPKVRYTQVWSHDVTIVANSTEASRKSLYDLTKSLVATSQVEDLVVNLVPLGRGTGGYPYDVPDYAGGLKVKIKVKDNPAVARGVLRLADKLKKAGVDVEIEIDLYGDEEQALATLAAMEAEVEELA*

first STV construct	MBD:PHPLC/budding domain:SynL/spacer:PCR XTEN-HiBiT-HA/RNA binding:tdPCP/assembly:HE0902	MHGLQDDPDLQALLKGSQLLKVKSSSWRRERFYKLQEDCKTIWQESR<br>KVMRSPESQLFSIEDIQEVRMGHRTEGLEKFARDIPEDRCFSIVFKDQR<br>NTLDLIAPSPADAQHWVQGLRKIIHHSGSMDQRQKLQTGPTAPPGS<br>GSPTAPPEYSGSGPSAPPMEEKLLDLGGSGPYKEGSGSPPPPYVGSGL<br>YPSLSGGFPIVSGGPLPPVGSGSYLDLGPGSEPATSGSETPGTSESATPE<br>SGPGGSGGVSGWRLFKKISGGSGSKTIVLSVGEATRTLTEIQSTADRQI<br>FEEKVGPLVGRLRLTASLRQNGAKTAYRVNLKLDQADVVDSGLPKVRY<br>TQVWSHDVTIVANSTEASRKSLYDLTKSLVATSQVEDLVVNLVPLGRG<br>GSGGSKTIVLSVGEATRTLTEIQSTADRQIFEEKVGPLVGRLRLTASLRQ<br>NGAKTAYRVNLKLDQADVVDSGLPKVRYTQVWSHDVTIVANSTEASR<br>KSLYDLTKSLVATSQVEDLVVNLVPLGRGTGGYPYDVPDYAGGFTEEEI<br>KKIRESLKLSVEALEVTPKDFEKALELLEEVAINLMEIFKDDPMKALKIAF<br>KFTNAIAKLYVAHESKDVADAMAIMAEVTKYILEILEKVLEE*
PHPLC	MBD	MHGLQDDPDLQALLKGSQLLKVKSSSWRRERFYKLQEDCKTIWQESR<br>KVMRSPESQLFSIEDIQEVRMGHRTEGLEKFARDIPEDRCFSIVFKDQR<br>NTLDLIAPSPADAQHWVQGLRKIIHHSGSMDQRQKLQ
N Split Luc	Cargo	MEDAKNIKKGPAPRYPLEDGTAGEQLHKAMKRYAQVPGTIAFTDAHI<br>EVNITYAEYFEMSVRLAEAMKRYGLNTNHRIVVCSENSLQFFMPVLGA<br>LFIGVAVAPANDIYNERELLNSMNISQPTVVFVSKKGLQKILNVQKKLPI<br>IQKIIIMDSKTDYQGFQSMYTFVTSHLPPGFNEYDFKPESFDRDKTIALI<br>MNSSGSTGLPKGVALPHRTACVRFSHARDPIFGNQIKPDTAILSVVPF<br>HHGFGMFTTLGYLICGFRVVLMYRFEEELFLRSLQDYKIQSALLVPTLFS<br>FFAKSTLIDKYDLSNLHEIASGGAPLSKEVGEAVAKRFHLPGIRQGYGLT<br>ETTSAILITPEGDDKPGAVGKVVPFFEAKVVDLDTGKTLGVNQRGELCV<br>RGPMIMCLDLKTQVQTPQGMKEISNIQVGDLVLSNTGYNEVLNVFPK<br>SKKKSYKITLEDGKEIICSEEHLFPTQTGEMNISGGLKEGMCLYVKEGPG<br>SEPATSGSETPGTSESATPESGPGTGEIAALEAKIAALKAKNAALKAEIA<br>ALEAGSGNSHGFPPEVEEQAAGTLPMSCAQESGMDRHPAACASARI<br>NV
C Split Luc	incl Intein etc, LgBit (S64)	KIAALKAEIAALEAENAALEAKIAALKAGPGSEPATSGSETPGTSESATP<br>ESGPGMMLKKILKIEELDERELIDIEVSGNHLFYANDILTHNSGYVNNPE<br>ATNALIDKDGWLHSGDIAYWDEDEHFFIVDRLKSLIKYKGYQVAPAEL<br>ESILLQHPNIRDAGVAGLPDDDAGELPAAVVVLEHGKTMTEKEIVDYV<br>ASQVTTAKKLRGGVVFVDEVPKGLTGKLDARKIREILIKAKKGGKIAV
N Split Luc-PP7	incl PP7	AUGGAAGAUGCCAAGAACAUCAAGAAGGGCCCUGCUCCUAGAU<br>ACCCUCUGGAAGAUGGAACCGCUGGCGAGCAGCUGCACAAGGCC<br>AUGAAGAGAUACGCUCAGGUGCCCGGCACAAUCGCCUUCACAGA<br>UGCCCACAUCGAAGUGAACAUCACCUACGCCGAGUACUUCGAGA<br>UGAGCGUGCGGCUGGCCGAAGCUAUGAAGCGAUACGGCCUGAA<br>CACCAACCACAGAAUCGUCGUGUGCAGCGAGAACAGCCUCCAGU<br>UCUUCAUGCCUGUGCUGGGCGCUCUGUUCAUCGGAGUGGCUG<br>UGGCUCCUGCCAACGACAUCUACAACGAGCGCGAGCUGCUGAAC<br>AGCAUGAACAUCAGCCAGCCUACCGUGGUGUUCGUGUCCAAGA<br>AGGGACUGCAAAAGAUCCUGAACGUGCAGAAGAAGCUGCCCAU<br>CAUCCAGAAAAUCAUCAUCAUGGACAGCAAGACCGACUACCAGG<br>GCUUCCAGAGCAUGUAUACCUUCGUGACCAGCCAUCUGCCACCA<br>GGCUUCAACGAGUACGACUUCAAGCCCGAGAGCUUCGACAGAG<br>ACAAGACAAUCGCCCUGAUCAUGAACAGCAGCGGCUCUACCGGA<br>CUGCCUAAAGGCGUUGCCCUGCCUCACAGAACAGCUUGCGUCAG<br>AUUCAGCCACGCCAGAGAUCCCAUCUUCGGCAACCAGAUCAAGC<br>CUGACACCGCUAUCCUGAGCGUGGUGCCUUUUCACCACGGCUU<br>CGGCAUGUUCACCACACUGGGCUACCUGAUCUGCGGCUUCAGA

		GUGGUGCUGAUGUAUCGCUUUGAGGAAGAACUGUUCCUGCGG<br>AGCCUCCAGGACUACAAGAUCCAGUCUGCUCUGCUGGUGCCUAC<br>UCUGUUCAGCUUCUUUGCCAAGAGCACCCUGAUCGAUAAGUAC<br>GACCUGAGCAACCUGCACGAGAUCGCCUCUGGCGGAGCCCCUCU<br>GUCUAAAGAAGUGGGCGAAGCCGUCGCCAAGAGAUUUCAUCUG<br>CCCGGCAUCAGACAAGGCUACGGACUGACCGAGACAACCAGCGC<br>CAUCCUGAUCACACCUGAGGGCGACGAUAAGCCUGGCGCUGUG<br>GGAAAAGUGGUGCCAUUCUUCGAGGCUAAGGUGGUGGACCUG<br>GACACCGGCAAAACACUGGGAGUGAAUCAGAGGGGCGAGCUGU<br>GUGUCAGAGGCCCUAUGAUCAUGUGCCUGGACCUUAAGACCCA<br>GGUGCAGACCCCCCAGGGCAUGAAGGAAAUCAGCAACAUCCAAG<br>UGGGCGACCUGGUGCUGAGCAACACCGGCUACAACGAGGUGCU<br>GAACGUGUUCCCCAAGAGCAAGAAGAAGUCCUACAAGAUCACCC<br>UGGAAGAUGGCAAAGAGAUCAUCUGCUCCGAGGAACACCUGUU<br>CCCAACCCAGACCGGCGAGAUGAACAUCUCUGGCGGCCUGAAAG<br>AGGGCAUGUGCCUGUACGUGAAAGAAGGUCCCGGCUCUGAGCC<br>UGCUACCAGUGGGUCCGAAACCCCUGGCACCUCCGAGUCUGCCA<br>CCCCAGAGUCCGGCCCUGGGACCGGUGAGAUCGCGGCCCUCGAG<br>GCUAAAAUCGCUGCCCUCAAAGCGAAGAACGCCGCUCUCAAGGC<br>AGAGAUCGCGGCACUGGAAGCAGGCAGCGGAAACAGCCACGGC<br>UUCCCACCCGAGGUGGAAGAACAGGCUGCCGGAACCCUGCCUAU<br>GAGCUGCGCUCAGGAAUCCGGCAUGGACAGGCACCCUGCCGCU<br>UGUGCCUCCGCCAGAAUCAACGUGUGAACGCGUGAAUUCCGAC<br>CAGAAGAUAUGGCUUCGGUUGGGUUCAAA
EGFP-PP7	incl PP7	AUGGUGAGCAAGGGCGAGGAGCUGUUCACCGGGGUGGUGCCCA<br>UCCUGGUCGAGCUGGACGGCGACGUAAACGGCCACAAGUUCAG<br>CGUGUCCGGCGAGGGCGAGGGCGAUGCCACCUACGGCAAGCUG<br>ACCCUGAAGUUCAUCUGCACCACCGGCAAGCUGCCCGUGCCCUG<br>GCCCACCCUCGUGACCACCCUGACCUACGGCGUGCAGUGCUUCA<br>GCCGCUACCCCGACCACAUGAAGCAGCACGACUUCUUCAAGUCC<br>GCCAUGCCCGAAGGCUACGUCCAGGAGCGCACCAUCUUCUUCAA<br>GGACGACGGCAACUACAAGACCCGCGCCGAGGUGAAGUUCGAG<br>GGCGACACCCUGGUGAACCGCAUCGAGCUGAAGGGCAUCGACU<br>UCAAGGAGGACGGCAACAUCCUGGGGCACAAGCUGGAGUACAA<br>CUACAACAGCCACAACGUCUAUAUCAUGGCCGACAAGCAGAAGA<br>ACGGCAUCAAGGUGAACUUCAAGAUCCGCCACAACAUCGAGGAC<br>GGCAGCGUGCAGCUCGCCGACCACUACCAGCAGAACACCCCCAUC<br>GGCGACGGCCCCGUGCUGCUGCCCGACAACCACUACCUGAGCAC<br>CCAGUCCGCCCUGAGCAAAGACCCCAACGAGAAGCGCGAUCACA<br>UGGUCCUGCUGGAGUUCGUGACCGCCGCCGGGAUCACUCUCGG<br>CAUGGACGAGCUGUACAAGACCGGUUGAACGCGUGAAUUCCGA<br>CCAGAAGAUAUGGCUUCGGUUGGGUUCAAA
SEND Cargo GFP	incl Peg10	GGAUUUGGCGCCCCCCCUCCUAGGAUCUCUCUAUAUAAGGCAA<br>GCAGUCCGGACUCCCGAUACACGCGUUUCCAACUUGAGUUGGU<br>GUGUGUCGAAGAAUCCUGACCAACUACGACCUGGGGAGAGCAG<br>CCAACCGAGAAGGUCCACCGAGCCUCGCCUAGGUCUGCUGCGCG<br>GGCUGCGGUCCGGAGCCUUCUCCGGACCGCGGUCACCCAGUGG<br>ACCGGGCCGUCGCGGGACCCCUCAUCCUUCGUGGCAUCGCAGAG<br>GAAUCCUCGUGUGGAACAGGCGGGUUUUAAGAACCAAAAGACG<br>CCAACCACGAGGGUCCCAGGAUCCAGGGCUCCCUCCCCAGGGGA<br>GUGAAGCCCCUCUCACCGCAGCCAUGGUGAGCAAGGGCGAGGA<br>GCUGUUCACCGGGGUGGUGCCCAUCCUGGUCGAGCUGGACGGC

		GACGUAAACGGCCACAAGUUCAGCGUGUCCGGCGAGGGCGAGG<br>GCGAUGCCACCUACGGCAAGCUGACCCUGAAGUUCAUCUGCACC<br>ACCGGCAAGCUGCCCGUGCCCUGGCCCACCCUCGUGACCACCCU<br>GACCUACGGCGUGCAGUGCUUCAGCCGCUACCCCGACCACAUGA<br>AGCAGCACGACUUCUUCAAGUCCGCCAUGCCCGAAGGCUACGUC<br>CAGGAGCGCACCAUCUUCUUCAAGGACGACGGCAACUACAAGAC<br>CCGCGCCGAGGUGAAGUUCGAGGGCGACACCCUGGUGAACCGC<br>AUCGAGCUGAAGGGCAUCGACUUCAAGGAGGACGGCAACAUCC<br>UGGGGCACAAGCUGGAGUACAACUACAACAGCCACAACGUCUAU<br>AUCAUGGCCGACAAGCAGAAGAACGGCAUCAAGGUGAACUUCA<br>AGAUCCGCCACAACAUCGAGGACGGCAGCGUGCAGCUCGCCGAC<br>CACUACCAGCAGAACACCCCCAUCGGCGACGGCCCCGUGCUGCU<br>GCCCGACAACCACUACCUGAGCACCCAGUCCGCCCUGAGCAAAGA<br>CCCCAACGAGAAGCGCGAUCACAUGGUCCUGCUGGAGUUCGUG<br>ACCGCCGCCGGGAUCACUCUCGGCAUGGACGAGCUGUACAAGU<br>AACAUCCUGCAGUGCUGCGUGAAACCUGUCAUGUCCUUUGUAG<br>UCUCUGCCCUCAACUUGAUCCUGUGCAGCUUCUCAAUCUAUGA<br>CUGUGUGGUACUGGACCUUCAGAGGCGCACAGAGCUCAAGUCA<br>GUUUUCGUCUUGACUGCCACUUUAUAAGUUGACAGGCCUGGG<br>UUUUACUUGUUAAAACCUCUCACCAUCUCAAUCACAGGCUGCCA<br>AGUGUCUUUACAAAGAAGCUGAUACAAACACAGGCCAUGCUGA<br>UUUCUUACAGAGGGAGAGAAGAGGAAGAGAAGAAGAAAGAGG<br>AGGAAGAGGACAUGACUUGCCCAUAUGCUGGGCACCUUAUAAA<br>GGAAGCCAGACUUUUCGGUGCAGUAUGGAAAGGCUUCCGUGA<br>UUCUCUUGCUGCACCCCACGAAACUUCACCACCUUCAAACUCCA<br>UUUUCACGGUUCCGUUAAUUUUCAAGGAGCAGCAACUCGACUG<br>GUUCUCUGCUACAUGAAACACCUCAGCUUGAAAAGGAAGUGCU<br>CUCUCAGACUGACUUGUGAGUGUGCCUUCACAUUCUGGUGCAA<br>AUCAUG
VLP		MGARASVLSGGELDRWEKIRLRPGGKKKYKLKHIVWASRELERFAVN<br>PGLLETSEGCRQILGQLQPSLQTGSEELRSLYNTVATLYCVHQRIEIKDT<br>KEALDKIEEEQNKSKKKAQQAAADTGHSNQVSQNYPIVQNIQGQMV<br>HQAISPRTLNAWVKVVEEKAFSPEVIPMFSALSEGATPQDLNTMLNT<br>VGGHQAAMQMLKETINEEAAEWDRVHPVHAGPIAPGQMREPRGS<br>DIAGTTSTLQEQIGWMTNNPPIPVGEIYKRWIILGLNKIVRMYSPTSILD<br>IRQGPKEPFRDYVDRFYKTLRAEQASQEVKNWMTETLLVQNANPDCK<br>TILKALGPAATLEEMMTACQGVGGPGHKARVLAEAMSQVTNSATIM<br>MQRGNFRNQRKIVKCFNCGKEGHTARNCRAPRKKGCWKCGKEGHQ<br>MKDCTERQANFLGKIWPSYKGRPGNFLQSRPEPTAPPEESFRSGVETT<br>TPPQKQEPIDKELYPLTSLRSLFGNDPSSQASNFTQFVLVDNGGTGDV<br>TVAPSNFANGVAEWISSNSRSQAYKVTCSVRQSSAQKRKYTIKVEVPK<br>VATQTVGGVELPVAAWRSYLNMELTIPIFATNSDCELIVKAMQGLLKD<br>GNPIPSAIAANSGIY
EPN		MHGLQDDPDLQALLKGSQLLKVKSSSWRRERFYKLQEDCKTIWQESR<br>KVMRSPESQLFSIEDIQEVRMGHRTEGLEKFARDIPEDRCFSIVFKDQR<br>NTLDLIAPSPADAQHWVQGLRKIIHHSGSMDQRQKLQSRPEPTAPPE<br>ESFRSGVETTTPPQKQEPIDKELYPLTSLRSLFGNDPSSQKIEELFKKHKI<br>VAVLRANSVEEAKKKALAVFLGGVHLIEITFTVPDADTVIKELSFLKEMG<br>AIIGAGTVTSVEQCRKAVESGAEFIVSPHLDEEISQFCKEKGVFYMPGV<br>MTPTELVKAMKLGHTILKLFPGEVVGPQFVKAMKGPFPNVKFVPTGG<br>VNLDNVCEWFKAGVLAVGVGSALVKGTPVEVAEKAKAFVEKIRGCTE<br>QKLISEEDLASNFTQFVLVDNGGTGDVTVAPSNFANGVAEWISSNSRS

		QAYKVTCSVRQSSAQKRKYTIKVEVPKVATQTVGGVELPVAAWRSYL NMELTIPIFATNSDCELIVKAMQGLLKDGNPIPSAIAANSGIY
VLP and EPN EGFP cargo MS2	incl. MS2	AUGGUGAGCAAGGGCGAGGAGCUGUUCACCGGGGUGGUGCCCA UCCUGGUCGAGCUGGACGGCGACGUAAACGGCCACAAGUUCAG CGUGUCCGGCGAGGGCGAGGGCGAUGCCACCUACGGCAAGCUG ACCCUGAAGUUCAUCUGCACCACCGGCAAGCUGCCCGUGCCCUG GCCCACCCUCGUGACCACCCUGACCUACGGCGUGCAGUGCUUCA GCCGCUACCCCGACCACAUGAAGCAGCACGACUUCUUCAAGUCC GCCAUGCCCGAAGGCUACGUCCAGGAGCGCACCAUCUUCUUCAA GGACGACGGCAACUACAAGACCCGCGCCGAGGUGAAGUUCGAG GGCGACACCCUGGUGAACCGCAUCGAGCUGAAGGGCAUCGACU UCAAGGAGGACGGCAACAUCCUGGGGCACAAGCUGGAGUACAA CUACAACAGCCACAACGUCUAUAUCAUGGCCGACAAGCAGAAGA ACGGCAUCAAGGUGAACUUCAAGAUCCGCCACAACAUCGAGGAC GGCAGCGUGCAGCUCGCCGACCACUACCAGCAGAACACCCCCAUC GGCGACGGCCCCGUGCUGCUGCCCGACAACCACUACCUGAGCAC CCAGUCCGCCCUGAGCAAAGACCCCAACGAGAAGCGCGAUCACA UGGUCCUGCUGGAGUUCGUGACCGCCGCCGGGAUCACUCUCGG CAUGGACGAGCUGUACAAGACCGGUUGAACGCGUAACAUGAGG AUCACCCAUGU
AAVS1 gRNA	gRNA, tracr, termination	GGGACCACCUUAUAUUCCCAGUUUAAGAGCUAUGCUGGAAACA GCAUAGCAAGUUUAAAUAAGGCUAGUCCGUUAUCAACUUGAAA AAGUGGCACCGAGUCGGUGCUUUUUUU
EGFP-P2A-Ascl1-PP7		AUGGUGAGCAAGGGCGAGGAGCUGUUCACCGGGGUGGUGCCCA UCCUGGUCGAGCUGGACGGCGACGUAAACGGCCACAAGUUCAG CGUGUCCGGCGAGGGCGAGGGCGAUGCCACCUACGGCAAGCUG ACCCUGAAGUUCAUCUGCACCACCGGCAAGCUGCCCGUGCCCUG GCCCACCCUCGUGACCACCCUGACCUACGGCGUGCAGUGCUUCA GCCGCUACCCCGACCACAUGAAGCAGCACGACUUCUUCAAGUCC GCCAUGCCCGAAGGCUACGUCCAGGAGCGCACCAUCUUCUUCAA GGACGACGGCAACUACAAGACCCGCGCCGAGGUGAAGUUCGAG GGCGACACCCUGGUGAACCGCAUCGAGCUGAAGGGCAUCGACU UCAAGGAGGACGGCAACAUCCUGGGGCACAAGCUGGAGUACAA CUACAACAGCCACAACGUCUAUAUCAUGGCCGACAAGCAGAAGA ACGGCAUCAAGGUGAACUUCAAGAUCCGCCACAACAUCGAGGAC GGCAGCGUGCAGCUCGCCGACCACUACCAGCAGAACACCCCCAUC GGCGACGGCCCCGUGCUGCUGCCCGACAACCACUACCUGAGCAC CCAGUCCGCCCUGAGCAAAGACCCCAACGAGAAGCGCGAUCACA UGGUCCUGCUGGAGUUCGUGACCGCCGCCGGGAUCACUCUCGG CAUGGACGAGCUGUACAAGACCGGUGACUACAAGGACGACGAC GACAAGGGAAGCGGCGCCACCAACUUUAGCCUGCUCAAACAGGC CGGGGACGUUGAAGAGAAUCCCGGACCUGGAUCCAUGGAAAGC UCUGCCAAGAUGGAGAGCGGCGGCGCCGGCCAGCAGCCCCAGCC GCAGCCCCAGCAGCCCUUCCUGCCGCCCGCAGCCUGUUUCUUUG CCACGGCCGCAGCCGCGGCGGCCGCAGCCGCCGCAGCGGCAGCG CAGAGCGCGCAGCAGCAGCAGCAGCAGCAGCAGCAGCAGCAGCA GGCGCCGCAGCUGAGACCGGCGGCCGACGGCCAGCCCUCAGGGG GCGGUCACAAGUCAGCGCCCAAGCAAGUCAAGCGACAGCGCUCG UCUUCGCCCGAACUGAUGCGCUGCAAACGCCGGCUCAACUUCAG CGGCUUUGGCUACAGCCUGCCGCAGCAGCAGCCGGCCGCCGUGG CGCGCCGCAACGAGCGCGAGCGCAACCGCGUCAAGUUGGUCAAC

		CUGGGCUUUGCCACCCUUCGGGAGCACGUCCCCAACGGCGCGGC<br>CAACAAGAAGAUGAGUAAGGUGGAGACACUGCGCUCGGCGGUC<br>GAGUACAUCCGCGCGCUGCAGCAGCUGCUGGACGAGCAUGACG<br>CGGUGAGCGCCGCCUUCCAGGCAGGCGUCCUGUCGCCCACCAUC<br>UCCCCCAACUACUCCAACGACUUGAACUCCAUGGCCGGCUCGCC<br>GGUCUCAUCCUACUCGUCGGACGAGGGCUCUUACGACCCGCUC<br>AGCCCCGAGGAGCAGGAGCUUCUCGACUUCACCAACUGGUUCU<br>GAACGCGUGAAUUCCGACCAGAAGAUAUGGCUUCGGUUGGGU<br>UCAAA
Cas13d-NCS-PP7	incl PP7	ACCAUGAUCGAGAAGAAGAAGUCCUUCGCCAAAGGCAUGGGCG<br>UGAAGUCGACCCUGGUGUCCGGCAGCAAGGUGUACAUGACCAC<br>AUUCGCCGAGGGCAGCGACGCCAGACUGGAAAAGAUUGUGGAA<br>GGCGACAGCAUCCGCAGCGUGAACGAGGGCGAAGCUUUCUCUG<br>CCGAGAUGGCCGAUAAGAACGCUGGCUACAAGAUCGGCAACGCC<br>AAGUUCUCUCACCCCAAGGGCUAUGCCGUGGUGGCUAACAACCC<br>ACUGUACACAGGACCCGUGCAGCAGGAUAUGCUGGGCCUGAAA<br>GAGACUCUGGAAAAGCGGUACUUCGGCGAGAGCGCCGACGGCA<br>ACGACAACAUCUGCAUCCAAGUGAUCCACAACAUCCUGGACAUC<br>GAAAAGAUCCUGGCCGAGUACAUCACCAACGCCGCCUACGCCGU<br>GAACAACAUCAGCGGCCUGGACAAGGACAUCAUCGGCUUCGGCA<br>AGUUCAGCACCGUGUACACCUACGACGAGUUCAAGGACCCCGAG<br>CACCACAGAGCCGCCUUCAACAACAACGACAAGCUGAUCAACGCC<br>AUCAAGGCCCAGUACGAUGAGUUCGACAACUUCCUGGAUAACCC<br>CAGACUGGGCUACUUCGGCCAAGCCUUCUUUAGCAAAGAGGGC<br>CGCAACUACAUCAUCAACUACGGGAACGAGUGCUACGACAUUCU<br>GGCCCUCUUAAGCGGCCUGAGGCACUGGGUCGUGCACAACAAU<br>GAGGAAGAGAGCCGGAUAUCCAGAACCUGGCUGUACAACCUGG<br>ACAAAAAUCUGGACAACGAGUAUAUCAGCACCCUGAACUACCUG<br>UACGACCGGAUCACAAACGAGCUGACCAACAGCUUCAGCAAGAA<br>CAGCGCCGCCAACGUGAACUAUAUCGCCGAGACACUGGGCAUCA<br>ACCCCGCCGAGUUUGCCGAGCAGUACUUCAGAUUCAGCAUCAU<br>GAAGGAACAGAAGAACCUGGGCUUCAACAUCACAAAGCUGCGG<br>GAAGUGAUGCUGGACAGAAAGGACAUGAGCGAGAUCCGCAAGA<br>ACCACAAGGUGUUCGACUCCAUCAGGACCAAGGUCUAUACCAUG<br>AUGGACUUCGUGAUCUAUCGGUACUACAUCGAAGAGGACGCCA<br>AGGUGGCCGCUGCCAACAAGUCUCUGCCCGACAACGAGAAGUCU<br>CUGAGCGAGAAGGACAUCUUCGUGAUUAACCUGAGGGGCAGCU<br>UCAACGACGACCAGAAGGACGCCCUGUACUACGAUGAGGCCAAC<br>AGAAUUUGGCGGAAGCUGGAAAACAUCAUGCACAACAUCAAAG<br>AGUUCCGGGGCAACAAGACCCGCGAGUACAAGAAGAAAGACGCC<br>CCUAGACUGCCCAGAAUCCUGCCUGCUGGCAGAGAUGUGUCCG<br>CCUUCUCUAAGCUGAUGUACGCCCUGACCAUGUUCCUGGACGG<br>CAAAGAGAUCAACGACCUGCUGACCACACUGAUCAACAAGUUCG<br>AUAACAUCCAGAGCUUUCUGAAAGUGAUGCCCCUGAUCGGCGU<br>GAACGCUAAGUUCGUGGAAGAGUACGCCUUCUUCAAGGACAGC<br>GCCAAGAUCGCCGACGAGCUGAGACUGAUCAAGAGCUUCGCCAG<br>AAUGGGCGAGCCUAUCGCCGAUGCUAGAAGGGCCAUGUACAUC<br>GACGCCAUUAGAAUCCUGGGCACCAACCUGAGCUACGACGAACU<br>GAAGGCUCUGGCUGACACCUUCAGCCUGGACGAGAACGGCAACA<br>AGCUGAAGAAAGGCAAGCACGGCAUGCGGAACUUUAUCAUCAA<br>CAACGUGAUCAGCAACAAGCGGUUCCACUACCUGAUCAGAUACG<br>GCGACCCCGCUCAUCUGCACGAGAUCGCUAAGAACGAGGCCGUG

		GUCAAGUUCGUGCUGGGCAGAAUCGCCGACAUCCAGAAGAAGCAGGGACAGAACGGAAAGAACCAGAUCGACCGCUACUACGAGACAUGCAUCGGCAAGGACAAGGGCAAGAGCGUGUCCGAGAAAGUGGACGCUCUGACCAAGAUCAUCACCGGCAUGAACUACGACCAGUUUGACAAAAAGCGGAGCGUGAUCGAGGACACCGGCAGAGAGAAUGCCGAGAGAGAGAAGUUCAAGAAGAUCAUCUCCCUGUACCUGACCGUGAUCUACCACAUCCUCAAGAACAUCGUGAACAUCAACGCUCGCUACGUGAUCGGCUUCCACUGCGUGGAAAGAGAUGCCCAGCUGUACAAAGAGAAGGGAUACGACAUCAACCUGAAGAAGCUCGAAGAAAAGGGCUUCAGCAGCGUGACCAAGCUGUGCGCUGGAAUCGACGAGACAGCCCCUGACAAGCGGAAGGACGUGGAAAAAGAGAUGGCUGAGAGAGCCAAAGAGAGCAUCGACAGCCUGGAAAGCGCCAAUCCUAAGCUGUACGCCAAUUACAUCAAGUACUCCGACGAGAAGAAAGCCGAAGAGUUCACCAGGCAGAUCAACAGAGAGAAGGCCAAGACAGCCCUGAACGCCUACCUGAGAAACACCAAGUGGAAUGUGAUCAUCCGGGAAGAUCUGCUGCGGAUCGAUAACAAGACCUGCACACUGUUCAGAAACAAGGCCGUGCACCUGGAAGUCGCGAGAUACGUGCACGCCUACAUCAACGACAUUGCCGAAGUGAACAGCUACUUCCAGCUCUACCACUAUAUCAUGCAGCGGAUCAUCAUGAACGAGCGAUAUGAGAAGUCCAGCGGCAAGGUGUCCGAGUACUUCGAUGCCGUGAACGAUGAGAAGAAGUACAACGACCGGCUGCUGAAACUGCUGUGCGUGCCAUUCGGCUACUGCAUCCCACGGUUCAAGAACCUGAGCAUCGAGGCCCUGUUCGACAGAAACGAGGCUGCCAAAUUCGACAAAGAAAAGAAAAAAGUGUCCGGGAACAGCGGAUCCACGCCGCCCAAGAAGAAGAGAAAGGUUGAGGACGGCGAGGGCUUGCAGCUGCCUCCACUGGAAAGACUGACACUCUCCGGAGCUGCUCCUGCCGCCAAGAAAAAGAAACUCGACUACAAGGACGACGACGACAAGUGAACGCGUGAAUUCCGACCAGAAGAUAUGGCUUCGGUUGGGUUCAAA
SARS-CoV-2 Cas13 crRNA PP7		GAACCCCUACCAACUGGUCGGGGUUUGAAACGUCAUCCAAUUUGAUGGCACCUGUGUGCUAGCGAAUUCCGACCAGAAGAUAUGGCUUCGGUUGGGUUCAAA
Cas13 non-target crRNA PP7		GAACCCCUACCAACUGGUCGGGGUUUGAAACCUCAAAGCGAUACAUCAGCACCACUCGCUAGCGAAUUCCGACCAGAAGAUAUGGCUUCGGUUGGGUUCAAA

Cas9-NLS-NES-PP7	incl PP7	AUGGACAAGAAGUACAGCAUCGGCCUGGACAUCGGCACCAACUC<br>UGUGGGCUGGGCCGUGAUCACCGACGAGUACAAGGUGCCCAGC<br>AAGAAAUUCAAGGUGCUGGGCAACACCGACCGGCACAGCAUCAA<br>GAAGAACCUGAUCGGAGCCCUGCUGUUCGACAGCGGCGAAACA<br>GCCGAGGCCACCCGGCUGAAGAGAACCGCCAGAAGAAGAUACAC<br>CAGACGGAAGAACCGGAUCUGCUAUCUGCAAGAGAUCUUCAGC<br>AACGAGAUGGCCAAGGUGGACGACAGCUUCUUCCACAGACUGG<br>AAGAGUCCUUCCUGGUGGAAGAGGAUAAGAAGCACGAGCGGCA<br>CCCCAUCUUCGGCAACAUCGUGGACGAGGUGGCCUACCACGAGA<br>AGUACCCCACCAUCUACCACCUGAGAAAGAAACUGGUGGACAGC<br>ACCGACAAGGCCGACCUGCGGCUGAUCUAUCUGGCCCUGGCCCA<br>CAUGAUCAAGUUCCGGGGCCACUUCCUGAUCGAGGGCGACCUG<br>AACCCCGACAACAGCGACGUGGACAAGCUGUUCAUCCAGCUGGU<br>GCAGACCUACAACCAGCUGUUCGAGGAAAACCCCAUCAACGCCA<br>GCGGCGUGGACGCCAAGGCCAUCCUGUCUGCCAGACUGAGCAA<br>GAGCAGACGGCUGGAAAAUCUGAUCGCCCAGCUGCCCGGCGAG<br>AAGAAGAAUGGCCUGUUCGGCAACCUGAUUGCCCUGAGCCUGG<br>GCCUGACCCCCAACUUCAAGAGCAACUUCGACCUGGCCGAGGAU<br>GCCAAACUGCAGCUGAGCAAGGACACCUACGACGACGACCUGGA<br>CAACCUGCUGGCCCAGAUCGGCGACCAGUACGCCGACCUGUUUC<br>UGGCCGCCAAGAACCUGUCCGACGCCAUCCUGCUGAGCGACAUC<br>CUGAGAGUGAACACCGAGAUCACCAAGGCCCCCCUGAGCGCCUC<br>UAUGAUCAAGAGAUACGACGAGCACCACCAGGACCUGACCCUGC<br>UGAAAGCUCUCGUGCGGCAGCAGCUGCCUGAGAAGUACAAAGA<br>GAUUUUCUUCGACCAGAGCAAGAACGGCUACGCCGGCUACAUU<br>GACGGCGGAGCCAGCCAGGAAGAGUUCUACAAGUUCAUCAAGC<br>CCAUCCUGGAAAAGAUGGACGGCACCGAGGAACUGCUCGUGAA<br>GCUGAACAGAGAGGACCUGCUGCGGAAGCAGCGGACCUUCGAC<br>AACGGCAGCAUCCCCCACCAGAUCCACCUGGGAGAGCUGCACGC<br>CAUUCUGCGGCGGCAGGAAGAUUUUUACCCAUUCCUGAAGGAC<br>AACCGGGAAAAGAUCGAGAAGAUCCUGACCUUCCGCAUCCCCUA<br>CUACGUGGGCCCUCUGGCCAGGGGAAACAGCAGAUUCGCCUGG<br>AUGACCAGAAAGAGCGAGGAAACCAUCACCCCCUGGAACUUCGA<br>GGAAGUGGUGGACAAGGGCGCUUCCGCCCAGAGCUUCAUCGAG<br>CGGAUGACCAACUUCGAUAAGAACCUGCCCAACGAGAAGGUGCU<br>GCCCAAGCACAGCCUGCUGUACGAGUACUUCACCGUGUAUAACG<br>AGCUGACCAAAGUGAAAUACGUGACCGAGGGAAUGAGAAAGCC<br>CGCCUUCCUGAGCGGCGAGCAGAAAAAGGCCAUCGUGGACCUGC<br>UGUUCAAGACCAACCGGAAAGUGACCGUGAAGCAGCUGAAAGA<br>GGACUACUUCAAGAAAAUCGAGUGCUUCGACUCCGUGGAAAUC<br>UCCGGCGUGGAAGAUCGGUUCAACGCCUCCCUGGGCACAUACCA<br>CGAUCUGCUGAAAAUUAUCAAGGACAAGGACUUCCUGGACAAU<br>GAGGAAAACGAGGACAUUCUGGAAGAUAUCGUGCUGACCCUGA<br>CACUGUUUGAGGACAGAGAGAUGAUCGAGGAACGGCUGAAAAC<br>CUAUGCCCACCUGUUCGACGACAAAGUGAUGAAGCAGCUGAAG<br>CGGCGGAGAUACACCGGCUGGGGCAGGCUGAGCCGGAAGCUGA<br>UCAACGGCAUCCGGGACAAGCAGUCCGGCAAGACAAUCCUGGAU<br>UUCCUGAAGUCCGACGGCUUCGCCAACAGAAACUUCAUGCAGCU<br>GAUCCACGACGACAGCCUGACCUUUAAAGAGGACAUCCAGAAAG<br>CCCAGGUGUCCGGCCAGGGCGAUAGCCUGCACGAGCACAUUGCC<br>AAUCUGGCCGGCAGCCCCGCCAUUAAGAAGGGCAUCCUGCAGAC<br>AGUGAAGGUGGUGGACGAGCUCGUGAAAGUGAUGGGCCGGCA<br>CAAGCCCGAGAACAUCGUGAUCGAAAUGGCCAGAGAGAACCAGA

		CCACCCAGAAGGGACAGAAGAACAGCCGCGAGAGAAUGAAGCGG<br>AUCGAAGAGGGCAUCAAAGAGCUGGGCAGCCAGAUCCUGAAAG<br>AACACCCCGUGGAAAACACCCAGCUGCAGAACGAGAAGCUGUAC<br>CUGUACUACCUGCAGAAUGGGCGGAUAUGUACGUGGACCAGG<br>AACUGGACAUCAACCGGCUGUCCGACUACGAUGUGGACCAUAU<br>CGUGCCUCAGAGCUUUCUGAAGGACGACUCCAUCGACAACAAG<br>GUGCUGACCAGAAGCGACAAGAACCGGGGCAAGAGCGACAACGU<br>GCCCUCCGAAGAGGUCGUGAAGAAGAUGAAGAACUACUGGCGG<br>CAGCUGCUGAACGCCAAGCUGAUUACCCAGAGAAAGUUCGACAA<br>UCUGACCAAGGCCGAGAGAGGCGGCCUGAGCGAACUGGAUAAG<br>GCCGGCUUCAUCAAGAGACAGCUGGUGGAAACCCGGCAGAUCAC<br>AAAGCACGUGGCACAGAUCCUGGACUCCCGGAUGAACACUAAGU<br>ACGACGAGAAUGACAAGCUGAUCCGGGAAGUGAAAGUGAUCAC<br>CCUGAAGUCCAAGCUGGUGUCCGAUUUCCGGAAGGAUUUCCAG<br>UUUUACAAAGUGCGCGAGAUCAACAACUACCACCACGCCCACGA<br>CGCCUACCUGAACGCCGUCGUGGGAACCGCCCUGAUCAAAAAGU<br>ACCCUAAGCUGGAAAGCGAGUUCGUGUACGGCGACUACAAGGU<br>GUACGACGUGCGGAAGAUGAUCGCCAAGAGCGAGCAGGAAAUC<br>GGCAAGGCUACCGCCAAGUACUUCUUCUACAGCAACAUCAUGAA<br>CUUUUUCAAGACCGAGAUUACCCUGGCCAACGGCGAGAUCCGG<br>AAGCGGCCUCUGAUCGAGACAAACGGCGAAACCGGGGAGAUCG<br>UGUGGGAUAAGGGCCGGGAUUUUGCCACCGUGCGGAAAGUGC<br>UGAGCAUGCCCCAAGUGAAUAUCGUGAAAAAGACCGAGGUGCA<br>GACAGGCGGCUUCAGCAAAGAGUCUAUCCUGCCCAAGAGGAACA<br>GCGAUAAGCUGAUCGCCAGAAAGAAGGACUGGGACCCUAAGAA<br>GUACGGCGGCUUCGACAGCCCCACCGUGGCCUAUUCUGUGCUG<br>GUGGUGGCCAAAGUGGAAAAGGGCAAGUCCAAGAAACUGAAGA<br>GUGUGAAAGAGCUGCUGGGGAUCACCAUCAUGGAAAGAAGCAG<br>CUUCGAGAAGAAUCCCAUCGACUUUCUGGAAGCCAAGGGCUAC<br>AAAGAAGUGAAAAAGGACCUGAUCAUCAAGCUGCCUAAGUACU<br>CCCUGUUCGAGCUGGAAAACGGCCGGAAGAGAAUGCUGGCCUC<br>UGCCGGCGAACUGCAGAAGGGAAACGAACUGGCCCUGCCCUCCA<br>AAUAUGUGAACUUCCUGUACCUGGCCAGCCACUAUGAGAAGCU<br>GAAGGGCUCCCCCGAGGAUAAUGAGCAGAAACAGCUGUUUGUG<br>GAACAGCACAAGCACUACCUGGACGAGAUCAUCGAGCAGAUCAG<br>CGAGUUCUCCAAGAGAGUGAUCCUGGCCGACGCUAAUCUGGAC<br>AAAGUGCUGUCCGCCUACAACAAGCACCGGGAUAAGCCCAUCAG<br>AGAGCAGGCCGAGAAUAUCAUCCACCUGUUUACCCUGACCAAUC<br>UGGGAGCCCCUGCCGCCUUCAAGUACUUUGACACCACCAUCGAC<br>CGGAAGAGGUACACCAGCACCAAAGAGGUGCUGGACGCCACCCU<br>GAUCCACCAGAGCAUCACCGGCCUGUACGAGACACGGAUCGACC<br>UGUCUCAGCUGGGAGGCGACGGAUCCACGCCGCCCAAGAAGAA<br>GAGAAAGGUUGAGGACGGCGAGGGCCCAGCCGCUAAGAGAGUG<br>AAACUGGAUUCCGGAGCUGCUCCUGCCGCCAAGAAAAAGAAACU<br>CGACUACAAGGACGACGACGACAAGUUGCAGCUGCCCCCUCUCG<br>AAAGGCUGACUCUCUGAACGCGUGAAUUCCGACCAGAAGAUAU<br>GGCUUCGGUUGGGUUCAAA
Exon51 DMD gRNA1 PP7	gRNA, PP7, tracr, termination	AGAGUUCCUAAGGUAGAGAGGUUUAAGAGCUAUGCUGGAGGG<br>GAAUUCCGACCAGAAGAUAUGGCUUCGGUUGGGUUCAAACCCA<br>ACAGCAUAGCAAGUUUAAAUAAGGCUAGUCCGUUAUCAACUUG<br>AAAAAGUGGCACCGAGUCGGUGCUGAGACGCGCGGUUCUAUCU<br>AGUUACGCGUUAAACCAACUAGAAUUUUUU

Exon51 DMD gRNA2 PP7	gRNA, PP7, tracr, termination	AUAAAGAUAAGAGCUGGCAGGUUUAAGAGCUAUGCUGGAGGG<br>GAAUUCCGACCAGAAGAUAUGGCUUCGGUUGGGUUCAAACCCA<br>ACAGCAUAGCAAGUUUAAAUAAGGCUAGUCCGUUAUCAACUUG<br>AAAAAGUGGCACCGAGUCGGUGCUGAGACGCGCGGUUCUAUCU<br>AGUUACGCGUUAAACCAACUAGAAUUUUUU
mRuby3-PP7	mRuby3, RNA size 1000nt, PP7	AUGGGAUCCGUGUCCAAGGGCGAAGAACUGAUCAAAGAAAACA<br>UGCGGAUGAAGGUGGUCAUGGAAGGCAGCGUGAACGGCCACCA<br>GUUCAAGUGUACAGGCGAAGGCGAGGGCAGACCUUAUGAGGGC<br>GUGCAGACCAUGAGAAUCAAAGUGAUCGAAGGCGGCCCUCUGC<br>CUUUCGCUUUCGACAUCCUGGCCACCAGCUUUAUGUACGGCAG<br>CAGAACCUUCAUCAAGUACCCCGCUGACAUCCCCGAUUUCUUCA<br>AGCAGAGCUUCCCCGAGGGCUUCACCUGGGAGAGAGUGACCAG<br>AUACGAGGAUGGCGGCGUGGUCACAGUGACCCAGGACACAUCU<br>CUGGAAGAUGGCGAGCUGGUGUACAACGUGAAAGUGCGGGGC<br>GUGAACUUCCCCUCUAACGGCCCUGUGAUGCAGAAAAAGACCAA<br>AGGCUGGGAGCCCAACACCGAGAUGAUGUACCCUGCUGAUGGC<br>GGCCUGAGAGGCUACACAGAUAUCGCCCUGAAAGUGGACGGCG<br>GAGGCCAUCUGCACUGCAACUUCGUGACCACCUACAGGUCCAAG<br>AAAACCGUGGGCAACAUCAAGAUGCCUGGGGUGCACGCCGUGG<br>ACCACAGACUGGAAAGAAUCGAGGAAAGCGACAACGAGACAUAC<br>GUGGUGCAGCGCGAAGUGGCCGUGGCCAAGUACUCUAAUCUCG<br>GAGGCGGCAUGGACGAGCUGUACAAAGGAUCUCUCCAGCUGCC<br>ACCUCUGGAAAGACUGACCCUGCAGGCAGGCUACCCUUACGACG<br>UGCCAGAUUACGCCUGAACGCGUGAAUUCCGACCAGAAGAUAU<br>GGCUUCGGUUGGGUUCAAA
mRuby3-Fillerup2000nt-PP7	mRuby3, RNA size 2000nt, PP7	AUGGGAUCCGUGUCCAAGGGCGAAGAACUGAUCAAAGAAAACA<br>UGCGGAUGAAGGUGGUCAUGGAAGGCAGCGUGAACGGCCACCA<br>GUUCAAGUGUACAGGCGAAGGCGAGGGCAGACCUUAUGAGGGC<br>GUGCAGACCAUGAGAAUCAAAGUGAUCGAAGGCGGCCCUCUGC<br>CUUUCGCUUUCGACAUCCUGGCCACCAGCUUUAUGUACGGCAG<br>CAGAACCUUCAUCAAGUACCCCGCUGACAUCCCCGAUUUCUUCA<br>AGCAGAGCUUCCCCGAGGGCUUCACCUGGGAGAGAGUGACCAG<br>AUACGAGGAUGGCGGCGUGGUCACAGUGACCCAGGACACAUCU<br>CUGGAAGAUGGCGAGCUGGUGUACAACGUGAAAGUGCGGGGC<br>GUGAACUUCCCCUCUAACGGCCCUGUGAUGCAGAAAAAGACCAA<br>AGGCUGGGAGCCCAACACCGAGAUGAUGUACCCUGCUGAUGGC<br>GGCCUGAGAGGCUACACAGAUAUCGCCCUGAAAGUGGACGGCG<br>GAGGCCAUCUGCACUGCAACUUCGUGACCACCUACAGGUCCAAG<br>AAAACCGUGGGCAACAUCAAGAUGCCUGGGGUGCACGCCGUGG<br>ACCACAGACUGGAAAGAAUCGAGGAAAGCGACAACGAGACAUAC<br>GUGGUGCAGCGCGAAGUGGCCGUGGCCAAGUACUCUAAUCUCG<br>GAGGCGGCAUGGACGAGCUGUACAAAGGAUCUCUCCAGCUGCC<br>ACCUCUGGAAAGACUGACCCUGCAGGCAGGCUACCCUUACGACG<br>UGCCAGAUUACGCCUGAGGUACCCUUGUACAGCUCGUCCAUGC<br>CGAGAGUGAUCCCGGCGGCGGUCACGAACUCCAGCAGGACCAUG<br>UGAUCGCGCUUCUCGUUGGGGUCUUUGCUCAGGGCGGACUGG<br>GUGCUCAGGUAGUGGUUGUCGGGCAGCAGCACGGGGCCGUCGC<br>CGAUGGGGGUGUUCUGCUGGUAGUGGUCGGCGAGCUGCACGC<br>UGCCGUCCUCGAUGUUGUGGCGGAUCUUGAAGUUCACCUUGA<br>UGCCGUUCUUCUGCUUGUCGGCCAUGAUAUAGACGUUGUGGC<br>UGUUGUAGUUGUACUCCAGCUUGUGCCCCAGGAUGUUGCCGU<br>CCUCCUUGAAGUCGAUGCCCUUCAGCUCGAUGCGGUUCACCAG

		GGUGUCGCCCUCGAACUUCACCUCGGCGCGGGUCUUGUAGUUG<br>CCGUCGUCCUUGAAGAAGAUGGUGCGCUCCUGGACGUAGCCUU<br>CGGGCAUGGCGGACUUGAAGAAGUCGUGCUGCUUCAUGUGGU<br>CGGGGUAGCGGCUGAAGCACUGCACGCCGUAGGUCAGGGUGGU<br>CACGAGGGUGGGCCAGGGCACGGGCAGCUUGCCGGUGGUGCAG<br>AUGAACUUCAGGGUCAGCUUGCCGUAGGUGGCAUCGCCCUCGC<br>CCUCGCCGGACACGCUGAACUUGUGGCCGUUUACGUCGCCGUC<br>CAGCUCGACCAGGAUGGGCACCACCCCGGUGAACAGCUCCUCGC<br>CCUUGCUCACCAUACGCGUGAAUUCCGACCAGAAGAUAUGGCU<br>UCGGUUGGGUUCAAA
mRuby3-Fillerup4000nt-PP7	mRuby3, RNA size 4000nt, PP7	AUGGGAUCCGUGUCCAAGGGCGAAGAACUGAUCAAAGAAAACA<br>UGCGGAUGAAGGUGGUCAUGGAAGGCAGCGUGAACGGCCACCA<br>GUUCAAGUGUACAGGCGAAGGCGAGGGCAGACCUUAUGAGGGC<br>GUGCAGACCAUGAGAAUCAAAGUGAUCGAAGGCGGCCCUCUGC<br>CUUUCGCUUUCGACAUCCUGGCCACCAGCUUUAUGUACGGCAG<br>CAGAACCUUCAUCAAGUACCCCGCUGACAUCCCCGAUUUCUUCA<br>AGCAGAGCUUCCCCGAGGGCUUCACCUGGGAGAGAGUGACCAG<br>AUACGAGGAUGGCGGCGUGGUCACAGUGACCCAGGACACAUCU<br>CUGGAAGAUGGCGAGCUGGUGUACAACGUGAAAGUGCGGGGC<br>GUGAACUUCCCCUCUAACGGCCCUGUGAUGCAGAAAAAGACCAA<br>AGGCUGGGAGCCCAACACCGAGAUGAUGUACCCUGCUGAUGGC<br>GGCCUGAGAGGCUACACAGAUAUCGCCCUGAAAGUGGACGGCG<br>GAGGCCAUCUGCACUGCAACUUCGUGACCACCUACAGGUCCAAG<br>AAAACCGUGGGCAACAUCAAGAUGCCUGGGGUGCACGCCGUGG<br>ACCACAGACUGGAAAGAAUCGAGGAAAGCGACAACGAGACAUAC<br>GUGGUGCAGCGCGAAGUGGCCGUGGCCAAGUACUCUAAUCUCG<br>GAGGCGGCAUGGACGAGCUGUACAAAGGAUCUCUCCAGCUGCC<br>ACCUCUGGAAAGACUGACCCUGCAGGCAGGCUACCCUUACGACG<br>UGCCAGAUUACGCCUGAGGUACCCUUGUACAGCUCGUCCAUGC<br>CGAGAGUGAUCCCGGCGGCGGUCACGAACUCCAGCAGGACCAUG<br>UGAUCGCGCUUCUCGUUGGGGUCUUUGCUCAGGGCGGACUGG<br>GUGCUCAGGUAGUGGUUGUCGGGCAGCAGCACGGGGCCGUCGC<br>CGAUGGGGGUGUUCUGCUGGUAGUGGUCGGCGAGCUGCACGC<br>UGCCGUCCUCGAUGUUGUGGCGGAUCUUGAAGUUCACCUUGA<br>UGCCGUUCUUCUGCUUGUCGGCCAUGAUAUAGACGUUGUGGC<br>UGUUGUAGUUGUACUCCAGCUUGUGCCCCAGGAUGUUGCCGU<br>CCUCCUUGAAGUCGAUGCCCUUCAGCUCGAUGCGGUUCACCAG<br>GGUGUCGCCCUCGAACUUCACCUCGGCGCGGGUCUUGUAGUUG<br>CCGUCGUCCUUGAAGAAGAUGGUGCGCUCCUGGACGUAGCCUU<br>CGGGCAUGGCGGACUUGAAGAAGUCGUGCUGCUUCAUGUGGU<br>CGGGGUAGCGGCUGAAGCACUGCACGCCGUAGGUCAGGGUGGU<br>CACGAGGGUGGGCCAGGGCACGGGCAGCUUGCCGGUGGUGCAG<br>AUGAACUUCAGGGUCAGCUUGCCGUAGGUGGCAUCGCCCUCGC<br>CCUCGCCGGACACGCUGAACUUGUGGCCGUUUACGUCGCCGUC<br>CAGCUCGACCAGGAUGGGCACCACCCCGGUGAACAGCUCCUCGC<br>CCUUGCUCACCAUUAAGGUACCUCUGGUGGGUAUAGAUUCUGU<br>GUAAAAUUGAUUCCAAUGCCAUCCACUUGAUAGGCACUUUGCC<br>UCCUUCUGCAUGGUAUUCUUUCUCUUCCGCACCCAGCAGUUUG<br>GCCAGCCCAAAAUCUGUGAUCUUGACAUGCUGCGGUGUUUUCA<br>CCAGUACGUUCCUGGCUGCCAGGUCGCGGUGCACCAAGCGACG<br>GUCCUCCAAGUAGUUCAUGCCCUUUGCGAUCUGCACACACCAG<br>UUGAGCAGGUACUGGGAGCCAAUAUUGUCUUUGUGUUCCCGG

		ACAUAGUCCAGGAGGCAGCCGAAGGGCAUGAGCUGCGUGAUGAGUUGCACGGUGGAGGUGAGGCAGAUGCCCAGCAGGCGGCACACGUGGGGGUUGUCCACGCUGGCCAUCACGUAGGCUUCAUCGAGGAUUUCCUUGUUGGCUUUCGGAGAUGUUGCUUCUCUUAAUUCCUUGAUAGCGACGGGAAUUUUAACUUUCUCACCUUCUGGGAUCCAGAGUCCCUUAUACACCGUGCCGAACGCACCGGAGCCCAGCACUUUGAUCUUUUUGAAUUCAGUUUCCUUCAAGAUCCUCAAGAGAGCUUGGUUGGGAGCUUCUCCACUGGGUGUAAGAGGCUCCACAAGCUCCCUCUCCUGCAGCAGCCUCCGCAGCGUGCGCUUCCGAACGAUGUGGCGCCUUCGCAUGAAGAGGCCGAUCCCCAGGGCCACCACCAGCAGCAAGAGGAGGGCCCCCACCAUCCCAGUGGCGAUGGACGGGAUCUUAGGCCCAUUCGUUGGACAGCCUUCAAGACCUGGCCCAGUGCAUCCGUAGGUGCAGUUUGGAUGGCACAGGUGGCACACAUGGCCGGCGUCUGCGUACUUCCAGACCAGGGUGUUGUUUUCUCCCAUGACUCCUGCCGGGCAGGUCUUGACGCAGUGGGGGCCGUCAAUGUAGUGGGCACACUGGAUACAGUUGUCUGGUCCCCGUCCUGUGCAGGUGAUGUUCAUGGCCUGAGGCAGGCACUCUGGGUGGCACUGUAUGCACUCAGAGUUCUCCACAAACUCCCUUGGCUCACCCUCCAGAAGGUUGCACUUGUCCACGCAUUCCCUGCCUCGGCUGACAUUCCGGCAAGAGACGCAGUCCCUGGGCUCCGGGCCCCAGCAGCCCUCGGGGGAGCACAAGGCAUGGCAGACCUGGCCUGUGGCCUUGCAGCUGUUUUCACCUCUGUUGCUUAUAAUUUUGGUUUUCUGACCGGAGGUCCCAAACAGUUUUUUCCAGUUUAUUGUAUUUGCAUAGCACAAAUUUUUGUUUCCUGAAAUUAUCACAUCUCCAUCACUUAUCUCCUUGAGGGAGCGUAAUCCCAAGGAUGUUAUGUUCAGGCUGACGACUGCAAGAGAAAACUGACCAUGUUGCUUGGUCCUGCCGCGUAUGAUUUCUAGGUUCUCAAAGGCAUGGAGGUCCGUCCUGUUUUCAGGCCAAGCCUGAAUCAGCAAAAACCCUGUGAUUUCCUUUACGGUUUUCAGAAUAUCCAGUUCCUGUGGAUCCAGAGGAGGAGUAUGUGUGAAGGAGUCACCCCUAAAUGCCACCGGCAGGAUGUGGAGAUCGCCACUGAUGGAGGUGCAGUUUUUGAAGUGUUUAAUAUUCGUAGCAUUUAUGGAGAGUGAGUCUUUAAAUUCACCAAUACCUAUUCCGUUACACACUUUGCGGCAAGGCCCUUCGCACUUCUUACACUUGCGGACGCCGUCUUCCUCCAUCUCAUAGCUGUCGGCCCCACAGGCUCGGACGCACGAGCCGUGAUCUGUCACCACAUAAUUACGGGGACACUUCUUCACGCAGGUGGCACCAAAGCUGUAUUUGCCCUCGGGGUUCACAUCCAUCUGGUACGUGGUGGGGUUGUAGAGCAUGAGUGGGGGGCAGGUGUCCUUGCACGUGGCUUCGUCUCGGAAUUUGCGGCAGACCAGGCAGUCGCUCUCCCGGGGGCCUGUGCAGCCUGCAGCACACUGGUUGUGGCAGCAGUCACUGGGGGACUUGACGCGUGAAUUCCGACCAGAAGAUAUGGCUUCGGUUGGGUUCAAA

mRuby3-Fillerup6000nt-PP7	mRuby3, RNA size 6000nt, PP7	AUGGGAUCCGUGUCCAAGGGCGAAGAACUGAUCAAAGAAAACA<br>UGCGGAUGAAGGUGGUCAUGGAAGGCAGCGUGAACGGCCACCA<br>GUUCAAGUGUACAGGCGAAGGCGAGGGCAGACCUUAUGAGGGC<br>GUGCAGACCAUGAGAAUCAAAGUGAUCGAAGGCGGCCCUCUGC<br>CUUUCGCUUUCGACAUCCUGGCCACCAGCUUUAUGUACGGCAG<br>CAGAACCUUCAUCAAGUACCCCGCUGACAUCCCCGAUUUCUUCA<br>AGCAGAGCUUCCCCGAGGGCUUCACCUGGGAGAGAGUGACCAG<br>AUACGAGGAUGGCGGCGUGGUCACAGUGACCCAGGACACAUCU<br>CUGGAAGAUGGCGAGCUGGUGUACAACGUGAAAGUGCGGGGC<br>GUGAACUUCCCCUCUAACGGCCCUGUGAUGCAGAAAAAGACCAA<br>AGGCUGGGAGCCCAACACCGAGAUGAUGUACCCUGCUGAUGGC<br>GGCCUGAGAGGCUACACAGAUAUCGCCCUGAAAGUGGACGGCG<br>GAGGCCAUCUGCACUGCAACUUCGUGACCACCUACAGGUCCAAG<br>AAAACCGUGGGCAACAUCAAGAUGCCUGGGGUGCACGCCGUGG<br>ACCACAGACUGGAAAGAAUCGAGGAAAGCGACAACGAGACAUAC<br>GUGGUGCAGCGCGAAGUGGCCGUGGCCAAGUACUCUAAUCUCG<br>GAGGCGGCAUGGACGAGCUGUACAAAGGAUCUCUCCAGCUGCC<br>ACCUCUGGAAAGACUGACCCUGCAGGCAGGCUACCCUUACGACG<br>UGCCAGAUUACGCCUGAGGUACCCUUGUACAGCUCGUCCAUGC<br>CGAGAGUGAUCCCGGCGGCGGUCACGAACUCCAGCAGGACCAUG<br>UGAUCGCGCUUCUCGUUGGGGUCUUUGCUCAGGGCGGACUGG<br>GUGCUCAGGUAGUGGUUGUCGGGCAGCAGCACGGGGCCGUCGC<br>CGAUGGGGGUGUUCUGCUGGUAGUGGUCGGCGAGCUGCACGC<br>UGCCGUCCUCGAUGUUGUGGCGGAUCUUGAAGUUCACCUUGA<br>UGCCGUUCUUCUGCUUGUCGGCCAUGAUAUAGACGUUGUGGC<br>UGUUGUAGUUGUACUCCAGCUUGUGCCCCAGGAUGUUGCCGU<br>CCUCCUUGAAGUCGAUGCCCUUCAGCUCGAUGCGGUUCACCAG<br>GGUGUCGCCCUCGAACUUCACCUCGGCGCGGGUCUUGUAGUUG<br>CCGUCGUCCUUGAAGAAGAUGGUGCGCUCCUGGACGUAGCCUU<br>CGGGCAUGGCGGACUUGAAGAAGUCGUGCUGCUUCAUGUGGU<br>CGGGGUAGCGGCUGAAGCACUGCACGCCGUAGGUCAGGGUGGU<br>CACGAGGGUGGGCCAGGGCACGGGCAGCUUGCCGGUGGUGCAG<br>AUGAACUUCAGGGUCAGCUUGCCGUAGGUGGCAUCGCCCUCGC<br>CCUCGCCGGACACGCUGAACUUGUGGCCGUUUACGUCGCCGUC<br>CAGCUCGACCAGGAUGGGCACCACCCCGGUGAACAGCUCCUCGC<br>CCUUGCUCACCAUUAAGGUACCUCUGGUGGGUAUAGAUUCUGU<br>GUAAAAUUGAUUCCAAUGCCAUCCACUUGAUAGGCACUUUGCC<br>UCCUUCUGCAUGGUAUUCUUUCUCUUCCGCACCCAGCAGUUUG<br>GCCAGCCCAAAAUCUGUGAUCUUGACAUGCUGCGGUGUUUUCA<br>CCAGUACGUUCCUGGCUGCCAGGUCGCGGUGCACCAAGCGACG<br>GUCCUCCAAGUAGUUCAUGCCCUUUGCGAUCUGCACACACCAG<br>UUGAGCAGGUACUGGGAGCCAAUAUUGUCUUUGUGUUCCCGG<br>ACAUAGUCCAGGAGGCAGCCGAAGGGCAUGAGCUGCGUGAUGA<br>GUUGCACGGUGGAGGUGAGGCAGAUGCCCAGCAGGCGGCACAC<br>GUGGGGGUUGUCCACGCUGGCCAUCACGUAGGCUUCAUCGAGG<br>AUUUCCUUGUUGGCUUUCGGAGAUGUUGCUUCUCUUAAUUCC<br>UUGAUAGCGACGGGAAUUUUAACUUUCUCACCUUCUGGGAUCC<br>AGAGUCCCUUAUACACCGUGCCGAACGCACCGGAGCCCAGCACU<br>UUGAUCUUUUUGAAUUCAGUUUCCUUCAAGAUCCUCAAGAGA<br>GCUUGGUUGGGAGCUUCUCCACUGGGUGUAAGAGGCUCCACAA<br>GCUCCCUCUCCUGCAGCAGCCUCCGCAGCGUGCGCUUCCGAACG<br>AUGUGGCGCCUUCGCAUGAAGAGGCCGAUCCCCAGGGCCACCAC<br>CAGCAGCAAGAGGAGGGCCCCCACCAUCCCAGUGGCGAUGGACG

		GGAUCUUAGGCCCAUUCGUUGGACAGCCUUCAAGACCUGGCCC<br>AGUGCAUCCGUAGGUGCAGUUUGGAUGGCACAGGUGGCACACA<br>UGGCCGGCGUCUGCGUACUUCCAGACCAGGGUGUUGUUUUCUC<br>CCAUGACUCCUGCCGGGCAGGUCUUGACGCAGUGGGGGCCGUC<br>AAUGUAGUGGGCACACUGGAUACAGUUGUCUGGUCCCCGUCCU<br>GUGCAGGUGAUGUUCAUGGCCUGAGGCAGGCACUCUGGGUGG<br>CACUGUAUGCACUCAGAGUUCUCCACAAACUCCCUUGGCUCACC<br>CUCCAGAAGGUUGCACUUGUCCACGCAUUCCCUGCCUCGGCUG<br>ACAUUCCGGCAAGAGACGCAGUCCCUGGGCUCCGGGCCCCAGCA<br>GCCCUCGGGGGAGCACAAGGCAUGGCAGACCUGGCCUGUGGCC<br>UUGCAGCUGUUUUCACCUCUGUUGCUUAUAAUUUUGGUUUUC<br>UGACCGGAGGUCCCAAACAGUUUUUUCCAGUUUAUUGUAUUU<br>GCAUAGCACAAAUUUUUGUUUCCUGAAAUUAUCACAUCUCCAU<br>CACUUAUCUCCUUGAGGGAGCGUAAUCCCAAGGAUGUUAUGUU<br>CAGGCUGACGACUGCAAGAGAAAACUGACCAUGUUGCUUGGUC<br>CUGCCGCGUAUGAUUUCUAGGUUCUCAAAGGCAUGGAGGUCCG<br>UCCUGUUUUCAGGCCAAGCCUGAAUCAGCAAAAACCCUGUGAU<br>UUCCUUUACGGUUUUCAGAAUAUCCAGUUCCUGUGGAUCCAGA<br>GGAGGAGUAUGUGUGAAGGAGUCACCCCUAAAUGCCACCGGCA<br>GGAUGUGGAGAUCGCCACUGAUGGAGGUGCAGUUUUUGAAGU<br>GUUUAAUAUUCGUAGCAUUUAUGGAGAGUGAGUCUUUAAAUU<br>CACCAAUACCUAUUCCGUUACACACUUUGCGGCAAGGCCCUUCG<br>CACUUCUUACACUUGCGGACGCCGUCUUCCUCCAUCUCAUAGCU<br>GUCGGCCCCACAGGCUCGGACGCACGAGCCGUGAUCUGUCACCA<br>CAUAAUUACGGGGACACUUCUUCACGCAGGUGGCACCAAAGCU<br>GUAUUUGCCCUCGGGGUUCACAUCCAUCUGGUACGUGGUGGG<br>GUUGUAGAGCAUGAGUGGGGGGCAGGUGUCCUUGCACGUGGC<br>UUCGUCUCGGAAUUUGCGGCAGACCAGGCAGUCGCUCUCCCGG<br>GGGCCUGUGCAGCCUGCAGCACACUGGUUGUGGCAGCAGUCAC<br>UGGGGGACUUGGUCGACCCUCCGUGUUGGAGAGCUGGUACCA<br>GGGCUGCUUGCCGUAGGUGAAGAUCUCCCAGAGCACCACGCCG<br>AAGCUCCACACGUCGCUCUCGGUGGUGAACUUACGGUACAGGA<br>UGCUCUCGGGCGGCAUCCAGCGAAUGGGCAGCAUGGUGCGGCC<br>UCCCACACGGUAAUAGUCGGUGCUGUAGAUAUCCCUGCUCAUG<br>CCAAAAUCACCAAUCUUGACCACCAGUCCCUGGCCCACUAGACA<br>GUUGCGUGUGGCCAGGUCCCGGUGCACAAAAUGCAGACCCGCC<br>AGGUACACCAUCCCCGCAGCGACCUGGCUAGCCACGGCCAGCAG<br>CUGCCCCAGACCCAGGGGGCCUGGAGCCACAUCCUCCCCACCAGC<br>CAGCAGCUUGGCAUCAGGUCCAUGGGAUCGGAGGAAGCGGUUG<br>AGGUCCCCGUGCCGCAUAUACUCAAAGACCAUGAGCAGGGGGC<br>GGCCCUCGGUGCAGACGCCGAAGAAGCGCACGAUGUGCUGGUG<br>CUGCAGCAUGGUGAGCAGCUCAGCCUCACGCUGGAAGUCCUGC<br>CGAGCACUCUCGGACGCCUCCUUCAGUGCCUUGACAGCCACCAG<br>CAUCUUGUCCUGCUCAGGCAGGAGGUUGUGGCACUCAGCAAGG<br>AAGACCUUCCCAAAGGCGCCCUCCCCCAGCUCCCACUUGAGCACG<br>AUGUCCCGGCGCUUGAUGUGGUGAACACAGGCAUCACUGAAGU<br>AUUGUGGGUUCUCGAUGAUGUGGCCUUGGAGCCCAGAGCCUU<br>UGCCCUCGGUGGGGGACAGGGAGCUGCCACCCAAUGUCAUGAA<br>AUGCAGGGACAUGGCCAGCCCAUCCUCUGGAGCCAGCACAGCCG<br>GGCGGUUGAUCCCAAACUUGUUUCUCCGUCCACAUUUGUUGAG<br>CACAAGGAGCAGCGUAGAAAGGAAGAGGCAGGCAAAGACGGCC<br>AGGCCCACAGCCACCGAGACCCCAAAAGGUGUUUCGUCCUUCUU<br>CUCCACCGGGUCUCCAGAUGUGCUGUUAGUGUCCACCGGCGAG

		AAGGAGACAGGGAUGGGGUCCUCGGGGUUGAACUCGAAAGGG<br>UUGUCCAUGAAGGCAGCCAUGAUGGAGGCGGAGGCCUGGCCGA<br>AGGGGUUGGCAGCCAGCAGCGUGUAGUUGCCGUUGUUGACGU<br>GGGUGGGCUGGUUGAGGCGCAGACACCCGUGCCGCACGGUCUC<br>AUUGGCUGCCGGCUCCAGGAACUCAGUGAAGAUGAAGCUGGUC<br>UCAUUGAGCACGGAGCCAUUGAAGAGCCAGCGCAGAGACGGUG<br>CCGGCUGCCCAUCCACAGAGAAGGGGAUGCACCAGUGGUGCAUC<br>UCCACCGCCGUGUGCAGCUGCACACUGGCCGGGAAGGAGACGU<br>UGACCUGAACAGAGACCUCUGCCCGGCCCACAUCGUUCUCUGCC<br>CAGCACGUCACGUUCUUCCUGUUGAGGUCACUGGUGACAUUGG<br>CCAGGGUCAGCCCCAGGGAUGGCAGACCCCCAGAUUUCAUCACC<br>GUGGCUGACUGCUCCAGCUCUGUGAGGAUCCAGCCGGCCUGCU<br>CCAGGCCCCGCCCCUCCACCUGGCACCGCAGCAGCACGUCGUCCC<br>CCACAUCCACCGAGGCAUUGGGCACCUGGACCUUCAGCGUGGGC<br>ACACCACAGCUGGCAUUGGGCAUGUGGGCCAGGGGCCCUUGCC<br>CAUGACACUGCAGCUUCUGUUCAGGCACUCCGCCCAGUCCCUCC<br>UCCUCCCAGCGCUGUAGCCAGCGCAGGGCACAAGAACAGUGCAG<br>AGGGUUCCCCGACAGGACCAGUUCCUGUAAGGAGAGGCCCUGC<br>ACAGUUUUCCAGGAGAGAGACUCCAGAGCGUUGAAGGAGAGAU<br>UCAGGCGACUGAGCCGAGGAGUGAAAUGGAAGGCAUCUGGCGC<br>CACGAAACGGAGACCACUCUUCACGAUGGUGAGGUUUCUCAGC<br>UCCCCCAGGCCCCUCAGAUCACGGAGCUCCAGAUGCUGCAGAUG<br>CUGCUGGUUCUCGAUGUAGAGCUCAGUCAGGUUCUCUGCGCCG<br>GGACCGGUUGAACGCGUGAAUUCCGACCAGAAGAUAUGGCUUC<br>GGUUGGGUUCAAA

mRuby3-Fillerup8000nt-PP7	mRuby3, RNA size 8000nt, PP7	AUGGGAUCCGUGUCCAAGGGCGAAGAACUGAUCAAAGAAAACA<br>UGCGGAUGAAGGUGGUCAUGGAAGGCAGCGUGAACGGCCACCA<br>GUUCAAGUGUACAGGCGAAGGCGAGGGCAGACCUUAUGAGGGC<br>GUGCAGACCAUGAGAAUCAAAGUGAUCGAAGGCGGCCCUCUGC<br>CUUUCGCUUUCGACAUCCUGGCCACCAGCUUUAUGUACGGCAG<br>CAGAACCUUCAUCAAGUACCCCGCUGACAUCCCCGAUUUCUUCA<br>AGCAGAGCUUCCCCGAGGGCUUCACCUGGGAGAGAGUGACCAG<br>AUACGAGGAUGGCGGCGUGGUCACAGUGACCCAGGACACAUCU<br>CUGGAAGAUGGCGAGCUGGUGUACAACGUGAAAGUGCGGGGC<br>GUGAACUUCCCCUCUAACGGCCCUGUGAUGCAGAAAAAGACCAA<br>AGGCUGGGAGCCCAACACCGAGAUGAUGUACCCUGCUGAUGGC<br>GGCCUGAGAGGCUACACAGAUAUCGCCCUGAAAGUGGACGGCG<br>GAGGCCAUCUGCACUGCAACUUCGUGACCACCUACAGGUCCAAG<br>AAAACCGUGGGCAACAUCAAGAUGCCUGGGGUGCACGCCGUGG<br>ACCACAGACUGGAAAGAAUCGAGGAAAGCGACAACGAGACAUAC<br>GUGGUGCAGCGCGAAGUGGCCGUGGCCAAGUACUCUAAUCUCG<br>GAGGCGGCAUGGACGAGCUGUACAAAGGAUCUCUCCAGCUGCC<br>ACCUCUGGAAAGACUGACCCUGCAGGCAGGCUACCCUUACGACG<br>UGCCAGAUUACGCCUGAGGUACCCUUGUACAGCUCGUCCAUGC<br>CGAGAGUGAUCCCGGCGGCGGUCACGAACUCCAGCAGGACCAUG<br>UGAUCGCGCUUCUCGUUGGGGUCUUUGCUCAGGGCGGACUGG<br>GUGCUCAGGUAGUGGUUGUCGGGCAGCAGCACGGGGCCGUCGC<br>CGAUGGGGGUGUUCUGCUGGUAGUGGUCGGCGAGCUGCACGC<br>UGCCGUCCUCGAUGUUGUGGCGGAUCUUGAAGUUCACCUUGA<br>UGCCGUUCUUCUGCUUGUCGGCCAUGAUAUAGACGUUGUGGC<br>UGUUGUAGUUGUACUCCAGCUUGUGCCCCAGGAUGUUGCCGU<br>CCUCCUUGAAGUCGAUGCCCUUCAGCUCGAUGCGGUUCACCAG<br>GGUGUCGCCCUCGAACUUCACCUCGGCGCGGGUCUUGUAGUUG<br>CCGUCGUCCUUGAAGAAGAUGGUGCGCUCCUGGACGUAGCCUU<br>CGGGCAUGGCGGACUUGAAGAAGUCGUGCUGCUUCAUGUGGU<br>CGGGGUAGCGGCUGAAGCACUGCACGCCGUAGGUCAGGGUGGU<br>CACGAGGGUGGGCCAGGGCACGGGCAGCUUGCCGGUGGUGCAG<br>AUGAACUUCAGGGUCAGCUUGCCGUAGGUGGCAUCGCCCUCGC<br>CCUCGCCGGACACGCUGAACUUGUGGCCGUUUACGUCGCCGUC<br>CAGCUCGACCAGGAUGGGCACCACCCCGGUGAACAGCUCCUCGC<br>CCUUGCUCACCAUUAAGGUACCUCUGGUGGGUAUAGAUUCUGU<br>GUAAAAUUGAUUCCAAUGCCAUCCACUUGAUAGGCACUUUGCC<br>UCCUUCUGCAUGGUAUUCUUUCUCUUCCGCACCCAGCAGUUUG<br>GCCAGCCCAAAAUCUGUGAUCUUGACAUGCUGCGGUGUUUUCA<br>CCAGUACGUUCCUGGCUGCCAGGUCGCGGUGCACCAAGCGACG<br>GUCCUCCAAGUAGUUCAUGCCCUUUGCGAUCUGCACACACCAG<br>UUGAGCAGGUACUGGGAGCCAAUAUUGUCUUUGUGUUCCCGG<br>ACAUAGUCCAGGAGGCAGCCGAAGGGCAUGAGCUGCGUGAUGA<br>GUUGCACGGUGGAGGUGAGGCAGAUGCCCAGCAGGCGGCACAC<br>GUGGGGGUUGUCCACGCUGGCCAUCACGUAGGCUUCAUCGAGG<br>AUUUCCUUGUUGGCUUUCGGAGAUGUUGCUUCUCUUAAUUCC<br>UUGAUAGCGACGGGAAUUUUAACUUUCUCACCUUCUGGGAUCC<br>AGAGUCCCUUAUACACCGUGCCGAACGCACCGGAGCCCAGCACU<br>UUGAUCUUUUUGAAUUCAGUUUCCUUCAAGAUCCUCAAGAGA<br>GCUUGGUUGGGAGCUUCUCCACUGGGUGUAAGAGGCUCCACAA<br>GCUCCCUCUCCUGCAGCAGCCUCCGCAGCGUGCGCUUCCGAACG<br>AUGUGGCGCCUUCGCAUGAAGAGGCCGAUCCCCAGGGCCACCAC<br>CAGCAGCAAGAGGAGGGCCCCCACCAUCCCAGUGGCGAUGGACG

		GGAUCUUAGGCCCAUUCGUUGGACAGCCUUCAAGACCUGGCCC<br>AGUGCAUCCGUAGGUGCAGUUUGGAUGGCACAGGUGGCACACA<br>UGGCCGGCGUCUGCGUACUUCCAGACCAGGGUGUUGUUUUCUC<br>CCAUGACUCCUGCCGGGCAGGUCUUGACGCAGUGGGGGCCGUC<br>AAUGUAGUGGGCACACUGGAUACAGUUGUCUGGUCCCCGUCCU<br>GUGCAGGUGAUGUUCAUGGCCUGAGGCAGGCACUCUGGGUGG<br>CACUGUAUGCACUCAGAGUUCUCCACAAACUCCCUUGGCUCACC<br>CUCCAGAAGGUUGCACUUGUCCACGCAUUCCCUGCCUCGGCUG<br>ACAUUCCGGCAAGAGACGCAGUCCCUGGGCUCCGGGCCCCAGCA<br>GCCCUCGGGGGAGCACAAGGCAUGGCAGACCUGGCCUGUGGCC<br>UUGCAGCUGUUUUCACCUCUGUUGCUUAUAAUUUUGGUUUUC<br>UGACCGGAGGUCCCAAACAGUUUUUUCCAGUUUAUUGUAUUU<br>GCAUAGCACAAAUUUUUGUUUCCUGAAAUUAUCACAUCUCCAU<br>CACUUAUCUCCUUGAGGGAGCGUAAUCCCAAGGAUGUUAUGUU<br>CAGGCUGACGACUGCAAGAGAAAACUGACCAUGUUGCUUGGUC<br>CUGCCGCGUAUGAUUUCUAGGUUCUCAAAGGCAUGGAGGUCCG<br>UCCUGUUUUCAGGCCAAGCCUGAAUCAGCAAAAACCCUGUGAU<br>UUCCUUUACGGUUUUCAGAAUAUCCAGUUCCUGUGGAUCCAGA<br>GGAGGAGUAUGUGUGAAGGAGUCACCCCUAAAUGCCACCGGCA<br>GGAUGUGGAGAUCGCCACUGAUGGAGGUGCAGUUUUUGAAGU<br>GUUUAAUAUUCGUAGCAUUUAUGGAGAGUGAGUCUUUAAAUU<br>CACCAAUACCUAUUCCGUUACACACUUUGCGGCAAGGCCCUUCG<br>CACUUCUUACACUUGCGGACGCCGUCUUCCUCCAUCUCAUAGCU<br>GUCGGCCCCACAGGCUCGGACGCACGAGCCGUGAUCUGUCACCA<br>CAUAAUUACGGGGACACUUCUUCACGCAGGUGGCACCAAAGCU<br>GUAUUUGCCCUCGGGGUUCACAUCCAUCUGGUACGUGGUGGG<br>GUUGUAGAGCAUGAGUGGGGGGCAGGUGUCCUUGCACGUGGC<br>UUCGUCUCGGAAUUUGCGGCAGACCAGGCAGUCGCUCUCCCGG<br>GGGCCUGUGCAGCCUGCAGCACACUGGUUGUGGCAGCAGUCAC<br>UGGGGGACUUGGUCGACCCUCCGUGUUGGAGAGCUGGUACCA<br>GGGCUGCUUGCCGUAGGUGAAGAUCUCCCAGAGCACCACGCCG<br>AAGCUCCACACGUCGCUCUCGGUGGUGAACUUACGGUACAGGA<br>UGCUCUCGGGCGGCAUCCAGCGAAUGGGCAGCAUGGUGCGGCC<br>UCCCACACGGUAAUAGUCGGUGCUGUAGAUAUCCCUGCUCAUG<br>CCAAAAUCACCAAUCUUGACCACCAGUCCCUGGCCCACUAGACA<br>GUUGCGUGUGGCCAGGUCCCGGUGCACAAAAUGCAGACCCGCC<br>AGGUACACCAUCCCCGCAGCGACCUGGCUAGCCACGGCCAGCAG<br>CUGCCCCAGACCCAGGGGGCCUGGAGCCACAUCCUCCCCACCAGC<br>CAGCAGCUUGGCAUCAGGUCCAUGGGAUCGGAGGAAGCGGUUG<br>AGGUCCCCGUGCCGCAUAUACUCAAAGACCAUGAGCAGGGGGC<br>GGCCCUCGGUGCAGACGCCGAAGAAGCGCACGAUGUGCUGGUG<br>CUGCAGCAUGGUGAGCAGCUCAGCCUCACGCUGGAAGUCCUGC<br>CGAGCACUCUCGGACGCCUCCUUCAGUGCCUUGACAGCCACCAG<br>CAUCUUGUCCUGCUCAGGCAGGAGGUUGUGGCACUCAGCAAGG<br>AAGACCUUCCCAAAGGCGCCCUCCCCCAGCUCCCACUUGAGCACG<br>AUGUCCCGGCGCUUGAUGUGGUGAACACAGGCAUCACUGAAGU<br>AUUGUGGGUUCUCGAUGAUGUGGCCUUGGAGCCCAGAGCCUU<br>UGCCCUCGGUGGGGGACAGGGAGCUGCCACCCAAUGUCAUGAA<br>AUGCAGGGACAUGGCCAGCCCAUCCUCUGGAGCCAGCACAGCCG<br>GGCGGUUGAUCCCAAACUUGUUUCUCCGUCCACAUUUGUUGAG<br>CACAAGGAGCAGCGUAGAAAGGAAGAGGCAGGCAAAGACGGCC<br>AGGCCCACAGCCACCGAGACCCCAAAAGGUGUUUCGUCCUUCUU<br>CUCCACCGGGUCUCCAGAUGUGCUGUUAGUGUCCACCGGCGAG

		AAGGAGACAGGGAUGGGGUCCUCGGGGUUGAACUCGAAAGGG<br>UUGUCCAUGAAGGCAGCCAUGAUGGAGGCGGAGGCCUGGCCGA<br>AGGGGUUGGCAGCCAGCAGCGUGUAGUUGCCGUUGUUGACGU<br>GGGUGGGCUGGUUGAGGCGCAGACACCCGUGCCGCACGGUCUC<br>AUUGGCUGCCGGCUCCAGGAACUCAGUGAAGAUGAAGCUGGUC<br>UCAUUGAGCACGGAGCCAUUGAAGAGCCAGCGCAGAGACGGUG<br>CCGGCUGCCCAUCCACAGAGAAGGGGAUGCACCAGUGGUGCAUC<br>UCCACCGCCGUGUGCAGCUGCACACUGGCCGGGAAGGAGACGU<br>UGACCUGAACAGAGACCUCUGCCCGGCCCACAUCGUUCUCUGCC<br>CAGCACGUCACGUUCUUCCUGUUGAGGUCACUGGUGACAUUGG<br>CCAGGGUCAGCCCCAGGGAUGGCAGACCCCCAGAUUUCAUCACC<br>GUGGCUGACUGCUCCAGCUCUGUGAGGAUCCAGCCGGCCUGCU<br>CCAGGCCCCGCCCCUCCACCUGGCACCGCAGCAGCACGUCGUCCC<br>CCACAUCCACCGAGGCAUUGGGCACCUGGACCUUCAGCGUGGGC<br>ACACCACAGCUGGCAUUGGGCAUGUGGGCCAGGGGCCCUUGCC<br>CAUGACACUGCAGCUUCUGUUCAGGCACUCCGCCCAGUCCCUCC<br>UCCUCCCAGCGCUGUAGCCAGCGCAGGGCACAAGAACAGUGCAG<br>AGGGUUCCCCGACAGGACCAGUUCCUGUAAGGAGAGGCCCUGC<br>ACAGUUUUCCAGGAGAGAGACUCCAGAGCGUUGAAGGAGAGAU<br>UCAGGCGACUGAGCCGAGGAGUGAAAUGGAAGGCAUCUGGCGC<br>CACGAAACGGAGACCACUCUUCACGAUGGUGAGGUUUCUCAGC<br>UCCCCCAGGCCCCUCAGAUCACGGAGCUCCAGAUGCUGCAGAUG<br>CUGCUGGUUCUCGAUGUAGAGCUCAGUCAGGUUCUCUGCGCCG<br>GGACCGGUGUCCAGCAUGGGCACAUAGUCCACCGACUCGUCCU<br>UGCUCAUGUCCAUGUAGCCACCGUCGCUCUCCCCGGUCAAGGAC<br>ACAUGGCUGGGCAGGGGGAGCCCAACGGGCAGAGCAUUGCUGU<br>AGAGCUCCGCGCUGGGCGGGCGGCGCUUGUCGGAGUGGUGCU<br>GCAGGAAGGUGUGUUUGUUGCGGUGCAGGUAGUCCACCAGGU<br>CUCCGUAGCGGCAGUACUCAGUGAUGAUAUAGAUGGGUCCUCC<br>UUUGGUGCAGGCCCCCAACAGGUUGACCACGUUCAGGUGGGGC<br>CCAAGGUGACUCAUGAUCUUCAGCUCCGACAUAAGGGCUUGCU<br>UCUCACUGCUGCGGGCUGUGGAUUUAAGCAUCUUGACGGCCAC<br>UUUCAUCGUGGCCUGAGAAUGGCUCAGGCCAUGAGCCGUGGCC<br>UCCACCACCUGCCCAAAGGCCCCAGAGCCGAGGGUGCGUCCCAG<br>CACAAGCUGGUCCCGCGGCAGCUCCCACGUGGAGUCAUAGGGCA<br>GCUGCAUGGGGUCCACGUAGAUGUACUCAUGGCCGUCAGAGCU<br>CACAGACUCAAUCACCUUCCAUCGGAUCUCGUAACGUGGCUUCU<br>UCUGCCAAAGCAUGAUGAGGAUGAUAAGGGAGAUGAUGGUGA<br>GCACCACCAGGGCCAGGAUGGCUGAGAUCACCACCACCUUAAAG<br>GGCAAGGAGUGUGGCACCACGAUGACCUCCUGCGUGUCCUGGC<br>CCACAGCGUUGCGCAGCGUGCAGCGCACCGACAGUGGCCGAUCC<br>ACGUGCUGCAGACGCAGUGUGCUCACCACCUCAAACUCCUGCUC<br>CUCCUCCCAGUACGUCACGUUAGUCUCCAGCUGGCUCUCCUCUU<br>CGGAACUGUUCCCCAGCAGCGUGGGCGGCAGCUCACGUGGACA<br>CCUUUUGAGGUCUCUGCAGGCAGACCAGAUGAUGUUCGGCUGG<br>GGCAUGCCCCGGCCACGACAGCGGACUGUCUGUUCCCCACUGUC<br>AGGGUGGCUCUCACUUAGCUCCAGCACUCGGACAGGGACAUUG<br>AUCUGUAGCUGGAAGGAGAGCUGGACCUCAGCAUCCUCAUGGA<br>AGGCCCGCAUGGUGUAGUGGCCAGCCUCUGCCACCUUCACGCGA<br>ACCAGUGUCAGCUCUGACACAUACCGGGUCUCCGACACGUUGCG<br>CGUGGACAGGGCGAUUUCGCCAGCGCUGGAGUCGCCCAGGGUG<br>CGGUUGUCUUUGAACCACAGGACAGUGGGCGGUGGGUAGGCC<br>UCGAACACUACCUGCAGUGUCCGGCUCCGAUGCAGCUCAGCAAA

		UUGUAGUGUGCCCACCUCUCCCAGGAGCCGCACGUAGCCGCUCUCAACCACGGUGAUGUUGAUGGCCUUUUCAUCCUGAUGGUCAUUCACACUCUCCGUCACAUUGCAGGUGUAGGUCCCCGAGUCUUCUAACUCGGCACUGGGGAUGUGCAGGAUGGAGCGGAUGUGGUAAGGCAUAUCCAAGAGGAAGUCAGUCACCGGCUCCACCAGCCGCCCACUUUCUUUGCGGGGGUAUGUCCACUCGAAGUUGACCACCUCAUUCCCGAUCACAAUGCACAUGAGGGUGAUGUUCUCACCCUGGCGGACCACAGUCUGCACUGCGUUCACAGAGACGUUGAUGGAUGACACCUGGAGUCUGUAGACAUAGUAGGCAUCAGAAUCCACCUCCCUGUCCCCAAUGGUGGUUUUGCAGAUGUAGCUUCUGUCCUCAAAGAUACCACUAAAGCCACGUUGGUGAUCAUAGGGGACAGGCAGUGCAACGUCCCCUUUCUUCUCGUGCAGUGUCACCACCAGCUGUGGGUCUGUUACUCGGCAUGGAAUGGUGAUCUCAGUUAUUUCCGUGAGAAAGAUGAAUAGUUCCUCGGCAUCAUUAGGGAGGAAGCCCACGGUGGGAUCUGGCACAAAGAUGUAGAGCCGUUUCCGCUCAUCGGUCUCCAGUCCACGGGAGUCAUUGUGGGUGCAAAAGUAUUCUCCCGUGUCUAGCCCAGUGAGGUUGGUCAGUGUGAGCACGCUGGAGAAGGUGCCAUCCUGGGCCUUGGCUGAACGCGUGAAUUCCGACCAGAAGAUAUGGCUUCGGUUGGGUUCAAA

mRuby3-Fillerup10000nt-PP7	mRuby3, RNA size 10000nt, PP7	AUGGGAUCCGUGUCCAAGGGCGAAGAACUGAUCAAAGAAAACA<br>UGCGGAUGAAGGUGGUCAUGGAAGGCAGCGUGAACGGCCACCA<br>GUUCAAGUGUACAGGCGAAGGCGAGGGCAGACCUUAUGAGGGC<br>GUGCAGACCAUGAGAAUCAAAGUGAUCGAAGGCGGCCCUCUGC<br>CUUUCGCUUUCGACAUCCUGGCCACCAGCUUUAUGUACGGCAG<br>CAGAACCUUCAUCAAGUACCCCGCUGACAUCCCCGAUUUCUUCA<br>AGCAGAGCUUCCCCGAGGGCUUCACCUGGGAGAGAGUGACCAG<br>AUACGAGGAUGGCGGCGUGGUCACAGUGACCCAGGACACAUCU<br>CUGGAAGAUGGCGAGCUGGUGUACAACGUGAAAGUGCGGGGC<br>GUGAACUUCCCCUCUAACGGCCCUGUGAUGCAGAAAAAGACCAA<br>AGGCUGGGAGCCCAACACCGAGAUGAUGUACCCUGCUGAUGGC<br>GGCCUGAGAGGCUACACAGAUAUCGCCCUGAAAGUGGACGGCG<br>GAGGCCAUCUGCACUGCAACUUCGUGACCACCUACAGGUCCAAG<br>AAAACCGUGGGCAACAUCAAGAUGCCUGGGGUGCACGCCGUGG<br>ACCACAGACUGGAAAGAAUCGAGGAAAGCGACAACGAGACAUAC<br>GUGGUGCAGCGCGAAGUGGCCGUGGCCAAGUACUCUAAUCUCG<br>GAGGCGGCAUGGACGAGCUGUACAAAGGAUCUCUCCAGCUGCC<br>ACCUCUGGAAAGACUGACCCUGCAGGCAGGCUACCCUUACGACG<br>UGCCAGAUUACGCCUGAGGUACCCUUGUACAGCUCGUCCAUGC<br>CGAGAGUGAUCCCGGCGGCGGUCACGAACUCCAGCAGGACCAUG<br>UGAUCGCGCUUCUCGUUGGGGUCUUUGCUCAGGGCGGACUGG<br>GUGCUCAGGUAGUGGUUGUCGGGCAGCAGCACGGGGCCGUCGC<br>CGAUGGGGGUGUUCUGCUGGUAGUGGUCGGCGAGCUGCACGC<br>UGCCGUCCUCGAUGUUGUGGCGGAUCUUGAAGUUCACCUUGA<br>UGCCGUUCUUCUGCUUGUCGGCCAUGAUAUAGACGUUGUGGC<br>UGUUGUAGUUGUACUCCAGCUUGUGCCCCAGGAUGUUGCCGU<br>CCUCCUUGAAGUCGAUGCCCUUCAGCUCGAUGCGGUUCACCAG<br>GGUGUCGCCCUCGAACUUCACCUCGGCGCGGUCUUGUAGUUG<br>CCGUCGUCCUUGAAGAAGAUGGUGCGCUCCUGGACGUAGCCUU<br>CGGGCAUGGCGGACUUGAAGAAGUCGUGCUGCUUCAUGUGGU<br>CGGGGUAGCGGCUGAAGCACUGCACGCCGUAGGUCAGGGUGGU<br>CACGAGGGUGGGCCAGGGCACGGGCAGCUUGCCGGUGGUGCAG<br>AUGAACUUCAGGGUCAGCUUGCCGUAGGUGGCAUCGCCCUCGC<br>CCUCGCCGGACACGCUGAACUUGUGGCCGUUUACGUCGCCGUC<br>CAGCUCGACCAGGAUGGGCACCACCCCGGUGAACAGCUCCUCGC<br>CCUUGCUCACCAUUAAGGUACCUCUGGUGGGUAUAGAUUCUGU<br>GUAAAAUUGAUUCCAAUGCCAUCCACUUGAUAGGCACUUUGCC<br>UCCUUCUGCAUGGUAUUCUUUCUCUUCCGCACCCAGCAGUUUG<br>GCCAGCCCAAAAUCUGUGAUCUUGACAUGCUGCGGUGUUUUCA<br>CCAGUACGUUCCUGGCUGCCAGGUCGCGGUGCACCAAGCGACG<br>GUCCUCCAAGUAGUUCAUGCCCUUUGCGAUCUGCACACACCAG<br>UUGAGCAGGUACUGGGAGCCAAUAUUGUCUUUGUGUUCCCGG<br>ACAUAGUCCAGGAGGCAGCCGAAGGGCAUGAGCUGCGUGAUGA<br>GUUGCACGGUGGAGGUGAGGCAGAUGCCCAGCAGGCGGCACAC<br>GUGGGGGUUGUCCACGCUGGCCAUCACGUAGGCUUCAUCGAGG<br>AUUUCCUUGUUGGCUUUCGGAGAUGUUGCUUCUCUUAAUUCC<br>UUGAUAGCGACGGGAAUUUUAACUUUCUCACCUUCUGGGAUCC<br>AGAGUCCCUUAUACACCGUGCCGAACGCACCGGAGCCCAGCACU<br>UUGAUCUUUUUGAAUUCAGUUUCCUUCAAGAUCCUCAAGAGA<br>GCUUGGUUGGGAGCUUCUCCACUGGGUGUAAGAGGCUCCACAA<br>GCUCCCUCUCCUGCAGCAGCCUCCGCAGCGUGCGCUUCCGAACG<br>AUGUGGCGCCUUCGCAUGAAGAGGCCGAUCCCCAGGGCCACCAC<br>CAGCAGCAAGAGGAGGGCCCCCACCAUCCCAGUGGCGAUGGACG

		GGAUCUUAGGCCCAUUCGUUGGACAGCCUUCAAGACCUGGCCC AGUGCAUCCGUAGGUGCAGUUUGGAUGGCACAGGUGGCACACA UGGCCGGCGUCUGCGUACUUCCAGACCAGGGUGUUGUUUUCUC CCAUGACUCCUGCCGGGCAGGUCUUGACGCAGUGGGGGCCGUC AAUGUAGUGGGCACACUGGAUACAGUUGUCUGGUCCCCGUCCU GUGCAGGUGAUGUUCAUGGCCUGAGGCAGGCACUCUGGGUGG CACUGUAUGCACUCAGAGUUCUCCACAAACUCCCUUGGCUCACC CUCCAGAAGGUUGCACUUGUCCACGCAUUCCCUGCCUCGGCUG ACAUUCCGGCAAGAGACGCAGUCCCUGGGCUCCGGGCCCCAGCA GCCCUCGGGGGAGCACAAGGCAUGGCAGACCUGGCCUGUGGCC UUGCAGCUGUUUUCACCUCUGUUGCUUAUAAUUUUGGUUUUC UGACCGGAGGUCCCAAACAGUUUUUUCCAGUUUAUUGUAUUU GCAUAGCACAAAUUUUUGUUUCCUGAAAUUAUCACAUCUCCAU CACUUAUCUCCUUGAGGGAGCGUAAUCCCAAGGAUGUUAUGUU CAGGCUGACGACUGCAAGAGAAAACUGACCAUGUUGCUUGGUC CUGCCGCGUAUGAUUUCUAGGUUCUCAAAGGCAUGGAGGUCCG UCCUGUUUUCAGGCCAAGCCUGAAUCAGCAAAAACCCUGUGAU UUCCUUUACGGUUUUCAGAAUAUCCAGUUCCUGUGGAUCCAGA GGAGGAGUAUGUGUGAAGGAGUCACCCCUAAAUGCCACCGGCA GGAUGUGGAGAUCGCCACUGAUGGAGGUGCAGUUUUUGAAGU GUUUAAUAUUCGUAGCAUUUAUGGAGAGUGAGUCUUUAAAUU CACCAAUACCUAUUCCGUUACACACUUUGCGGCAAGGCCCUUCG CACUUCUUACACUUGCGGACGCCGUCUUCCUCCAUCUCAUAGCU GUCGGCCCCACAGGCUCGGACGCACGAGCCGUGAUCUGUCACCA CAUAAUUACGGGGACACUUCUUCACGCAGGUGGCACCAAAGCU GUAUUUGCCCUCGGGGUUCACAUCCAUCUGGUACGUGGUGGG GUUGUAGAGCAUGAGUGGGGGGCAGGUGUCCUUGCACGUGGC UUCGUCUCGGAAUUUGCGGCAGACCAGGCAGUCGCUCUCCCGG GGGCCUGUGCAGCCUGCAGCACACUGGUUGUGGCAGCAGUCAC UGGGGGACUUGGUCGACCCUCCGUGUUGGAGAGCUGGUACCA GGGCUGCUUGCCGUAGGUGAAGAUCUCCCAGAGCACCACGCCG AAGCUCCACACGUCGCUCUCGGUGGUGAACUUACGGUACAGGA UGCUCUCGGGCGGCAUCCAGCGAAUGGGCAGCAUGGUGCGGCC UCCCACACGGUAAUAGUCGGUGCUGUAGAUAUCCCUGCUCAUG CCAAAAUCACCAAUCUUGACCACCAGUCCCUGGCCCACUAGACA GUUGCGUGUGGCCAGGUCCCGGUGCACAAAAUGCAGACCCGCC AGGUACACCAUCCCCGCAGCGACCUGGCUAGCCACGGCCAGCAG CUGCCCCAGACCCAGGGGGCCUGGAGCCACAUCCUCCCCACCAGC CAGCAGCUUGGCAUCAGGUCCAUGGGAUCGGAGGAAGCGGUUG AGGUCCCCGUGCCGCAUAUACUCAAAGACCAUGAGCAGGGGGC GGCCCUCGGUGCAGACGCCGAAGAAGCGCACGAUGUGCUGGUG CUGCAGCAUGGUGAGCAGCUCAGCCUCACGCUGGAAGUCCUGC CGAGCACUCUCGGACGCCUCCUUCAGUGCCUUGACAGCCACCAG CAUCUUGUCCUGCUCAGGCAGGAGGUUGUGGCACUCAGCAAGG AAGACCUUCCCAAAGGCGCCCUCCCCCAGCUCCCACUUGAGCACG AUGUCCCGGCGCUUGAUGUGGUGAACACAGGCAUCACUGAAGU AUUGUGGGUUCUCGAUGAUGUGGCCUUGGAGCCCAGAGCCUU UGCCCUCGGUGGGGGACAGGGAGCUGCCACCCAAUGUCAUGAA AUGCAGGGACAUGGCCAGCCCAUCCUCUGGAGCCAGCACAGCCG GGCGGUUGAUCCCAAACUUGUUUCUCCGUCCACAUUUGUUGAG CACAAGGAGCAGCGUAGAAAGGAAGAGGCAGGCAAAGACGGCC AGGCCCACAGCCACCGAGACCCCAAAAGGUGUUUCGUCCUUCUU CUCCACCGGGUCUCCAGAUGUGCUGUUAGUGUCCACCGGCGAG

		AAGGAGACAGGGAUGGGGUCCUCGGGGUUGAACUCGAAAGGG<br>UUGUCCAUGAAGGCAGCCAUGAUGGAGGCGGAGGCCUGGCCGA<br>AGGGGUUGGCAGCCAGCAGCGUGUAGUUGCCGUUGUUGACGU<br>GGGUGGGCUGGUUGAGGCGCAGACACCCGUGCCGCACGGUCUC<br>AUUGGCUGCCGGCUCCAGGAACUCAGUGAAGAUGAAGCUGGUC<br>UCAUUGAGCACGGAGCCAUUGAAGAGCCAGCGCAGAGACGGUG<br>CCGGCUGCCCAUCCACAGAGAAGGGGAUGCACCAGUGGUGCAUC<br>UCCACCGCCGUGUGCAGCUGCACACUGGCCGGGAAGGAGACGU<br>UGACCUGAACAGAGACCUCUGCCCGGCCCACAUCGUUCUCUGCC<br>CAGCACGUCACGUUCUUCCUGUUGAGGUCACUGGUGACAUUGG<br>CCAGGGUCAGCCCCAGGGAUGGCAGACCCCCAGAUUUCAUCACC<br>GUGGCUGACUGCUCCAGCUCUGUGAGGAUCCAGCCGGCCUGCU<br>CCAGGCCCCGCCCCUCCACCUGGCACCGCAGCAGCACGUCGUCCC<br>CCACAUCCACCGAGGCAUUGGGCACCUGGACCUUCAGCGUGGGC<br>ACACCACAGCUGGCAUUGGGCAUGUGGGCCAGGGGCCCUUGCC<br>CAUGACACUGCAGCUUCUGUUCAGGCACUCCGCCCAGUCCCUCC<br>UCCUCCCAGCGCUGUAGCCAGCGCAGGGCACAAGAACAGUGCAG<br>AGGGUUCCCCGACAGGACCAGUUCCUGUAAGGAGAGGCCCUGC<br>ACAGUUUUCCAGGAGAGAGACUCCAGAGCGUUGAAGGAGAGAU<br>UCAGGCGACUGAGCCGAGGAGUGAAAUGGAAGGCAUCUGGCGC<br>CACGAAACGGAGACCACUCUUCACGAUGGUGAGGUUUCUCAGC<br>UCCCCCAGGCCCCUCAGAUCACGGAGCUCCAGAUGCUGCAGAUG<br>CUGCUGGUUCUCGAUGUAGAGCUCAGUCAGGUUCUCUGCGCCG<br>GGACCGGUGUCCAGCAUGGGCACAUAGUCCACCGACUCGUCCU<br>UGCUCAUGUCCAUGUAGCCACCGUCGCUCUCCCCGGUCAAGGAC<br>ACAUGGCUGGGCAGGGGGAGCCCAACGGGCAGAGCAUUGCUGU<br>AGAGCUCCGCGCUGGGCGGGCGGCGCUUGUCGGAGUGGUGCU<br>GCAGGAAGGUGUGUUUGUUGCGGUGCAGGUAGUCCACCAGGU<br>CUCCGUAGCGGCAGUACUCAGUGAUGAUAUAGAUGGGUCCUCC<br>UUUGGUGCAGGCCCCCAACAGGUUGACCACGUUCAGGUGGGGC<br>CCAAGGUGACUCAUGAUCUUCAGCUCCGACAUAAGGGCUUGCU<br>UCUCACUGCUGCGGGCUGUGGAUUUAAGCAUCUUGACGGCCAC<br>UUUCAUCGUGGCCUGAGAAUGGCUCAGGCCAUGAGCCGUGGCC<br>UCCACCACCUGCCCAAAGGCCCCAGAGCCGAGGGUGCGUCCCAG<br>CACAAGCUGGUCCCGCGGCAGCUCCCACGUGGAGUCAUAGGGCA<br>GCUGCAUGGGGUCCACGUAGAUGUACUCAUGGCCGUCAGAGCU<br>CACAGACUCAAUCACCUUCCAUCGGAUCUCGUAACGUGGCUUCU<br>UCUGCCAAAGCAUGAUGAGGAUGAUAAGGGAGAUGAUGGUGA<br>GCACCACCAGGGCCAGGAUGGCUGAGAUCACCACCACCUUAAAG<br>GGCAAGGAGUGUGGCACCACGAUGACCUCCUGCGUGUCCUGGC<br>CCACAGCGUUGCGCAGCGUGCAGCGCACCGACAGUGGCCGAUCC<br>ACGUGCUGCAGACGCAGUGUGCUCACCACCUCAAACUCCUGCUC<br>CUCCUCCCAGUACGUCACGUUAGUCUCCAGCUGGCUCUCCUCUU<br>CGGAACUGUUCCCCAGCAGCGUGGGCGGCAGCUCACGUGGACA<br>CCUUUUGAGGUCUCUGCAGGCAGACCAGAUGAUGUUCGGCUGG<br>GGCAUGCCCCGGCCACGACAGCGGACUGUCUGUUCCCCACUGUC<br>AGGGUGGCUCUCACUUAGCUCCAGCACUCGGACAGGGACAUUG<br>AUCUGUAGCUGGAAGGAGAGCUGGACCUCAGCAUCCUCAUGGA<br>AGGCCCGCAUGGUGUAGUGGCCAGCCUCUGCCACCUUCACGCGA<br>ACCAGUGUCAGCUCUGACACAUACCGGGUCUCCGACACGUUGCG<br>CGUGGACAGGGCGAUUUCGCCAGCGCUGGAGUCGCCCAGGGUG<br>CGGUUGUCUUUGAACCACAGGACAGUGGGCGGUGGGUAGGCC<br>UCGAACACUACCUGCAGUGUCCGGCUCCGAUGCAGCUCAGCAAA

		UUGUAGUGUGCCCACCUCUCCCAGGAGCCGCACGUAGCCGCUCU<br>CAACCACGGUGAUGUUGAUGGCCUUUUCAUCCUGAUGGUCAUU<br>CACACUCUCCGUCACAUUGCAGGUGUAGGUCCCCGAGUCUUCU<br>AACUCGGCACUGGGGAUGUGCAGGAUGGAGCGGAUGUGGUAA<br>GGCAUAUCCAAGAGGAAGUCAGUCACCGGCUCCACCAGCCGCCC<br>ACUUUCUUUGCGGGGGUAUGUCCACUCGAAGUUGACCACCUCA<br>UUCCCGAUCACAAUGCACAUGAGGGUGAUGUUCUCACCCUGGC<br>GGACCACAGUCUGCACUGCGUUCACAGAGACGUUGAUGGAUGA<br>CACCUGGAGUCUGUAGACAUAGUAGGCAUCAGAAUCCACCUCCC<br>UGUCCCCAAUGGUGGUUUUGCAGAUGUAGCUUCUGUCCUCAAA<br>GAUACCACUAAAGCCACGUUGGUGAUCAUAGGGGACAGGCAGU<br>GCAACGUCCCCUUUCUUCUCGUGCAGUGUCACCACCAGCUGUG<br>GGUCUGUUACUCGGCAUGGAAUGGUGAUCUCAGUUAUUUCCG<br>UGAGAAAGAUGAAUAGUUCCUCGGCAUCAUUAGGGAGGAAGCC<br>CACGGUGGGAUCUGGCACAAAGAUGUAGAGCCGUUUCCGCUCA<br>UCGGUCUCCAGUCCACGGGAGUCAUUGUGGGUGCAAAAGUAUU<br>CUCCCGUGUCUAGCCCAGUGAGGUUGGUCAGUGUGAGCACGCU<br>GGAGAAGGUGCCAUCCUGGGCCUUGGCUGACGAUCGAUCGGAU<br>GCAUUUACACUCGAGUCAGUCAGUCAACGUAUCACCUGGUGCU<br>CCAGGUGCCCAUGCGGCCCCCUCCUCCCCAGGCUGGGUCUGGCC<br>CAUCGGGAUUAUCCAUGUUCUCAUAAGAGUCUGCAUCUUCCUC<br>AUGAUUGGGUCCAGGCUGGCCCCGAAUGGAGCGGAGCUGGGG<br>GGCUGCAUACAGGAUUCCUCUCAUAUCCUCAUAGGACUGGGAC<br>CCCAGGGAGGUUGCUUCCCGGCUGGGGUCCCAGGCUGACCCAU<br>GAGGGCUCAGGAAGUCCAUUGUCCUGGCGACCGGCUGGGUCAG<br>CUCUUCAUCCUCGUUCUCAUAAGACUCAGCGUUGGAGAAGGAG<br>UCUUCAUCCUCAGGACCCAGGGGCUCAUCCUCAGGGUUCUCGU<br>AGCCGCUGCCAUCCUGGGAGAGCUGGUCCUGCCCAAGGUUGGA<br>GUCGUUCUCAUAGAACUCGGAGUCCUCCUCACUGUCAGGUUCC<br>UCAUAGCCCUCCCCUUCCUCUUCUUCUGGGCCCACUCCCGGCGG<br>GCUCCGGGACCCCAAGGCUCCAUCCGCCUGGACGUCGCUGCUCG<br>GGUUUCCAUAAGACGGGGCAGUGCCCCCAGGCCUGCGGCCCAA<br>CGCUGGGCGCGUCCGAGGCCUGAGGUGGGUGUGGGGAGAGAC<br>AGCACGUUCCCGUACUGGUUCUGGGGCCCGCUUCCUGGGGGAG<br>GCGUCACUUUGAAGAAUCUCCUGGUGGGGUCAGUCAUUCGCUU<br>UCUUUUCCUCCUCAGGACCAGGGCUCUUUGAAGAUGAAGAAUG<br>CCCACAAGGGAACACAGGCAGAAGAUCAGAUAAGCCAAAGUCAC<br>AGCUGAGACCUUCCAGCCACCAGUCCUCAGCAGCCAGUGCCAUA<br>GUACUGGCCGAGCAGUGAUCUCCAGGUGGAAUGACAUGGUCAG<br>GUUGCCACGGUGACAAUAAUACUUUCCAGCGUCUUGAGCUGUG<br>GCCCGGGGCAACAACAGACCCGUCUCCAUUACCCACAUAUCUCU<br>GGCCGGGCGAUCGUCCUUCAGCUCUAGGCUCAGCAAUGACUUA<br>GGCCCCUUGGGGUGCACAUGGGUCCAGGAGAGGGGGCCCCUGG<br>ACACAGAGUCAGGGGGUACCCCACAGGACAGCCAGAGUGUGGA<br>GCCAGGGGCCAUGGUGAGGUCCUGGCUGAGGCUCUGGUUCAG<br>GCUGUCCCUCGGUGGGAGACACGGAGGCUCUCCCUCCCAGAUC<br>UCAGGGCGGUCUUUGGCCCACACAUACAGCUUGGGGCUCAUGA<br>GCUUCCCGGAAGGGGAGCUGGGGCCCUCUGAGGACCUGUUCUU<br>CAGGCCACAGCCCAGGCCACCUAGGUCCGAAACAUUCCACCGGA<br>ACAGCUCCCCGCUGCCCUCCACAUUGACUGUCCAGCCAGGCUGC<br>CAGGCCUUCUCAGAGGGGGGCCCCGGCUGGCACAGGUAGAAGC<br>CCCCCAUCUGUUGAGAGACGUUGAAGAUGAAAAGCCAGAUGGC<br>CAGGGGCCUCAUGUGGAUUCCCAGGCCUGGCAGCCCCAGGCUG

		AGUUUUAAGAAGGGUUUAAGCGGGGACUCCCGAGACCAGGUCA<br>GCUGCUGAGUGGGGCCAUCUGAGGUCCCCUUGAGGCACUGCAG<br>CACAGCGUUAUCUCCCUCUUCCACCUUCACCACUAGAGGUUCCU<br>CGGGCCUGACUUCCAUGGGGGUGAGGAAGAGGAGGAAGAAGA<br>GGAGGCGAGGAGGUGGCAUAAGCUUUUAAACGCGUGAAUUCC<br>GACCAGAAGAUAUGGCUUCGGUUGGGUUCAAA
Wildtype VSV-G	Wildtype Vesicular Stomatitis Virus (VSV) Glycoprotein (VSV-G)	MKCLLYLAFLFIGVNCKFTIVFPHNQKGNWKNVPSNYHYCPSSSDLN<br>WHNDLIGTALQVKMPKSHKAIQADGWMCHASKWVTTCDFRWYGP<br>KYITHSIRSFTPSVEQCKESIEQTKQGTWLNPGFPPQSCGYATVTDAEA<br>VIVQVTPHHVLVDEYTGEWVDSQFINGKCSNYICPTVHNSTTWHSDY<br>KVKGLCDSNLISMDITFFSEDGELSSLGKEGTGFRSNYFAYETGGKACK<br>MQYCKHWGVRLPSGVWFEMADKDLFAAARFPECPEGSSISAPSQTS<br>VDVSLIQDVERILDYSLCQETWSKIRAGLPISPVDLSYLAPKNPGTGPAF<br>TIINGTLKYFETRYIRVDIAAPILSRMVGMISGTTTERELWDDWAPYED<br>VEIGPNGVLRTSSGYKFPLYMIGHGMLDSDLHLSSKAQVFEHPHIQDA<br>ASQLPDDESLFFGDTGLSKNPIELVEGWFSSWKSSIASFFFIIGLIIGLFLV<br>LRVGIHLCIKLKHTKKRQIYTDIEMNRLGK

exemplary AAVS1 donor	Used for stable integration of EGFR and IL7Ra in HEK293T (Red: 5' and 3' Homology Arms; Dark Blue: CAG Promoter; Green: **Start-to-stop codon for gene-of-interest**; Yellow: Internal Ribosomal Entry Site (IRES); Light Blue: Puromycin Resistance Gene	CTTTGAGCTCTACTGGCTTCTGCGCCGCCTCTGGCCCACTGTTTCCC<br>CTTCCCAGGCAGGTCCTGCTTTCTCTGACCTGCATTCTCTCCCCTGG<br>GCCTGTGCCGCTTTCTGTCTGCAGCTTGTGGCCTGGGTCACCTCTAC<br>GGCTGGCCCAGATCCTTCCCTGCCGCCTCCTTCAGGTTCCGTCTTCC<br>TCCACTCCCTCTTCCCCTTGCTCTCTGCTGTGTTGCTGCCCAAGGATG<br>CTCTTTCCGGAGCACTTCCTTCTCGGCGCTGCACCACGTGATGTCCT<br>CTGAGCGGATCCTCCCCGTGTCTGGGTCCTCTCCGGGCATCTCTCCT<br>CCCTCACCCAACCCCATGCCGTCTTCACTCGCTGGGTTCCCTTTTCCT<br>TCTCCTTCTGGGGCCTGTGCCATCTCTCGTTTCTTAGGATGGCCTTCT<br>CCGACGGATGTCTCCCTTGCGTCCCGCCTCCCCTTCTTGTAGGCCTG<br>CATCATCACCGTTTTTCTGGACAACCCCAAAGTACCCCGTCTCCCTG<br>GCTTTAGCCACCTCTCCATCCTCTTGCTTTCTTTGCCTGGACACCCCG<br>TTCTCCTGTGGATTCGGGTCACCTCTCACTCCTTTCATTTGGGCAGC<br>TCCCCTACCCCCCTTACCTCTCTAGTCTGTGCTAGCTCTTCCAGCCCC<br>CTGTCATGGCATCTTCCAGGGGTCCGAGAGCTCAGCTAGTCTTCTTC<br>CTCCAACCCGGGCCCCTATGTCCACTTCAGGACAGCATGTTTGCTGC<br>CTCCAGGGATCCTGTGTCCCCGAGCTGGGACCACCTTATATTCGCG<br>GCCCGCGCCGGATTCGACATTGATTATTGACTAGTTATTAATAGTA<br>ATCAATTACGGGGTCATTAGTTCATAGCCCATATATGGAGTTCCGC<br>GTTACATAACTTACGGTAAATGGCCCGCCTGGCTGACCGCCCAACG<br>ACCCCCGCCCATTGACGTCAATAATGACGTATGTTCCCATAGTAACG<br>CCAATAGGGACTTTCCATTGACGTCAATGGGTGGAGTATTTACGGT<br>AAACTGCCCACTTGGCAGTACATCAAGTGTATCATATGCCAAGTAC<br>GCCCCCTATTGACGTCAATGACGGTAAATGGCCCGCCTGGCATTAT<br>GCCCAGTACATGACCTTATGGGACTTTCCTACTTGGCAGTACATCTA<br>CGTATTAGTCATCGCTATTACCATGGTCGAGGTGAGCCCCACGTTCT<br>GCTTCACTCTCCCCATCTCCCCCCCCTCCCCACCCCCAATTTTGTATTT<br>ATTTATTTTTTAATTATTTTGTGCAGCGATGGGGGCGGGGGGGGGG<br>GGGGGGCGCGCCAGGCGGGGCGGGGCGGGGCGAGGGGCGG<br>GGCGGGGCGAGGCGGAGAGGTGCGGCGGCAGCCAATCAGAGCG<br>GCGCGCTCCGAAAGTTTCCTTTTATGGCGAGGCGGCGGCGGC<br>GGCCCTATAAAAAGCGAAGCGCGCGGCGGGCGGGAGTCGCTGCG<br>TCGCGCCTTCGCCCCGTGCCCCGCTCCGCCGCCGCCTCGCGCCGCC<br>CGCCCCGGCTCTGACTGACCGCGTTACTCCCACAGGTGAGCGGGC<br>GGGACGGCCCTTCTCCTCCGGGCTGTAATTAGCGCTTGGTTTAATG<br>ACGGCTCGTTTCTTTTCTGTGGCTGCGTGAAAGCCTTAAAGGGCTC<br>CGGGAGGGCCCTTTGTGCGGGGGGGAGCGGCTCGGGGGGTGCGT<br>GCGTGTGTGTGTGCGTGGGGAGCGCCGCGTGCGGCCCGCGCTGCC<br>CGGCGGCTGTGAGCGCTGCGGGCGCGGCGCGGGGCTTTGTGCGCT<br>CCGCGTGTGCGCGAGGGGAGCGCGGCCGGGGGCGGTGCCCCGCG<br>GTGCGGGGGGGCTGCGAGGGGAACAAAGGCTGCGTGCGGGGTGT<br>GTGCGTGGGGGGGTGAGCAGGGGGTGTGGGCGCGGCGGTCGGG<br>CTGTAACCCCCCCCTGCACCCCCCTCCCCGAGTTGCTGAGCACGGCC<br>CGGCTTCGGGTGCGGGGCTCCGTGCGGGGCGTGGCGCGGGGCTC<br>GCCGTGCCGGGCGGGGGGTGGCGGCAGGTGGGGGTGCCGGGCG<br>GGGCGGGGCCGCCTCGGGCCGGGGAGGGCTCGGGGGAGGGGCG<br>CGGCGGCCCCGGAGCGCCGGCGGCTGTCGAGGCGCGGCGAGCCG<br>CAGCCATTGCCTTTTATGGTAATCGTGCGAGAGGGCGCAGGGACTT<br>CCTTTGTCCCAAATCTGGCGGAGCCGAAATCTGGGAGGCGCCGCC<br>GCACCCCCTCTAGCGGGCGCGGGCGAAGCGGTGCGGCGCCGGCA<br>GGAAGGAAATGGGCGGGGAGGGCCTTCGTGCGTCGCCGCGCCGC<br>CGTCCCCTTCTCCATCTCCAGCCTCGGGGCTGCCGCAGGGGGACGG<br>CTGCCTTCGGGGGGGACGGGGCAGGGCGGGGTTCGGCTTCTGGC

		GTGTGACCGGCGGCTCTAGAGCCTCTGCTAACCATGTTCATGCCTT<br>CTTCTTTTTCCTACAGATCCTTAATTAAGCCGCCACC**ATGNNNNNN**<br>**NNNTAA**ACGCGTTGTACACCCCCCTCTCCCTCCCCCCCCCCTAACGT<br>TACTGGCCGAAGCCGCTTGGAATAAGGCCGGTGTGCGTTTGTCTAT<br>ATGTTATTTTCCACCATATTGCCGTCTTTTGGCAATGTGAGGGCCCG<br>GAAACCTGGCCCTGTCTTCTTGACGAGCATTCCTAGGGGTCTTTCCC<br>CTCTCGCCAAAGGAATGCAAGGTCTGTTGAATGTCGTGAAGGAAG<br>CAGTTCCTCTGGAAGCTTCTTGAAGACAAACAACGTCTGTAGCGAC<br>CCTTTGCAGGCAGCGGAACCCCCCACCTGGCGACAGGTGCCTCTGC<br>GGCCAAAAGCCACGTGTATAAGATACACCTGCAAAGGCGGCACAA<br>CCCCAGTGCCACGTTGTGAGTTGGATAGTTGTGGAAAGAGTCAAAT<br>GGCTCTCCTCAAGCGTATTCAACAAGGGGCTGAAGGATGCCCAGA<br>AGGTACCCCATTGTATGGGATCTGATCTGGGGCCTCGGTGCACATG<br>CTTTACATGTGTTTAGTCGAGGTTAAAAAACGTCTAGGCCCCCCGA<br>ACCACGGGGACGTGGTTTTCCTTTGAAAAACACGATGATAATATGG<br>CCACCACCATGACCGAGTACAAGCCCACAGTGCGGCTGGCCACCA<br>GGGACGATGTGCCAAGAGCTGTGCGGACACTGGCCGCTGCCTTCG<br>CCGATTACCCTGCCACAAGACACACCGTGGACCCCGACCGGCACAT<br>CGAGAGAGTGACCGAGCTGCAGGAACTGTTTCTGACCAGAGTGGG<br>CCTGGACATCGGCAAAGTGTGGGTGGCCGATGATGGCGCCGCTGT<br>GGCTGTGTGGACAACCCCTGAGTCTGTGGAAGCCGGCGCTGTGTT<br>CGCCGAGATCGGACCTAGAATGGCCGAGCTGAGCGGCTCTAGACT<br>GGCTGCCCAGCAGCAGATGGAAGGCCTGCTGGCCCCCCACAGACC<br>AAAAGAGCCTGCCTGGTTTCTGGCCACCGTGGGCGTGTCACCTGAC<br>CACCAGGGCAAGGGACTGGGATCTGCTGTGGTGCTGCCTGGGGTG<br>GAAGCTGCTGAAAGGGCTGGCGTGCCCGCCTTCCTGGAAACAAGC<br>GCCCCCAGAAACCTGCCCTTCTACGAGAGACTGGGCTTCACCGTGA<br>CCGCCGACGTGGAAGTGCCTGAGGGCCCTAGAACCTGGTGCATGA<br>CCAGAAAGCCTGGCGCCTGAATAACTTCGTATAATGTATGCTATAC<br>GAAGTTATGGCGCGCCAAATGATTGCAGATCCACTAGTTCTAGAGC<br>TCGCTGATCAGCCTCGACTGTGCCTTCTAGTTGCCAGCCATCTGTTG<br>TTTGCCCCTCCCCCGTGCCTTCCTTGACCCTGGAAGGTGCCACTCCC<br>ACTGTCCTTTCCTAATAAAATGAGGAAATTGCATCGCATTGTCTGAG<br>TAGGTGTCATTCTATTCTGGGGGGTGGGGTGGGGCAGGACAGCAA<br>GGGGGAGGATTGGGAAGACAATAGCAGGCATGCTGGGGATGCGG<br>TGGGCTCTATGGCTTCTGAGGCGGAAAGAACCAGCTGGGGCTCGA<br>GATCCACTAGTTCTAGCCTCGAGGCTAGAGCGGCCGGCCGCCCAG<br>GGCCGGTTAATGTGGCTCTGGTTCTGGGTACTTTTATCTGTCCCCTC<br>CACCCCACAGTGGGGCCACTAGGGACAGGATTGGTGACAGAAAAG<br>CCCCATCCTTAGGCCTCCTCCTTCCTAGTCTCCTGATATTGGGTCTAA<br>CCCCCACCTCCTGTTAGGCAGATTCCTTATCTGGTGACACACCCCCA<br>TTTCCTGGAGCCATCTCTCTCCTTGCCAGAACCTCTAAGGTTTGCTT<br>ACGATGGAGCCAGAGAGGATCCTGGGAGGGAGAGCTTGGCAGGG<br>GGTGGGAGGGAAGGGGGGGATGCGTGACCTGCCCGGTTCTCAGT<br>GGCCACCCTGCGCTACCCTCTCCCAGAACCTGAGCTGCTCTGACGC<br>GGCCGTCTGGTGCGTTTCACTGATCCTGGTGCTGCAGCTTCCTTACA<br>CTTCCCAAGAGGAGAAGCAGTTTGGAAAAACAAAATCAGAATAAG<br>TTGGTCCTGAGTTCTAACTTTGGCTCTTCACCTTTCTAGTCCCCAATT<br>TATATTGTTCCTCCGTGCGTCAGTTTTACCTGTGAGATAAGGCCAGT<br>AGCCAGCCCCGTCCTGGCAGGGCTGTGGTGAGGAGGGGGGTGTC<br>CGTGTGGAAAACTCCCTTTGTGAGAATGGTGCGTCCTAGGTGTTCA<br>CCAGGTCGTGGCCGCCTCTACTCCCTTTCTCTTTCTCCATCCTTCTTT<br>CCTTAAAGAGTCCCCAGTGCTATCTGGGACATATTCCTCCGCCCAGA

		GCAGGGTCCCGCTTCCCTAAGGCCCTGCTCTGGGCTTCTGGGTTTG AGTCCTTGGC

References

1 Schwinn, M. K. *et al.* CRISPR-Mediated Tagging of Endogenous Proteins with a Luminescent Peptide. *ACS Chem Biol* **13**, 467-474, doi:10.1021/acschembio.7b00549 (2018).

2 Plaper, T. *et al.* Coiled-coil heterodimers with increased stability for cellular regulation and sensing SARS-CoV-2 spike protein-mediated cell fusion. *Sci Rep* **11**, 9136, doi:10.1038/s41598-021-88315-3 (2021).

3 Carvajal-Vallejos, P., Pallisse, R., Mootz, H. D. & Schmidt, S. R. Unprecedented rates and efficiencies revealed for new natural split inteins from metagenomic sources. *J Biol Chem* **287**, 28686-28696, doi:10.1074/jbc.M112.372680 (2012).
