# Supplementary material for: Creating bottom-up RNA transfer vehicles from synthetic protein assemblies
Source: Nature. 2026 Sep 2;658(8136):805–13. doi: 10.1038/s41586-026-10952-3 (PMC13668671; doi:10.1038/s41586-026-10952-3)
Supplement: Supplementary file 2 [file 41586_2026_10952_MOESM2_ESM.pdf]

nature portfolio

Corresponding author(s): Christoph Gruber, Florian Giesert, Wolfgang Wurst

Last updated by author(s): Jul 9, 2026

Reporting Summary

Nature Portfolio wishes to improve the reproducibility of the work that we publish. This form provides structure for consistency and transparency in reporting. For further information on Nature Portfolio policies, see our Editorial Policies and the Editorial Policy Checklist.

Statistics

For all statistical analyses, confirm that the following items are present in the figure legend, table legend, main text, or Methods section.

n/a	Confirmed	
☐	☒	The exact sample size (*n*) for each experimental group/condition, given as a discrete number and unit of measurement
☐	☒	A statement on whether measurements were taken from distinct samples or whether the same sample was measured repeatedly
☐	☒	The statistical test(s) used AND whether they are one- or two-sided<br>*Only common tests should be described solely by name; describe more complex techniques in the Methods section.*
☒	☐	A description of all covariates tested
☒	☐	A description of any assumptions or corrections, such as tests of normality and adjustment for multiple comparisons
☐	☒	A full description of the statistical parameters including central tendency (e.g. means) or other basic estimates (e.g. regression coefficient) AND variation (e.g. standard deviation) or associated estimates of uncertainty (e.g. confidence intervals)
☐	☒	For null hypothesis testing, the test statistic (e.g. *F*, *t*, *r*) with confidence intervals, effect sizes, degrees of freedom and *P* value noted<br>*Give P values as exact values whenever suitable.*
☒	☐	For Bayesian analysis, information on the choice of priors and Markov chain Monte Carlo settings
☒	☐	For hierarchical and complex designs, identification of the appropriate level for tests and full reporting of outcomes
☒	☐	Estimates of effect sizes (e.g. Cohen's *d*, Pearson's *r*), indicating how they were calculated

Our web collection on statistics for biologists contains articles on many of the points above.

Software and code

Policy information about availability of computer code

Data collection	VILBER Fusion FX, Berthold Centro LB 960, EVOS Cell Imaging, CellInsight NTX High Content Analysis, IncuCyte S3 Live-Cell Analysis System (Sartorius), TitanG4 (ThermoFisher), BD FACSaria III (BD Bioscience), Axio Imager M2 fluorescence microscope (Carl Zeiss), Fusion SL Vilber machine (Peqlab). RNA sequencing library preparation and Illumina paired-end sequencing was performed by GENEWIZ (Leipzig, Germany). Mass spectrometry was performed by Helmholtz Munich core facility (Munich, Germany).
Data analysis	Software used: Geneious Prime (2025.1.2), BD FACSDiva Software (Version 6.1.3, BD Biosciences), Fiji, Microsoft Excel, IncuCyte S3, FlowJo V10. Frequency, mean, and standard deviations were calculated using GraphPad Prism.

For manuscripts utilizing custom algorithms or software that are central to the research but not yet described in published literature, software must be made available to editors and reviewers. We strongly encourage code deposition in a community repository (e.g. GitHub). See the Nature Portfolio guidelines for submitting code & software for further information.

Data

Policy information about availability of data

All manuscripts must include a data availability statement. This statement should provide the following information, where applicable:

- Accession codes, unique identifiers, or web links for publicly available datasets
- A description of any restrictions on data availability
- For clinical datasets or third party data, please ensure that the statement adheres to our policy

All data are available in the manuscript or supplementary information. The RNA-seq data generated in this study have been deposited in the NCBI Sequence Read Archive (SRA) under accession number PRJNA1489338 and in the NCBI Gene Expression Omnibus (GEO) under accession number GSE338002.

Human research participants

Policy information about studies involving human research participants and Sex and Gender in Research.

Reporting on sex and gender	No human research participants were involved.
Population characteristics	/
Recruitment	/
Ethics oversight	/

Note that full information on the approval of the study protocol must also be provided in the manuscript.

Field-specific reporting

Please select the one below that is the best fit for your research. If you are not sure, read the appropriate sections before making your selection.

☒ Life sciences ☐ Behavioural & social sciences ☐ Ecological, evolutionary & environmental sciences

For a reference copy of the document with all sections, see nature.com/documents/nr-reporting-summary-flat.pdf

Life sciences study design

All studies must disclose on these points even when the disclosure is negative.

Sample size	Experiments were performed in 96 well format with 30,000 cells per well, in 24 well format with 220,000 cells per well, in 6 well plates with 750,000 cells per well or in 10cm dishes with 4,000 000 cells per dish. No formal statistical method was used to predetermine sample size. Sample sizes were selected based on previous experience with the experimental models, established practice in the field, and ethical considerations to use the minimum number of animals required to obtain robust and reproducible results. The chosen sample sizes were sufficient to detect biologically meaningful effects and to support the statistical analyses described in the Methods. Exact sample sizes are provided in the corresponding figure legends.
Data exclusions	No data were excluded.
Replication	Replicates were chosen based on expected variation based on prior experience, but at least in biological duplicates. Unless otherwise stated, immunofluorescence images and gel electrophoresis images shown throughout the manuscript are representative of at least three independent experiments that yielded comparable results. Whenever quantitative analyses are presented, the corresponding sample size (n) is indicated in the figure legends and/or within the figures.
Randomization	After seeding, experimental and control group were allocated randomly. For 96 well transfections the outer wells were not transfected.
Blinding	Blinding was not applicable for most in vitro and molecular experiments because the outcomes were based on objective quantitative measurements (e.g., immunoblotting, immunofluorescence, sequencing, and biochemical assays) analyzed using predefined criteria or standardized analysis pipelines, without subjective scoring. Samples from different experimental groups were processed in parallel under identical conditions to minimize technical bias. Where applicable, automated image analysis and quantitative data processing were used.

Reporting for specific materials, systems and methods

We require information from authors about some types of materials, experimental systems and methods used in many studies. Here, indicate whether each material, system or method listed is relevant to your study. If you are not sure if a list item applies to your research, read the appropriate section before selecting a response.

Materials & experimental systems

n/a	Involved in the study	
☐	☒	Antibodies
☐	☒	Eukaryotic cell lines
☒	☐	Palaeontology and archaeology
☐	☒	Animals and other organisms
☒	☐	Clinical data
☒	☐	Dual use research of concern

Methods

n/a	Involved in the study	
☒	☐	ChIP-seq
☐	☒	Flow cytometry
☒	☐	MRI-based neuroimaging

Antibodies

Antibodies used

All antibodies used in this study are commercially available and validated:
1. Mouse anti-HA primary antibody (Sigma-Aldrich, H3663, clone HA-7, ICC/WB: 1 µg/mL)
2. Chicken anti-GFP primary antibody (Abcam, ab13970, ICC/IF: 1:1000)
3. Rabbit anti-RPE65 primary antibody (Proteintech, 17939-1-AP, IF: 1:200)
4. Rat anti-GFP primary antibody (Santa Cruz Biotechnology, sc-101536, clone 1A5, ICC/IF: 1:100)
5. Rabbit Anti-Mash1 (ASCL1) primary antibody (Abcam, ab211327, clone EPR19840, IF: 1:100)
6. Donkey anti-mouse AlexaFluor488 secondary antibody (Thermo Fisher, A21202, ICC/IF: 10 µg/mL)
7. Donkey anti-chicken AlexaFluor488 secondary antibody (Dianova/Jackson ImmunoResearch, 703-546-155, ICC/IF: 1:200)
8. Donkey anti-rabbit AlexaFluor594 secondary antibody (Thermo Fisher, A21207, ICC/IF: 10 µg/mL)
9. Donkey anti-rat AlexaFluor555 secondary antibody (Abcam, ab150154, ICC/IF: 1:200)
10. Goat anti-rat AlexaFluor488 secondary antibody (Thermo Fisher A11006, ICC/IF: 10 µg/mL)

Validation

All antibodies used in this study have been validated by the manufacturer and used in various other studies. Manufacturer validation statements are available on the respective product webpages:
1. Anti-HA (Sigma-Aldrich, H3663) validated by the manufacturer for immunofluorescence and/or immunoblot applications and reactive with the HA epitope: https://www.sigmaaldrich.com/DE/de/product/sigma/h3663
2. Anti-GFP (Abcam, ab13970) validated by the manufacturer for immunofluorescence and immunoblotting and reactive with GFP: https://www.abcam.com/products/primary-antibodies/chicken-gfp-antibody-ab13970
3. Anti-RPE65 (Proteintech, 17939-1-AP) validated by the manufacturer for immunofluorescence and immunoblotting in human, mouse and rat samples: https://www.ptglab.com/products/RPE65-Antibody-17939-1-AP.htm
4. Anti-GFP (Santa Cruz Biotechnology, sc-101536) validated by the manufacturer for immunofluorescence and reactive with GFP: https://www.scbt.com/p/gfp-antibody-1a5-sc-101536
5. Anti-Mash1/ASCL1 (Abcam, ab211327) validated by the manufacturer for immunofluorescence and reactive with human and mouse ASCL1/Mash1: https://www.abcam.com/products/primary-antibodies/ascl1-mash1-antibody-epr19840-ab211327
The following highly cross-adsorbed secondary antibodies are validated by the manufacturers for species-specific detection with minimal cross-reactivity and were used according to the manufacturers' instructions:
6. Donkey anti-mouse AlexaFluor488: https://www.thermofisher.com/antibody/product/Alexa-Fluor-488-Donkey-anti-Mouse-IgG-H-L-Highly-Cross-Adsorbed-Secondary-Antibody-Polyclonal/A-21202
7. Donkey anti-chicken AlexaFluor488: https://www.jacksonimmuno.com/catalog/products/703-546-155
8. Donkey anti-rabbit AlexaFluor594: https://www.thermofisher.com/antibody/product/Alexa-Fluor-594-Donkey-anti-Rabbit-IgG-H-L-Highly-Cross-Adsorbed-Secondary-Antibody-Polyclonal/A-21207
9. Donkey anti-rat AlexaFluor555: https://www.abcam.com/products/secondary-antibodies/donkey-rat-igg-hl-alexa-fluor-555-ab150154
10. Goat anti-rat AlexaFluor488: https://www.thermofisher.com/antibody/product/Alexa-Fluor-488-Goat-anti-Rat-IgG-H-L-Cross-Adsorbed-Secondary-Antibody-Polyclonal/A-11006

Eukaryotic cell lines

Policy information about cell lines and Sex and Gender in Research

Cell line source(s)

HEK293T (a gift from the Institute of Developmental Genetics, Helmholtz Munich),
Vero E6, N2a, and HepG2 cells (a gift from the Institute of Virology, Helmholtz Munich)
A549-IFN-GFP cells (a gift from Ralf Bartenschlager)
C2C12 mouse myoblasts (a gift from the Institute of Developmental Genetics, Helmholtz Munich)

Authentication

The original cell lines were authenticated by the supplier.

Mycoplasma contamination

Cell lines were tested for mycoplasma contamination.

Commonly misidentified lines
(See ICLAC register)

No commonly misidentified cell lines were used.

Animals and other research organisms

Policy information about studies involving animals; ARRIVE guidelines recommended for reporting animal research, and Sex and Gender in Research

Laboratory animals	For probing potential immunological response and liver toxicity upon systemic STV-C8 injection, C57BL/6N mice with an age of 11 weeks have been used. For the analysis of mouse whole-body biodistribution of STV-C8 mediated EGFP expression, 4 week old Balb/c mice have been used. As a large animal model; Sus scrofa (German landrace pig) has been used at an age of 3 month.
Wild animals	no wild animals were used in the study
Reporting on sex	similar numbers of animals of both sex were used
Field-collected samples	No field collected samples were used in the study.
Ethics oversight	Ethics committee of the Regierung von Oberbayern

Note that full information on the approval of the study protocol must also be provided in the manuscript.

Flow Cytometry

Plots

Confirm that:

☒ The axis labels state the marker and fluorochrome used (e.g. CD4-FITC).

☒ The axis scales are clearly visible. Include numbers along axes only for bottom left plot of group (a 'group' is an analysis of identical markers).

☒ All plots are contour plots with outliers or pseudocolor plots.

☒ A numerical value for number of cells or percentage (with statistics) is provided.

Methodology

Sample preparation	Cells were prepared by detaching with Accutase and mixing with PBS, 5% (v/v) FBS, 2 mM EDTA. Subsequently, samples were processed through a cell-strainer cap and analyzed.
Instrument	BD FACSaria III
Software	BD FACSDiva Software (Version 6.1.3, BD Biosciences) & FlowJo 10.0
Cell population abundance	At least 10,000 single cells per condition and replicate were analyzed for each experiment.
Gating strategy	Living/single cell population was gated based on forward/side scatter and this population subsequently analyzed for fluorescence intensity

☒ Tick this box to confirm that a figure exemplifying the gating strategy is provided in the Supplementary Information.
