# Supplementary material for: Creating bottom-up RNA transfer vehicles from synthetic protein assemblies
Source: Nature. 2026 Sep 2;658(8136):805–13. doi: 10.1038/s41586-026-10952-3 (PMC13668671; doi:10.1038/s41586-026-10952-3)
Supplement: Supplementary file 3 [file 41586_2026_10952_MOESM3_ESM.pdf]

nature portfolio

Peer Review File

Creating bottom-up RNA transfer vehicles from synthetic protein assemblies

Corresponding Author: Professor Wolfgang Wurst

Any redactions in this file are there to maintain patient confidentiality, the confidentiality of unpublished data, or to remove third-party material.

This file contains all reviewer reports in order by version, followed by all author rebuttals in order by version.

Version 0:

Reviewer comments:

Referee #1

(Remarks to the Author)
This manuscript employs previously published AI-derived oligomeric domains, links them to previously established modules for RNA binding and membrane interaction, and then applies the resultant novel constructs for RNA transfer in a variety of cell-line and animal systems. It is likely that there are some favorable properties of these constructs. The constructs are benchmarked to lipid nanoparticle and other protein carrier systems, although it is not fully clear that the rival approaches are ideally assembled and so the key property of interest is an absolute level of delivery efficiency.

From a conceptual point of view, the text expresses surprise that AI-derived domains show activity, whereas I would suggest that the field is fully accustomed to the activity of AI-based proteins over the last 2 years. The achievement of the AI field is not just that such sequences have some activity but that they adopt desired assembly with nearly atomic precision. In the work here there is little evidence as to the adopted assembly of the novel constructs: the proposed organisation (e.g. Fig. 1a,b and extended Fig. 4a) is not well supported, with the only exploration being the tomographic analysis in Fig. 2e.

From a functional point of view, delivery of RNA to cells in culture has been well established through many methods, so the delivery in animals would have the higher importance for establishing novel performance of the approach. Here the data have important uncertainties. It would be important to show data from a mouse treated with negative control particles and imaged under the same conditions for EGFP delivery. Dystrophin exon deletion seems to have been assessed by PCR with flanking primers. PCR is known to preferentially amplify shorter inserts, i.e. the deleted version. Analyzing the amount of PCR products of different size either on a gel or by nanopore sequencing would be subject to the same potential amplification bias. Therefore further evidence would be required for confidence in the frequency of deletion and thereby the activity of the method. More robust assays of stable genetic change that avoid potential PCR amplification issues are now standard in the gene therapy field.
The work is described “as a treatment strategy for Duchenne Muscular Dystrophy” and yet the EGFP data seem to show little delivery to skeletal muscle from intravenous delivery.
I would suggest that these conceptual and practical uncertainties would need to be resolved before the manuscript is of broad interest for the protein design or genetic delivery communities.

Minor points:
At various points proteins structures are shown, without explanation of what these represent, i.e. an experimental structure, a schematic or a simulation.

Also Figure legends often need more detail for a reader to understand the constructs and the experiment shown.

Based on the dynamic light scattering, the particles seem substantially smaller than the particle shown from tomography.

Referee #2

(Remarks to the Author)
In this study, the authors use AI to design protein assemblies that combine natural and synthetic protein domains to create a large number of novel RNA transport vehicles. They show that these vehicles are able to transfer RNA much more efficiently than currently available vehicles and that their tropism can be programmed by incorporating computationally designed peptide binders that target specific cell-surface receptors. They use these modified vehicles to transfer cargo RNAs, including gene editors, to a range of cell-based systems. Finally, the authors show that these vehicles can be used in vivo to transfer RNAs to mice by IV injection and to perform gene editing in pigs by packaging CRISPR/Cas9 to delete an exon from the Duchenne muscular dystrophy gene.
The work is remarkable in many respects and shows that generative AI can be harnessed to engineer protein assemblies with potentially powerfully useful properties, including gene editing in vivo. In my view, the findings here are novel and highly significant. Despite my enthusiasm, there are some issues that should be addressed. Foremost among them is that the authors are very sparing in what they report. A basic tenant of scientific publishing is that enough detail should be provided so that others can reproduce the published work. In this case, this will be difficult. Below I elaborate on this and mention a few additional points, some rather minor.
1. Exactly how the authors performed their AI-based protein engineering is very difficult to glean from the information provided. I recognize that the main text may be constrained by space limitations, but supplemental material could describe in much more detail what was done.
2. The authors design their vehicles using well-known membrane-targeting domains, viral late domains (in tandem), and RNA binding motifs, but no sequences are provided in the material that I reviewed. In particular, what were the nature and sequences of linker domains used? Again, anyone wishing to verify these findings would need to start ab initio and would have no way of generating the exact vehicles used.
3. Extended Fig. 2 shows that VSV-G alone is highly efficient for RNA delivery. The authors should comment.
4. Extended Fig. 4e. It appears that the best vehicles simply release better. The authors should comment. Along these lines, have the authors attempted to measure what would be analogous to “specific infectivity” in a viral system; i.e., how efficiently a vehicle transfers RNA normalized for protein content? This could provide useful information regarding whether some vehicles are particularly efficient at delivering packaged RNA, other things (particle release, RNA packaging) being equal.
5. Very minor, line 188: “mitochondrial RNAs were depleted from STV-C8 particles” doesn’t quite make sense. I think the authors mean “excluded”. Similarly, line 952.
6. The authors report that their STV-C8 vehicles contain protein assemblies with a diameter of around 22 nm inside much larger vesicles (diameter 150-300 nm). I find this very intriguing. Generally, enveloped viruses contain capsids (or the equivalent) that are much more similar in diameter to the outer membrane; indeed, the protein component bends the membrane as it buds, essentially wrapping itself closely in the membrane. For example, budding retroviruses contain an immature Gag lattice that is only slightly smaller in diameter than the surrounding membrane. It’s not clear to me, as claimed on line 196, that the assemblies are acting similarly to natural capsid protomers in bending the membrane. Similarly, if the RNA is bound to the outer surface of the protein oligomers it might well interfere with membrane association (via charge repulsion). The authors should discuss this and also mention, a propos of this point, whether individual vesicles contain multiple protein/RNA assemblies.
7. Fig. 2f. Do the authors have any thoughts as to why the efficiency of EGFP expression is so variable with the different cell lines tested?
8. Fig. 2j. The fold change in EGFP expression with the different protein binders is not particularly impressive, around 2-fold. The authors should comment.
9. Line 269. Reference for “traffic light reporter” cells should be provided.

Referee #3

(Remarks to the Author)
The manuscript by Schuhmacher et al. titled: ”Creating bottom-up RNA transfer vehicles from synthetic 1 protein assemblies “describes a ML-based design of an RNA carrier. The authors screened a variety of different assemblies and delivered various payloads in mouse and pig models. It is an interesting, innovative and timely work although there are several concerns that need to be address prior to further consideration.

The following concerns are listed below to help the authors to improve their manuscript:
1. A general question: what is the reason to establish a complicated reporter system when EGFP can be effectively delivered?
2. Line 123: Some explanation of HE0902 nature is suggested. It would also be beneficial to the readers to present it in Figure 1A.
3. Line 130: RNA release into the supernatant. It is unclear how this test was performed and what conclusion can be made from this experiment. What triggers the RNA release into the supernatant, and wouldn’t it be an obstacle to efficient intracellular delivery?
4. Line 183: STV-C8 vesicles enriched their cargo RNA more than 10,000-fold in the supernatant (Fig. 2b) – compared to what?
5. Line 187: In line with previous reports, mitochondrial RNAs were depleted from STV-C8 particles, presumably because they are not accessible for packaging – are other types of cellular RNAs also packaged into the particle? If so, this could be a safety concern and will require further investigation to show there are no issues.
6. Line 209: Why is the comparison to LNP performed differently from comparison to other methods?
7. Line 210: Unmodified mRNA is a potent trigger for the innate immune response – comparison to synthetic unmodified RNA is required to claim that the “biological” production alleviates the immunogenicity.
8. Line 254: confirming their ability to penetrate deeper into the tissue – judging by the Figure, STV-C8 transduced outer

layers more efficiently and didn't get deep inside of the organoid.
9. Line 295: To assess the delivery vehicle's applicability to muscular dystrophies, it is essential to ensure that the RNA is delivered to muscles and not to other cells at the location of injections. To do that, the authors can perform a western blot and show the expression of the truncated protein.
10. What is missing in the paper is to verify that these RNA carriers are not causing liver damage (via examining liver enzyme release and histology of the liver) and do not induce a pro-inflammatory response by triggering pro-inflammatory cytokines, interferon responsive genes and activating lymphocytes.

Version 1:

Reviewer comments:

Referee #1

(Remarks to the Author)
My comment on benchmarking to other delivery tools has been well addressed.
The more careful quantification of the gene editing has improved the work.
My previous comment has not been addressed:
"The achievement of the AI field is not just that such sequences have some activity but that they adopt desired assembly with nearly atomic precision. In the work here there is little evidence as to the adopted assembly of the novel constructs: proposed organisation (e.g. Fig. 1a,b and extended Fig. 4a) are not well supported by the tomographic analysis in Fig. 2e."

It is very common that oligomerization domains change their multimeric state upon fusion to different components. The tomographic image in Fig. 2e lacks sufficient resolution or controls to assign the density to the intended assemblies. Vesicles will often contain cell components other than those explicitly directed for packaging.

I am not persuaded that tests of transduction of an immortalized muscle cell-line convey the important challenge of therapeutic delivery to real skeletal muscle.

Overall my assessment is that there is some good biological performance demonstrated but the molecular assembly strategy would not surprise those in the field; together with the lack of molecular validation of the intended assemblies I would not recommend publication in Nature.

Minor points:
Dynamic light scattering graphs should substitute size for radius or diameter, for less uncertainty.

In vivo imaging would preferably spell out the meaning of grayscale and green coloring in each legend.

Referee #2

(Remarks to the Author)
the authors have addressed my concerns.

Referee #3

(Remarks to the Author)
The authors have address nicely my concerns.
Please find below my extra comment(s) for each concern:

Comment 1:
I appreciate your addition of the detailed explanation in the supplementary note, as well as the correlation analysis.

Comment 2:
Although I believe that showing the parent structure as a prior art would be beneficial for highlighting the novelty of new structures, I agree with the decision to display in the extended data. Thank you.

Comment 3:
Thank you for clarifying this point. ine 183: STV-C8 vesicles enriched their cargo RNA more than 10,000-fold in the supernatant (Fig. 2b) – compared to what?
Thank you for updating this figure 2, it is now clear.

Commet 4:
Fine. Thanks

Comment 5:
I appreciate the effort to modify this figure.
I would be grateful if it was possible to clarify (and point out in the main text) the means of comparing the amount of

particles/mRNA when benchmarking genetically encoded systems, i.e. was the concentration based on the volume of supernatant collected from producer cells or was the number of particles evaluated or was the amount of RNA within the particles evaluated? The same question can be extended to Fig.1h.

In addition, it wasn't mentioned what type of cells were transfected for the LNP benchmarking. For the purposes of uniformity, I would strongly suggest comparing LNP and STV-C8 on the same cell lines as shown in Fig.2f.

Comment 6:
I appreciate the additional data and agree with this conclusion.

Comment 7:
Thank you for rephrasing this.

Comment 8:
The addition of myotubules to the list of cell models is beneficial, since they are known to be hard to transfect. Please note that there is no indication of the concentration of STVs used in the methods section.

Indeed, showing a truncated protein compared to wild-type by means of western blot would not be an option in this case and I understand the difficulties related to the use of large animals. However, I believe that this experiment is not demonstrating the clinical applicability of STV-C8 to MDs to its full potential. If it would be possible, I would suggest using cDNA to demonstrate that the deletion happened in cells that actually express dystrophin. If it is not possible, I would suggest highlighting some limitations in the applicability of this approach to DMD: 1) i/m injections are currently not applicable to DMD due to cardiac and diaphragm involvement; 2) judging by the mouse experiments, the non-targeted STV do not show muscle biodistribution, so they wouldn't be ideal to systemic injection; 3) since many other delivery vehicles are capable of penetrating the muscle upon i/m injection, they will need to be compared in the future; 4) cell-level specificity analysis would be required in the future. I agree that these initial experiments are nonetheless valuable, but I suggest altering the wording for a more realistic evaluation.

Comment 9:
This data sufficiently demonstrates low toxicity and immunogenicity of STV. Thank you.

Version 2:

Reviewer comments:

Referee #1

(Remarks to the Author)
I acknowledge that the authors have made substantial efforts but the central points from my previous review have not been addressed. In fact the extra data strengthen my assessment that the manuscript's interpretation of achieving the desired structural organization is not supported by the data.

My central point has been that there are many established ways to deliver nucleic acid into cells and an important advance would require either:

(i) exceptional delivery efficiency to relevant tissues inside a living animal

or

(ii) substantial progress in building a well-validated sophisticated protein architecture.

Here there has been further progress of cell transduction ex vivo, which does not meet criterion 1, since optimized delivery to muscle inside an animal is the key challenge for the field. Some degree of delivery to muscle for DMD in living animals has been shown for more than 20 years, e.g.
https://pubmed.ncbi.nlm.nih.gov/9239410/
https://pubmed.ncbi.nlm.nih.gov/11812282/

This revision does not provide any evidence that supports that the design goals have been met, for criterion 2. The data further support a highly heterogeneous mix of unknown composition.

Validation of the protein engineering aspect of the paper and the integrity of the assembly depends on Fig. 2e and Ext. Data Fig. 6. Fig. 2e shows a clear high contrast membrane and heterogeneous low contrast contents of the vesicle. Ext. Data Fig. 6a shows weak signal to noise and high variability of the proposed vesicles. Ext. Data Fig. 6e does not strengthen my

confidence in their assembly; the data show a long smear of assemblies, consistent with highly heterogeneous assemblies. Indeed there are some bands there as a small proportion of the total sample. Additionally it is hard to be sure what molecular weight the bands relate to, because native PAGE mobility is not proportional to molecular weight but a relationship between shape, size and charge. Ext. Data Fig. 6f appears only to be wishful thinking. Lots of shapes would fit into the vesicle tomogram, and this figure inserts what the authors would like it to be, without any evaluation of how well it fits or what else could fit. I could not recommend publication of this interpretation of this figure section at any journal.

Referee #3

(Remarks to the Author)
The authors have nicely addressed my concerns.

Version 4:

Reviewer comments:

Referee #1

(Remarks to the Author)
The authors have added additional mouse imaging of high efficiency of mRNA delivery compared to LNP delivery, although without describing to what extent this fits desired tissue tropism. They have also performed further quantification of STV-C8 assemblies by EM from concentrated supernatant, which supports the formation of particles in the desired size range. CryoET data, as is intrinsic to the method, are only able to give low resolution information on the particle assembly and the authors have not added further statistics or quantification of uncertainty of fit to protein models and with RNA segmentation. Hence the data support the generation of designed particles with high delivery efficiency in different preclinical models, without the ability to validate details of the structural organisation because of the difficulty of the analytical systems.

Open Access This Peer Review File is licensed under a Creative Commons Attribution 4.0 International License, which permits use, sharing, adaptation, distribution and reproduction in any medium or format, as long as you give appropriate credit to the original author(s) and the source, provide a link to the Creative Commons license, and indicate if changes were made.
In cases where reviewers are anonymous, credit should be given to 'Anonymous Referee' and the source.
The images or other third party material in this Peer Review File are included in the article's Creative Commons license, unless indicated otherwise in a credit line to the material. If material is not included in the article's Creative Commons license and your intended use is not permitted by statutory regulation or exceeds the permitted use, you will need to obtain permission directly from the copyright holder.
To view a copy of this license, visit https://creativecommons.org/licenses/by/4.0/

Revision of: Creating bottom-up RNA transfer vehicles from synthetic protein assemblies (Nature 2024-09-19965)

Response to Referees

We respond point by point to the referees' comments and include these changes in the manuscript attached. The referees' comments are colored in **blue**, while our responses are in **black**. The revised parts in the main text of the manuscript are highlighted in yellow.

Referee #1:

This manuscript employs previously published AI-derived oligomeric domains, links them to previously established modules for RNA binding and membrane interaction and then applies the resultant novel constructs for RNA transfer in a variety of cell-line and animal systems. It is likely that there are some favorable properties of these constructs. The constructs are benchmarked to lipid nanoparticle and other protein carrier systems, although it is not fully clear that the rival approaches are ideally assembled and so the key property of interest is an absolute level of delivery efficiency.

We appreciate the referee's insightful comments and fully agree that a fair and rigorous benchmarking against established delivery systems is essential to evaluate the true efficiency of our constructs. Motivated by this point, we carefully expanded the benchmarking experiments - this time using solely materials obtained from the original laboratories via Addgene.

In addition to our previous approach, where we adapted the rival approaches to our experimental setup (identical plasmid backbones, EGFP cargo mRNA, medium-sized production scale), we now performed the comparison using the original setup of the rival approaches. To do so, we obtained all packaging and cargo RNA coding plasmids directly from Addgene and adapted our STV-C8 system to package their Cre cargo mRNA. Additionally, we concentrated the samples by ultracentrifugation as described in the original manuscripts.

We compared Cre mRNA delivery efficiency of STV-C8 and the other genetically encoded delivery vehicles (SEND, VLP, EPN24) in Lox-stop-Lox (LSL) EGFP reporter cells for two particle concentrations. In this assay, STV-C8 induced EGFP expression in nearly 100% of cells even at the lowest particle concentration tested, whereas EPN24, VLP, and SEND reached only about 60%, 20%, and less than 10% EGFP-positive cells, respectively, confirming our initial results shown in Figure 2f. We included these additional results in the new Extended Data Fig. 6d.

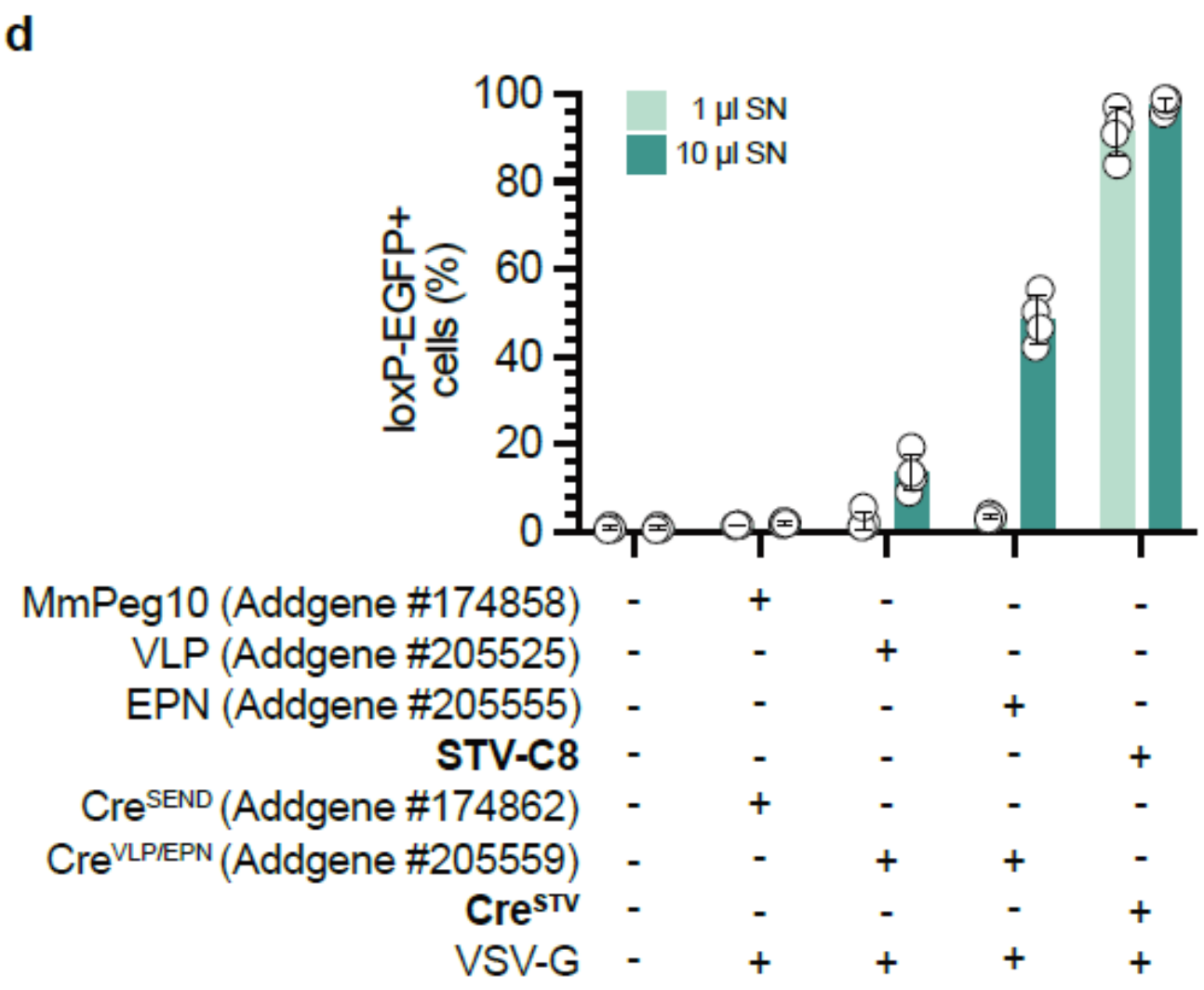

Extended Data Fig.6 | Comprehensive characterization and benchmarking of STV-C8. (...) **d,** Benchmarking of STV-C8 efficiency for delivering Cre mRNA into lox-stop-lox (LSL) EGFP reporter cells for different cell-derived RNA transport vehicles (mean ± s.d. for n = 4 biological replicates). (...)

Furthermore, we set up an alternative benchmark experiment for lipid nanoparticles (LNP). For this, STV-C8 was compared to clinically used lipid nanoparticles, formulated with the ionizable lipid ALC-0315 that is used in the COVID-19 vaccine BNT162b2. We performed a serial dilution of LNP and STV-C8 packaged EGFP mRNA on target cells and found a more than 1,000-fold higher transfection rate of STV-C8 compared to LNPs (see new Fig. 2g). The data of the previous experiment have been moved to Extended Data Fig. 6e.

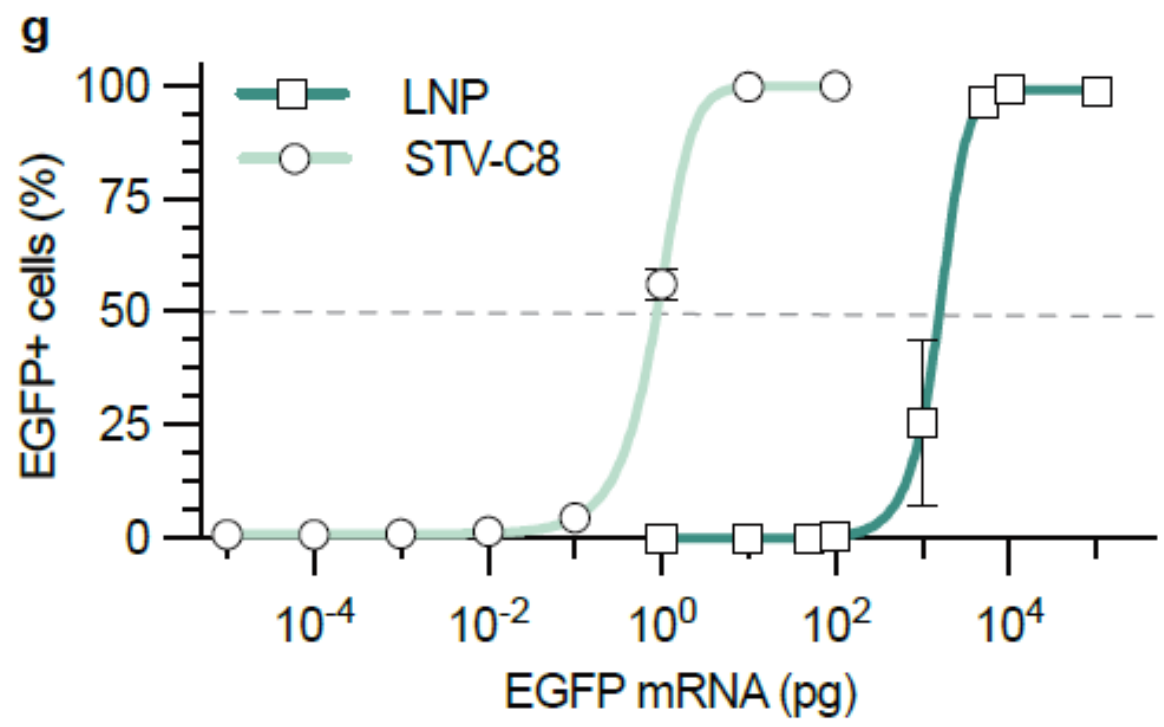

Fig. 2 | Characterization and programming of the STV-C8 RNA carrier. (...) **g,** Percentage of EGFP positive cells as a function of EGFP mRNA input delivered by STV C8 or by LNPs. STV-C8 RNA content was quantified by absolute RT-qPCR against an in vitro transcribed reference EGFP RNA. The expression analysis was performed by Flow Cytometry (mean ± s.d. for n = 3 biological replicates). (...)

In sum, the benchmarking experiments confirmed higher mRNA cargo transduction using STV-C8 compared to other genetically engineered vehicles or LNP delivery systems (see Fig. 2f,g, Extended Data Fig. 6d,e). We believe that this additional data now provides a clear and quantitative demonstration of the delivery efficiency of STV-C8 and addresses the referee's concern directly. We are grateful for this suggestion, as it significantly strengthened our study and allowed us to more convincingly highlight the advantages of STV-C8 over current state-of-the-art systems. Additionally, we provide all sequences in supplementary tables and plan to distribute the plasmids via Addgene to enable the delivery community to perform their own benchmark experiments.

Following the referee's insightful suggestion to perform an absolute quantification, we determined absolute values of RNA transduction in picograms of STV protein and compared these with absolute values of translated target mRNA in recipient cells. Interestingly, STVs with cyclic symmetries exhibit a higher ratio of RNA per packaging protein, suggesting that these symmetries contribute to an increased RNA loading capacity. We included this new data point as Extended Data Fig. 2d. Furthermore, we added the following paragraph to the Results section of the manuscript: "Additionally, we found a clear correlation between the efficiency of STV release and cargo RNA delivery. STVs built on cyclic symmetries transferred more RNA per packaging protein, indicating higher RNA loading capacity compared to other symmetries such as icosahedral (Extended Data Fig. 2d).".

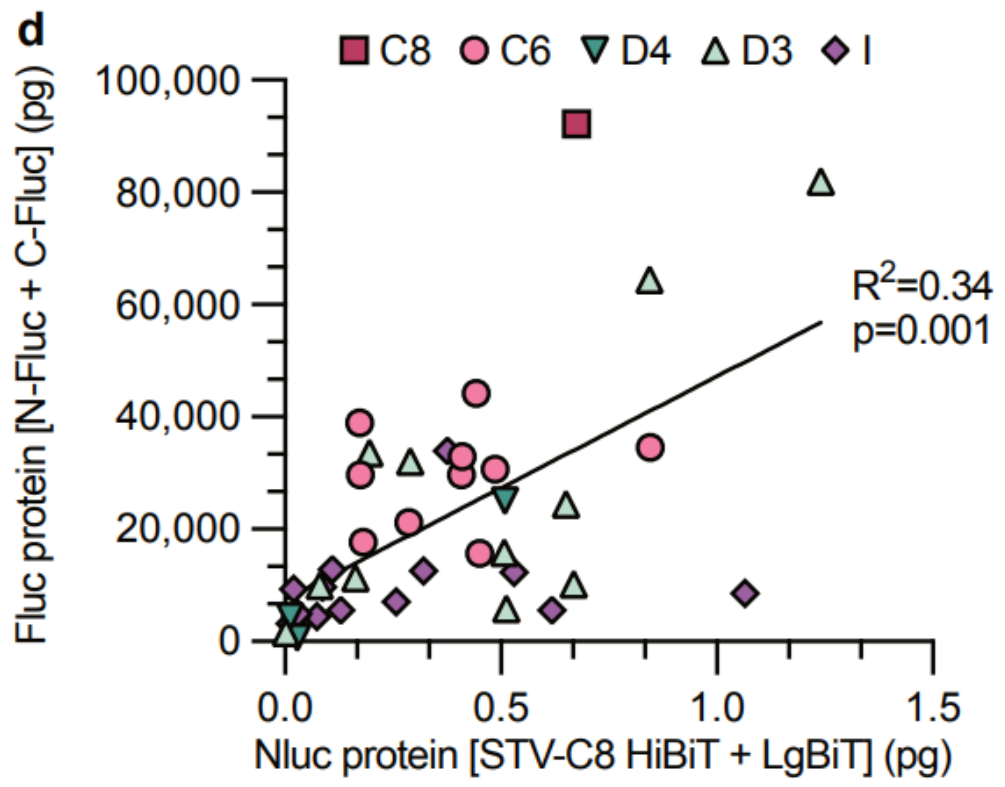

Extended Data Fig. 2 | Establishment and validation of the dual Split-Luciferase based screening method. (…) **d,** Correlation analysis of STV release into the supernatant and mRNA delivery efficiency into target cells for different STV symmetries (C, cyclic; D, dihedral; and I, icosahedral). Both dimensions are measured as total protein content by standardizing against Fluc or Nluc reference proteins (each data point shown as mean from n = 6 biological replicates). (…)

We thank the referee for this insightful input and are confident that we have adequately addressed the points regarding particle release and RNA packaging.

1B: From a conceptual point of view, the text expresses surprise that AI-derived domains show activity, whereas I would suggest that the field is fully accustomed to the activity of AI-based proteins over the last 2 years. The achievement of the AI field is not just that such sequences have some activity but that they adopt desired assembly with nearly atomic precision. In the work here there is little evidence as to the adopted assembly of the novel constructs: the proposed organization (e.g. Fig. 1a,b and extended Fig. 4a) is not well supported, with the only exploration being the tomographic analysis in Fig. 2e.

As the referee correctly pointed out, AI-derived proteins have been applied in recent studies. As an example, we cite in our manuscript impressive studies reporting the identification of a novel Cas9 enzyme (Ruffolo et al.) and novel GFP variants (Hayes et al.) with unique properties. These works start from natural template proteins and generate new variants through AI-guided protein engineering. In contrast, truly *de novo* designed proteins—i.e., proteins without any natural template—remain rare.

Most efforts in the *de novo* design space focus on protein binders, whereas the AI-designed assemblies forming the scaffold of our STV-C8 construct have not yet been applied to address biological questions. We agree with the referee that the distinction between *de novo* design and the redesign of natural template proteins was not sufficiently described in the manuscript. We have now clarified this point by revising the sentence as follows: "Applying such tools that enable true *de novo* design of proteins has the potential to overcome the limitations of natural protein diversity, unlocking new directions in various areas of biological and biomedical research."

Overall, our wording was indeed misleading. Our intention was to highlight that this study is among the first to demonstrate the activity of a complex, AI-designed system explicitly *in vivo* and in particular in a large-animal model. This distinguishes it from previous work that focused primarily on *in vitro* validation. To further emphasize the biological relevance of our work, we added extensive immunological and toxicological analysis to the manuscript in Extended Data Fig. 9.

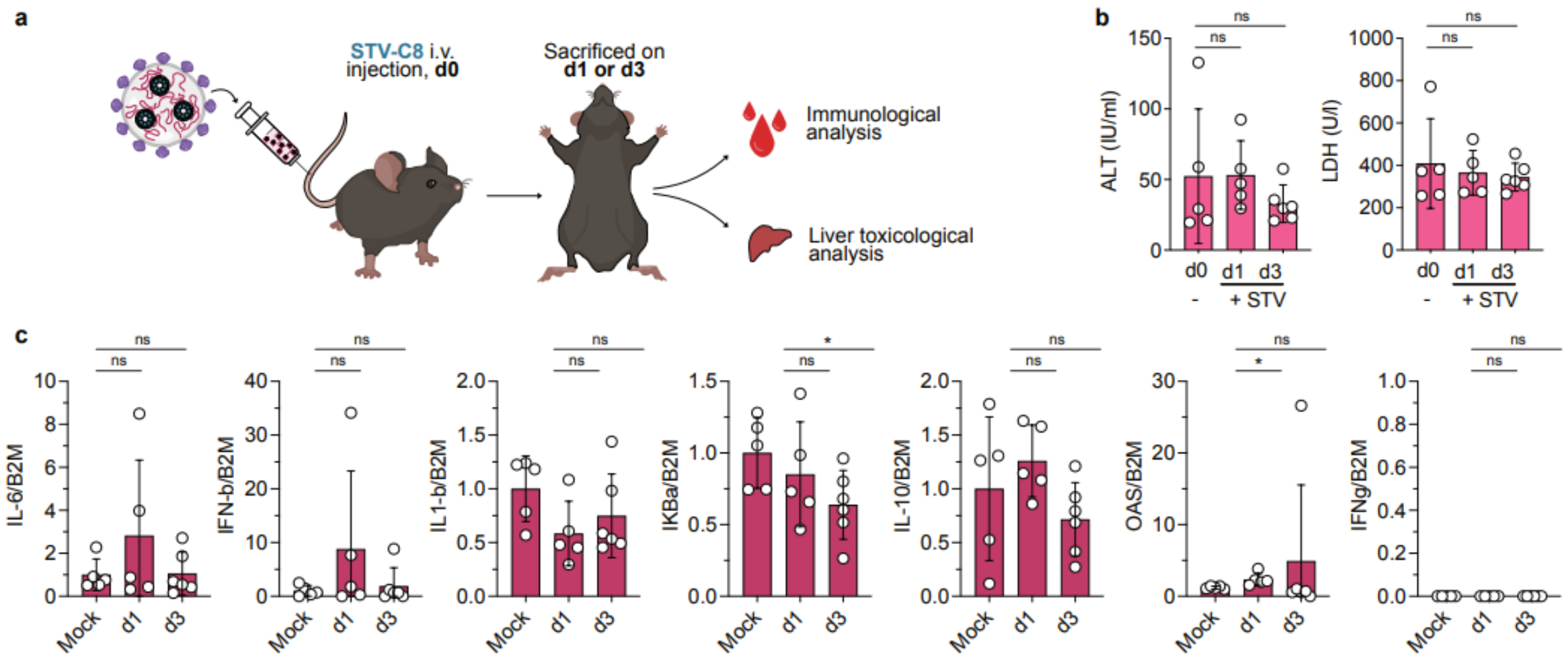

Extended Data Fig. 9 | Evaluation of STV-C8-mediated immunological response and liver damage in vivo. a, Scheme of the experimental setup for testing immunological markers in blood and liver toxicity. **b,** Analysis of potential liver toxicity (ALT, alanine aminotransferase) and broader toxicological side effects (LDH, lactate dehydrogenase) of animals, treated by systemic STV-C8 injection (mean ± s.d. for n = 5-6 treated animals). **c,** Testing for immunological markers by RT-qPCR (one-way ANOVA, *P < 0.05, mean ± s.d. for n = 5-6 treated animals).

Notably, during the review process of this manuscript, a study by Vazquez Torres et al. was published in *Nature* (2025), titled "De novo designed proteins that neutralize lethal snake venom toxins." In that work, the designed inhibitors were validated both in vitro and in vivo in mice. We have revised the corresponding sentence and toned down the claim to reflect the appearance of related in vivo work during the review process. The corresponding sentence [line 315f] now reads: "To our knowledge, this is one of the first examples of an AI-designed protein tested *in vivo* so far."

In summary, our work is the first to use an AI-designed protein assembly in a biological context, the first that comprehensively describes immunological reactions of an AI-designed protein *in vivo,* and

the first to validate the function of such a protein in a large-animal model. Therefore, we believe that our work is of broad interest for the protein design as well as the delivery community.

1C: From a functional point of view, delivery of RNA to cells in culture has been well established through many methods, so the delivery in animals would have the higher importance for establishing novel performance of the approach. Here the data have important uncertainties. It would be important to show data from a mouse treated with negative control particles and imaged under the same conditions for EGFP delivery.

We fully agree with the referee: in contrast to i*n vitro* applications, where RNA transfer is well established, efficient *in vivo* RNA delivery remains challenging and often represents a major bottleneck for therapeutic applications. To validate our novel vehicle, we therefore focused on demonstrating its performance specifically *in vivo*, using different animal models. We tested various RNA cargos in mice and pigs to assess both tissue distribution and biological activity. As suggested by the referee, for this revision, we performed additional systemic injections of STV-C8 without EGFP mRNA as negative control under identical conditions. The subsequent whole-body clearing and imaging revealed no nonspecific signal or background throughout the body. We included this important control as a whole-body overview in panel b of Figure 4 (Fig.4b).

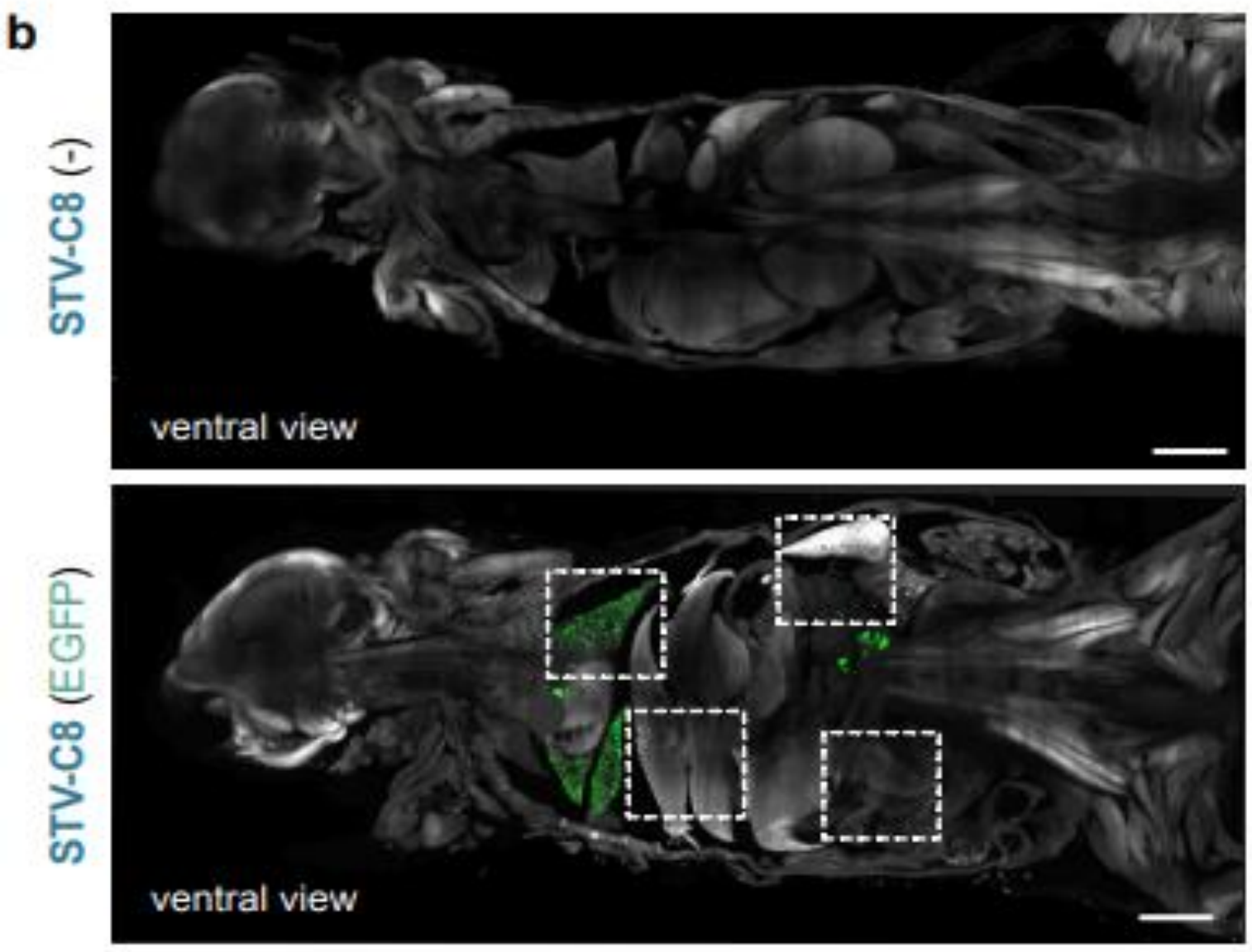

Fig. 4 | STV-C8 biodistribution and delivery of gene editing cargos in mouse and pig models. (…) **b,** Ventral view of amplified EGFP expression in cleared mouse body, treated with EGFP mRNA or empty STV-C8 vesicles, imaged by light-sheet microscopy, 72 h post i.v. injection (scale bar: 5 mm). (…)

1D: Dystrophin exon deletion seems to have been assessed by PCR with flanking primers. PCR is known to preferentially amplify shorter inserts, i.e. the deleted version. Analyzing the amount of PCR products of different size either on a gel or by nanopore sequencing would be subject to the same potential amplification bias. Therefore, further evidence would be required for confidence in the

frequency of deletion and thereby the activity of the method. More robust assays of stable genetic change that avoid potential PCR amplification issues are now standard in the gene therapy field.

This point is well taken. The unbiased detection of a large deletion as applied in this proof-of-concept experiment is prone to potential amplification bias. We have addressed this valid critique in several ways: first, by removing the passages that referred to specific editing efficiencies. The proof-of-concept experiment presented here is intended solely to demonstrate the general applicability of STV-C8 in the context of a gene editing approach, as well as to highlight the safety and tolerability of our novel targeting vehicle. Translational or therapeutic applications, in order to be clinically meaningful, would need to be delivered via systemic administration. To this end, we are currently adapting our modular system to achieve optimal transduction in muscle tissue. However, this lies beyond the scope of the present manuscript. To further substantiate our claims regarding the immunological tolerability of our delivery system, we have performed additional analyses of the local injections and included the results as a new panel in the manuscript (Extended Data Fig. 10; see also our response to the following question). Furthermore, to validate our previous statements and the relative editing efficiency shown in Fig. 4e, we employed an alternative approach in new experiments, using digital PCR to determine the efficiency. Although we observed considerable variability—which is to be expected following a local, focal injection—the results (see Rebuttal Fig. 1) support our original analysis.

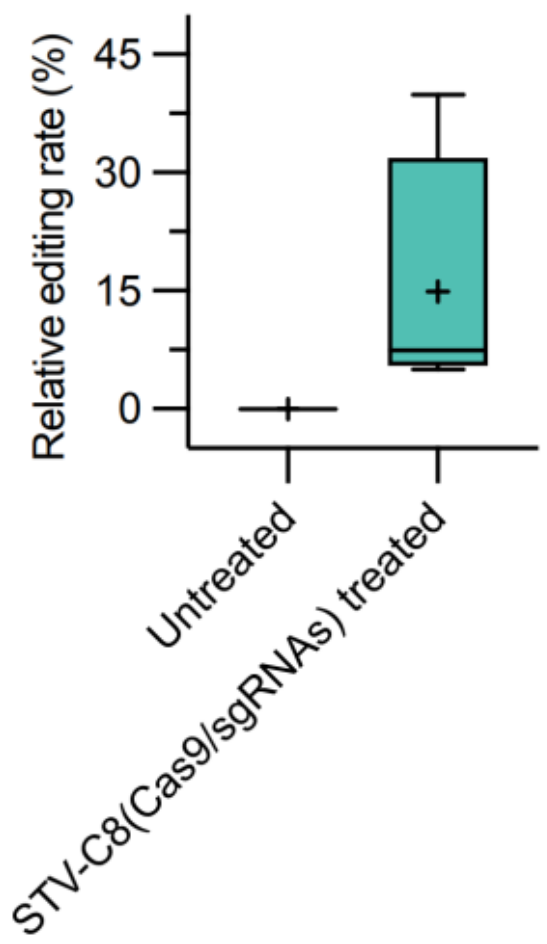

Rebuttal Fig. 1 | STV-C8 delivery of gene editing cargos in pig. Editing efficiency analysis with digital PCR (dPCR) of treated and untreated pig samples.

1E: The work is described “as a treatment strategy for Duchenne Muscular Dystrophy” and yet the EGFP data seem to show little delivery to skeletal muscle from intravenous delivery. I would suggest that these conceptual and practical uncertainties would need to be resolved before the manuscript is of broad interest for the protein design or genetic delivery communities.

We apologize that this aspect was not elaborated comprehensively enough. As the referee correctly pointed out, in the systemic injection of the EGFP reporter into mice, pronounced skeletal muscle delivery is not obvious. Nevertheless, in an independent project, we observed that muscle tissue surrounding the injection site showed pronounced delivery. This prompted us, that also the direct injection into pig muscle should result in a good transduction efficacy. The obtained editing frequencies of about 8 to 40 % depending on the distance of the injection site analyzed confirmed this. Since only

DNA and RNA samples from this specific animal was taken, we could not further elucidate this question. Unfortunately, due to ethical restrictions using large animal models hindered us from repeating this exact experiment to get additional material. Nevertheless, we could confirm the general activity of STVs by conducting an experiment on myotubes. Here, a highly efficient delivery of an EGFP reporter into 89.6% of myotubes could be observed. These results were added to the manuscript as the new Extended Data Fig. 10,a together with the following statement in the Results section: "We first confirmed that STV-C8 efficiently deliver their cargo RNA into muscle cells (Extended Data Fig. 10a)." As a next step, we will harness the indicated possibility to modify STVs in a modular way for cell-type specific delivery. Setting up porcine muscle-specific STVs is currently ongoing but will be part of another, more DMD-focused manuscript. For this work, the respective paragraph is to demonstrate in a pure proof-of-concept study the general applicability of STVs in a large animal model, with high relevance to human applications. We revised the respective paragraph to point out the proof-of-concept characteristic of this experiment (please refer also to our answers to the previous question).

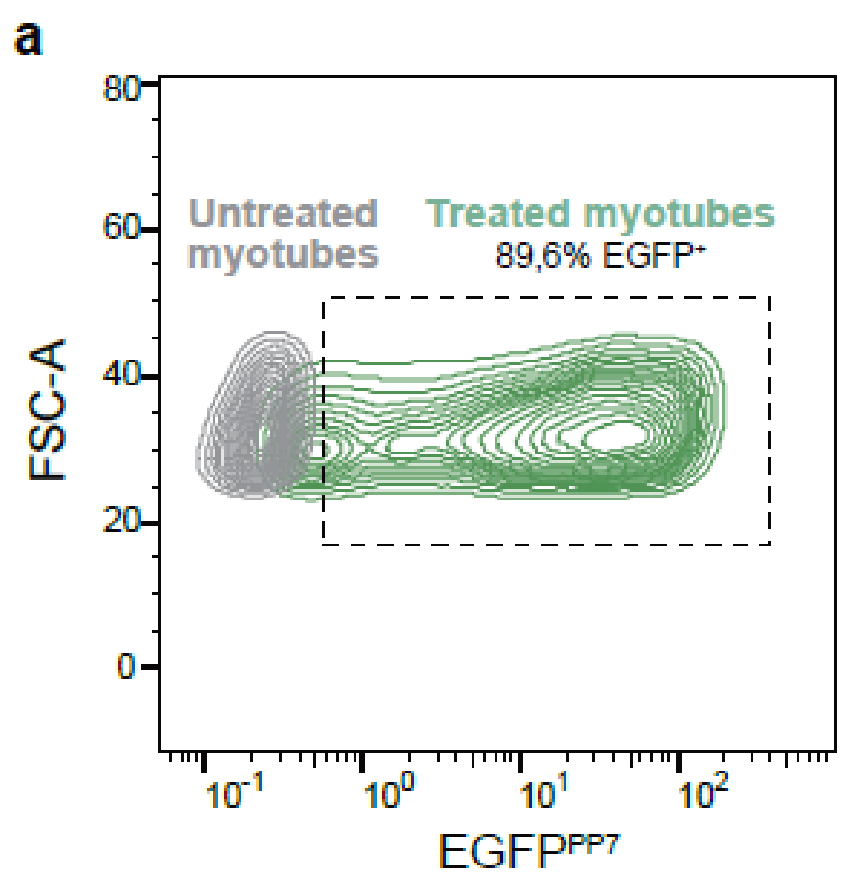

Extended Data Fig. 10 |Continued analysis of intramuscular delivery of mRNAs with STV-C8. a, Quantification of the EGFP mRNA delivery into C2C12 mouse myotubes by Flow Cytometry. (…)

Furthermore, we used the revision to further confirm the safety of the application by analyzing possible local immunological reactions following STV treatment. The respective results were added to the manuscript as the new Extended Data Fig. 10,c-e.

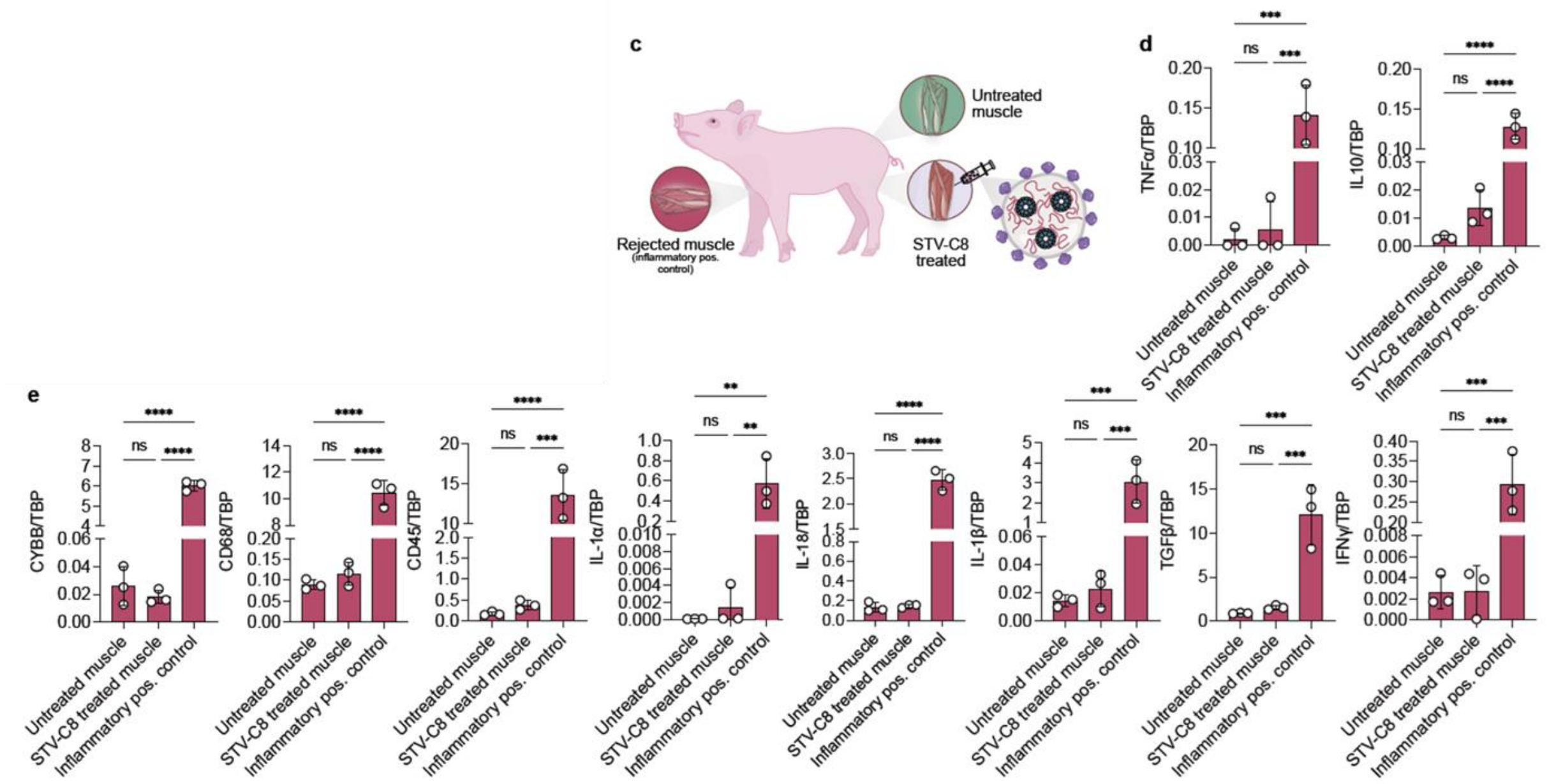

Extended Data Fig. 10 | Continued analysis of intramuscular delivery of mRNAs with STV-C8. (…) **d-e,** Analysis of inflammatory markers of muscle samples by RT-qPCR (one-way ANOVA, ** $P < 0.01$,*** $P < 0.001$, ****$P < 0.0001$, mean ± s.d. for n = 3 replicates of different injection sites).

Minor points:

At various points proteins structures are shown, without explanation of what these represent, i.e. an experimental structure, a schematic or a simulation. – Also Figure legends often need more detail for a reader to understand the constructs and the experiment shown.

We apologize for these shortcomings. As suggested by the referee, we extended throughout the text descriptions of schemes, illustrations, and the figure legends at various points (e.g., see revised figure description of Fig. 1). A detailed description of the experimental setups, along with all construct sequences used, is provided in the revised Supplementary Information section. We added more detailed description of shown protein structures as the referee correctly suggests (e.g. **Extended Data Fig. 1 | Detailed characterization of fundamental STV domains. a,** Solved structure and size of the initial assembly domain HE0902[8].)

Based on the dynamic light scattering, the particles seem substantially smaller than the particle shown from tomography.

Based on this referee remark, we extended our cryo-ET analysis extensively and performed a size distribution analysis of almost 1,000 STV-C8 vesicles confirming a particle size in the range of 50-300 nm, which supports our previous results, obtained from DLS. Furthermore, the characteristics (protein/RNA content, delivery efficiency) of different fractions of STV-C8 vesicles from iodixanol gradients were characterized. For this, STV-C8(EGFP) vesicles were purified through iodixanol gradients and RNA was extracted from three different fractions. The relative amount of EGFP mRNA between the fractions was analyzed by RT-qPCR. The STV protein content was characterized by HiBiT measurement, and the delivery efficiency was measured by adding the fractions to target cells and analyzing EGFP expression by Flow Cytometry after 24 h. This analysis confirmed, that the smaller (15%

iodixanol) fraction contains the highest amount of functional, active STVs. These findings are now included in the manuscript as the new Extended Data Fig. 6a, c.

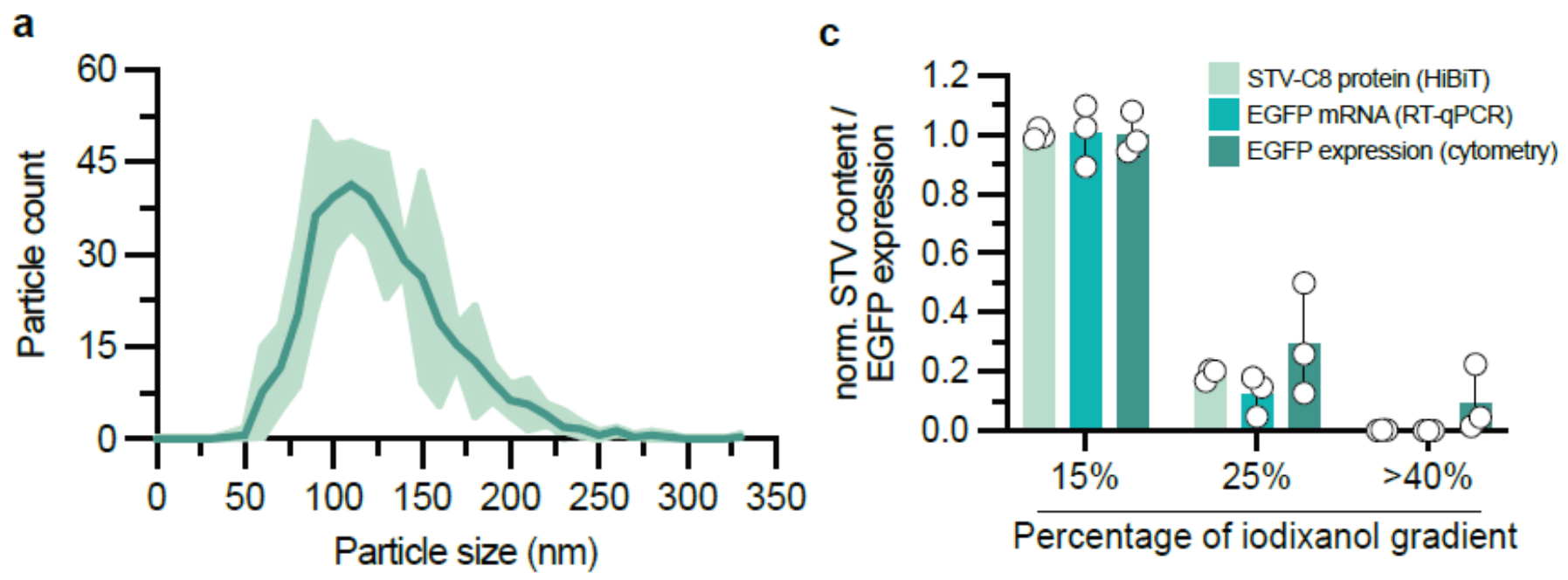

Extended Data Fig. 6 |Comprehensive characterization and benchmarking of STV-C8. a, Size distribution of different STV-C8 vesicles on EM grids (1,100 vesicles analyzed in total, mean ± s.d. for n = 3 biological replicates). (...) **c,** Characterization of different iodixanol concentrations for purifying STV-C8 vesicles by ultracentrifugation, based on STV protein or cargo RNA content and expression in target cells (mean ± s.d. for n = 3 biological replicates).

Based on this extensive additional analysis, we exchanged the tomographic reconstruction in Fig. 2e with a more representative example from the new dataset.

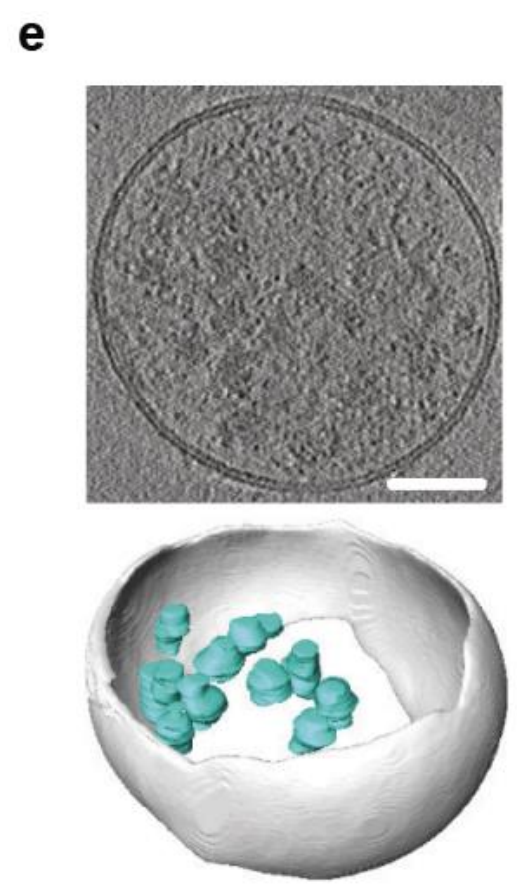

Fig. 2 | Characterization and programming of the STV-C8 RNA carrier. (...) **e,** 6.52 Å-thick slice from a tomographic volume of a representative STV-C8 vesicle (top) and corresponding 3D segmentation showing the STV membrane in grey and the STV-C8 protein assemblies in cyan (bottom) (scale bar: 50 nm). (...)

We thank referee 1 for the helpful comments that allowed us to improve the clarity of our manuscript regarding the novelty of our work and the interpretation of Gene Editing results.

Referee #2:

In this study, the authors use AI to design protein assemblies that combine natural and synthetic protein domains to create a large number of novel RNA transport vehicles. They show that these vehicles are able to transfer RNA much more efficiently than currently available vehicles and that their tropism can be programmed by incorporating computationally designed peptide binders that target specific cell-surface receptors. They use these modified vehicles to transfer cargo RNAs, including gene

editors, to a range of cell-based systems. Finally, the authors show that these vehicles can be used in vivo to transfer RNAs to mice by IV injection and to perform gene editing in pigs by packaging CRISPR/Cas9 to delete an exon from the Duchenne muscular dystrophy gene. The work is remarkable in many respects and shows that generative AI can be harnessed to engineer protein assemblies with potentially powerfully useful properties, including gene editing in vivo. In my view, the findings here are novel and highly significant. Despite my enthusiasm, there are some issues that should be addressed. Foremost among them is that the authors are very sparing in what they report. A basic tenant of scientific publishing is that enough detail should be provided so that others can reproduce the published work. In this case, this will be difficult. Below I elaborate on this and mention a few additional points, some rather minor. (Describe experiments in more detail)

1. Exactly how the authors performed their AI-based protein engineering is very difficult to glean from the information provided. I recognize that the main text may be constrained by space limitations, but supplemental material could describe in much more detail what was done

This point is well taken, and we apologize for the shortcomings identified in the earlier version of the manuscript. In line with the referee's recommendations, we have undertaken a thorough revision by substantially expanding the descriptions of schemes, illustrations, and figure legends throughout the text. In addition, we have provided detailed accounts of the experimental setups, and the complete sequences of all constructs employed are now presented in the supplementary information. Additionally, we added a detailed description of the reporter system in Supplementary Note 1.

Regarding the AI-based proteins, we used diverse RFdiffusion designed proteins from simple molecular starting points. In our previous experiments, we filtered RFdiffusion-designed symmetric oligomers for successfully assembled structures using size exclusion chromatography as the primary selection criterion (see, Watson et al. De novo design of protein structure and function with RFdiffusion. Nature 2023). From the original pool, we synthesized 39 candidate sequences (eBlocks, IDT, DNA) and cloned them as C-terminal fusions to additional STV components, including PHPLC (membrane binding), SynL (late budding), and tdPCP (RNA-binding protein). We then functionally tested these constructs for their ability to support STV-C8 release, STV uptake, and mRNA delivery (see also Fig. 1).

2. The authors design their vehicles using well-known membrane-targeting domains, viral late domains (in tandem), and RNA binding motifs, but no sequences are provided in the material that I reviewed. In particular, what were the nature and sequences of linker domains used? Again, anyone wishing to verify these findings would need to start ab initio and would have no way of generating the exact vehicles used.

Thank you for pointing towards missing parts of the supplementary information. We made sure now, that all sequences and materials are accessible in the supplementary information tables. Furthermore, we have updated and expanded the information in the supplement (see Supplementary Note 1 | Detailed description of the STV screening and reporter system). All sequences can be found in the Supplement in section "Supplementary Table 1 | Protein, RNA, and DNA sequences used in this study" [line 103]. We would like to apologize that this information was not available in the initial manuscript. Additionally, we plan to distribute the relevant plasmids via Addgene.

3. Extended Fig. 2 shows that VSV-G alone is highly efficient for RNA delivery. The authors should comment.

It is correct that VSV-G alone also shows activity with respect to mRNA delivery in this assay. The vesicular stomatitis virus glycoprotein (VSV-G) itself is able to induce budding vesicles containing cytosolic proteins (Campbell et al., 2019). We added this information and reference to the manuscript now: "VSV-G expression in mammalian cells induces the release of vesicles[21]."

In comparison to the poorly performing cage variant STV-HE0902, which was used in this experiment, this activity appears relatively strong. However, compared to well-performing cage variants such as HE0690 (STV-C8), the activity of VSV-G alone is minor. To demonstrate this, we conducted a new experiment comparing mRNA delivery into reporter cells by vesicles released through VSV-G expression, the original STV design based on HE0902, and the optimized STV-C8 version. This comparison revealed an almost 60-fold higher activity of the optimized STV-C8 compared to VSV-G alone. We have included these new results as a new panel in the manuscript (Extended Data Fig. 5d) and complemented the Results part of the manuscript with "VSV-G expression in mammalian cells induces the release of vesicles[21]. We show that the expression of STV-C8 enhances this intrinsic budding ability of VSV-G by more than 50-fold (Extended Data Fig. 5d).".

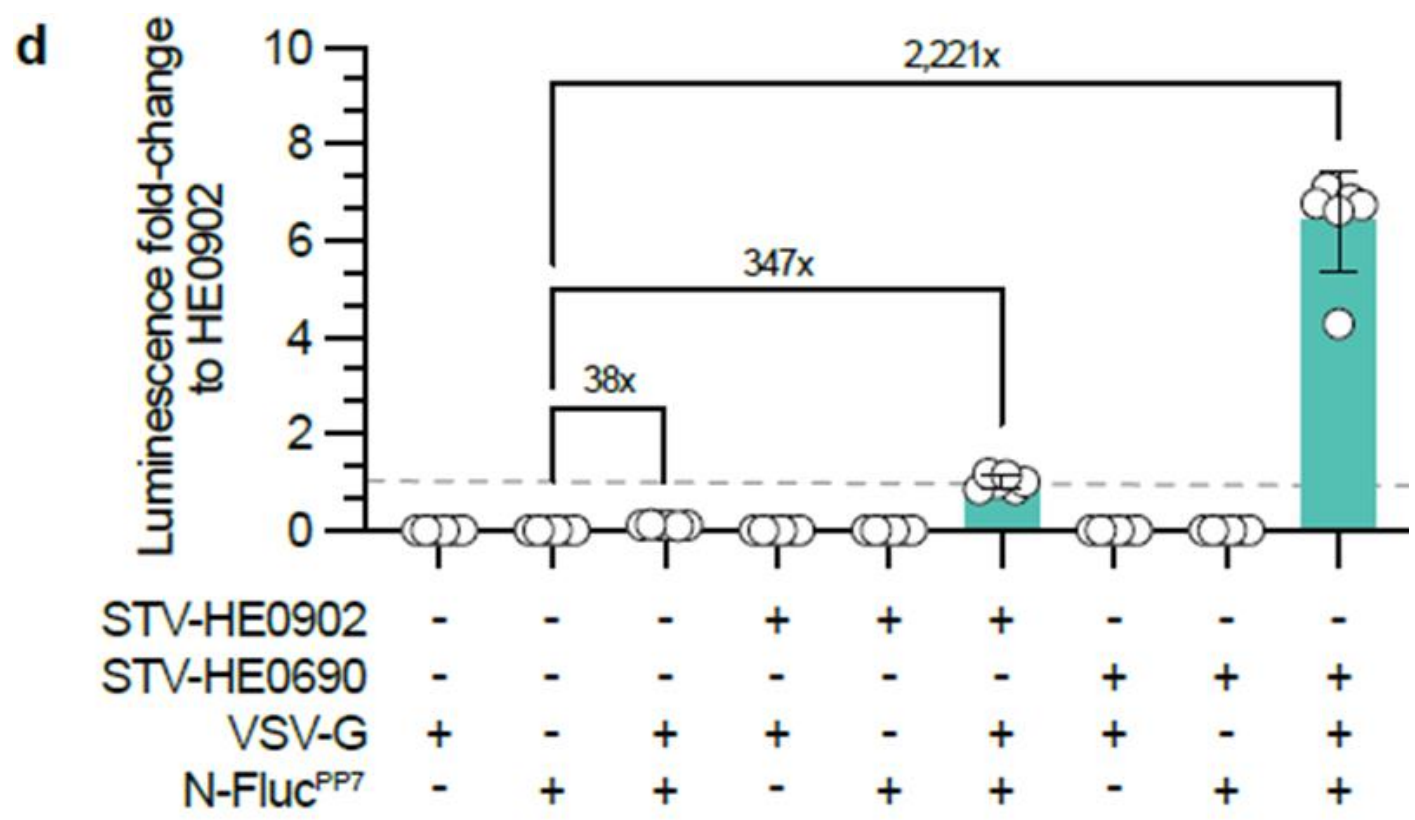

Extended Data Fig. 5 | Analysis of purity and particle parameters of STV-C8. (…) **d,** Comparison of N-Split mRNA delivery into Split-Luc reporter cells for vesicles released by VSV-G expression, the original STV design based on HE0902, and the optimized STV-C8 (mean ± s.d. for n = 6 biological replicates).

4. Extended Data Fig. 4e. It appears that the best vehicles simply release better. The authors should comment. Along these lines, have the authors attempted to measure what would be analogous to "specific infectivity" in a viral system; i.e., how efficiently a vehicle transfers RNA normalized for protein content? This could provide useful information regarding whether some vehicles are particularly efficient at delivering packaged RNA, other things (particle release, RNA packaging) being equal.

This is a valuable observation that prompted us to include an additional data point in the manuscript. We performed a correlation analysis between STV release and mRNA delivery efficiency for different STV symmetries (cyclic, dihedral, and icosahedral). As the referee already indicated, we indeed observe

a correlation between STV release and RNA delivery efficiency. Interestingly, STVs with cyclic symmetries exhibit a higher ratio of RNA per packaging protein, suggesting that these symmetries contribute to an increased RNA loading capacity. We included this new data point as Extended Data Fig. 2d. Furthermore, we added the following paragraph to the Results section of the manuscript: "Additionally, we found a clear correlation between the efficiency of STV release and cargo RNA delivery. STVs built on cyclic symmetries transferred more RNA per packaging protein, indicating higher RNA loading capacity compared to other symmetries such as icosahedral (Extended Data Fig. 2d)."

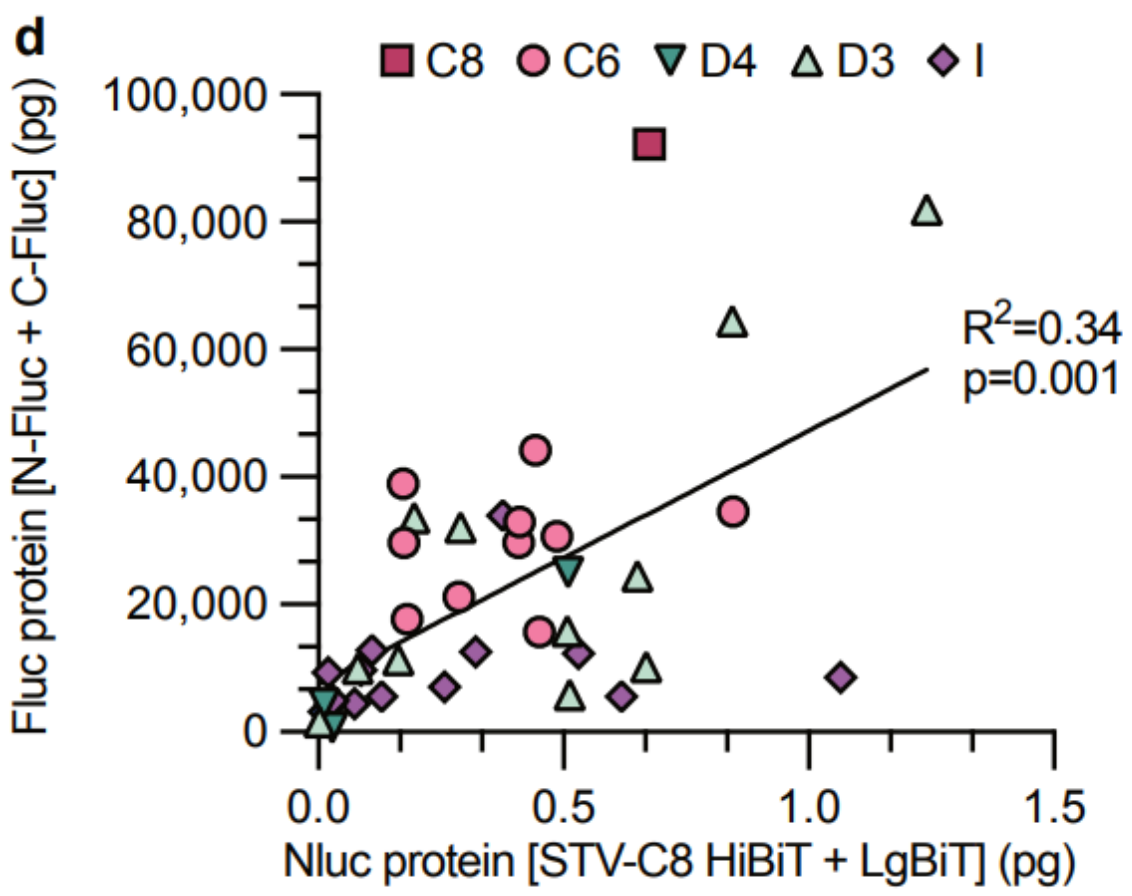

Extended Data Fig. 2 | Establishment and validation of the dual Split-Luciferase based screening method. (…) **d,** Correlation analysis of STV release into the supernatant and mRNA delivery efficiency into target cells for different STV symmetries (C, cyclic; D, dihedral; and I, icosahedral). Both dimensions are measured as total protein content by standardizing against Fluc or Nluc reference proteins (each data point shown as mean from n = 6 biological replicates) (…)

We thank the referee for this insightful input and are confident that we have adequately addressed the points regarding particle release and RNA packaging

5. Very minor, line 188: "mitochondrial RNAs were depleted from STV-C8 particles" doesn't quite make sense. I think the authors mean "excluded". Similarly, line 952.

Thank you for your careful observation of this inaccuracy. Accordingly, we have revised the wording from "depleted" to "excluded" in both instances.

6. The authors report that their STV-C8 vehicles contain protein assemblies with a diameter of around 22 nm inside much larger vesicles (diameter 150-300 nm). I find this very intriguing. Generally, enveloped viruses contain capsids (or the equivalent) that are much more similar in diameter to the outer membrane; indeed, the protein component bends the membrane as it buds, essentially wrapping itself closely in the membrane. For example, budding retroviruses contain an immature Gag lattice that is only slightly smaller in diameter than the surrounding membrane. It's not clear to me, as claimed on line 196, that the assemblies are acting similarly to natural capsid protomers in bending

the membrane. Similarly, if the RNA is bound to the outer surface of the protein oligomers it might well interfere with membrane association (via charge repulsion). The authors should discuss this and also mention, a propos of this point, whether individual vesicles contain multiple protein/RNA assemblies.

We thank the referee for bringing these interesting points to discussion. Based on this, we decided to verify and complement our initial data based on Dynamic Light Scattering (DLS) with a detailed cryo-electron tomography (cryo-ET) analysis. We imaged purified STV-C8 vesicles and analyzed the vesicle size of more than 1,000 vesicles. The vesicles showed an average size distribution of about 50 to 300 nm with a clear peak around 110 nm. This new cryo-ET data allowed us to repeat the analysis of the vesicle content as well. As seen in the previous analysis, a typical vesicle contained dozens of distinct protein structures with a typical diameter of 22 +/- 2 nm. For comparison, we predicted an oligomeric structure of STV-C8 using AlphaFold3. The size and shape of the prediction matched the structures observed in Cryo-ET (Extended Data Fig. 6b).

Next, to determine if this observation is in line with the purification conditions, we compared different fractions based on different iodixanol contents (15%, 25% and >40%) in regard to their relative STV protein content, EGFP mRNA content and EGFP expression. What we observed is, that the 15% fraction clearly contains the highest amount of STVs, mRNA and activity which supports our previous findings. We included these new data points as Extended Data Fig. 6a-c and added the following paragraph to the Results section of the manuscript: "Next, we imaged these STV-C8 induced vesicles and characterized their size distribution using cryo-electron tomography (cryo-ET) (Fig. 2e). In purified supernatants, we observed vesicles ranging from 50 to 300 nm in diameter, with the majority measuring 110 nm (Extended Data Fig. 6a). Inside these vesicles, we detected dozens of protein structures with a typical diameter of 22 +/- 2 nm. Their size and shape matched the AlphaFold-predicted oligomeric structure of STV-C8 (Extended Data Fig. 6b)" as well as "Based on the observed size distribution of STV-C8 vesicles, we characterized the impact of different purification conditions. We found a clear enrichment of STV-C8 vesicles in the 15% iodixanol fraction, as indicated by enriched RNA and protein content and the highest delivery efficiency into target cells (Extended Data Fig. 6c)".

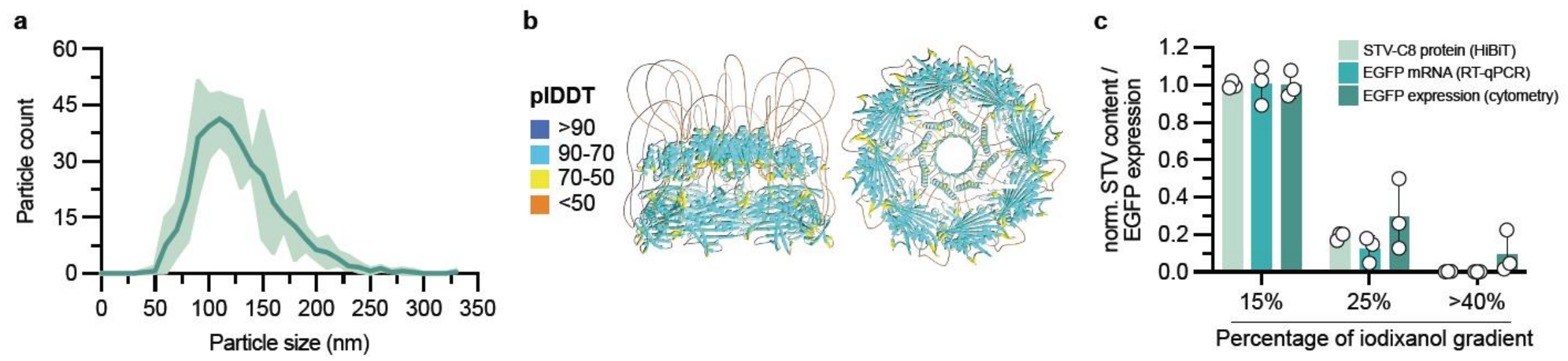

Extended Data Fig. 6 |Comprehensive characterization and benchmarking of STV-C8. a, Size distribution of different STV-C8 vesicles on EM grids (1,100 vesicles analyzed in total, mean ± s.d. for n= 3 biological replicates). **b,** AlphaFold3 structure prediction of the STV-C8 oligomer, colored based on pIDDT score. **c,** Characterization of different iodixanol concentrations for purifying STV-C8 vesicles by ultracentrifugation, based on STV protein or cargo RNA content and expression in target cells (mean ± s.d. for n = 3 biological replicates). (…)

Furthermore, we exchanged Fig. 2e with a more representative example from the new dataset.

e

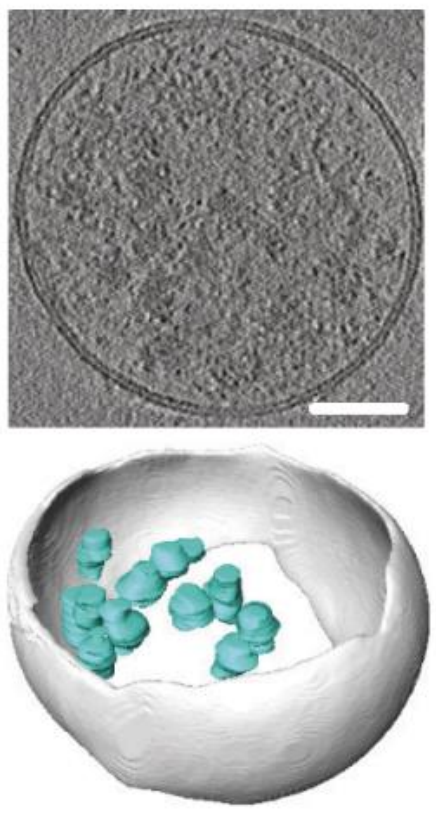

Fig. 2 | Characterization and programming of the STV-C8 RNA carrier. (…) **e,** 6.52 Å-thick slice from a tomographic volume of a representative STV-C8 vesicle (top) and corresponding 3D segmentation showing the STV membrane in grey and the STV-C8 protein assemblies in cyan (bottom) (scale bar: 50 nm). (…)

At this stage, we are confident that both our quantification of vesicle size and our analysis of STV-C8 protein assembly are accurate. While individual STV-C8 assemblies are generally smaller than viral capsids, they nevertheless induce the release of vesicles comparable in size to those of enveloped viruses. This finding suggests a biophysical optimum for vesicle size and supports our initial hypothesis that non-natural synthetic protein assemblies can mimic natural capsid protomers by initiating budding through membrane bending. Moreover, STV-C8 assemblies may not act solely as single units but could also form larger, functional structures. However, the precise mechanism of vesicle budding requires further investigation.

Taken together, we thank the referee for these comments and are convinced that the additional data have strengthened this study considerably, even though not all mechanistic questions could be fully resolved within the scope of this manuscript.

7. Fig. 2f. Do the authors have any thoughts as to why the efficiency of EGFP expression is so variable with the different cell lines tested?

This point is well taken. It is indeed likely that different cell types express distinct surface markers, which can influence membrane fusion, tropism, and subsequent mRNA transfer. For example, the VSV-G envelope used in this experiment is known to depend on its endogenous targets, such as LDL receptors (LDLR). Given this natural restriction and considering the substantial differences in surface marker composition among the tested cell lines, the observed variability was not unexpected. To clarify this aspect in the manuscript, we modified the figure caption to explicitly note the use of VSV-G as the envelope protein: "**f,** Comparison of EGFP mRNA delivery efficiency for STV-C8 and other genetically encoded delivery vehicles, enveloped with VSV-G, into different target cell lines. EGFP expression in target cells was measured by Flow Cytometry (mean ± s.d. for n = 4 biological replicates). Each mRNA was tagged with its corresponding packaging signal."

8. Fig. 2j. The fold change in EGFP expression with the different protein binders is not particularly impressive, around 2-fold. The authors should comment.

This point is well taken. With this proof-of-concept experiment, we wanted to indicate the general ability to use STVs for cell-type-specific delivery and have chosen an approach utilizing mutant VSV-G variants that retain endosomal escape activity but lack LDL-R binding, which can be redirected through conjugation with peptide binders. Indeed, the overall fold change shown in Fig. 2j is modest, particularly in the case of the EGFR binders. However, for IL-7Rα we consider the difference between on- and off-target cell expression to be promising although background activity remains evident. We use here a previously described mutant version of VSV-G that retains its fusogenic activity without binding affinity for the LDL receptor. In line with previous reports (Strebinger et al.), we observed substantial background activity of the VSV-G mutant that interferes with specific targeting applications. To further elaborate on this limitation, we added Extended Data Fig. 7d where we show that mutant VSV-G without any targeting moiety retains almost 50% of its wild-type. We complemented this part of the study with an alternative approach using an established antibody fragment directed against the surface marker CD19. In this experiment, we observed robust and cell–type–specific targeting of CD19-positive cells, with a 4.4-fold increase compared to control cells. To support our peptide binder experiments, we have included this new dataset as Extended Data Fig. 7d and added the following sentence to the Results section of the manuscript: "This finding could be explored further for precise cell type specific targeting and may offer enhanced uptake efficiency compared to previously described targeting strategies based on antibody fragments (Extended Data Fig. 7c, d)[26]". Together, these results demonstrate that both synthetic binders and antibody fragments can impart programmable tropism to STV-C8 particles. We recognize, however, that this promising feature of STVs still offers considerable scope for further optimization. In particular, it is required to substantially decrease the background activity of mutant VSV-G.

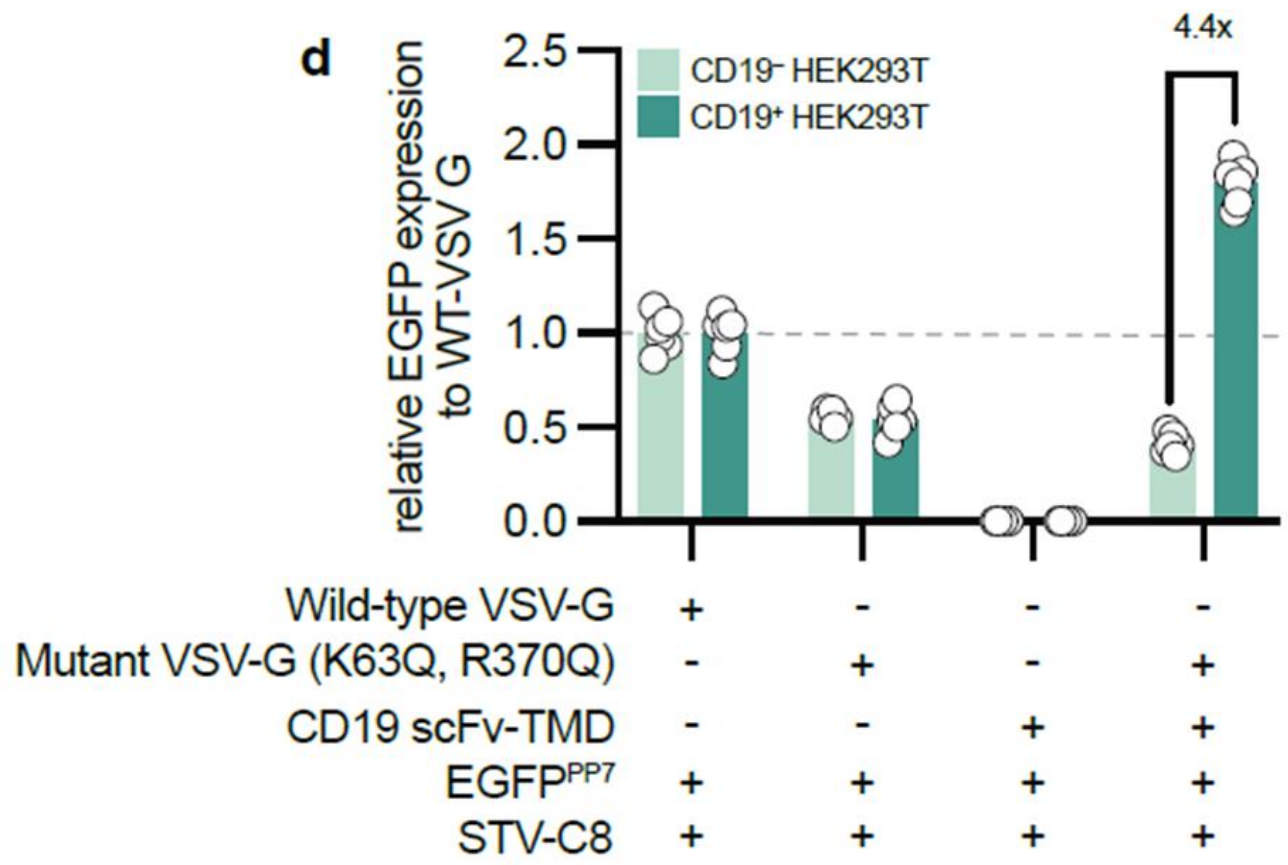

Extended Data Fig. 7 | Programming of STV-C8 cell-type specificity with AI-designed minibinders. (...) **d,** Delivering EGFP mRNA into CD19-expressing cells by co-expressing a signal peptide/transmembrane domain (TMD) fused CD19 scFv for targeting (mean ± s.d. for n = 6 biological replicates), analyzed by Flow Cytometry.

9. Line 269. Reference for "traffic light reporter" cells should be provided.

Indeed, the reference for the traffic light reporter cells (*Truong, D. J. et al. Exonuclease-enhanced prime editors. Nat Methods 21, 455-464, 2024*) was only provided in the Methods section. We have now

added the reference to the corresponding passage in the Results section: "We demonstrated that one of these proteins, OpenCRISPR-1[29], can efficiently be delivered with STV-C8 and induce similar editing rates as wild-type Cas9 in Traffic Light Reporter (TLR) cells[30] (Supplementary Fig. 2b)"

We thank referee 2 for the clear and constructive comments that helped us to resolve uncertainties around aspects such as the STV-C8 vesicle size or targeting specificity.

Referee #3:

The manuscript by Schuhmacher et al. titled: "Creating bottom-up RNA transfer vehicles from synthetic protein assemblies" describes a ML-based design of an RNA carrier. The authors screened a variety of different assemblies and delivered various payloads in mouse and pig models. It is an interesting, innovative and timely work although there are several concerns that need to be address prior to further consideration. The following concerns are listed below to help the authors to improve their manuscript:

1. A general question: what is the reason to establish a complicated reporter system when EGFP can be effectively delivered?

We apologize that the rationale for establishing the reporter cell line was not described comprehensively enough. We designed the split luciferase reporter system, because it enables us to describe the relevant dimensions of a novel delivery system as we describe it here (vehicle release, vehicle uptake, RNA delivery). First, luciferase systems are highly sensitive and provide a broad dynamic range, allowing precise quantitative readout. In addition, while STV release into the supernatant can be monitored via the HiBiT tag fused to the STV construct, complementation of HiBiT with recombinant LgBiT protein within the reporter cells enables us to distinguish between particle release and subsequent uptake by recipient cells. For mRNA delivery, reconstitution of the C-terminal split part of Firefly luciferase (Fluc) with the STV-delivered N-terminal split part of Fluc is required. This additional safeguard makes the system less susceptible to artifacts such as EGFP carryover from STV producer cells. This feature, and given that well-established microtiter assays exist, supports the large-scale screening of constructs while minimizing false positives, ensuring that only genuinely effective constructs are selected for STV production. Moreover, it allowed us to characterize and subsequently optimize production at multiple stages of the workflow, including STV release, STV uptake, and mRNA delivery. To further demonstrate the valuable insights that can be derived from this multi-dimensional screening system we now added a correlation analysis of STV release to RNA delivery efficiency. As expected, a correlation between these two dimensions exist, but interestingly we observed a higher RNA transfer to STV release ratio for cyclic compared to icosahedral symmetries, suggesting that cyclic structures have a higher loading capacity. We added this finding to the revised manuscript in Extended Data Fig. 2d now.

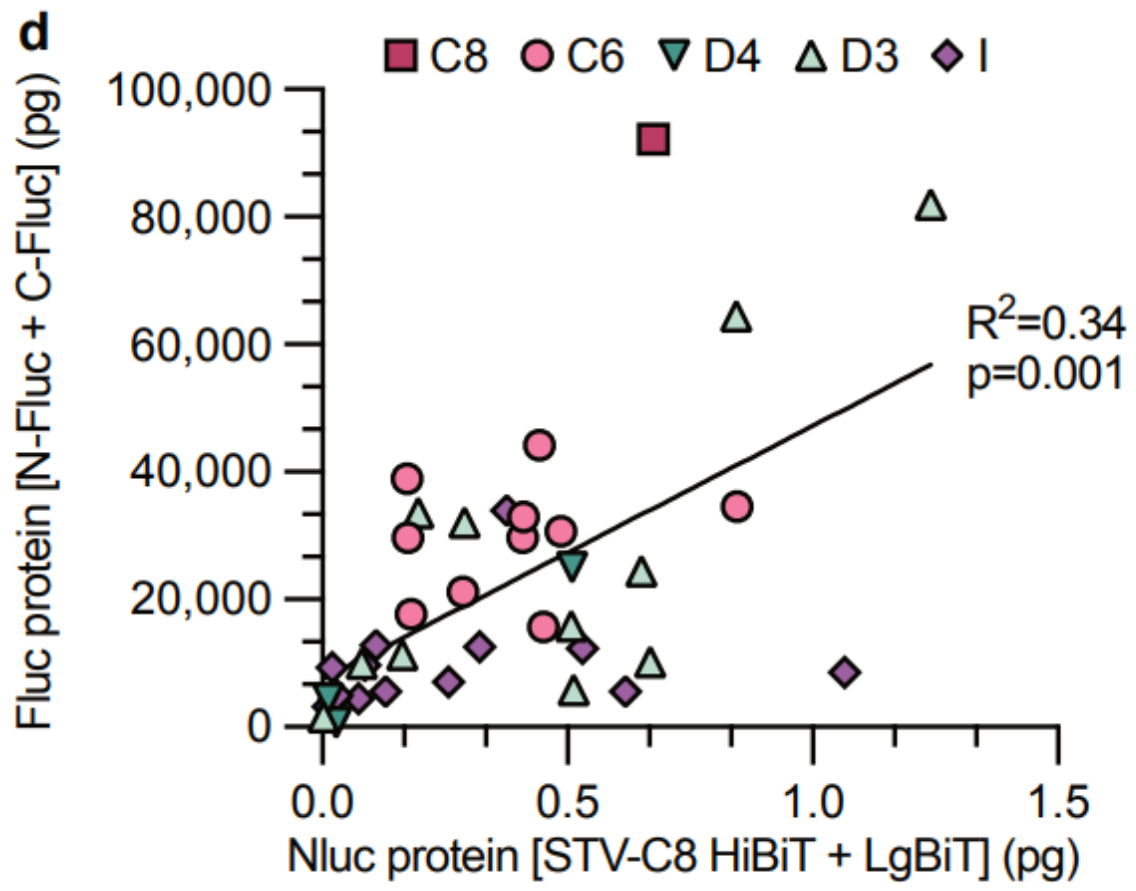

Extended Data Fig. 2 | Establishment and validation of the dual Split-Luciferase based screening method. (…) **d,** Correlation analysis of STV release into the supernatant and mRNA delivery efficiency into target cells for different STV symmetries (C, cyclic; D, dihedral; and I, icosahedral). Both dimensions are measured as total protein content by standardizing against Fluc or Nluc reference proteins (each data point shown as mean from n = 6 biological replicates) (…)

Additionally, we agree with the referee that the reporter system was not sufficiently described. To clarify this point, we added an extensive description of the reporter system in Supplementary Note 1 and revised the corresponding section in the Results: "In brief (a detailed description is provided in Supplementary Note 1), STV constructs were fused to a HiBiT tag, enabling the quantification of STV release into the supernatant by complementing HiBiT with recombinant LgBiT protein. For characterizing the uptake efficiency, we created a reporter cell line expressing LgBiT to measure STV uptake by HiBiT/LgBiT complementation in target cells. Additionally, the reporter cells expressed the C-split part of Firefly luciferase (Fluc). STV-mediated delivery of mRNA coding for the N-split part of Fluc enabled the quantification of mRNA delivery efficiency by reconstitution of full-length Fluc (Fig. 1d, Extended Data Fig. 2a-c)"

In addition, we used our reporter cells to investigate the uptake kinetics of STV-C8. Using the live-luciferase substrate Endurazine, we were able to monitor STV-C8 uptake and expression at 15-minute intervals over a period of three days. We incorporated this dataset as the new panel Extended Data Fig. 6f and added the following description to the Results section of the manuscript: "The HibiT tag, which is part of the STV-C8 construct, allowed us to additionally characterize uptake kinetics. By adding the live luciferase substrate Endurazine to STV-C8-treated Split-Luc reporter cells, we monitored the uptake of STV-C8 vesicles into target cells and found an onset after 2-4 h and an uptake maximum after 10-12 h (Extended Data Fig. 6f)"

f

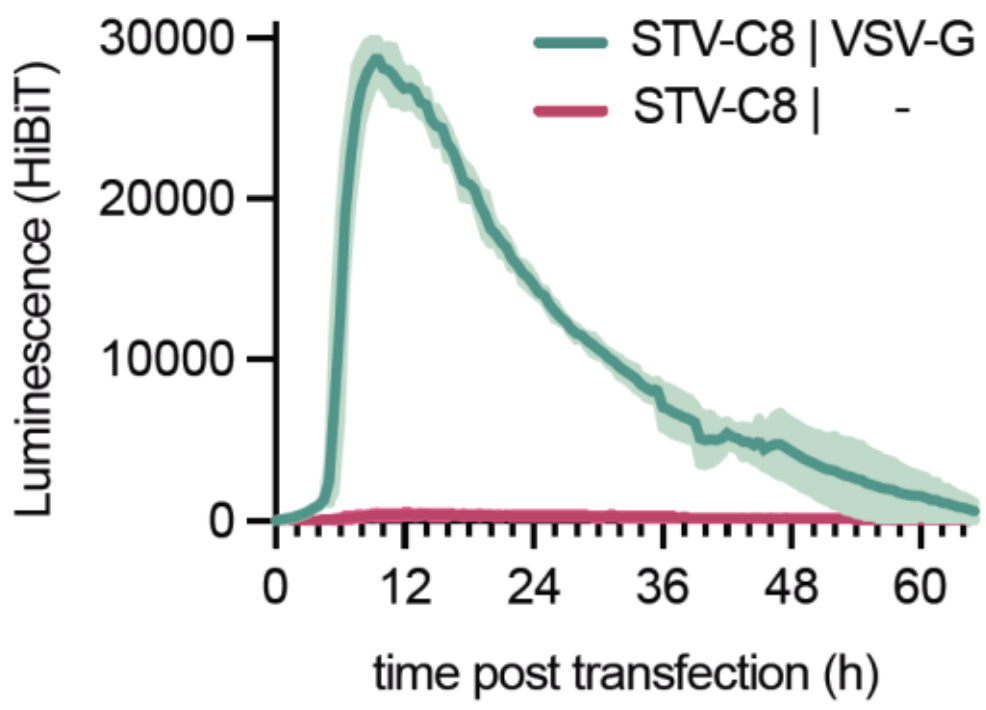

Extended Data Fig. 6 |Comprehensive characterization and benchmarking of STV-C8. a, (...) **f,** Live measurement of STV-C8 uptake kinetics into target cells by HiBiT-LgBiT reconstitution in Split-Luc target cells. (mean ± s.d. for n = 3 biological replicates).

2. Line 123: Some explanation of HE0902 nature is suggested. It would also be beneficial to the readers to present it in Figure 1A.

This point is well taken. Our intention with Fig. 1a was to present general examples of different symmetries (icosahedral, dihedral, cyclic). To clarify this, we extended the figure caption of Fig. 1a with the sentence: "Representative icosahedral (I), dihedral (D), and cyclic (C) symmetries are shown". the performance of HE0902 was limited and we subsequently focused on our optimized assembly based on HE0690, we hope the referee agrees that it is reasonable to put our initial starting point HE0902 not too much into the focus, in order to maintain a more streamlined storyline. The structure of HE0902 is nevertheless provided in Extended Data Fig. 1a. Additionally, we further specified the symmetry of HE0902 now: "As the initial scaffold for such STV carriers, we employed the well-characterized, icosahedral protein assembly HE0902 (Extended Data Fig. 1a)"

3. Line 130: RNA release into the supernatant. It is unclear how this test was performed and what conclusion can be made from this experiment. What triggers the RNA release into the supernatant, and wouldn't it be an obstacle to efficient intracellular delivery?

We apologize for the misleading wording at this point. In this context, RNA release refers to the specific packaging and active secretion of the EGFP target RNA in STV vesicles into the supernatant. Naturally, to a certain extent, EGFP mRNA can also be detected under control conditions, for example, through passive release from disrupted producer cells. However, in all conditions where STVs harbored variants of RNA-binding proteins, we consistently detected significant levels of target RNA in the supernatant, confirming their functionality. Further validation and characterization of RNA packaging and release with the final STV version, STV-C8, are presented in Fig. 2a–c. As the referee correctly pointed out, our original wording was misleading; we therefore revised the corresponding sentence to "We transfected HEK293T cells with these initial STV constructs, along with RNAs containing the corresponding packaging signal, and quantified the STV-mediated target RNA release into the cell culture supernatant". We hope this clarification resolves the inaccuracy

4. Line 183: STV-C8 vesicles enriched their cargo RNA more than 10,000-fold in the supernatant (Fig. 2b) – compared to what?

Thank you for pointing out the insufficient description of this graph. In this experiment, we compared the RNA release efficiency measured by RT-qPCR quantification of the STV-C8 assembly with and without the membrane-binding protein pleckstrin homology domain. By adding this domain we increased the amount of STV-mediated target RNA release by more than 10,000-fold (see Fig. 2b). We complemented the description in the Results section with "compared to a control missing the membrane binding domain" and updated the figure caption of Fig. 2b accordingly.

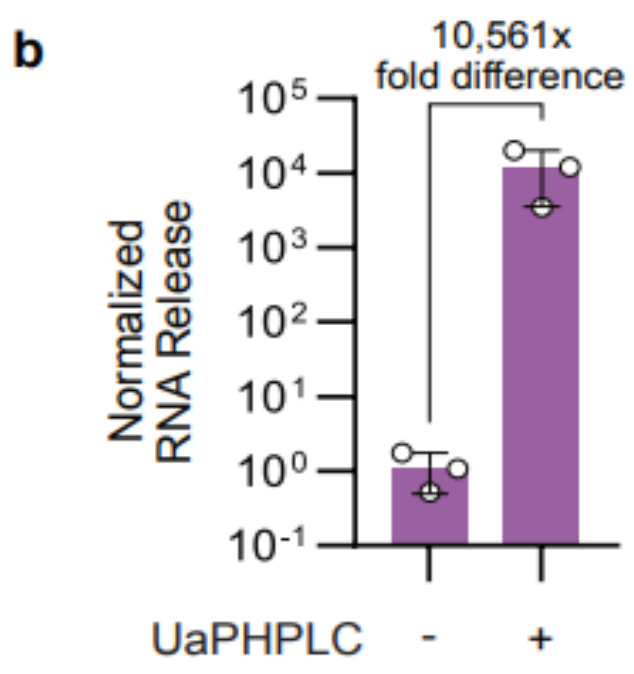

Fig. 2 | Characterization and programming of the STV-C8 RNA carrier. (…) **b,** RT-qPCR quantification of target RNA release into the supernatant by STV-C8, relative to a control construct lacking the membrane binding domain (mean ± s.d. for n = 3 biological replicates). (…)

5. Line 187: In line with previous reports, mitochondrial RNAs were depleted from STV-C8 particles, presumably because they are not accessible for packaging – are other types of cellular RNAs also packaged into the particle? If so, this could be a safety concern and will require further investigation to show there are no issues.

The referee is correct that mitochondrial RNAs were excluded from STV-C8 particles, as they are not accessible, which we demonstrate in Fig. 2c. We apologize that the role of cellular RNAs was not explained clearly enough. To address this, we extended the referring figure caption to "**c,** RNA-seq based correlation analysis of producer cell and STV-C8 RNA content (n = 4 biological replicates). For each mRNA the read count in supernatant and cytosol relative to all mRNAs is shown. Mitochondrial RNAs are colored in red and the EGFP cargo transcript in green". Importantly, this feature of STV-C8 particles is not different from other therapeutically applied particles produced in cell lines, such as lentiviruses. A more detailed analysis where we perform an enrichement/depletion analysis of RNA and protein in STVs (Extended Data Fig. 5b,c) likewise did not reveal any STV-C8–specific issues in this regard. Nevertheless, in response to this point, we substantially expanded our toxicological and immunological characterization of STV-C8 *in vitro* as well as in mice and pigs. For details, please refer to the new Extended Data Fig. 9 and Extended Data Fig. 10 and our response to question 10.

6. Line 209: Why is the comparison to LNP performed differently from comparison to other methods?

We acknowledge that our rationale for employing different benchmarking regimes was not described in sufficient detail. Genetically encoded systems such as VLPs, SEND, and EPN24 can be directly compared with the genetically encoded STV-C8 system. In contrast, LNPs are based on the *in vitro* assembly of lipids with synthetic RNA, which required us to adapt the benchmarking approach to enable proper normalization. To this end, we quantified the total RNA content within purified STVs and matched it to the corresponding amount of synthetic RNA in the LNPs.

For this revision, we further refined our benchmarking experiment to allow a more direct comparison. STV-C8 were benchmarked against clinically used Lipid Nanoparticles (LNP), formulated with the ionizable lipid ALC-0315 that is used in the COVID-19 vaccine BNT162b2[23]. We performed a serial dilution of LNP and STV-C8 packaged EGFP mRNA on target cells and found a more than 1,000-fold higher transfection rate of STV-C8 compared to LNPs. This dataset is now included as the new panel Fig. 2g. The data of the previous experiment has been moved to Extended Data Fig 6e.

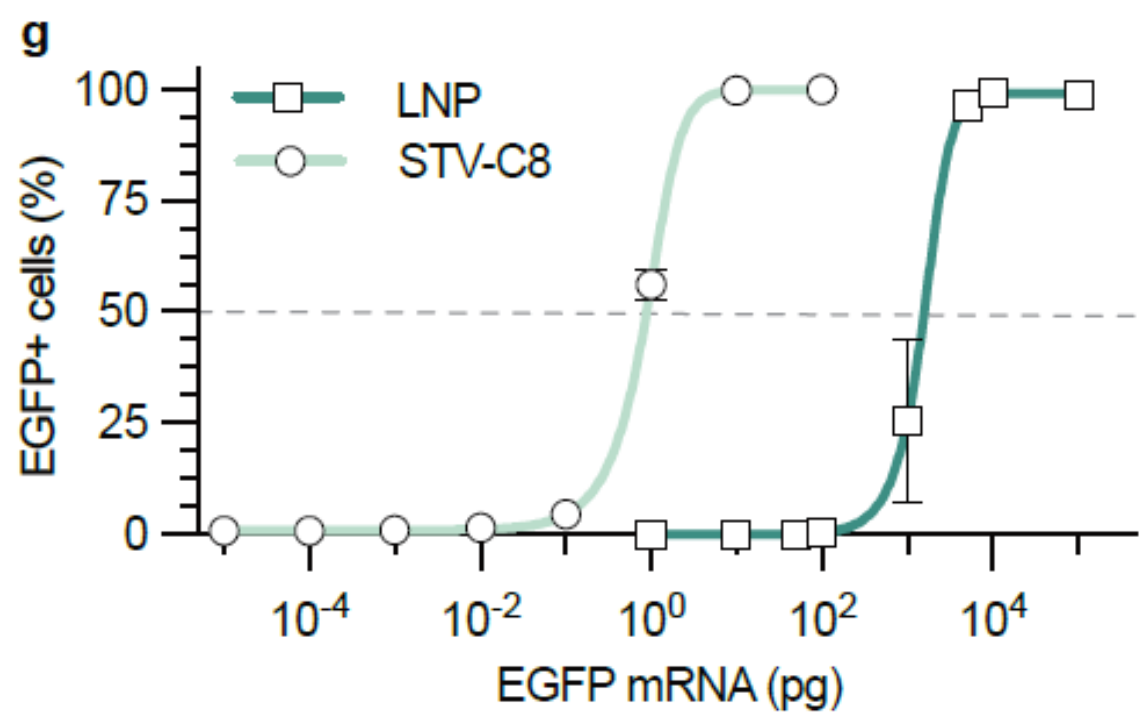

Fig. 2 | Characterization and programming of the STV-C8 RNA carrier. (...) **g,** Percentage of EGFP positive cells as a function of EGFP mRNA input delivered by STV-C8 or by LNPs. STV-C8 RNA content was quantified by absolute RT-qPCR against an in vitro transcribed reference EGFP RNA. The expression analysis was performed by Flow Cytometry (mean ± s.d. for n = 3 biological replicates).

7. Line 210: Unmodified mRNA is a potent trigger for the innate immune response – comparison to synthetic unmodified RNA is required to claim that the "biological" production alleviates the immunogenicity.

We thank the referee for bringing this important aspect into discussion. Following the referee's suggestion, we investigated whether mRNA delivered by STV-C8 or by LNPs induces an innate immune response depending on the form of the mRNA. To this end, we produced *in vitro*–transcribed mTagBFP2 mRNA either with N1- Methylpseudouridine modification or unmodified. A549-IFN-GFP reporter cells, which report interferon signaling by GFP expression, were then treated with STV-C8 containing mTagBFP2 mRNA or with LNPs carrying modified or unmodified mTagBFP2 mRNA, adjusted to achieve comparable mTagBFP2 expression levels. IFN-GFP activation was quantified by flow cytometry. Strikingly, cells treated with unmodified in vitro–transcribed mTagBFP2 mRNA showed a clear interferon response, whereas both modified *in vitro*–transcribed mTagBFP2 mRNA and STV-C8–delivered ("biologically" produced) mTagBFP2 mRNA did not induce a detectable response and were indistinguishable from untreated controls, despite exhibiting higher mTagBFP2 expression levels. We

incorporated this dataset as the new panel Fig. 2h and added the following description to the Results section of the manuscript: "We hypothesized that the biological production process of the cargo RNA alleviates the interferon response. To test this hypothesis, we transcribed mTagBFP2 mRNA in vitro (capped and polyadenylated) containing either regular uridine or N1-Methylpseudouridine. We packaged these mRNAs into LNPs and produced, in parallel, mTagBFP2 mRNA, packaged in STV-C8. We treated A549-IFN reporter cells with these samples. As expected, unmodified mRNA caused a strong activation of reporter cells, while modified mRNA and STV-C8-delivered mRNA, did not induce activation of the reporter cells (Fig. 2h)."

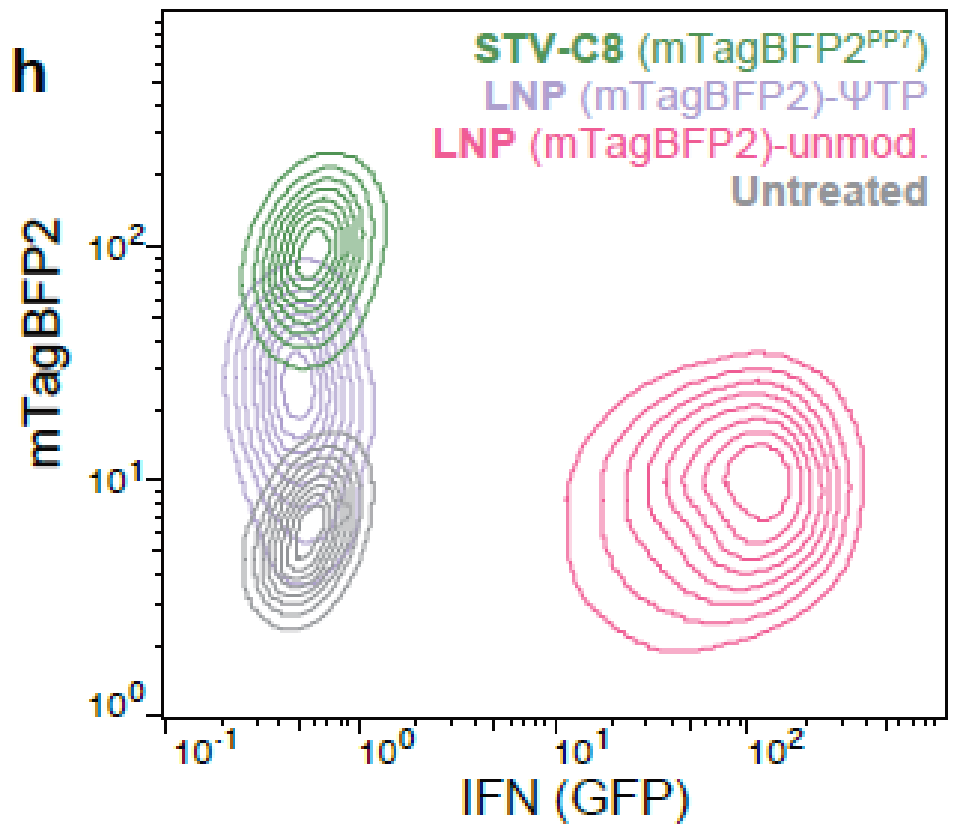

Fig. 2 | Characterization and programming of the STV-C8 RNA carrier. (...) **h,** Induction of GFP expression under the control of the IFN- β promoter in A549 reporter cells upon delivering either cellular transcribed mTagBFP2 mRNA, packaged in STV-C8 or in vitro transcribed N1-methyl Pseudo-UTP modified or unmodified mRNA, packaged in LNPs. mRNA amounts for both vehicle types were adapted to reach similar expression levels. (...)

Thanks to the referee's suggestion and the additional data, we were able to substantiate our claim that 'biologically' produced RNA indeed alleviates immunogenicity. This finding is further supported by the new immunological and toxicological data we generated for both local and systemic administration of STV-C8 in mice and pigs. For details, we kindly refer to our response to question 10.

8. Line 254: confirming their ability to penetrate deeper into the tissue – judging by the Figure, STV-C8 transduced outer layers more efficiently and didn't get deep inside of the organoid.

We agree with the referee that the extent of STV-C8 penetration into retinal pigment epithelium (RPE) spheroids, as shown in Fig. 3a,b, is not particularly high. This outcome was, to some degree, expected, since spheroid models generally lack vascularization, which limits penetration. In this specific case, the challenge is even greater: the RPE forms tight junctions that constitute the blood–retina barrier (BRB), a highly restrictive barrier for macromolecule entry. Evidence from the literature indicates that RPE spheroids also recapitulate these features, making them a particularly stringent model (*Hellinen et al., Pharmaceutics. 2020; Nishida et al., Biological & pharmaceutical bulletin, 2023*). Against this background, we find it encouraging that STV-C8, albeit at lower efficiency, was still able to mediate detectable EGFP expression in deeper layers of the spheroids. Nevertheless, we fully acknowledge that the overall level of STV-mediated expression in inner layers remains limited, with the majority of particles retained in the outer layers. To reflect this point more accurately, we have carefully moderated our statements in the manuscript from "We transduced iPSC-derived retinal pigment

epithelium (RPE) spheroids with STV-C8(EGFP) and detected EGFP expression in the inner layers of the spheroids, confirming their ability to penetrate deeper tissue (Fig. 3a,b and Extended Data Fig. 9a,b)." to "We transduced iPSC-derived retinal pigment epithelium (RPE) spheroids with STV-C8(EGFP) and observed EGFP expression also in inner spheroid layers, demonstrating that STV-C8 can mediate delivery beyond the outer cell layers (Fig. 3a,b and Supplementary Fig. 3a,b)." We hope that this clarification adequately address the referee's concern.

9. Line 295: To assess the delivery vehicle's applicability to muscular dystrophies, it is essential to ensure that the RNA is delivered to muscles and not to other cells at the location of injections. To do that, the authors can perform a western blot and show the expression of the truncated protein.

We thank the referee for this comment. The observed editing frequencies of ~8% to 40%, depending on the distance from the injection site, in a relatively homogeneous tissue such as skeletal muscle strongly suggest that Cas9 is predominantly delivered into muscle cells, although we had not directly demonstrated this in our initial experiments. Ethical restrictions on the use of large animal models prevented us from repeating this study to obtain additional material. Furthermore, since our targeting strategy relies on the removal of a single exon, Western blot analysis was not suitable for detecting such a subtle change in a very large protein like dystrophin, which spans 79 exons and has a molecular weight of 427 kDa. To directly demonstrate efficient delivery into muscle cells, we performed experiments on myotubes and observed robust transduction, with 89.6% of cells expressing the EGFP reporter. These results have been added to the manuscript as the new Extended Data Fig. 10a, together with the following statement in the Results section: "We first confirmed that STV-C8 efficiently deliver their cargo RNA into muscle cells (Extended Data Fig. 10a)."

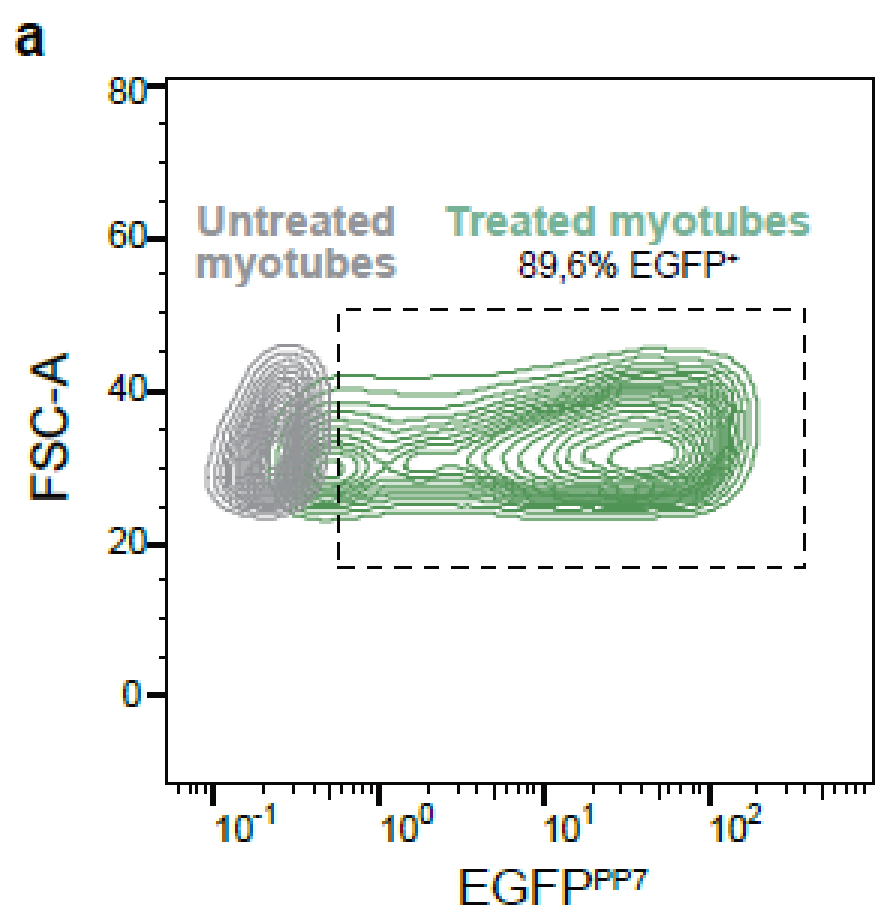

Extended Data Fig. 10 |Continued analysis of intramuscular delivery of mRNAs with STV-C8. a, Quantification of the EGFP mRNA delivery into C2C12 mouse myotubes by Flow Cytometry. (…)

10. What is missing in the paper is to verify that these RNA carriers are not causing liver damage (via examining liver enzyme release and histology of the liver) and do not induce a pro-inflammatory response by triggering pro-inflammatory cytokines, interferon responsive genes and activating lymphocytes.

We fully agree with the referee that a thorough characterization of this novel RNA carrier system with respect to toxicity and pro-inflammatory responses is essential to demonstrate its applicability. Given that the synthetic nature of this RNA transfer vehicle makes it a promising candidate for applications requiring low immunogenicity, the initial *in vitro* experiments alone were indeed insufficient to substantiate such claims. In line with the referee's suggestion, we therefore conducted new experiments to evaluate toxicity and immune responses in systemically injected mice. Specifically, 11-week-old C57BL/6J mice received 50 µL of STV-C8 or PBS as control via tail vein injection and were analyzed 1 and 3 days post-injection. Serum alanine aminotransferase (ALT) levels were measured using the ALAT (GPT) FS (IFCC mod.) assay. ALT activity in serum reflects hepatocellular damage and serves as a highly sensitive and specific biomarker of hepatotoxicity in both preclinical and clinical settings. In addition, liver cytotoxicity was assessed by LDH assay, which measures cell membrane integrity via LDH release. Together, these approaches provide reliable indicators of potential cell death and liver toxicity. We include the results of this new experimental set as Extended Data Fig. 9b, showing that both enzymes are not altered after treatment of mice with RNA loaded STV-C8 indicating that there is no liver toxicity induced. Furthermore, we studied inflammatory marker expression such as Il-6, Interferon, Ill-1, Il-10; OAS, IFNg as well as IkBa and could not detect significant alterations as well. We included this dataset as the new Extended Data Fig. 9c. Thus, these studies neither indicate any liver damage nor any induced immunoinflammatory response, therefore we included the following sentence to the Results section of the manuscript: "Additionally, we could not detect immunological or toxicological side effects of systemic STV-C8 injection (Extended Data Fig. 9)."

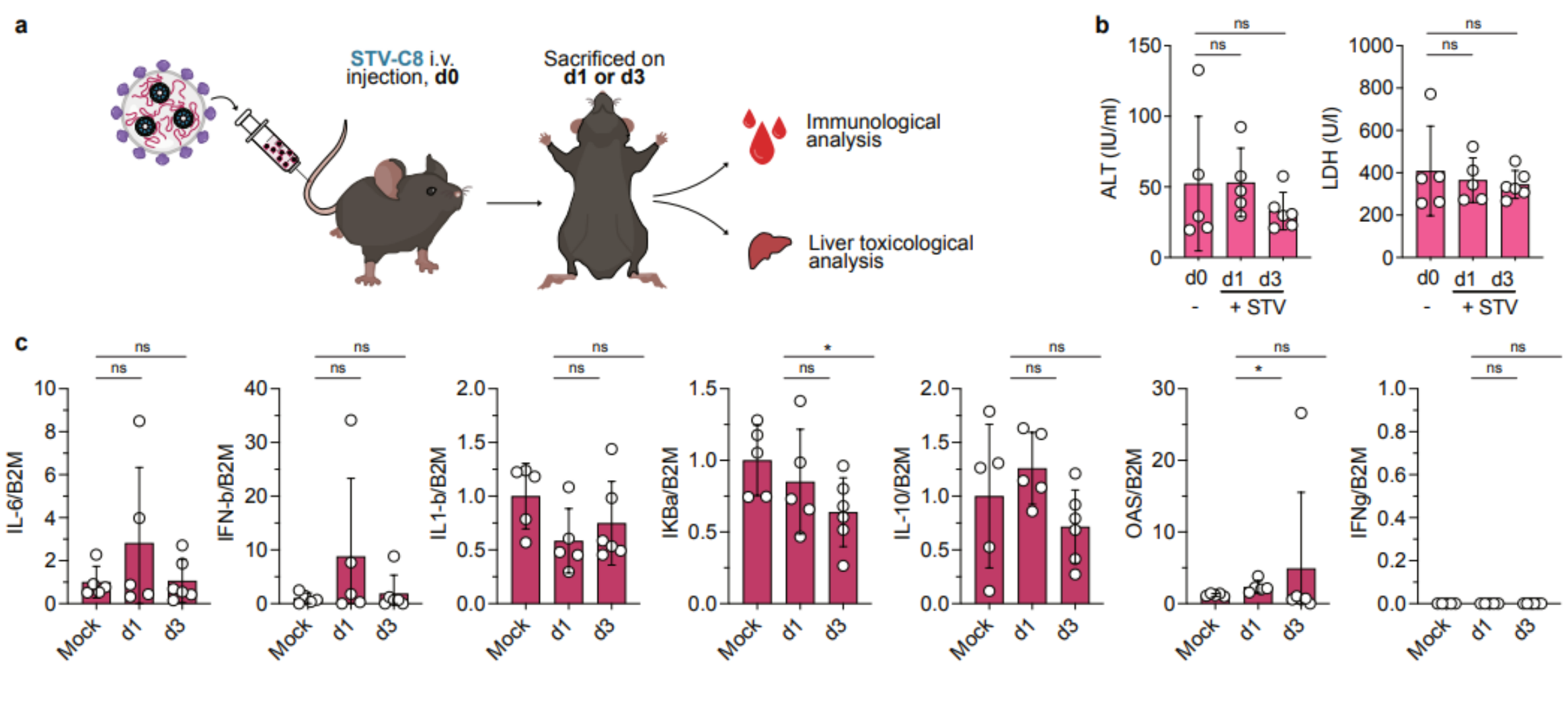

Extended Data Fig. 9 | Evaluation of STV-C8-mediated immunological response and liver damage in vivo. a, Scheme of the experimental setup for testing immunological markers in blood and liver toxicity. **b,** Analysis of potential liver toxicity (ALT, alanine aminotransferase) and broader toxicological side effects (LDH, lactate dehydrogenase) of animals, treated by systemic STV-C8 injection (mean ± s.d. for n = 5-6 treated animals). **c,** Testing for immunological markers by RT-qPCR (one-way ANOVA, *$P < 0.05$, mean ± s.d. for n = 5-6 treated animals).

In addition, to complement this acute systemic approach in mouse, we analyzed the local muscle injection of STV-C8 (Cas9/sgRNA). Via RT-qPCR we investigated different inflammatory markers like TNFa, Il-10, CyBB, CD68, CD 45, Il1 a, Il18, Il-1b, TGFb and IFNg for altered expression level. We did not

identify significant alteration of STV-C8 treated tissue compared to negative controls (uninjected muscle) while muscle samples from rejected tissue, used as a positive indicator, showed strong inflammatory response. We included these results as the new Extended Data Fig. 10d,e and extended the corresponding Results section with the following revised paragraph: "Similar to the systemic injection, we could not detect a immunological response in this local application of the STV-C8 treated muscle (Extended Data Fig. 10c-e). The successful deletion of exon 51 in a large animal model, combined with its beneficial safety profile, emphasizes the strong clinical potential of STV-C8.". We thank the referee for these comments and are convinced that these additional datapoints have strengthened the manuscript significantly.

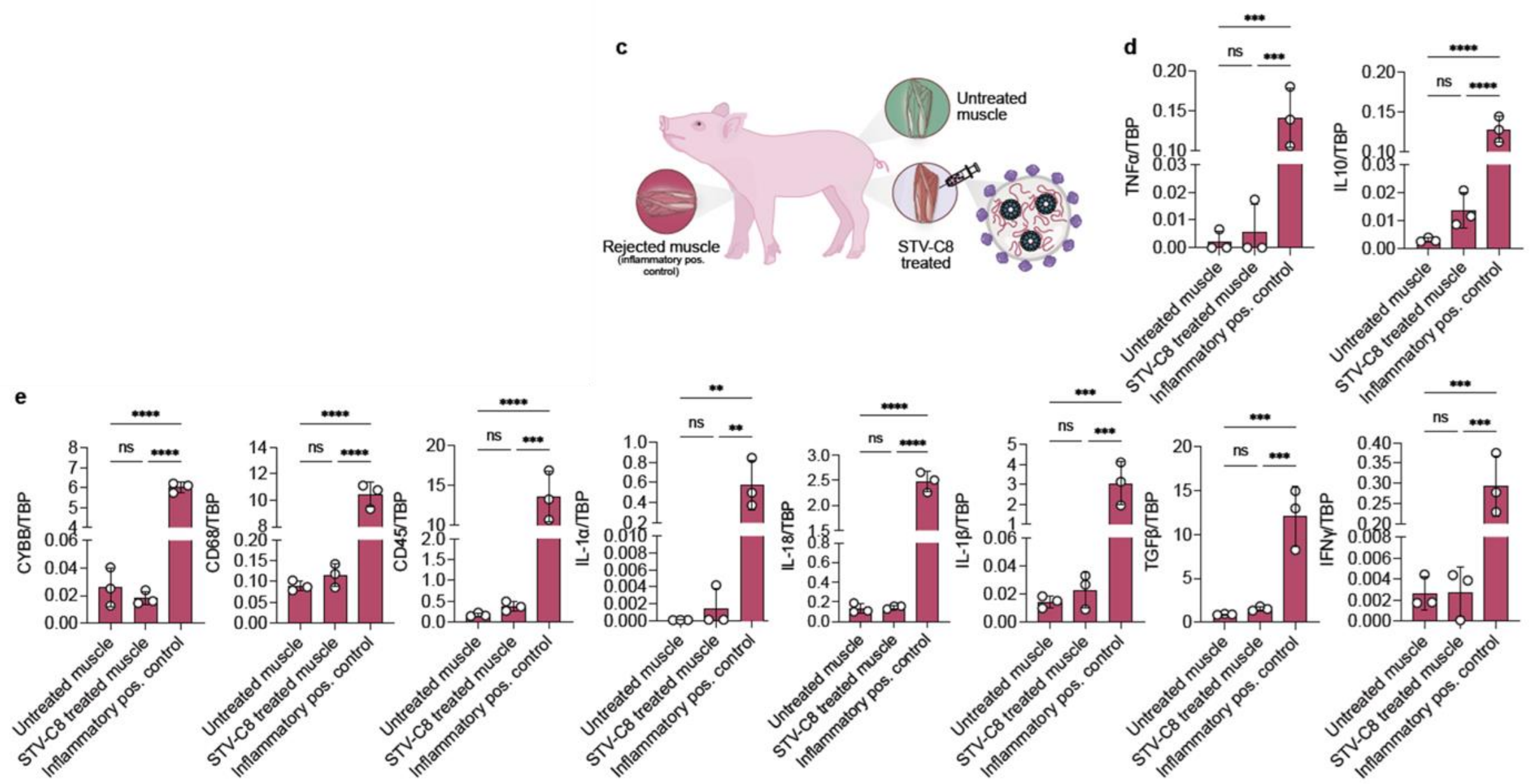

Extended Data Fig. 10 |Continued analysis of intramuscular delivery of mRNAs with STV-C8. (...) **d-e,** Analysis of inflammatory markers of muscle samples by RT-qPCR (one-way ANOVA, ** P < 0.01,*** P < 0.001, ****P < 0.0001, mean ± s.d. for n = 3 replicates of different injection sites).

We thank referee 3 for helping us to substantially improve the quality of our manuscript by adding new aspects such as an improved immunological and toxicological analysis.

Second Revision of: Creating bottom-up RNA transfer vehicles from synthetic protein assemblies (Nature 2024-09-19965A)

Response to Referees

We respond point by point to the referees' comments and include these changes in the manuscript attached. The referees' comments are colored in **blue**, while our responses are in **black**. The revised parts in the main text of the manuscript are highlighted in yellow.

Referee #1 (Remarks to the Author):

My comment on benchmarking to other delivery tools has been well addressed. The more careful quantification of the gene editing has improved the work.

The authors wish to express their gratitude to the referee for the thoughtful comments and helpful suggestions that have enhanced the quality of the paper. The following addresses the comments that are still open.

"The achievement of the AI field is not just that such sequences have some activity but that they adopt desired assembly with nearly atomic precision. In the work here there is little evidence as to the adopted assembly of the novel constructs: proposed organisation (e.g. Fig. 1a,b and extended Fig. 4a) are not well supported by the tomographic analysis in Fig. 2e."

We fully agree that one of the key achievements of the AI field is to provide precisely predicted assembly structures. To demonstrate that our assemblies indeed correspond to the proposed organization, we have substantially extended the cryo-ET analysis.

To address this question, it is necessary to visualize the assemblies in their native context within the vesicle. Determining the atomic structure of a protein within an intact vesicle is highly challenging, as conventional structural methods require isolated proteins, leaving cryo-electron tomography (cryo-ET) with advanced image processing as the only technique capable of near-atomic in situ visualization. The cryo-ET data was crucial for characterizing the vesicles and determining their size distribution. It also enabled us to estimate the proportion of vesicles containing complexes and RNAs, as well as the quantity of complexes per vesicle, and to visualize their spatial distribution. This information, which is specific to a single vesicle, could not be obtained using indirect measurement methods. However, cryo-ET does not provide atomic resolution as X-ray crystallography, single-particle (SPA) cryo-EM or AI models. Realistically, we could obtain a structure with a resolution of ~10 Å after extensive data collection. Nevertheless, this would not be sufficient to confirm the predicted AI model with confidence. While a higher resolution structure is theoretically feasible as demonstrated by highly specialized studies recently published in *Nature* (Huang, Y., *et al.*, *Nature* **633**, 224–231 (2024); Wagner, J., *et al., Nature* **633**, 459–464 (2024); Zheng, W., *et al., Nature* **631**, 232–239 (2024)), which use high-resolution in situ cryo-electron tomography and image processing to visualize protein complexes in their native membrane context and resolve near-atomic functional architectures of chaperonins, coronavirus replication organelles, and mammalian

respiratory supercomplexes, it would require a substantial effort well beyond the scope of the present work.

Near-atomic resolution is, however, not required to validate the proposed organizational model. To gain deeper insight into the structural architecture of the STV-C8 oligomer within released vesicles, we refined the reconstructed vesicle tomograms with the corresponding AlphaFold3-predicted structure and subsequently segmented the encapsulated RNA to reveal its organization within the vesicles. The positioning of the model generated by AlphaFold within the segmentation is consistent in terms of dimensionality. Firstly, while we do not have access to the fine details, the size corresponds well to that of the observed complex. Secondly, the complexes can also be plausibly paired, even though the atomic details have not been determined. The consistent dimensions of the segmented complexes, along with the systematic presence of observable RNA strands for each complex, confirm their nature and rule out the presence of other complexes of an equivalent size that would also be encapsulated. We included this new dataset as Extended Data Figure 6f.

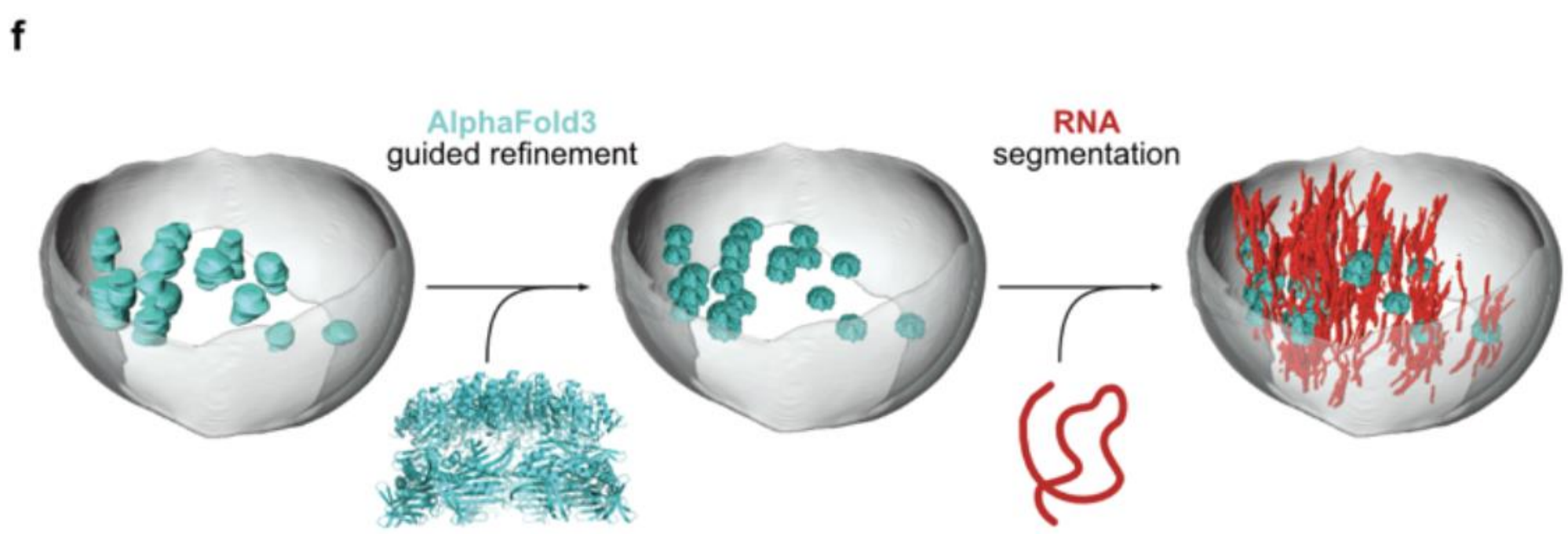

Extended Data Fig. 6 | Comprehensive characterization and benchmarking of STV-C8. (…) **f,** Segmentation of an STV-C8 vesicle tomogram, showing the STV-C8 oligomer with a docked AlphaFold3-predicted atomic model, as well as the segmentation of the RNA. (…)

It is very common that oligomerization domains change their multimeric state upon fusion to different components.

While fusion to heterologous domains or changes in structural context can in some cases influence quaternary assembly, many oligomerization domains are intrinsically robust and preserve their defined multimeric state across diverse fusion constructs and functional contexts, a feature widely exploited in protein engineering and synthetic biology (e.g. Ramšak, M., Ramirez, D.A., Hough, L.E. et al., 2023, *Nat Commun* 14, 7973).

To exclude the possibility that, in the context of the optimized STV-C8 domain architecture, fusion of additional functional elements perturbs the native oligomeric assembly of the oligomerization domain, we extended our analysis of the synthetic protein assemblies at multiple levels. First, we performed an *in silico* assessment of the oligomeric state using state-of-the-art AI-based structure prediction tools, employing the full STV-C8 domain composition as input. Notably, the resulting predictions closely resemble those obtained for the isolated oligomerization domain and strongly favor the formation of defined multimeric assemblies, indicating that fusion of additional domains is unlikely to disrupt the

intrinsic oligomeric state of the oligomerization module within the complete STV-C8 construct. AlphaFold3-based prediction of the HE0690 oligomeric state demonstrate that only the 8-mer configuration show pronounced ipTM values well above the considered confidence threshold of 0.8. This analysis is now included as the new Supplementary Figure 2.

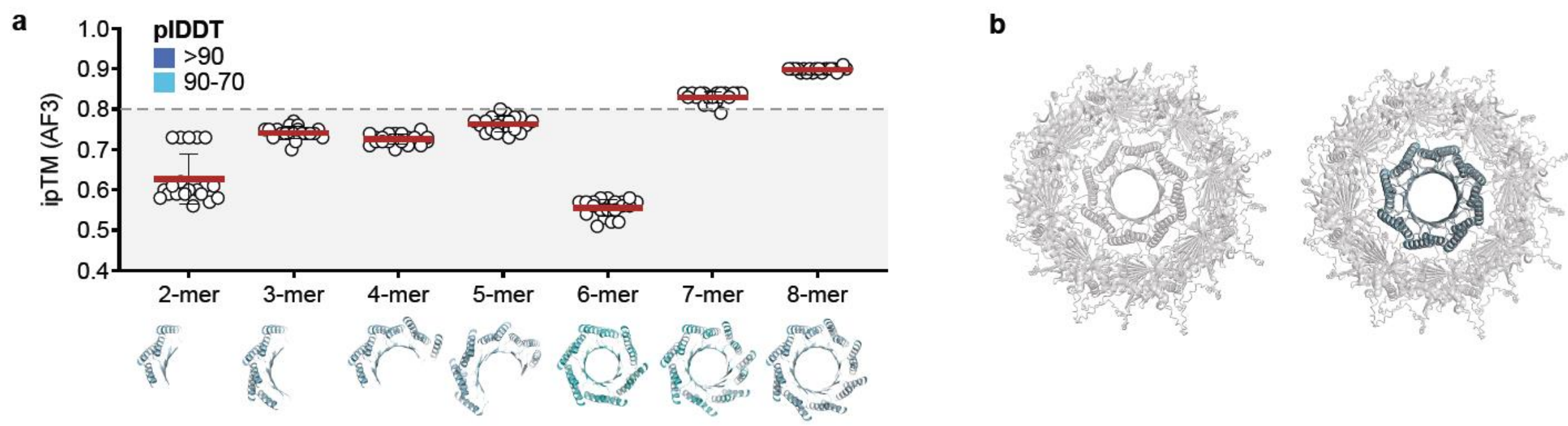

Supplementary Fig. 2 | Modelling of the STV-C8 structural features. a, AlphaFold3-based prediction of the HE0690 oligomeric state. An ipTM threshold of 0.8 is considered as highly confident. **b,** Structural alignment of the predicted HE0690 scaffold (blue) with STV-C8 (grey).

To further support our findings with additional experimental evidence, we performed a native PAGE analysis of purified STV-C8 and included the results as the new Extended Data Fig. 6e. The data confirms the presence of high-molecular-weight assemblies consistent with the predicted size of the STV-C8 multimer.

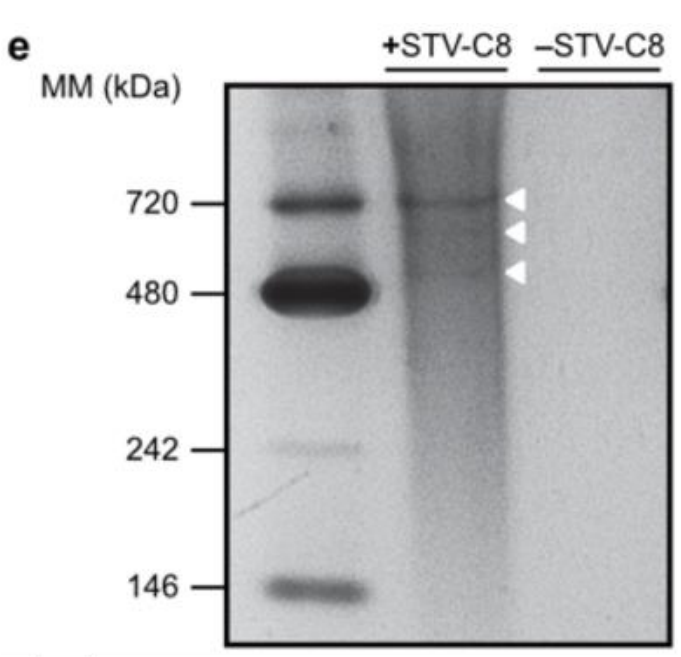

Extended Data Fig. 6 | Comprehensive characterization and benchmarking of STV-C8. (…)

e, Native PAGE of concentrated supernatants from STV-C8–transfected and untransfected cells. High–molecular-mass assemblies are indicated by white triangles. (…)

Combined with our refined cryo-ET analysis presented above, these results strengthen our confidence that the overall structure of the STV-C8 assemblies corresponds to the predicted conformation. We thank the referee for raising this point, which prompted us to refine this part of our analysis, and we are confident that our revisions satisfactorily resolve the concern.

The tomographic image in Fig. 2e lacks sufficient resolution or controls to assign the density to the intended assemblies.

This point is well taken, and we apologize for the oversight. We have additionally performed a comprehensive analysis of a cryo-ET dataset comprising concentrated supernatants from STV-C8–transfected and untransfected cells. As expected, cells not transfected with STV-C8 showed no detectable vesicles, whereas cells transfected with STV-C8 released abundant vesicles into the supernatant. We included this important control condition as the new Extended Figure 6a in the revised manuscript.

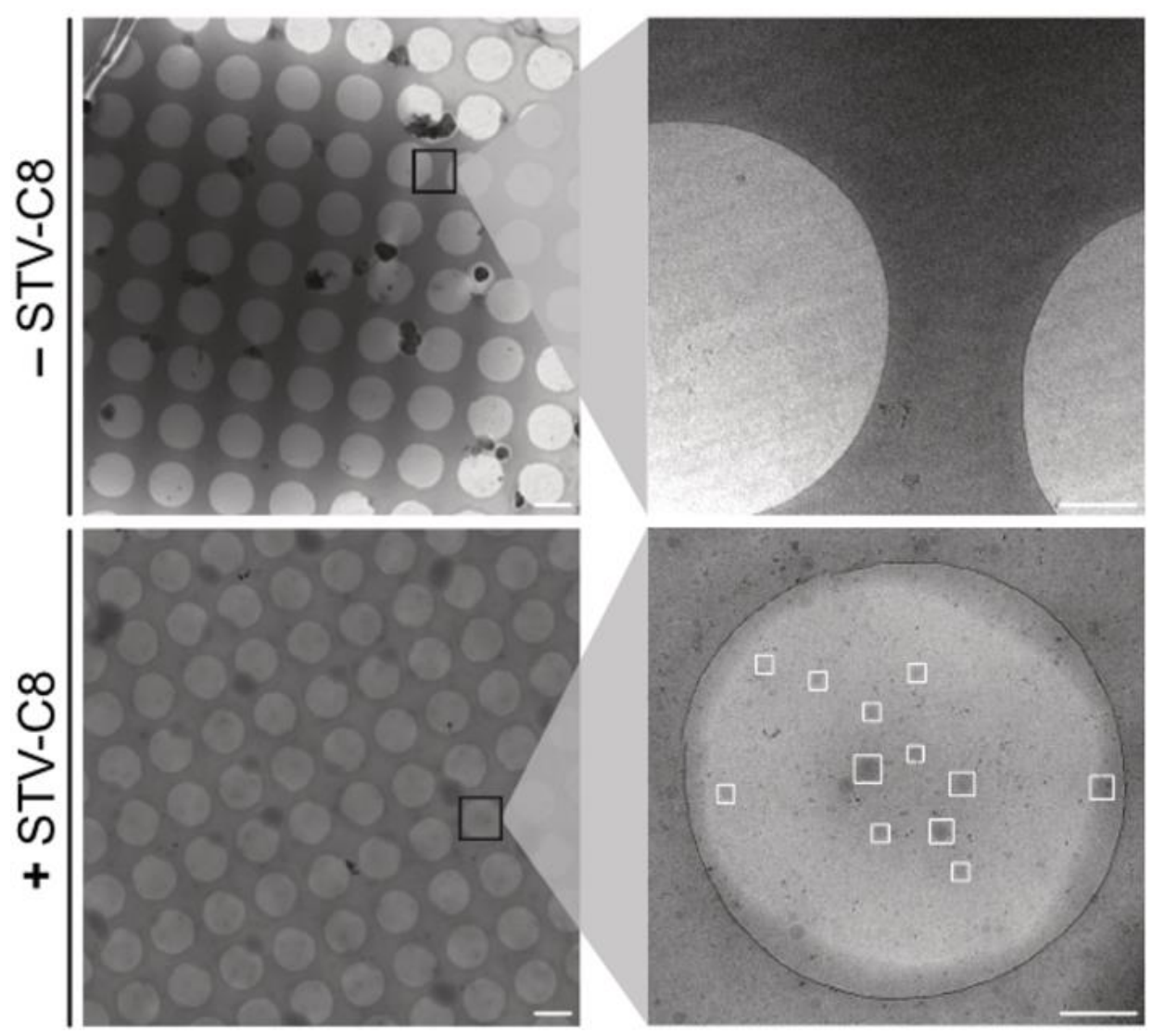

Extended Data Fig. 6 | Comprehensive characterization and benchmarking of STV-C8.

a, Search map on the grid (scale bar: 3 µm) and zoomed-in region (scale bar: 500 nm) of concentrated supernatants from STV-C8 transfected or untransfected cells**.** Representative STV-C8 vesicles are indicated by white squares. (…)

Vesicles will often contain cell components other than those explicitly directed for packaging.

To address this point, we carefully examined our cryo-ET dataset for additional cellular components. While the vast majority of STV-C8 vesicles contain complexes and RNA, occasional vesicles lack visible complexes but contain RNA, small proteins, and actin filaments. All visible complexes, besides these exceptions, are similar in size and shape (see Rebuttal Figure 1). However, the population of protein complexes within the vesicles is homogeneous.

These results make it unlikely that the observed assemblies correspond to other cellular components. Along with the newly added cryo-ET data, this additional evidence provides the referee with compelling proof that the analyzed particles represent bona fide STV-C8 assemblies.

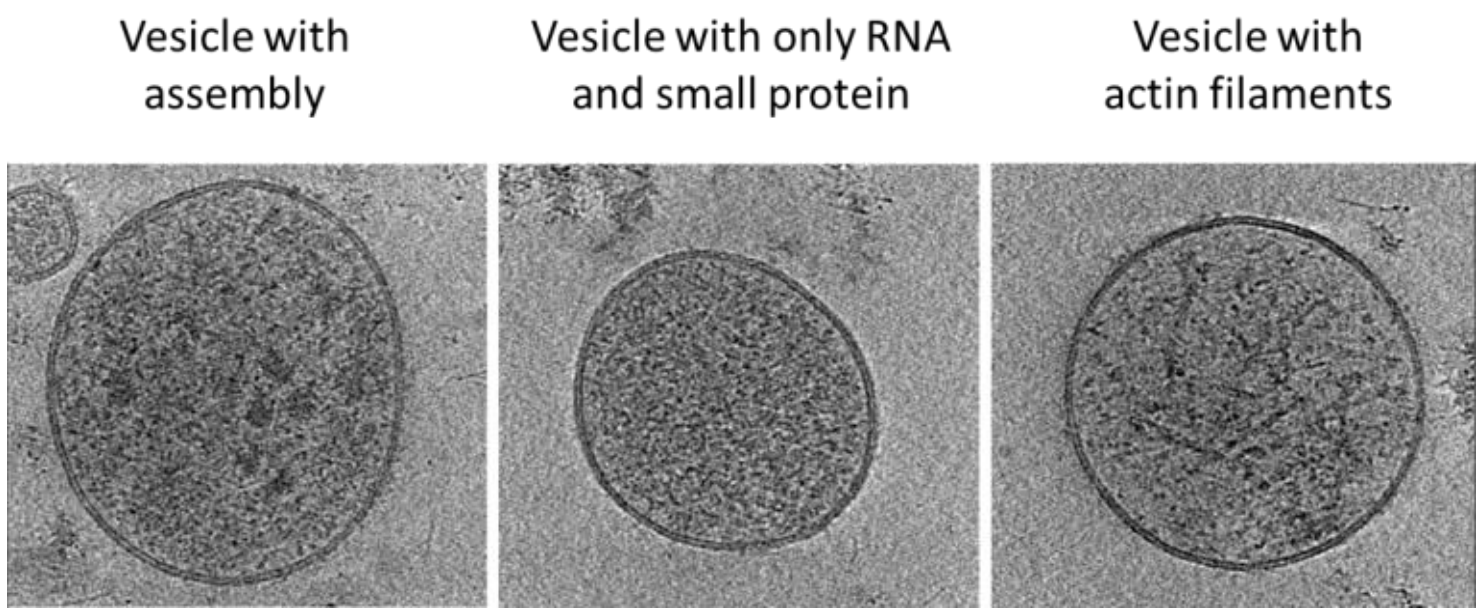

Rebuttal Figure 1. Representative examples of correctly formed vesicles containing assemblies, alongside vesicles containing RNA, small proteins, and actin filaments (rare).

I am not persuaded that tests of transduction of an immortalized muscle cell-line convey the important challenge of therapeutic delivery to real skeletal muscle.

This point is well taken. To address this shortcoming, we performed additional experiments and generated new data using a highly pertinent cellular system—skeletal muscle cells derived from control and DMD patient-specific iPSCs. Specifically, hiPSC-derived myoblasts were differentiated into myotubes and subsequently treated with STV-C8 loaded with GFP mRNA. We demonstrate that, in human skeletal muscle cells, STV-C8 efficiently transduces GFP into differentiated, multinucleated myotubes (see the new Extended Figure 10g and the new Supplementary Figure 6a).

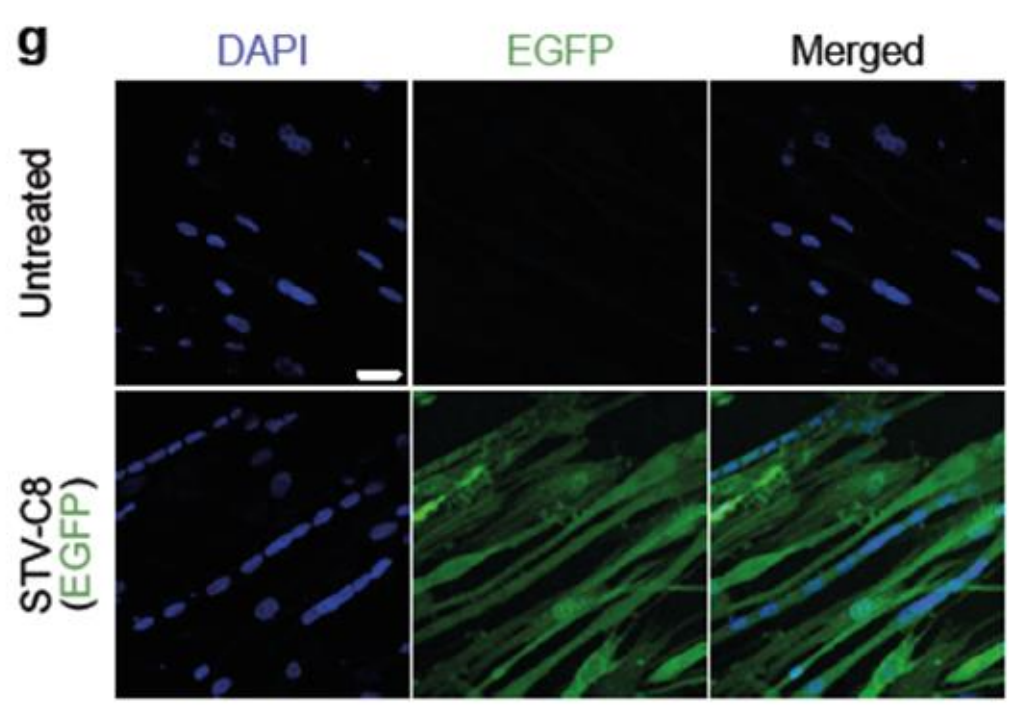

Extended Data Fig. 10 | Continued analysis of intramuscular delivery of mRNAs with STV-C8. (...) **g,** Imaging of STV-C8(EGFP) treated myotubes (scale bar: 25 µm). (...)

Robust endogenous GFP expression indicates high transduction efficiency and remains detectable for at least 72 hours. Immunocytochemical (ICC) analysis clearly confirms the differentiated myotube state of the transduced cells, as evidenced by their characteristic multinucleation.

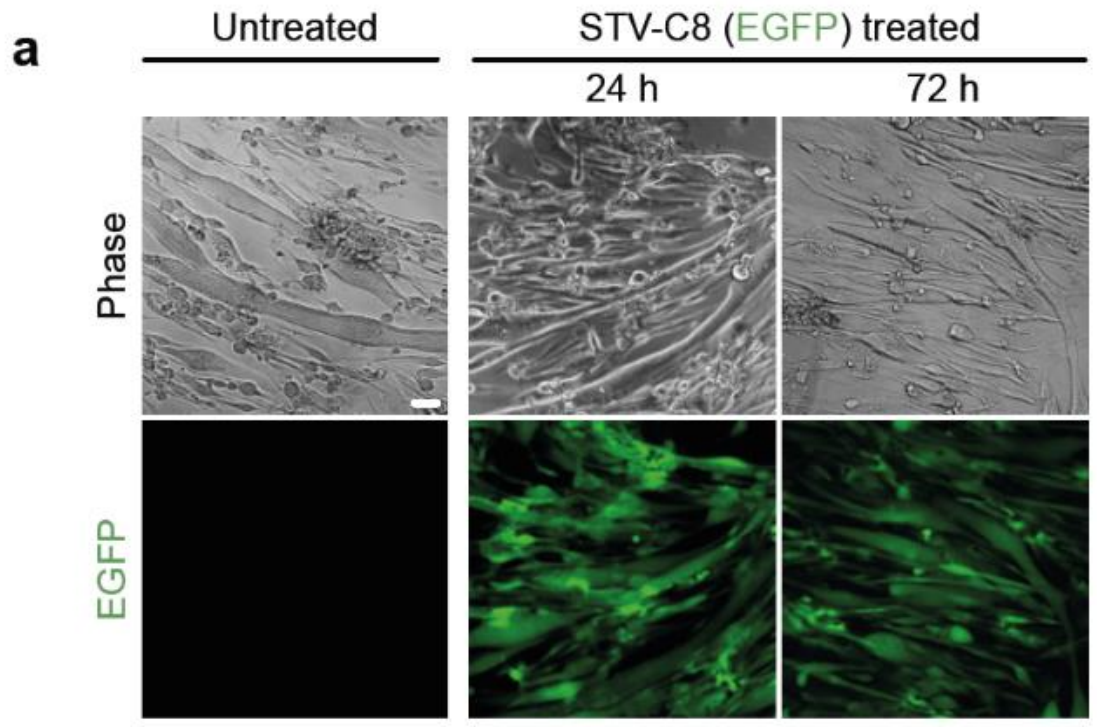

Supplementary Fig. 6 | Extended analysis of STV-C8 treated myotubes. a, Live imaging of STV-C8(EGFP) treated myotubes (scale bar: 25 µm). (...)

Moreover, we treated patient-derived skeletal myotubes with STV-C8 carrying a CRISPR/Cas9 system designed to delete exon 51 of the dystrophin gene, closely mirroring our porcine in vivo approach. In myoblasts derived from a DMD patient harboring a deletion of exon 52, STV-C8–mediated excision of exon 51 restores the dystrophin reading frame and leads to robust re-expression of a truncated but reframed, functional DMD mRNA (see the new Extended Figure 10f,h and the new Supplementary Figure 6b). These data demonstrate that STV-C8 enables efficient CRISPR/Cas9 delivery and functional exon reframing in human patient–derived skeletal muscle cells, underscoring the translational relevance of this strategy for DMD gene therapy.

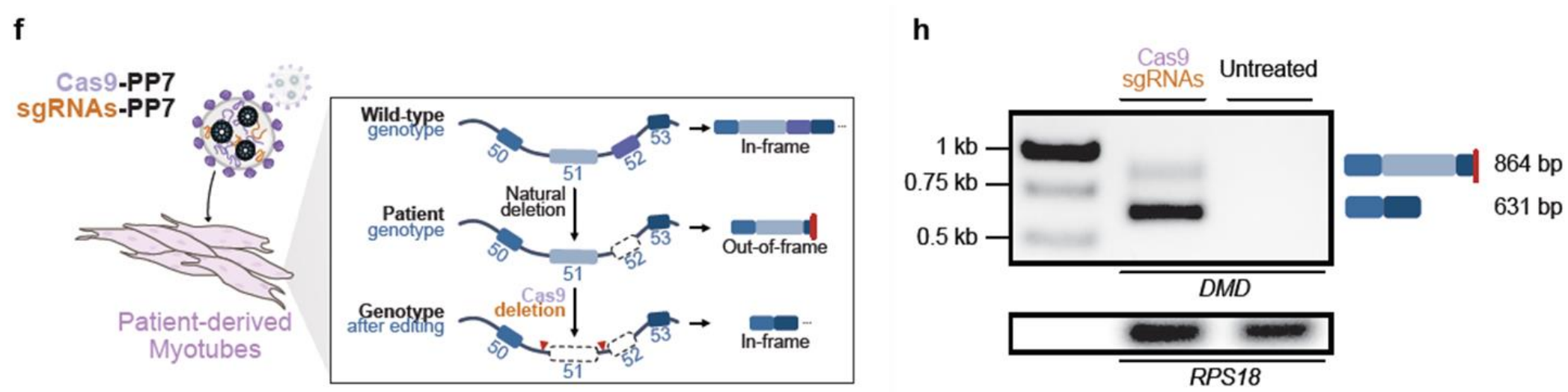

Extended Data Fig. 10 | Continued analysis of intramuscular delivery of mRNAs with STV-C8. (...) **f,** Schematic overview of the experimental setup and genotypes of edited DMD patient-derived myotubes. (...) **h,** Size analysis of amplified *DMD* exon 49-54 cDNA obtained from treated and untreated DMD patient-derived myotubes.

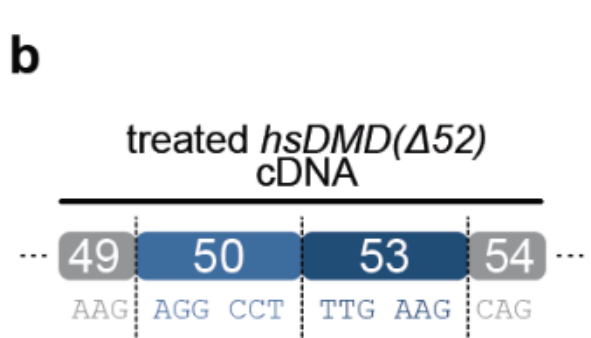

Supplementary Fig. 6 | Extended analysis of STV-C8 treated myotubes. (...) **b,** Exon-exon junction analysis of cDNA derived from edited patient-derived myotubes by nanopore sequencing.

In response to the reviewer's comment, we substantially extended the study by generating new data in a highly relevant human system using control and DMD patient–derived iPSC skeletal muscle cells. We think that these additional experiments demonstrate efficient STV-C8–mediated transgene delivery and CRISPR/Cas9-based exon reframing in differentiated, multinucleated human myotubes, significantly strengthening both the biological relevance and translational impact of the work.

Overall my assessment is that there is some good biological performance demonstrated but the molecular assembly strategy would not surprise those in the field; together with the lack of molecular validation of the intended assemblies I would not recommend publication in Nature.

We have added new cryo-ET data revealing the molecular architecture of STV-C8–containing vesicles and their association with the RNA payload. For gene-editing applications, we introduced a new human model system using skeletal muscle cells derived from control and DMD patient-specific hiPSCs, demonstrating the therapeutic potential of STV-C8. These new datasets substantially strengthen the manuscript, and we anticipate that, given the superior performance of our approach compared to existing delivery methods demonstrated in our benchmark experiments, STV-C8–based RNA transport vehicles will have broad applicability, such as in RNA-based therapies. We believe this study will be of strong interest to a broad readership.

Minor points:
Dynamic light scattering graphs should substitute size for radius or diameter, for less uncertainty.

Thank you for this suggestion. We changed "Particle size" to "Particle diameter" in the Extended Data Figure 6b.

In vivo imaging would preferably spell out the meaning of grayscale and green coloring in each legend.
We updated the respective description in line with a previous publication of this technique (Cai, R., Kolabas, Z.I., Pan, C. et al., 2023, *Nat Protoc* 18, 1197–1242) as *Tissue autofluorescence* for grayscale and *EGFP* for green (640 nm, 647-boosted GFP signal) in the figure legends of Figure 4 and Supplementary Figure 5.

Referee #2 (Remarks to the Author):

the authors have addressed my concerns.

We would like to thank the referee again for the valuable comments and suggestions which prompted us to improve the work at several points. We are happy to have addressed all your concerns to your satisfaction.

Referee #3 (Remarks to the Author):

The authors have address nicely my concerns.
Please find below my extra comment(s) for each concern:

The questions, comments, and suggestions received to date have been gratefully acknowledged. We are pleased to have addressed all concerns raised to this point. Below, we would like to respond to the comments that remain open.

Comment 5:

I appreciate the effort to modify this figure.

I would be grateful if it was possible to clarify (and point out in the main text) the means of comparing the amount of particles/mRNA when benchmarking genetically encoded systems, i.e. was the concentration based on the volume of supernatant collected from producer cells or was the number of particles evaluated or was the amount of RNA within the particles evaluated? The same question can be extended to Fig.1h.

In addition, it wasn't mentioned what type of cells were transfected for the LNP benchmarking. For the purposes of uniformity, I would strongly suggest comparing LNP and STV-C8 on the same cell lines as shown in Fig.2f.

We apologize for the lack of clarity regarding these points. In the benchmarking experiment (Fig. 2f), we indeed used identical volumes of purified supernatant. To make this clearer, we modified the corresponding sentence in the main text as follows: "We cloned the coding sequence of each system into a CAG promoter-driven plasmid backbone, transfected identical amounts of VSV-G, cargo, and packaging plasmid into HEK293T cells, collected the supernatant for three consecutive days, concentrated the supernatant, and added the processed samples in identical volumes to the target cells."

The benchmarking against LNPs (Fig. 2g) was performed in HEK293T cells and therefore used the same cell line as, among others, in Fig. 2f. We have added this information to the respective description: "We performed a serial dilution of LNP and STV-C8 packaged EGFP mRNA on target HEK293T cells and found a more than 1,000-fold higher transfection rate of STV-C8 compared to LNPs (Fig. 2g)."

Thank you for pointing out these shortcomings. We hope that these changes have clarified the issues.

Comment 8:

The addition of myotubules to the list of cell models is beneficial, since they are known to be hard to transfect. Please note that there is no indication of the concentration of STVs used in the methods section. Indeed, showing a truncated protein compared to wild-type by means of western blot would not be an option in this case and I understand the difficulties related to the use of large animals. However, I believe that this experiment is not demonstrating the clinical applicability of STV-C8 to MDs to its full potential. If it would be possible, I would suggest using cDNA to demonstrate that the deletion happened in cells that

actually express dystrophin. If it is not possible, I would suggest highlighting some limitations in the applicability of this approach to DMD: 1) i/m injections are currently not applicable to DMD due to cardiac and diaphragm involvement; 2) judging by the mouse experiments, the non-targeted STV do not show muscle biodistribution, so they wouldn't be ideal to systemic injection; 3) since many other delivery vehicles are capable of penetrating the muscle upon i/m injection, they will need to be compared in the future; 4) cell-level specificity analysis would be required in the future. I agree that these initial experiments are nonetheless valuable, but I suggest altering the wording for a more realistic evaluation.

We fully agree with the referee on this point. While the initial large-animal experiment is valuable, it has inherent limitations. As stated previously, ethical restrictions on the use of large animal models prevented us from repeating this study to obtain additional material. To address these limitations, we therefore expanded the manuscript by including an additional, highly relevant human model system using hiPSCs differentiated into skeletal myotubes. Using STV-C8 loaded with GFP mRNA, we demonstrate efficient transduction of myotubes derived from control hiPSCs. Treatment with higher doses of STV-C8 resulted in robust endogenous GFP expression detectable after 24 hours and persisting up to 72 hours post-transduction, while lower doses also produced strong GFP expression at 24 hours with a modest decline by 72 hours. Subsequent fixation and DAPI staining confirmed that the transduced cells were multinucleated myotubes. These data are included as the new Extended Figure 10g and the new Supplementary Figure 6a.

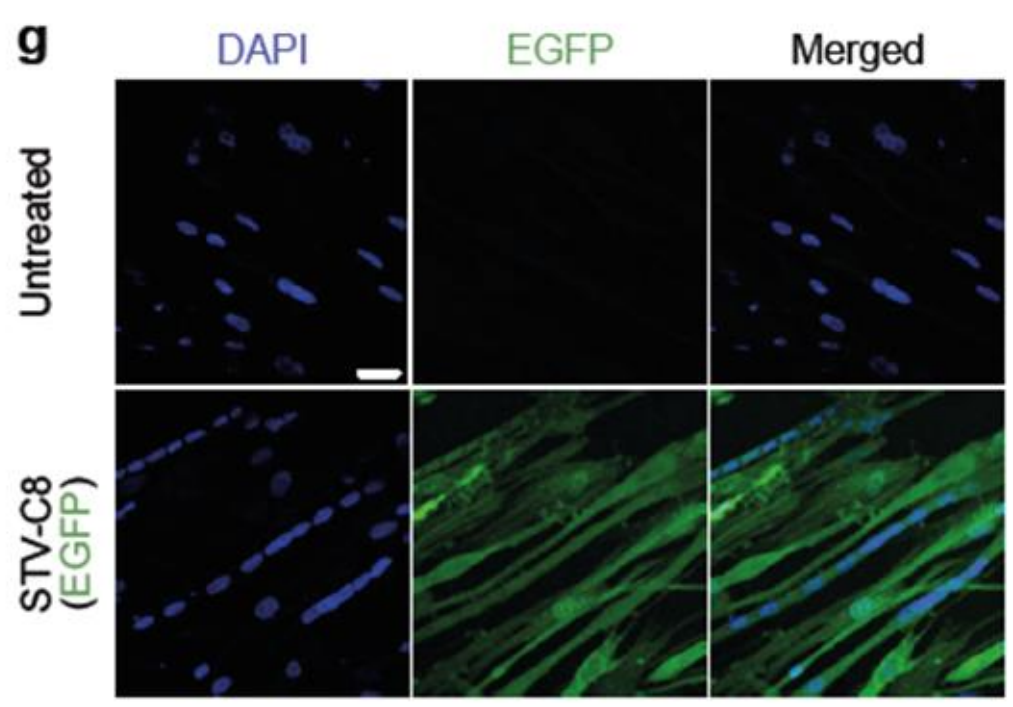

Extended Data Fig. 10 | Continued analysis of intramuscular delivery of mRNAs with STV-C8. (…) **g,** Imaging of STV-C8(EGFP) treated myotubes (scale bar: 25 µm). (…)

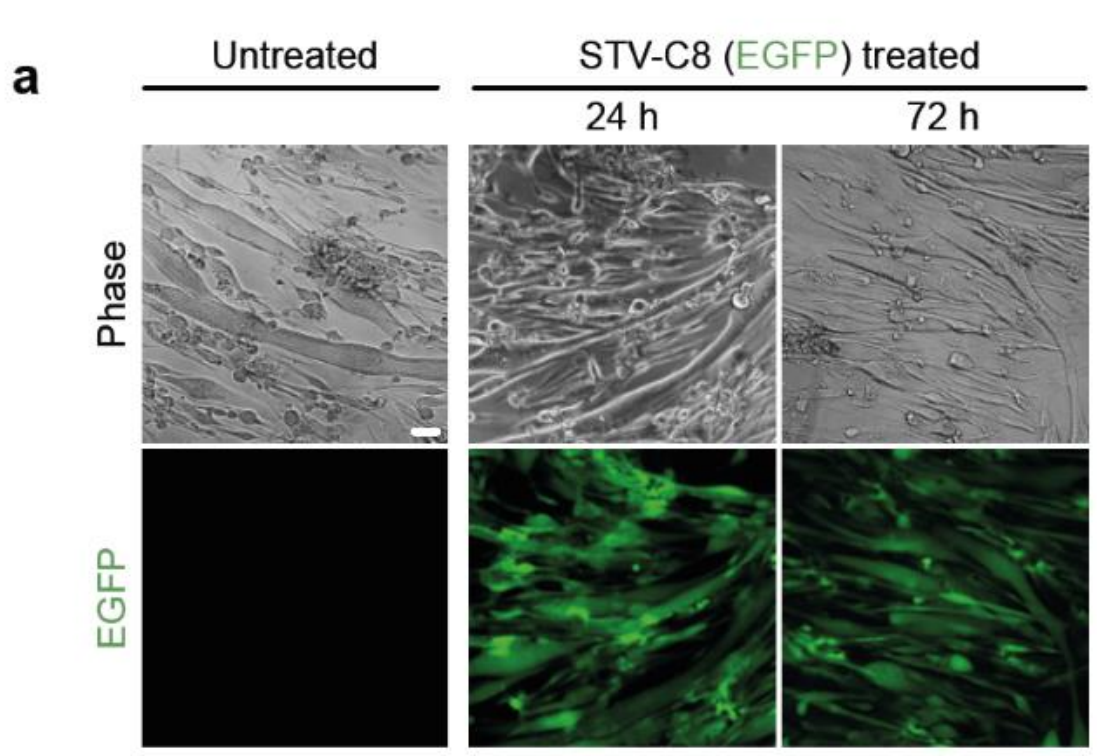

Supplementary Fig. 6 | Extended analysis of STV-C8 treated myotubes. a, Live imaging of STV-C8(EGFP) treated myotubes (scale bar: 25 µm). (…)

To address the referee's suggestion to demonstrate gene editing in dystrophin-expressing cells, we next applied a gene-editing strategy analogous to that used in the porcine model. STV-C8 particles loaded with Cas9 mRNA and two gRNAs targeting DMD introns 50 and 51 were used to treat myotubes differentiated from hiPSCs derived from a DMD patient harboring a deletion of exon 52. As expected, due to the resulting frameshift mutation, dystrophin expression was undetectable -even at the RNA level - in untreated patient-derived cells. Following STV-C8 treatment and successful excision of exon 51, we observed robust re-expression of a truncated but reframed DMD transcript. Nanopore sequencing confirmed the expected splice pattern. This new dataset is presented in the new Extended Figure 10f,h and the new Supplementary Figure 6b.

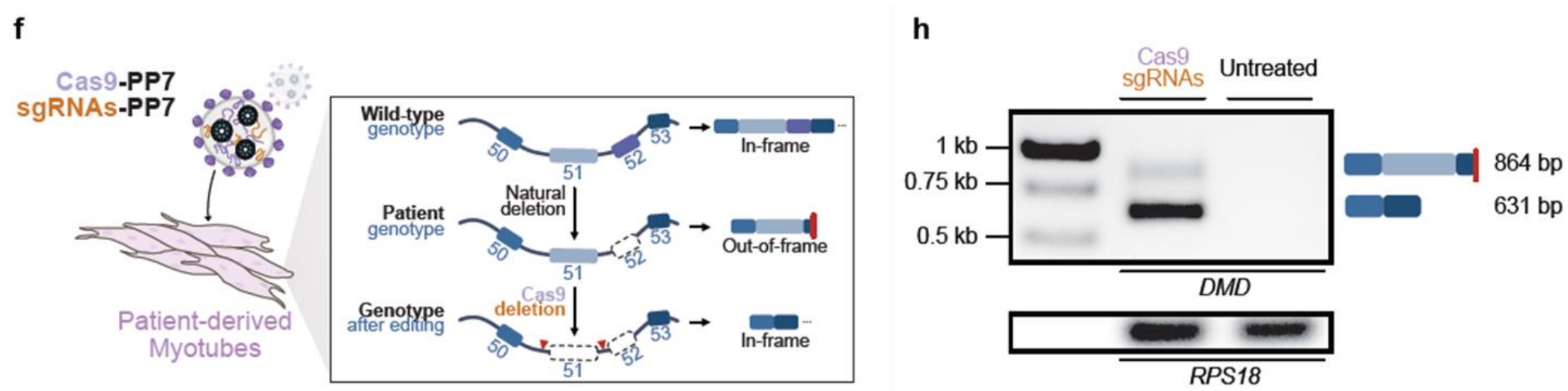

Extended Data Fig. 10 | Continued analysis of intramuscular delivery of mRNAs with STV-C8. (...) **f,** Schematic overview of the experimental setup and genotypes of edited DMD patient-derived myotubes. (...) **h,** Size analysis of amplified *DMD* exon 49-54 cDNA obtained from treated and untreated DMD patient-derived myotubes.

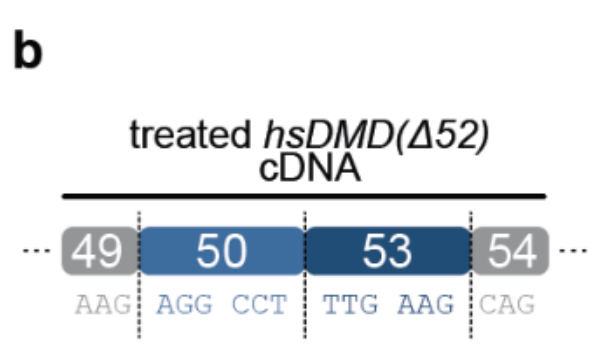

Supplementary Fig. 6 | Extended analysis of STV-C8 treated myotubes. (...) **b,** Exon-exon junction analysis of cDNA derived from edited patient-derived myotubes by nanopore sequencing.

Although these new data confirm and extend our initial in vivo findings in the porcine model, we agree with the referee that the limitations of the current study should be clearly acknowledged. Accordingly, we revised the relevant section of the Results as follows:

"While acknowledging that intramuscular delivery is not directly applicable to DMD and that future studies will need to address comparative performance, targeting, and cell-type specificity, the successful deletion of exon 51 in a large animal model and DMD patient-derived skeletal muscle cells, combined with its beneficial safety profile, emphasizes the strong clinical potential of STV-C8."

We believe that the inclusion of these new human iPSC-derived data, together with the revised wording, adequately addresses the referee's concerns and presents a balanced and realistic assessment of the translational potential of STV-C8.

Rebuttal letter for the manuscript:
Creating bottom-up RNA transfer vehicles from synthetic protein assemblies
(2024-09-19965B)

We address here Referee 1's remaining concerns about in vivo data and assembly heterogeneity. The referees' comments are colored in **blue**, while our responses are in **black**.

My central point has been that there are many established ways to deliver nucleic acid into cells and an important advance would require either:

(i) exceptional delivery efficiency to relevant tissues inside a living animal

We respectfully remind the referee that the manuscript already contains extensive in vivo data, including efficient delivery to lung tissue in mice and to skeletal muscle in a pig model. Criterion (i) is therefore directly addressed by the existing evidence. We are not sure what additional in vivo experiments the referee considers necessary. Given that the manuscript already spans a broad experimental range, from in silico design through primary and patient-derived cells to mouse and pig, we respectfully disagree that further in vivo data are required to support our claims. However, if the referee insists, we can provide further in vivo benchmarking confirming the markedly improved delivery efficiency of STV-C8 compared to clinically used LNPs (Rebuttal Fig. 1).

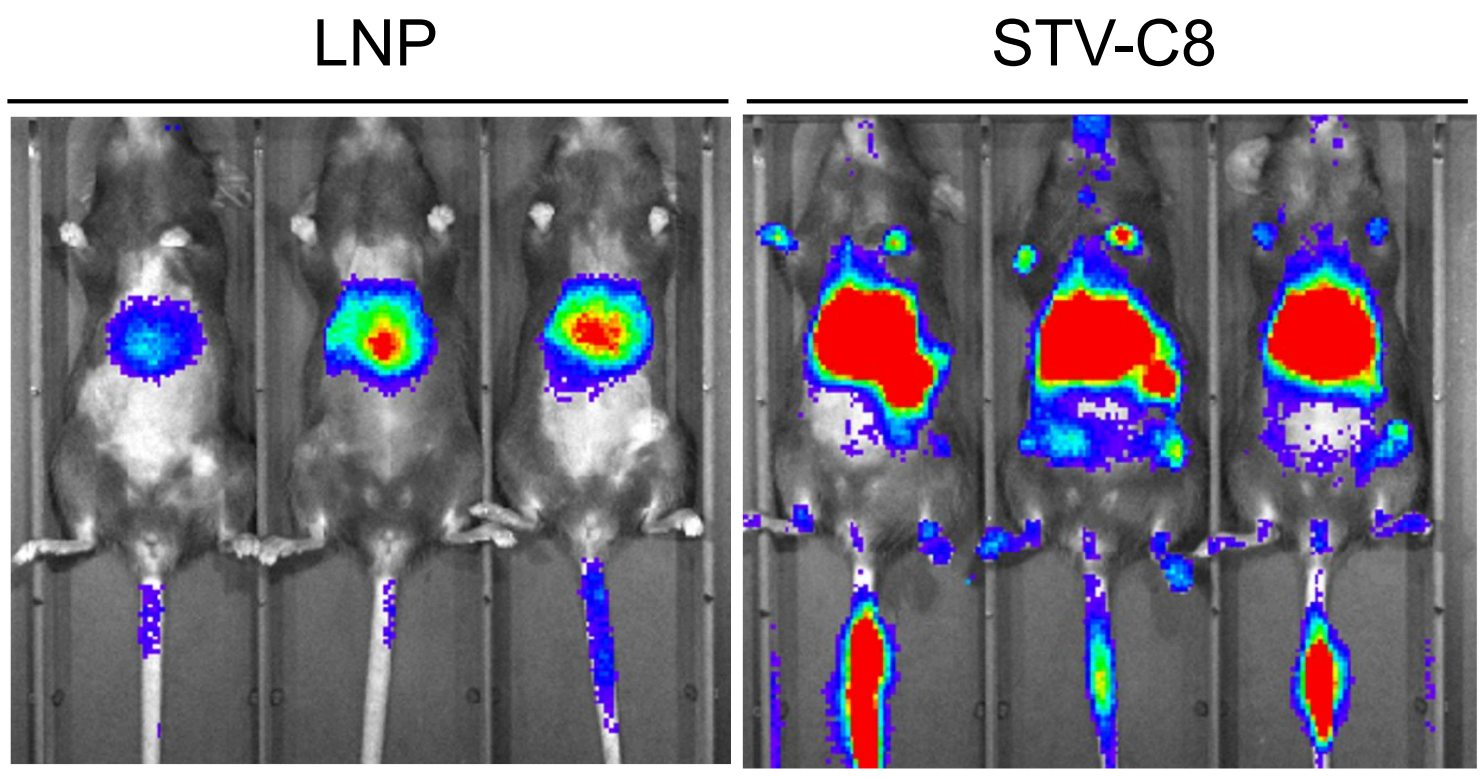

Rebuttal Fig. 1, STV-C8 or LNP-based delivery of Akaluc mRNA upon systemic i.v. injection.

or (ii) substantial progress in building a well-validated sophisticated protein architecture.

As Referee 2 already acknowledged, the main contribution of our study is the unexpected finding that expression of small, engineered, AI-designed protein assemblies, functionalized with membrane binding, budding, and RNA binding domains, is sufficient to drive highly efficient release of extracellular vesicles (Referee 2: "The work is remarkable in many respects and shows that generative AI can be harnessed to engineer protein assemblies with potentially powerfully useful properties, including gene editing in vivo.").

Building on our prior cryo ET work, we have now added new data that directly support the conclusion that the process described is driven by rational engineering of a homogeneous STV-C8 assembly population:

1. Only cells expressing STV-C8 release vesicles (Rebuttal Fig. 2a).

2. Cryo ET reconstructions of these vesicles show the presence of more than a dozen small, highly homogeneous protein assemblies (Rebuttal Fig. 2b).
3. These assemblies closely match the predicted AlphaFold3 structure (Rebuttal Fig. 2c).
4. Quantification of the size distribution of 500 assemblies shows a distinct, narrow distribution centered at 22 ± 1.4 nm and supports that this assembly population drives vesicle release in a size range comparable to that described for viruses (Rebuttal Fig. 2d).
5. Together, these data support a model in which homogeneous STV-C8 assemblies induce extensive vesicle release (Rebuttal Fig. 2e).

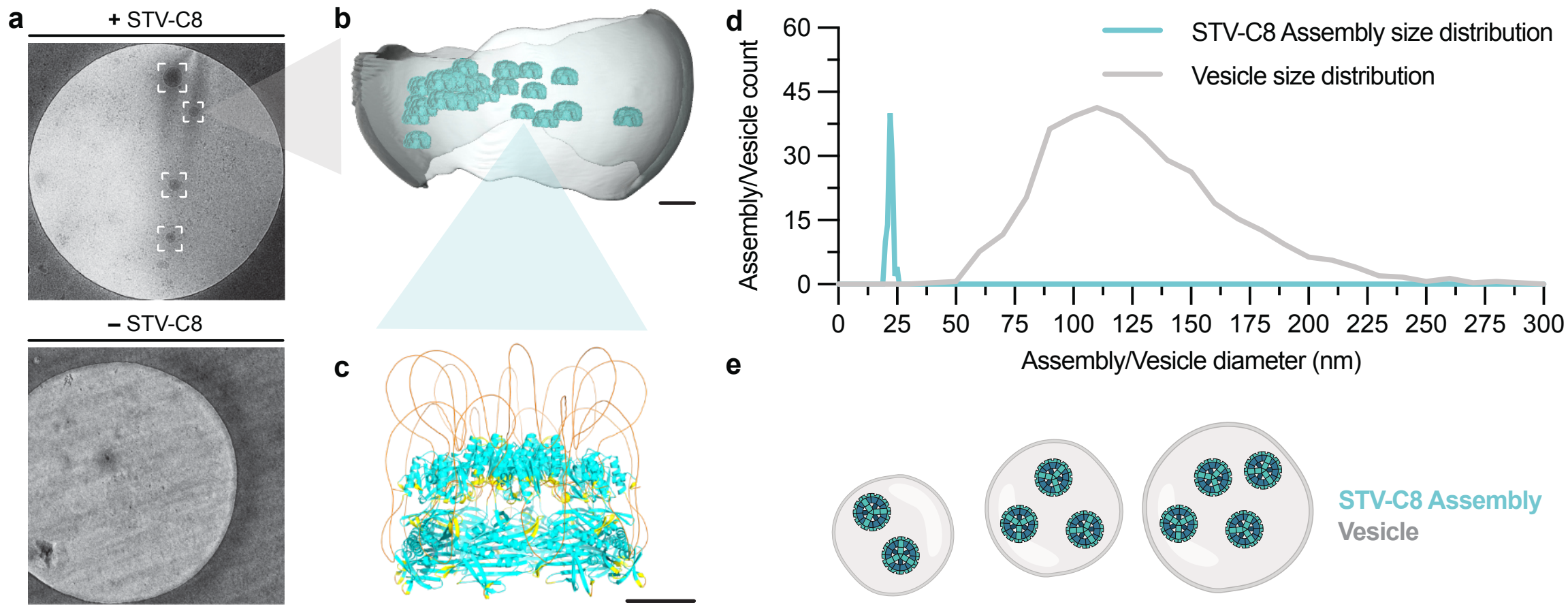

Rebuttal Fig. 2, Characterization of STV-C8 assembly homogeneity. a, Imaging of concentrated supernatants from STV-C8 transfected and untransfected cells by cryo EM. b, Segmentation of an STV-C8 vesicle tomogram (scale bar: 20 nm). c, AlphaFold3 structure prediction of the STV-C8 assembly (scale bar: 5 nm). d, Size distribution of different STV-C8 assemblies (cyan) and vesicles (grey) on EM grids (500 assemblies and 1,100 vesicles analyzed in total). e, Schematic representation of STV-C8 assembly-dependent vesicle formation.

We agree and also confirm here that assembly homogeneity is crucial for the engineered STV system. Vesicle formation, instead, is an inherently stochastic biological process and cannot be fully controlled. But this is not a concern for the therapeutic applicability, since established chromatographic purification workflows can be used to enrich the desired vesicle fraction, as is routinely done for lentiviral vectors.

Given that the Referee requests "atomic resolution" from our in situ cryo ET data, we would like to clarify a potential misunderstanding about what can be concluded in this setup. In many protein design studies, the designed proteins are expressed and purified, and their structures are validated at high resolution by X-ray crystallography or single-particle cryo-EM. That conventional approach is not feasible here because the engineered assembly functions in a cellular context, binds membranes, and is encapsulated within vesicles. Structural interrogation, therefore, requires in situ cryo ET, a method for which atomic resolution is not achievable with current technologies.

Exemplarily, recently published *Nature* papers that focused primarily on in situ structural characterization do not reach atomic resolution in comparable settings (Huang, Y., et al., Nature 633, 224–231 (2024); Wagner, J., et al., Nature 633, 459–464 (2024)). While our cryo ET approach reaches excellent resolution, we view an additional degree of structural depth as beyond the scope of a manuscript whose primary goal is not exhaustive structural determination of a single particle, but rather to establish a general strategy by which AI-designed protein assemblies can be harnessed for RNA delivery. In ongoing work, we are

establishing a screening platform for more than 10,000 STV designs to define quantitative design criteria for optimal vehicles. This scale exceeds prior vehicle campaigns by a wide margin and, in our view, opens a new research direction for genetically encoded delivery vehicles for which we lay the decisive groundwork in this manuscript.

While we acknowledge the critical perspective of Referee 1, we do not see a discrepancy between our data and the conclusions stated in the manuscript, and we hope the referee will agree with this assessment based on this additional clarification. We would also like to thank Referees 2 and 3 again for their constructive input, which helped clarify important aspects of the work.

3rd revision of the manuscript:
Creating bottom-up RNA transfer vehicles from synthetic protein assemblies
(2024-09-19965C)

Referees' comments are colored in **blue**, while our responses are colored in **black**.
Passages added to the manuscript are marked in yellow.

My central point has been that there are many established ways to deliver nucleic acid into cells and an important advance would require either:

(i) exceptional delivery efficiency to relevant tissues inside a living animal

We respectfully remind the referee that the manuscript already contains extensive *in vivo* data, including efficient delivery to lung tissue in mice and to skeletal muscle in a pig model. Criterion (i) is therefore directly addressed by the existing evidence. Given that the manuscript already spans a broad experimental range, from *in silico* design through primary and patient-derived cells to mouse and pig, we respectfully question whether further *in vivo* data are required to support our claims. However, we added additional *in vivo* benchmarking data to the manuscript, confirming the markedly improved delivery efficiency of STV-C8 compared to clinically used LNPs (Supplementary Fig. 3).

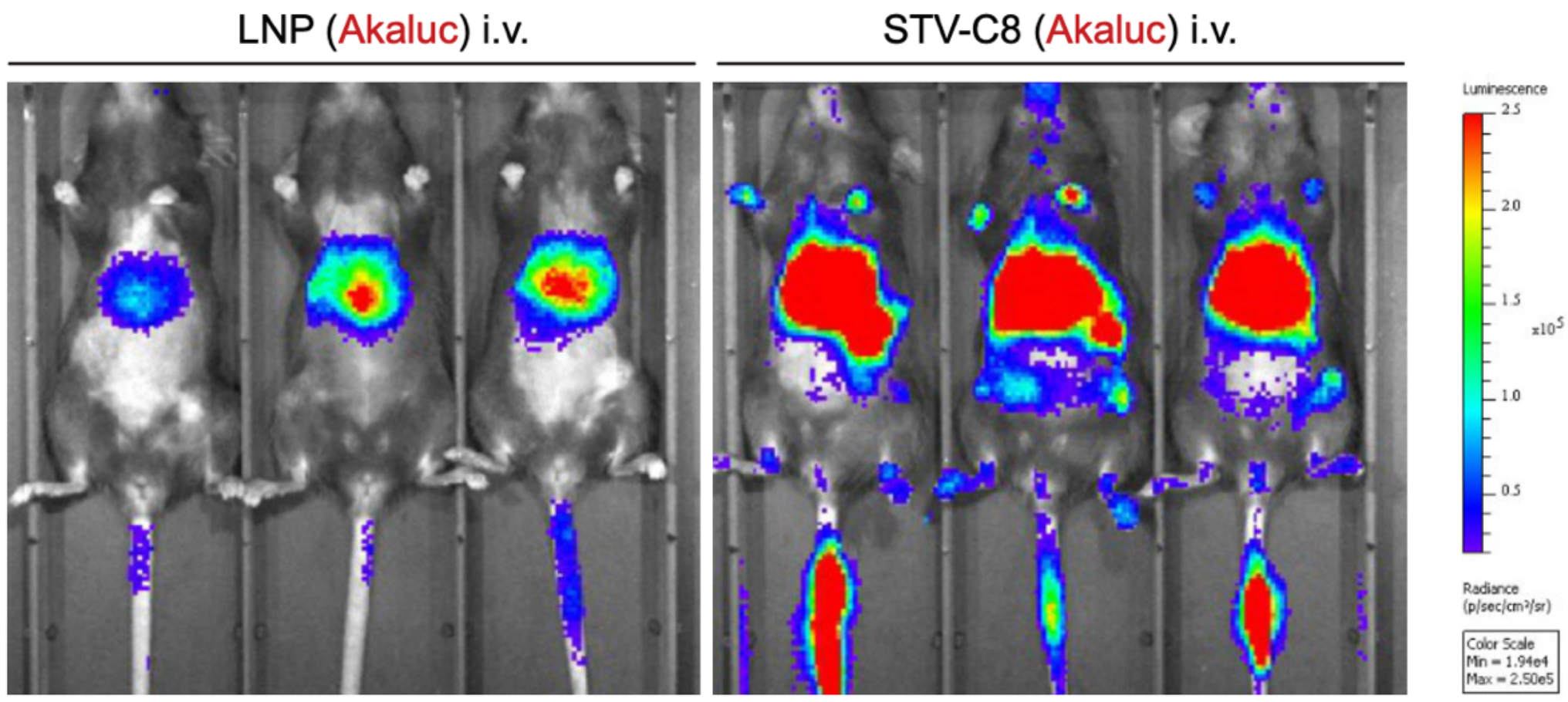

Supplementary Fig. 3 | in vivo comparison of STV-C8 and LNP-mediated mRNA delivery. Female C57BL/6 mice were intravenously injected with LNPs or STV-C8 carrying Akaluc mRNA and imaged 24 h later after substrate administration.

or (ii) substantial progress in building a well-validated sophisticated protein architecture.

We would like to clarify that the main contribution of our study is not the characterization of a single STV design, but the unexpected finding that small, engineered, AI-designed protein assemblies, functionalized with membrane-binding, budding, and RNA-binding domains, are sufficient to drive highly efficient vesicle release (Referee 2: "*The work is remarkable in many respects and shows that generative AI can be harnessed to engineer protein assemblies with potentially powerfully useful properties, including gene editing in vivo.*").

However, we have added new data, further supporting the conclusion that the process described is driven by rational engineering of a homogeneous STV-C8 assembly population. In addition, we have refined our previous analysis and clarify in the following four steps our reasoning:

1. Only cells expressing STV-C8 release vesicles. To make this clearer, we replaced the previous medium-resolution image with a higher-resolution one that better shows the vesicles released from STV-C8-transfected cells (Extended Data Fig. 6a).
2. Cryo ET reconstructions of vesicles show the presence of more than a dozen small, highly homogeneous protein assemblies (Fig. 2e). We would like to remind the referee that the cryo ET reconstruction shown in Fig. 2e was not only generated from the single image in the same panel, but from a tilt series of images that were aligned and reconstructed into a 3D tomogram using widely accepted methods and algorithm.
3. We used AlphaFold3 to gain additional insight into the STV-C8 assembly structure, because atomic-resolution structure determination of such vesicle-associated assemblies is hardly achievable with current in situ cryo ET methods (please see a detailed description of this technical limitation below). The predicted structure has a high-confidence pLDDT score above 80 and closely matches the shape and size of the assemblies observed by cryo-ET (Extended Data Fig. 6d, f).
4. We added new data that confirms assembly uniformity within vesicles by quantifying the size distribution of 500 assemblies. This analysis shows a distinct, narrow distribution centered at 22 ± 1.4 nm, supporting the well-controlled engineering of the STV-C8 assembly (Extended Data Fig. 6b).

We agree and also confirm that **assembly** homogeneity is crucial for the engineered STV system. Derived **vesicle** formation, instead, is an inherently stochastic biological process and cannot be fully controlled. But this is not a concern for the therapeutic applicability, since established chromatographic purification workflows can be used to enrich the desired vesicle fraction, as it is routinely done for lentiviral vectors.

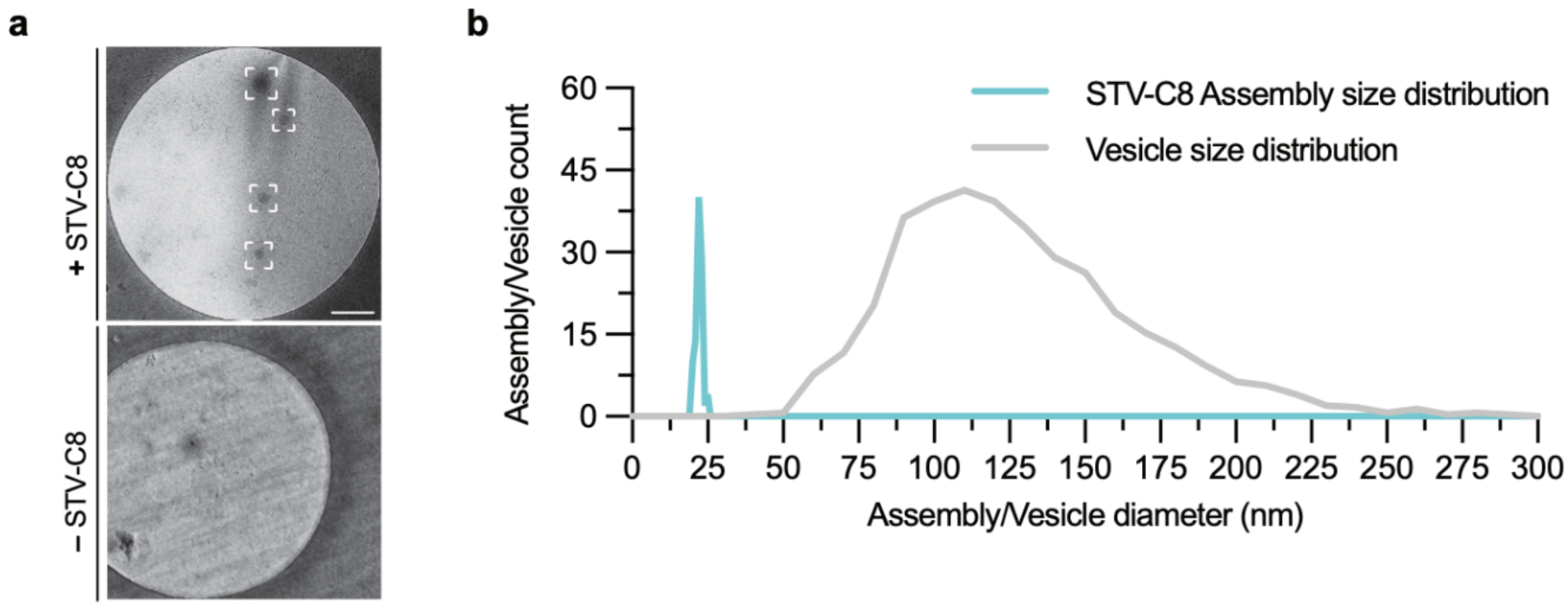

Extended Data Fig. 6a, Zoomed-in region on the EM grid (scale bar: 500 nm) of concentrated supernatants from STV-C8 transfected or untransfected cells. Representative STV-C8 vesicles are indicated by white squares. **b,** Size distribution of STV-C8 assemblies within vesicles and vesicles on EM grids (500 assemblies and 1,100 vesicles analyzed).

Given that the Referee requests "atomic resolution" from our in situ cryo ET data, we would like to clarify a potential misunderstanding about what can be concluded in this setup. In many protein design studies, the designed proteins are expressed and purified, and their structures are validated at high resolution by X-ray crystallography or single-particle cryo-EM. That conventional approach is not feasible here because the engineered assembly functions in a cellular context, binds membranes, and is encapsulated within vesicles. Structural interrogation, therefore, requires in situ cryo ET, a method for which atomic resolution is not achievable with current technologies. Exemplarily, recently published *Nature* papers that focused primarily on *in situ* structural characterization do not reach atomic resolution in

comparable settings (Huang, Y., et al., Nature 633, 224–231 (2024); Wagner, J., et al., Nature 633, 459–464 (2024)).

While our cryo ET approach reaches excellent resolution, we view an additional degree of structural depth as beyond the scope of a manuscript whose primary goal is not exhaustive structural determination of a single particle, but rather to establish a general strategy by which AI-designed protein assemblies can be harnessed for RNA delivery. In ongoing work, we are establishing a screening platform for more than 10,000 STV designs to define quantitative design criteria for optimal vehicles. This scale exceeds prior vehicle campaigns by a wide margin and, in our view, opens a new research direction for genetically encoded delivery vehicles for which we lay the decisive groundwork in this manuscript.

Beyond the two main concerns raised by Referee 1, we would also like to respond to the referee's additional comments:

Here there has been further progress of cell transduction ex vivo, which does not meet criterion 1, since optimized delivery to muscle inside an animal is the key challenge for the field. Some degree of delivery to muscle for DMD in living animals has been shown for more than 20 years, e.g. https://pubmed.ncbi.nlm.nih.gov/9239410/, https://pubmed.ncbi.nlm.nih.gov/11812282/

We acknowledge the referee's point that optimized systemic delivery to skeletal muscle *in vivo* remains a major challenge for the DMD field, and we agree that our study does not provide a complete therapeutic solution for DMD. This was not the intended scope of our work, as stated in the manuscript ("*While acknowledging that intramuscular delivery is not directly applicable to DMD and that future studies will need to address comparative performance, targeting, and cell-type specificity, the successful deletion of exon 51 in a large animal model and DMD patient-derived skeletal muscle cells, combined with its beneficial safety profile, emphasizes the strong clinical potential of STV-C8*" (line 350)). We therefore present the DMD experiments as a disease-relevant proof of principle, not as a solution to systemic muscle delivery. The central advance of our work is more relevant for the broad readership of *Nature*: we show that simple, compact, AI-designed synthetic protein assemblies can function as potent RNA delivery vehicles for diverse cargos.

This revision does not provide any evidence that supports that the design goals have been met, for criterion 2. The data further support a highly heterogeneous mix of unknown composition. Validation of the protein engineering aspect of the paper and the integrity of the assembly depends on Fig. 2e and Ext. Data Fig. 6. Fig. 2e shows a clear high contrast membrane and heterogeneous low contrast contents of the vesicle.

We respectfully disagree with the referee's interpretation that the data support a highly heterogeneous mixture of unknown composition. To address this concern, we have added the mentioned assembly-size distribution analysis, which shows a distinct assembly-population at 22 ± 1.4 nm. We would also like to clarify that the cryo-ET reconstruction shown in Fig. 2e was not generated from a single 2D image, but from a full tilt series that was computationally aligned and reconstructed into a three-dimensional tomogram, as described in the Methods section. This is a standard approach in the field and provides substantially more structural information than an individual projection image. As explained above, we agree that assembly homogeneity is critical for the engineered STV system and is achieved in our design. In contrast, biologically derived vesicles are inherently heterogeneous due to the stochastic nature of vesicle formation. This is not a limitation for therapeutic application, as established purification workflows can enrich the desired vesicle fraction. We hope this

clarifies the apparent conflation of STV-C8 assembly homogeneity with the natural heterogeneity of biologically derived vesicles.

Ext. Data Fig. 6a shows weak signal to noise and high variability of the proposed vesicles.

We apologize for the suboptimal image quality in the previous version. We have now replaced Extended Data Fig. 6a with a higher-contrast image that more clearly shows vesicles in STV-C8-producing cells, but not in the corresponding control cells. As mentioned, vesicle release is a biological process that introduces a degree of variability. The vesicle-inducing engineered STV-C8 assemblies, in contrast, are highly homogeneous.

Ext. Data Fig. 6e does not strengthen my confidence in their assembly; the data show a long smear of assemblies, consistent with highly heterogeneous assemblies. Indeed there are some bands there as a small proportion of the total sample. Additionally it is hard to be sure what molecular weight the bands relate to, because native PAGE mobility is not proportional to molecular weight but a relationship between shape, size and charge.

The native PAGE data show the expected complexity of a biologically produced vesicle preparation, in which additional co-packaged proteins contribute to certain background signal, as our mass spectrometry data confirmed already (Fig. 2d). Importantly, distinct bands corresponding to discrete assembly states are clearly detectable, as expected for a multimeric protein assembly.

We agree with the referee that native PAGE mobility is not determined by molecular weight alone, but reflects a combination of size, shape, charge, and assembly state. We therefore did not claim to identify a precise molecular mass. Rather, we used it as qualitative orthogonal evidence supporting the presence of discrete higher-order STV-C8 assemblies.

Ext. Data Fig. 6f appears only to be wishful thinking. Lots of shapes would fit into the vesicle tomogram, and this figure inserts what the authors would like it to be, without any evaluation of how well it fits or what else could fit. I could not recommend publication of this interpretation of this figure section at any journal.

We respectfully disagree with the referee's characterization of Extended Data Fig. 6f as "*wishful thinking*". We clearly describe the methodological approach in the methods section to reach this conclusion and cite relevant literature, which is widely accepted in the cryo-ET community. We consider our experimental design and interpretation to be valid, as the reviewer did not provide any indication of errors in the choice of experimental procedures or of any specific technical flaws.

I acknowledge that the authors have made substantial efforts but the central points from my previous review have not been addressed. In fact the extra data strengthen my assessment that the manuscript's interpretation of achieving the desired structural organization is not supported by the data.

We thank the referee for acknowledging our efforts. Unfortunately, the remaining criticism has not been accompanied by concrete guidance on which experimental approach should be taken to convince the referee. Although we disagree with the referee's interpretation on some points, we thank the referee for the critical comments, which led us to refine our analysis and strengthen our assessment of assembly homogeneity.

Overall, we believe that, over the course of the revision process, we have addressed the concerns raised by the referee (e.g. benchmarking and gene editing quantification). We agree that the referee's request for atomic-resolution information on the STV-C8 assembly would be interesting. However, such structural resolution for vesicle-associated assemblies is currently hardly achievable with today's cryo-ET methods. We hope that the referee acknowledges this technical limitation and agrees with our assessment that the core contribution of our study lies elsewhere: AI-designed protein assemblies can be used to generate potent RNA delivery vehicles, opening a path toward rationally designed delivery systems.

Referee #2 (Remarks to the Author):

The authors have addressed my concerns.

We would like to thank Referee 2 again for the helpful comments, which allowed us to strengthen the manuscript, including the benchmarking experiments.

Referee #3 (Remarks to the Author):

The authors have nicely addressed my concerns.

We would like to thank Referee 3 again for the helpful input, which helped us clarify important aspects of the work, including the potential immunogenicity of STV-C8.

4th revision of the manuscript:
Creating bottom-up RNA transfer vehicles from synthetic protein assemblies
(2024-09-19965D)

The referees' remaining comment is colored in **blue**, while our responses is colored in **black**. The revised parts in the main text of the manuscript are highlighted in yellow.

Referee #1:
The authors have added additional mouse imaging of high efficiency of mRNA delivery compared to LNP delivery, although without describing to what extent this fits desired tissue tropism. They have also performed further quantification of STV-C8 assemblies by EM from concentrated supernatant, which supports the formation of particles in the desired size range. CryoET data, as is intrinsic to the method, are only able to give low resolution information on the particle assembly and the authors have not added further statistics or quantification of uncertainty of fit to protein models and with RNA segmentation. Hence the data support the generation of designed particles with high delivery efficiency in different preclinical models, without the ability to validate details of the structural organisation because of the difficulty of the analytical systems.

Our data suggest the formation of particles consistent with the intended STV-C8 design. However, as the referee correctly noted, *in situ* cryo-ET is inherently limited in the structural resolution it can provide. To address this point, we have now included a limitation statement in line 202 of the manuscript. We appreciate the referee's assessment that this limitation reflects the inherent limits of the method rather than a technical flaw in our analysis.

We also thank the referee for the important comments raised throughout the revision process. These points helped us improve the quality and clarity of the manuscript.
